# Metalloradical activation of α-formyldiazoacetates for the catalytic asymmetric radical cyclopropanation of alkenes†
†Electronic supplementary information (ESI) available. CCDC 1532256. For ESI and crystallographic data in CIF or other electronic format see DOI: 10.1039/c7sc00658f



**DOI:** 10.1039/c7sc00658f

**Published:** 2017-03-31

**Authors:** Xue Xu, Yong Wang, Xin Cui, Lukasz Wojtas, X. Peter Zhang

**Affiliations:** a Department of Chemistry , Merkert Chemistry Center , Boston College , Chestnut Hill , Massachusetts 02467 , USA . Email: peter.zhang@bc.edu; b Department of Chemistry , University of South Florida , Tampa , FL 33620 , USA

## Abstract

For the first time, α-formyldiazoacetates have been successfully applied for the asymmetric cyclopropanation of alkenes *via* Co(ii)-based metalloradical catalysis. The cobalt(ii) complex of the *D*_2_-symmetric chiral amidoporphyrin [Co(3,5-Di^*t*^Bu-ChenPhyrin)] is an effective metalloradical catalyst that can activate α-formyldiazoacetates to cyclopropanate both aromatic and aliphatic olefins with varied electronic properties, affording the synthetically useful 1,1-cyclopropaneformylesters in high yields with both high diastereo- and enantioselectivity.

## Abstract 2

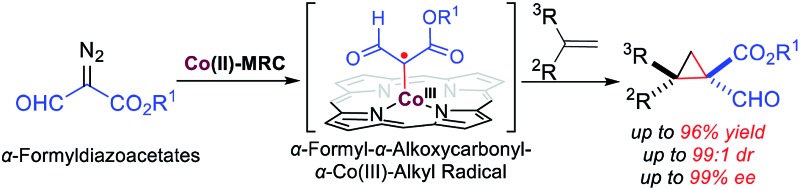
For the first time, α-formyldiazoacetates (FDA), have been successfully applied for asymmetric olefin cyclopropanation *via* Co(ii)-based metalloradical catalysis.

## Introduction

**Scheme 1:**
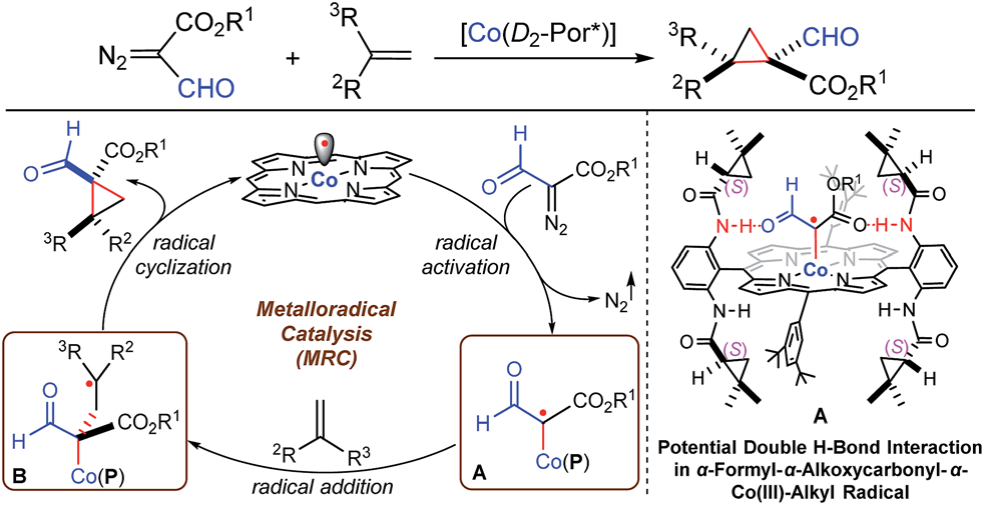
Working proposal for the radical cyclopropanation of alkenes with FDA *via* Co(ii)-MRC.

Radical reactions have been increasingly exploited as attractive tools in modern organic synthesis as they exhibit a number of unique features, including tolerance of functional groups.^1^ To address the existing challenges such as the control of enantioselectivity,^2^ metalloradical catalysis (MRC) has rendered a fundamentally new approach that enables the catalytic generation of metal-stabilized organic radicals as well as the selective control of their subsequent radical reactions.^3,4^ As stable metalloradicals, cobalt(ii) complexes of the *D*_2_-symmetric chiral amidoporphyrins [Co(*D*_2_-Por*)] have emerged as a class of effective catalysts for asymmetric olefin cyclopropanation through a distinct radical process involving the catalytic generation of α-metalloalkyl radicals as the key intermediates (Scheme 1: **A**).^5^ It has been suggested that the unusual capability of [Co(*D*_2_-Por*)] in activating acceptor/acceptor-substituted diazo reagents as well as regulating the reactivity and selectivity of the radical processes is further enhanced by the postulated double hydrogen-bonding interactions between the amide N–H donors on the amidoporphyrin ligand and the two acceptors on the C-centered radical moiety (Scheme 1).^5e,g,h^ Considering the demonstrated functional group tolerance of Co(ii)-based metalloradical catalysis (Co(ii)-MRC),^5d,e,j–l^ we were attracted to the possibility of accessing a new type of α-metalloalkyl radical bearing both α-formyl and α-alkoxycarbonyl functionalities from the metalloradical activation of α-formyldiazoacetates (FDA).^6^ Despite the fact that free α-formylalkyl radicals are scarce and prone to H-atom abstraction because of the weak aldehydic C–H bonds,^7^ we reasoned that this type of α-metalloalkyl radical might be accessible on the basis of the combined effects of metal stabilization, double H-bonding interaction, and protection by the well-defined cavity of the ligand system (Scheme 1: **A**). Assuming that the α-formyl-α-alkoxycarbonyl-α-Co(iii)-alkyl radicals (**A**) are capable of undergoing stereoselective radical addition with olefins, followed by the effective 3-*exo*-tet radical cyclization^8^ of the corresponding γ-Co(iii)-alkyl radicals (**B**), we anticipated the potential development of a new catalytic process for the asymmetric synthesis of optically active cyclopropanes bearing both aldehyde and ester functionalities, which would be valuable for stereoselective organic synthesis (Scheme 1).^9^

The catalytic asymmetric cyclopropanation of alkenes with diazo reagents represents the most general approach for the stereoselective synthesis of optically active cyclopropanes.^10^ While a number of diazo reagents have been successfully employed, there is no previous report of a catalytic system that is effective for asymmetric olefin cyclopropanation with α-formyldiazoacetates (FDAs). The development of this catalytic process apparently confronts the formidable challenges associated with the inherent low reactivity of the acceptor/acceptor-substituted diazo reagents and the incompatibility of the aldehyde functionality with existing catalytic systems.^11^ Recently, Fokin and coworkers developed a Rh_2_-catalyzed system for a highly asymmetric cyclopropanation with *N*-sulfonyl-1,2,3-triazoles for the production of cyclopropyl imines, which could be subsequently transformed into the corresponding formyl cyclopropane derivatives.^12^ While this offers a valuable alternative for the preparation of optically active formyl cyclopropanes, the direct synthesis *via* asymmetric cyclopropanation with α-formyl diazo reagents is an appealing process that remains to be developed. As a new application of Co(ii)-MRC, we herein wish to report the first catalytic system based on [Co(*D*_2_-Por*)] that is highly effective in activating FDA for asymmetric cyclopropanation. This asymmetric radical process is generally applicable for a broad scope of alkenes, offering a direct method for the high-yielding synthesis of 1,1-cyclopropaneformylesters with excellent control of the diastereo- and enantioselectivity. The products can be readily transformed into other chiral 1,1-bifunctionalized cyclopropanes and chiral dihydrofurans.

## Results and discussion

Initial experiments were carried out with styrene as the model substrate to examine the suitability of FDA for the catalytic radical cyclopropanation by Co(ii)-MRC (Table 1). While [Rh_2_(OAc)_4_] was indeed incompatible (entry 1), [Co(TPP)] only produced trace amounts of the corresponding cyclopropane from ethyl α-formyldiazoacetate (EFDA) (entry 2). Remarkably, when the Co(ii) complex of the *D*_2h_-symmetric achiral amidoporphyrin [Co(**P1**)]^13^ was used as the catalyst, the reaction proceeded successfully to form the desired (*E*)-1,1-cyclopropaneformylester in a 46% yield (entry 3). The dramatic difference in the catalytic activity between [Co(TPP)] and [Co(**P1**)] is in alignment with the hypothesized role of the double H-bonding interaction in activating EFDA and stabilizing the resulting intermediate **A** (Scheme 1). By switching to [Co(**P2**)],^14^ the reactivity was further enhanced with the observation of a significant level of enantioselectivity (entry 4). Of the solvents examined, toluene was proven to be the medium of choice (entries 4–8). Lowering the reaction temperature further increased the enantioselectivity, but decreased the yield (entries 8–10). The diastereoselectivity was greatly improved when the bulkier *tert*-butyl α-formyldiazoacetate (*t*-BFDA) was used, affording cyclopropane **1a** in a 78% yield with 95 : 5 dr and 96% ee (entry 11). The product yield could be further improved to 84% by increasing the catalyst loading to 5 mol% while maintaining the high level of diastereo- and enantioselectivity (entry 12).

**Table 1:** The catalytic asymmetric cyclopropanation of styrene with FDA^*a*^

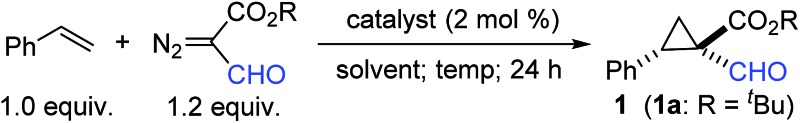							
Entry	Catalyst	R	Solvent	Temp. (°C)	Yield^*b*^ (%)	(*E*) : (*Z*)	ee^*c*^ (%)
1	[Rh_2_(OAc)_4_]	Et	DCM	60	0	—	—
2	[Co(TPP)]	Et	DCM	60	<10^*d*^	—	—
3	[Co(**P1**)]	Et	DCM	60	46	84 : 16	—
4	[Co(**P2**)]	Et	DCM	60	81	80 : 20	81
5	[Co(**P2**)]	Et	EtOAc	60	81	80 : 20	81
6	[Co(**P2**)]	Et	PhCl	60	73	82 : 18	83
7	[Co(**P2**)]	Et	Hexanes	60	73	80 : 20	82
8	[Co(**P2**)]	Et	Toluene	60	86	85 : 15	86
9	[Co(**P2**)]	Et	Toluene	40	73	86 : 14	90
10	[Co(**P2**)]	Et	Toluene	RT	60	87 : 13	93
11	[Co(**P2**)]	^*t*^Bu	Toluene	40	78	95 : 5	96
**12** ^*e*^	**[Co(** **P2** **)]**	^***t***^ **Bu**	**Toluene**	**40**	**84**	**95** **:** **5**	**96**
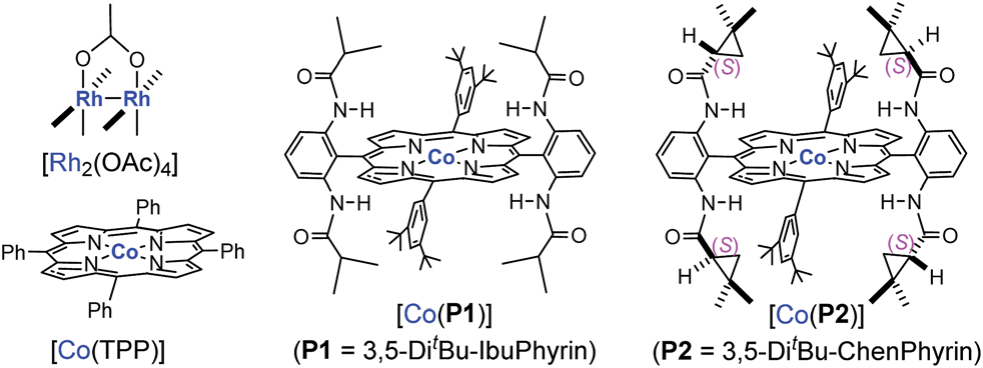							

^*a*^Carried out in one-portion under N_2_ with [olefin] = 0.20 M.

^*b*^Isolated yields.

^*c*^ee of major (*E*)-diastereomer determined by chiral HPLC.

^*d*^Determined by ^1^H-NMR.

^*e*^With 5 mol% of catalyst for 20 h.

Under the optimized conditions, the scope of this Co(ii)-based asymmetric radical cyclopropanation was investigated (Table 2). Like styrene, its derivatives bearing substituents with varied electronic and steric properties could be cyclopropanated by [Co(**P2**)] with *t*-BFDA. For example, *p*- and *m*-alkyl styrenes were cyclopropanated to formylcyclopropanes **1b–1d** in high yields with excellent diastereo- and enantioselectivity (entries 1–3). Halogenated (entries 4–6) and electron-deficient (entries 7 and 8) styrene derivatives could also undergo high-yielding cyclopropanation, producing **1e–1i** with high stereoselectivities. The configurations of the two contiguous chiral centers in **1h** were established as [1*R*,2*S*] by X-ray crystal structural analysis (see ESI†). The cyclopropanation was also suitable for other aromatic olefins as exemplified with 2-naphthalene for near quantitative formation of cyclopropane **1j** (entry 9). In addition, 1,1-disubstituted olefins such as α-methylstyrene could also be effectively employed, affording (*E*)-formylcyclopropane **1k** in a 93% yield with remarkable control of both the diastereo- and enantioselectivity of the two newly-generated contiguous all-carbon quaternary stereogenic centers (entry 10). To demonstrate the functional group tolerance of the Co(ii)-based radical cyclopropanation, *m*-formylstyrene could be effectively cyclopropanated to cyclopropane **1l** in a high yield with high diastereo- and enantioselectivity (entry 11). Notably, the two unprotected formyl groups were well tolerated by the metalloradical system. It is also worth mentioning that the Co(ii)-catalyzed cyclopropanation process could be scaled up ten-fold as demonstrated with the high-yielding synthesis of the cyclopropane **1d** on a 1.0 mmol scale without affecting the excellent stereoselectivity (entry 3).

**Table 2:** The asymmetric cyclopropanation of alkenes with *t*-BFDA by [Co(**P2**)]^*a*^ ^,^ ^*b*^ ^,^ ^*c*^

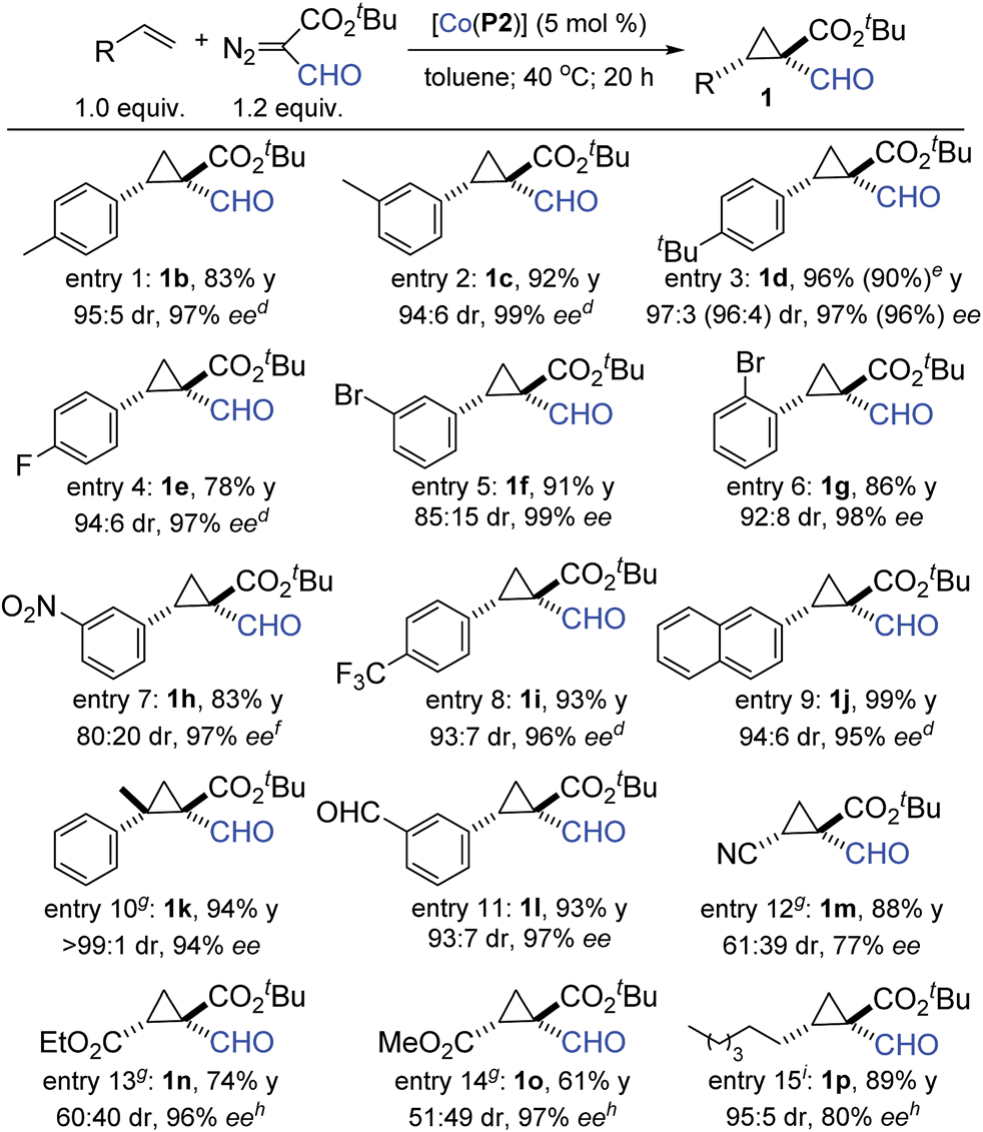

^*a*^Carried out in one-portion under N_2_ with [olefin] = 0.20 M.

^*b*^Isolated yields.

^*c*^ee of major (*E*)-diastereomer determined by chiral HPLC.

^*d*^ee determined upon derivatization.

^*e*^Results in the parentheses were obtained for the reaction performed on a 1.0 mmol scale.

^*f*^Absolute configuration determined by X-ray diffraction as [1*R*,2*S*].

^*g*^5 equiv. olefin.

^*h*^ee determined by chiral GC.

^*i*^Neat condition.

The Co(ii)-based radical cyclopropanation was further highlighted for its exceptional reactivity toward electron-deficient olefins, which are typically problematic substrates for catalytic systems involving electrophilic metallocarbene intermediates. For example, [Co(**P2**)] could catalyze the C=C cyclopropanation of acrylonitrile with *t*-BFDA to form 1,1,2-cyclopropaneformylesternitrile **1m** in a high yield with good enantioselectivity (entry 12), leaving the cyano group untouched. In marked contrast, when treated with Rh_2_-based catalyst, acrylonitrile was previously shown to react with the C≡N bond of FDA to form oxazoles.^15^ Other electron-deficient olefins such as ethyl and methyl acrylates could also be cyclopropanated to form the 1,1,2-cyclopropaneformyldiesters **1n** and **1o** in good yields with 96% ee and 97% ee, respectively, although with diminished control of diastereoselectivity (entries 13 and 14). The presence of three electron-withdrawing groups in the cyclopropanes **1m–1o** renders them highly electrophilic, making them valuable intermediates for synthetic applications.^16^ Furthermore, aliphatic olefins, another class of challenging substrate for asymmetric cyclopropanation, could also be cyclopropanated by [Co(**P2**)] as exemplified by the high-yielding reaction of 1-octene under neat condition, forming **1p** with high stereoselectivity (entry 15).

As an initial exploration of applications, the formyl unit of the resulting chiral 1,1-cyclopropaneformylesters could be readily converted into other functional groups, forming various cyclopropane derivatives while retaining high enantiopurity. For example, the formyl group in (*E*)-**1a** could be transformed into a *trans*-vinyl unit *via* the Horner–Wadsworth–Emmons reaction, affording (*E*)-1,1-cyclopropanevinylester **2** in a 78% yield with full retention of both the relative and absolute configurations (eqn (1)). When treated with Bestmann–Ohira reagent, the formyl group in (*E*)-**1a** could be smoothly converted to a terminal alkyne functionality, resulting in chiral (*E*)-1,1-cyclopropaneethynylester **3** in a 70% yield without any diminishment of the original stereochemistry (eqn (2)). This transformation provides an alternative way to direct asymmetric cyclopropanation with α-ethynyldiazoacetates for chiral 1,1-cyclopropaneethynylesters.^17^ It is noted that α-ethynyldiazoacetates containing terminal alkyne units seem synthetically inaccessible. While the [Co(**P2**)]-catalyzed cyclopropanation with FDA generally forms (*E*)-cyclopropanes, the (*Z*)-diastereoisomers could be conveniently accessed through the stereospecific epimerization previously reported.^5*e*^ As demonstrated with (*E*)-**1a**, treatment with 5 equivalents of NaI at room temperature resulted in the formation of (*Z*)-**1a** as the major diastereomer with only partial loss of the original optical purity (eqn (3)). Interestingly, when (*E*)-**1g** was treated with 10 equivalents of NaI at an elevated temperature, a ring-expansion involving the formyl group occurred instead, affording 2,3-dihydrofuran **4** in a 74% yield (eqn (4)). In the absence of any external chiral induction, the enantiopurity appeared to be largely retained during the rearrangement.
1

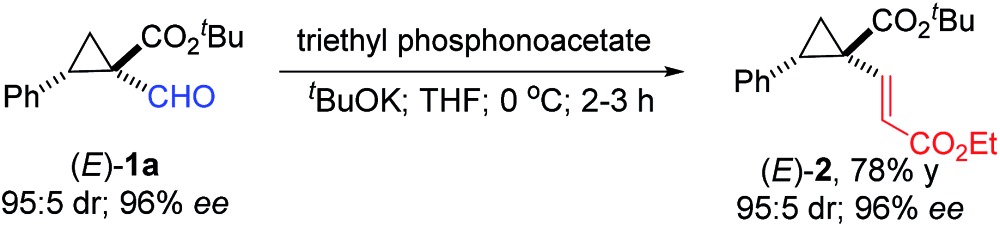



2

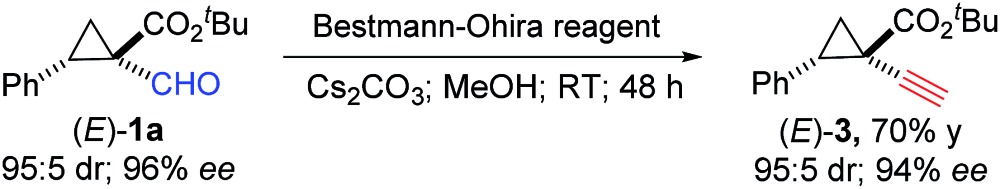



3

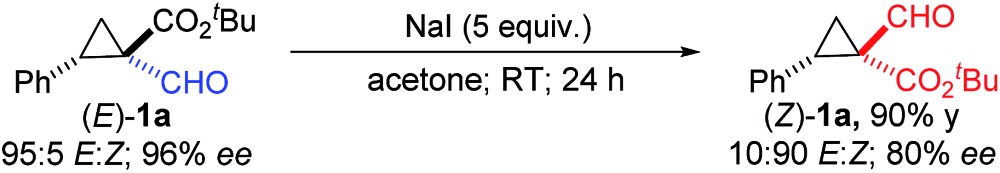



4

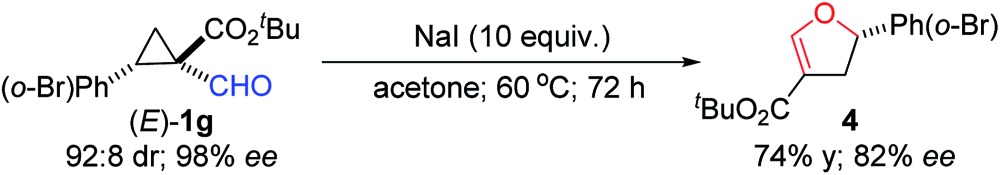




## Conclusions

In summary, we have demonstrated that the metalloradical catalyst [Co(**P2**)] can effectively activate α-formyldiazoacetates (FDAs) for a highly asymmetric olefin cyclopropanation, without affecting the otherwise reactive aldehyde functionality. This represents the first application of α-formyldiazo reagents for metal-catalyzed asymmetric cyclopropanation. The Co(ii)-based radical cyclopropanation with FDA can be successfully applied to a broad scope of olefin substrates, permitting the direct synthesis of chiral 1,1-cyclopropaneformylesters in high yields with high diastereo- and enantioselectivity. Given that the resulting enantioenriched cyclopropanes contain two contiguous chiral centers in the ring structure, including one all-carbon quaternary stereogenic center bearing both aldehyde and ester functionalities, this new Co(ii)-based asymmetric radical cyclopropanation process should find wide applications in stereoselective organic synthesis.
