# Supplementary material for: Metalloradical activation of α-formyldiazoacetates for the catalytic asymmetric radical cyclopropanation of alkenes
Source: Chem Sci. 2017 Mar 31;8(6):4347–51. doi: 10.1039/c7sc00658f (PMC5605771; doi:10.1039/c7sc00658f)
Supplement: Supplementary file 1 [file SC-008-C7SC00658F-s001.pdf]

Electronic Supplementary Material (ESI) for Chemical Science.
This journal is © The Royal Society of Chemistry 2017

Supporting Information

Metalloradical Activation of *α*-Formyldiazoacetates for Catalytic Asymmetric Radical Cyclopropanation of Alkenes

Xue Xu,[†] Yong Wang,[§] Xin Cui,[†] Lukasz Wojtas,[†] and X. Peter Zhang*[§†]

[§] Department of Chemistry, Merkert Chemistry Center, Boston College, Chestnut Hill, Massachusetts 02467, United States

† Department of Chemistry, University of South Florida, Tampa, Florida 33620, United States

Table of Contents

I. General Considerations--S2

II. General Procedure for α-Formyldiazoacetates Synthesis--S2

III. General Procedure for the Reduction of Cyclopropane Carbaldehydes------------------------S3

IV. General Procedure for Asymmetric Cyclopropanation--S3

V. Further Transformations of Chiral 1,1-Cyclopropaneformylesters------------------------------S10

VI. X-ray Crystallography--S13

VII. References--S15

X. Spectra---S16

I. General Considerations. All cyclopropanation reactions were carried out under a nitrogen atmosphere in oven-dried glassware following standard Schlenk techniques. Toluene was distilled under nitrogen from sodium benzophenone ketyl. Thin layer chromatography was performed on Merck TLC plates (silica gel 60 F254). Flash column chromatography was performed with ICN silica gel (60 Å, 230-400 mesh, 32-63 μm). Proton and carbon nuclear magnetic resonance spectra (^{1}H NMR and ^{13}C NMR) were recorded on a Bruker250 (250 MHz) or Varian Inova400 (400 MHz) or UnityInova 600 (600 MHz) instrument with chemical shifts reported relative to residual solvent. HPLC measurements were carried out on a Shimadzu HPLC system with Whelk-O1,Chiralcel OD-H, OJ-H, and AD-H columns. GC measurements were carried out on a Shimadzu GCMS system with Chiral GTA and Dex-CB column. HRMS data was obtained on an Agilent 1100 LC/MS ESI/TOF mass spectrometer with electrospray ionization.

II. General Procedure for *α*-Formyldiazoacetates Synthesis:[1]

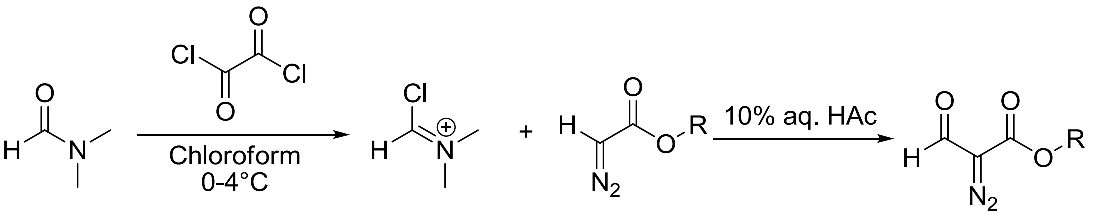

(***Diazo reagents are a class of potentially explosive compounds. Protections must be applied for the operation. Handling of diazo compounds must be done within a fume hood with a safety shield around the reaction. All the diazo reagents used in this work were stored in a freezer at -20˚C***)

In a dried three-necked flask is placed a $CHC1_3$ solution of DMF (1.1 g, 15 mmol/30 ml). While stirring in a freezing mixture, 2.2 g of oxalyl chloride (18 mmol) was added dropwise. After the vigorous evolution of CO and CO_2 has ceased, the mixture was heated to 40 °C for 10 min. The mixture was then cooled to -10 °C and diazoacetate (30 mmol) was added dropwise, maintaining the temperature no higher than 0 °C. After the brisk evolution of N_2 has subsided, the mixture was stirred at r.t. for 1 h. The solution was concentrated, and 100 ml of cold dry Et_2O were added. The formed crystals were filtered immediately, washed successively with dry Et_2O and then dissolved in 10% aq. AcOH. After stirring for 2 h, the mixture was extracted with Et_2O, washed with brine,

dried with Na_2SO_4, filtered, and concentrated, followed by flash silica gel chromatography (pentane/diethyl ether) to afford desired product with around 20% overall yield.

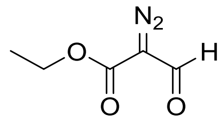

ethyl 2-diazo-3-oxopropanoate[1]**:** 19% yield. ^{1}H NMR (250 MHz, $CDCl_3$): δ 9.63 (s, 1H), 4.29 (dq, *J* = 7.11, 1.32 Hz, 2H), 1.28 (dt, *J* = 7.13, 1.32 Hz, 3H). ^{13}C NMR (62.5 MHz, $CDCl_3$): δ 181.3, 161.2, 61.9, 14.3.

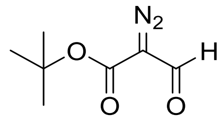

***tert*-butyl 2-diazo-3-oxopropanoate:** 20% yield. ^{1}H NMR (250 MHz, $CDCl_3$): δ 9.66 (s, 1H), 1.52 (s, 9H)**.** ^{13}C NMR (62.5 MHz, $CDCl_3$): δ 181.8, 160.3, 83.9, 28.3. HRMS (ESI) ($[M+Na]^+$) Calcd. for $C_7H_{10}N_2O_3Na$: 193.0584, Found: 193.0586. IR (neat, cm-1): 2139, 1706, 1665, 1370, 1308, 1241, 1119, 783.

III. General Procedure for the Reduction of Cyclopropane Carbaldehydes (*In some cases, a further reduction step is needed for the determination of ee*)

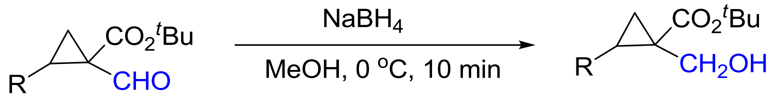

To a stirred at 0 °C solution of cyclopropyl aldehydes (0.1 mmol) in methanol (5 ml) was added dropwise a solution of sodium borohydride (1.2-1.5 equiv.) in methanol (1 ml). The mixture was stirred for 10 min at 0 °C, and then quenched by 3N aqueous NaOH (3 ml), the mixture was then extracted by ethyl acetate (3 x 5 ml), and the combined organic phases were washed with brine, dried with Na_2SO_4, filtered, and concentrated. Purification of the crude residue by flash silica gel chromatography afforded the corresponding alcohol.

IV. General Procedure for Asymmetric Cyclopropanation: [Co(**P2**)] (5 mol %) was placed in an oven-dried, resealable Schlenk tube. The tube was capped with a Teflon screwcap, evacuated, and backfilled with nitrogen. The screwcap was replaced with a rubber septum. 1.0 Equivalent of

alkene (0.10 mmol), 1.2 equivalents of diazo compound (0.12 mmol) in 0.5 ml toluene was added once via syringe. Schlenk tube was capped by teflon screwcap instead of rubber septum and stirred at room temperature. Following completion of the reaction, the reaction mixture was purified by flash chromatography. The fractions containing product were collected and concentrated by rotary evaporation to afford the compound. In most cases, the product was visualized on TLC using the cerium ammonium molybdate (CAM) stain.

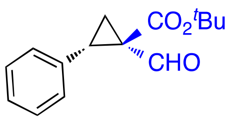

***tert*-butyl 1-formyl-2-phenylcyclopropanecarboxylate (1a):** $[\alpha]^{20}_D$ = -79.04 (c = 1.01, $CHCl_3$). ^1H NMR (250 MHz, $CDCl_3$): δ 9.85 (s, 1H), 7.25-7.08 (m, 5H), 3.29 (t, J = 8.91 Hz, 1H), 2.26 (dd, J = 8.70, 4.35 Hz, 1H), 1.90 (dd, J = 9.11, 4.34 Hz, 1H), 1.48 (s, 9H). ^{13}C NMR (62.5 MHz, $CDCl_3$): δ 195.4, 169.6, 133.1, 129.7, 128.1, 127.7, 82.5, 42.0, 41.3, 28.2, 19.9. IR (neat, cm^{-1}): 1704, 1369, 1293, 1259, 1144, 763, 697. HRMS (ESI) ($[M+H]^+$) Calcd. for $C_{15}H_{19}O_3$: 247.1329, Found: 247.1330. HPLC analysis: *ee* =96 %. OJ-H (97% hexanes: 3% isopropanol, 0.8 ml/min) t_{major} =20.43 min, t_{minor} =16.50 min.

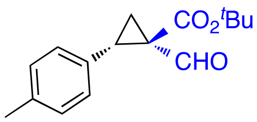

***tert*-butyl 1-formyl-2-p-tolylcyclopropanecarboxylate (1b):** $[\alpha]^{20}_D$ = -80.60 (c = 0.34, $CHCl_3$). ^1H NMR (400 MHz, $CDCl_3$): δ 9.84 (s, 1H), 7.09 (m, 4H), 3.26 (t, J = 8.90 Hz, 1H), 1.88 (dd, J = 9.09, 4.30 Hz, 1H), 2.26-2.20 (m, 4H), 1.48 (s, 9H). ^{13}C NMR (62.5 MHz, $CDCl_3$): δ 195.5, 169.7, 137.4, 130.0, 129.5, 128.8, 82.5, 42.2, 41.4, 28.4, 28.2, 21.2, 19.9. IR (neat, cm^{-1}): 1702, 1369, 1275, 1154, 1127, 1098, 763, 700. HRMS (ESI) ($[M+Na]^+$) Calcd. for $C_{16}H_{20}O_3Na$: 283.1305, Found: 283.1306.

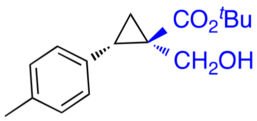

***tert*-butyl 1-(hydroxymethyl)-2-p-tolylcyclopropanecarboxylate (1ba):** ^1H NMR (250 MHz, $CDCl_3$): δ 7.05 (m, 4H), 3.40 (ddd, J = 12.33, 2.91, 0.92 Hz, 1H), 3.26 (dd, J = 12.32, 5.27 Hz, 1H), 2.82 (br, 1H), 2.25 (s, 3H),1.51 (dd, J = 8.69, 4.58 Hz, 1H), 1.44 (s, 9H), 1.28 (dd, J = 7.14, 4.75 Hz, 1H). ^{13}C NMR (62.5 MHz, $CDCl_3$): δ 173.8, 136.6, 132.9, 129.0, 129.0, 81.4, 62.3, 32.3, 30.5, 28.2, 21.1, 17.4. HRMS (ESI) ($[M+Na]^+$) Calcd. for $C_{16}H_{22}O_3Na$: 285.1461, Found: 285.1457. HPLC analysis: *ee* =97 %. Whelk (99% hexanes: 1% isopropanol, 0.8 ml/min) t_{major} = 24.24 min, t_{minor} = 28.18 min.

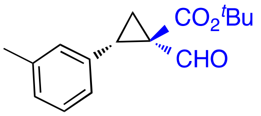

***tert*-butyl 1-formyl-2-m-tolylcyclopropanecarboxylate (1c):** $[\alpha]^{20}_{D}$ = -90.03 (*c* = 0.12, $CHCl_3$). ^1H NMR (250 MHz, $CDCl_3$): δ 9.84 (s, 1H), 7.15-6.86 (m, 4H), 1.88 (dd, *J* = 9.09, 4.36 Hz, 1H), 2.25 (dd, *J* = 14.24, 5.67 Hz, 1H), 2.25 (s, 3H) 3.26 (t, *J* = 8.93 Hz, 1H), 1.48 (s, 9H). ^{13}C NMR (62.5 MHz, $CDCl_3$): δ 195.5, 169.7, 137.7, 133.0, 130.4, 128.4, 128.0, 126.6, 82.5, 42.0, 41.3, 28.2, 21.4, 19.8. IR (neat, cm^{-1}): 1702, 1369, 1275, 1127, 1098, 763. HRMS (ESI) ($[M+H]^+$) Calcd. for $C_{16}H_{21}O_3$: 261.1485, Found: 261.1480.

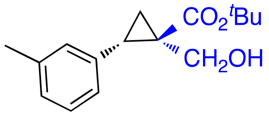

***tert*-butyl 1-(hydroxymethyl)-2-m-tolylcyclopropanecarboxylate (1ca):** ^1H NMR (250 MHz, $CDCl_3$): δ 7.17-6.91 (m, 4H), 3.46 (d, *J* = 12.32 Hz, 1H), 3.29-3.13 (m, 1H), 2.83 (t, *J* = 8.33 Hz, 1H), 2.45-2.33 (m, 1H), 2.26 (s, 3H), 1.56-1.50 (m, 1H), 1.31 (dd, *J* = 7.18, 4.78 Hz, 1H), 1.44 (s, 9H). ^{13}C NMR (62.5 MHz, $CDCl_3$): δ 173.7, 137.9, 135.9, 129.9, 128.2, 127.8, 126.1, 81.5, 62.3, 32.3, 30.7, 28.1, 21.4, 17.4. HRMS (ESI) ($[M+Na]^+$) Calcd. for $C_{16}H_{23}O_3$: 285.1461, Found: 285.1461. HPLC analysis: *ee* = 99%. AD-H (99% hexanes: 1% isopropanol, 0.8 ml/min) t_{major} = 33.50 min, t_{minor} = 21.76 min.

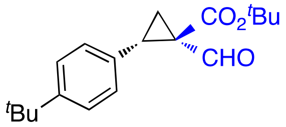

***tert*-butyl 2-(4-*tert*-butylphenyl)-1-formylcyclopropanecarboxylate (1d):** $[\alpha]^{20}_{D}$ = -117.53 (*c* = 0.98, $CHCl_3$). ^1H NMR (250 MHz, $CDCl_3$): δ 9.84 (s, 1H), 7.26-7.19 (m, 2H), 7.07 (d, *J* = 8.24 Hz, 2H), 3.25 (t, *J* = 8.97 Hz, 1H), 2.25 (dd, *J* = 8.74, 4.36 Hz, 1H), 1.89 (dd, *J* = 9.10, 4.32 Hz, 1H), 1.48 (s, 9H), 1.23 (s, 9H). ^{13}C NMR (62.5 MHz, $CDCl_3$): δ 195.5, 169.7, 150.6, 130.0, 129.3, 125.067, 82.5, 42.3, 41.3, 34.5, 31.3, 28.2, 19.8. IR (neat, cm^{-1}): 1721, 1369, 1294, 1259, 1146, 1098, 841, 753. HRMS (ESI) ($[M+Na]^+$) Calcd. for $C_{19}H_{26}O_3Na$: 325.1774, Found: 325.1773. HPLC analysis: *ee* = 97%. Whelk (99% hexanes: 1% isopropanol, 0.8 ml/min) t_{major} = 11.56 min, t_{minor} = 33.36 min.

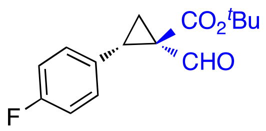

***tert*-butyl 2-(4-fluorophenyl)-1-formylcyclopropanecarboxylate (1e):** $[\alpha]^{20}_{D}$ = -193.32 (*c* = 0.67, $CHCl_3$). ^1H NMR (250 MHz, $CDCl_3$): δ 9.87 (s, 1H), 7.08-7.14 (m, 2H), 6.95-6.84 (m, 2H), 3.26 (t, *J* = 8.91 Hz, 1H), 2.21 (dd, *J* = 8.68, 4.38 Hz, 1H), 1.91 (dd, *J* = 9.13, 4.34 Hz, 1H), 1.48 (s, 9H). ^{13}C NMR (62.5 MHz, $CDCl_3$): δ 195.5, 169.5, 164.1, 160.2, 131.3, 131.2, 128.8, 128.8, 115.2, 114.9, 82.7, 42.0, 40.6, 28.2, 20.3. IR (neat, cm^{-1}): 1702, 1514, 1369, 1295, 1146, 1109, 841, 756. HRMS (ESI) ($[M+Na]^+$) Calcd. for $C_{15}H_1FO_3Na$: 287.1054, Found: 287.1055.

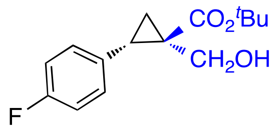

tert-butyl 2-(4-fluorophenyl)-1-(hydroxymethyl) cyclopropanecarboxylate (1ea): ^{1}H NMR (250 MHz, $CDCl_3$): δ7.16 (dd, J = 8.55, 5.76 Hz, 2H), 6.92 (t, J = 8.68 Hz, 2H), 3.31 (s, 2H), 2.81 (m, 1H), 2.57-2.40 (br, 1H), 1.53 (dd, J = 8.97, 4.79 Hz, 1H), 1.44 (s, 9H), 1.21 (m, 1H). ^{13}C NMR (62.5 MHz, $CDCl_3$): δ 173.6, 163.8, 159.9, 131.8, 130.9, 130.8, 129.2, 128.3, 115.4, 115.0, 81.7, 62.2, 32.2, 30.1, 28.1, 17.6. HRMS (ESI) ($[M+Na]^+$) Calcd. for $C_{15}H_{19}FO_3Na$: 289.1210, Found: 289.1211. HPLC analysis: *ee* = 97%. AD-H (99% hexanes: 1% isopropanol, 0.8 ml/min) t_{major} = 25.90 min, t_{minor} = 23.42 min.

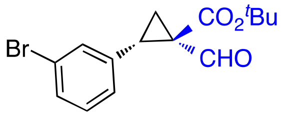

tert-butyl 2-(3-bromophenyl)-1-formylcyclopropanecarboxylate (1f): $[\alpha]^{20}_D$ = -67.81 (c = 1.00, $CHCl_3$). ^{1}H NMR (250 MHz, $CDCl_3$): δ 9.87 (s, 1H), 7.29 (m, 2H), 7.07 (m, 2H), 3.23 (t, J = 8.85 Hz, 1H), 2.20 (dd, J = 8.60, 4.38 Hz, 1H), 1.89 (dd, J = 9.13, 4.37 Hz, 1H), 1.49 (s, 9H). ^{13}C NMR (62.5 MHz, $CDCl_3$): δ 195.2, 169.3, 135.5, 132.8, 130.8, 129.6, 128.2, 122.2, 82.8, 41.8, 40.1, 28.2, 20.1. IR (neat, cm^{-1}): 1702, 1370, 1326, 1296, 1146, 846, 752. HRMS (ESI) ($[M+H]^+$) Calcd. for $C_{15}H_{18}BrO_3$: 325.0434, Found: 325.0442. HPLC analysis: *ee* = 99%. Whelk (98% hexanes: 2% isopropanol, 0.8 ml/min) t_{major} = 12.65 min, t_{minor} = 27.37 min.

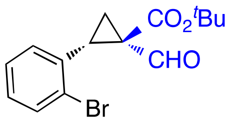

tert-butyl 2-(2-bromophenyl)-1-formylcyclopropanecarboxylate (1g): $[\alpha]^{20}_D$ = 26.94 (c = 0.88, $CHCl_3$). ^{1}H NMR (250 MHz, $CDCl_3$): δ 10.01 (s, 1H), 7.43 (d, J = 7.87 Hz, 1H), 7.21 (m, 2H), 7.11-7.02 (m, 1H), 3.12 (t, J = 8.79 Hz, 1H), 2.20 (dd, J = 8.71, 4.20 Hz, 1H), 1.97 (dd, J = 8.89, 4.24 Hz, 1H), 1.50 (s, 9H). ^{13}C NMR (62.5 MHz, $CDCl_3$): δ 194.9, 169.5, 134.0, 132.1, 131.4, 129.3, 127.1, 126.5, 82.5, 42.2, 40.7, 28.2, 20.9. IR (neat, cm^{-1}): 1702, 1368, 1315, 1297, 1146, 845, 759. HRMS (ESI) ($[M+Na]^+$) Calcd. for $C_{15}H_{17}BrO_3Na$: 347.0253, Found: 347.0252. HPLC analysis: *ee* = 98%. Whelk (99% hexanes: 1% isopropanol, 0.8 ml/min) t_{major} = 15.50 min, t_{minor} = 57.33 min.

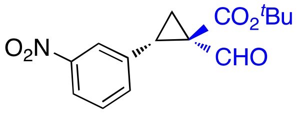

tert-butyl 1-formyl-2-(3-nitrophenyl)cyclopropanecarboxylate (1h): $[\alpha]^{20}_D$ = -73.59 (c = 1.04, $CHCl_3$). ^{1}H NMR (250 MHz, $CDCl_3$): δ 9.93 (s, 1H), 8.08-7.98 (m, 2H), 7.54-7.32 (m, 2H), 3.35 (t, J = 8.89 Hz, 1H), 2.27 (dd, J = 8.61, 4.45 Hz, 1H), 2.00 (dd, J = 9.13, 4.43 Hz, 1H), 1.51 (s, 9H). ^{13}C NMR (62.5 MHz, $CDCl_3$): δ 194.3, 167.9, 146.9, 134.6, 127.9, 123.8, 121.6, 82.1, 40.5, 38.5, 27.1, 19.8. IR

(neat, cm^{-1}): 1702, 1530, 1349, 1296, 1146, 752. HRMS (ESI) ([M+H]$^{+}$) Calcd. for $C_{15}H_{18}NO_5$: 292.1179, Found: 292.1180. HPLC analysis: *ee* = 97%. Whelk (98% hexanes: 2% isopropanol, 0.8 ml/min) t_{major} = 29.74 min, t_{minor} = 50.48 min.

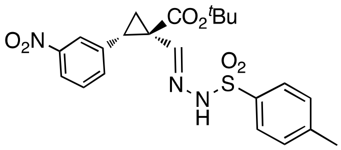

(1R,2S)-tert-butyl 2-(3-nitrophenyl)-1-((E)-(2-tosylhydrazono)methyl)cyclopropanecarboxylate (1ha): $[\alpha]^{20}_{D}$ = -29.08 (*c* = 1.01, $CHCl_3$). ^{1}H NMR (400 MHz, $CDCl_3$): δ 7.97-7.92 (m, 2H), 7.68 (d, *J* = 8.31 Hz, 2H), 7.57 (s, 1H), 7.29 (d, *J* = 8.42 Hz, 2H), 7.26 (d, *J* = 8.02 Hz, 2H), 7.16 (m, 1H), 3.09 (t, *J* = 8.57 Hz, 1H), 2.46 (s, 3H), 2.19-2.13 (m, 1H), 1.91 (dd, *J* = 9.19, 5.11 Hz, 1H), 1.46 (s, 9H). ^{13}C NMR (100 MHz, $CDCl_3$): δ 169.8, 147.8, 144.3, 144.3, 136.2, 135.1, 134.7, 129.6, 128.8, 127.9, 124.9, 122.0, 82.6, 38.1, 36.4, 28.0, 21.6, 17.6. IR (neat, cm^{-1}): 3232, 2926, 1716, 1530, 1348, 1284, 1148. HRMS (ESI) ([M+Na]$^{+}$) Calcd. for $C_{22}H_{25}N_3O_6SNa$: 482.1356, Found: 482.1381. HPLC analysis: *ee* = 96%. Whelk (90% hexanes: 10% isopropanol, 1.0 ml/min) t_{major} = 31.48 min, t_{minor} = 27.42 min.

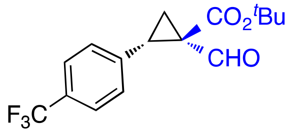

***tert*-butyl 1-formyl-2-(4-(trifluoromethyl)phenyl) cyclopropanecarboxylate (1i):** $[\alpha]^{20}_{D}$ = -96.62 (*c* = 1.02, $CHCl_3$). ^{1}H NMR (250 MHz, $CDCl_3$): δ 9.88 (s, 1H), 7.46 (d, *J* = 8.10 Hz, 2H), 7.25 (d, *J* = 8.31 Hz, 2H), 3.30 (t, *J* = 8.88 Hz, 1H), 2.25 (dd, *J* = 8.65, 4.42 Hz, 1H), 1.94 (dd, *J* = 9.06, 4.34 Hz, 1H), 1.49 (s, 9H). ^{13}C NMR (62.5 MHz, $CDCl_3$): δ 195.2, 169.2, 137.2, 130.5, 130.0, 129.4, 128.9, 126.2, 125.8, 125.1, 125.0, 125.0, 124.9, 121.8, 82.9, 41.9, 40.1, 28.1, 27.6, 20.2. IR (neat, cm^{-1}): 1720, 1698, 1327, 1296, 1140, 1119, 1068, 850. HRMS (ESI) ([M+H]$^{+}$) Calcd. for $C_{17}H_{18}F_3O_3$: 315.1203, Found: 315.1210.

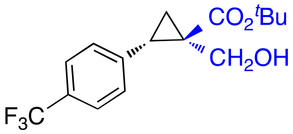

***tert*-butyl 1-(hydroxymethyl)-2-(4-(trifluoromethyl)phenyl) cyclopropanecarboxylate (1ia):** ^{1}H NMR (600 MHz, $CDCl_3$): δ 7.53 (d, *J* = 8.18 Hz, 2H), 7.37 (d, *J* = 8.06 Hz, 2H), 3.41 (d, *J* = 12.47 Hz, 1H), 3.33 (d, *J* = 12.39 Hz, 1H), 2.93 (t, *J* = 8.00 Hz, 1H), 2.52 (s, 1H), 1.63 (dd, *J* = 8.91, 4.92 Hz, 1H), 1.50 (s, 9H), 1.34 (dd, *J* = 7.13, 4.94 Hz, 1H). ^{13}C NMR (62.5 MHz, $CDCl_3$): δ 172.2, 139.3, 128.6, 128.4, 127.9, 125.2, 124.3, 124.2, 124.1, 124.1, 120.9, 80.9, 60.9, 31.5, 29.3, 27.1, 16.3. HRMS (ESI) ([M+Na]$^{+}$) Calcd. for $C_{16}H_{19}F_3O_3Na$: 339.1179, Found: 339.1172. HPLC

analysis: *ee* = 96%. AD-H (99% hexanes: 1% isopropanol, 0.8 ml/min) t_{major} = 21.08 min, t_{minor} = 46.67 min.

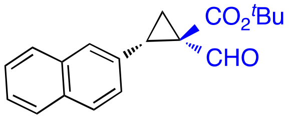

***tert*-butyl 1-formyl-2-(naphthalen-2-yl)cyclopropanecarboxylate (1j):** $[\alpha]^{20}_{D}$ = -170.82 (*c* = 1.00, $CHCl_3$). ^{1}H NMR (250 MHz, $CDCl_3$): δ 9.86 (s, 1H), 7.79-7.14 (m, 7H), 3.45 (t, *J* = 8.91 Hz, 1H), 2.40 (dd, *J* = 8.66, 4.36 Hz, 1H), 1.98 (dd, *J* = 9.04, 4.32 Hz, 1H), 1.50 (s, 9H)**.** ^{13}C NMR (62.5 MHz, $CDCl_3$): δ 195.2, 169.6, 133.0, 132.7, 130.5, 128.6, 127.9, 127.7, 127.6, 127.5, 126.2, 126.1, 82.6, 42.2, 41.5, 28.2, 20.0. IR (neat, cm^{-1}): 1702, 1369, 1315, 1142, 1101, 752, 736. HRMS (ESI) ($[M+H]^+$) Calcd. for $C_{19}H_{21}O_3$: 297.1485, Found: 297.1490.

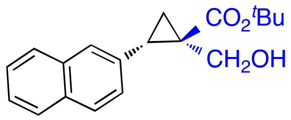

***tert*-butyl 1-(hydroxymethyl)-2-(naphthalen-2-yl) cyclopropanecarboxylate (1ja):** $[\alpha]^{20}_{D}$ = -153.60 (*c* = 0.89, $CHCl_3$). ^{1}H NMR (250 MHz, $CDCl_3$): δ 7.71 (d, *J* = 8.80 Hz, 3H), 7.59 (s, 1H), 7.47-7.31 (m, 3H), 3.48-3.36 (m, 1H), 3.35-3.23 (m, 1H), 3.01 (t, *J* = 7.94 Hz, 1H), 2.4(br, 1H), 1.62 (dd, *J* = 8.86, 4.85 Hz, 1H), 1.47 (m,10H). ^{13}C NMR (62.5 MHz, $CDCl_3$): δ 173.7, 133.7, 133.2, 132.5, 127.9, 127.8, 127.6, 127.3, 126.9, 126.2, 125.8, 81.6, 62.2, 32.7, 31.1, 28.2, 17.5. HRMS (ESI) ($[M+Na]^+$) Calcd. for $C_{19}H_{22}O_3Na$: 321.1461, Found: 321.1456. HPLC analysis: *ee* = 95%. OD-H (97% hexanes: 3% isopropanol, 0.8 ml/min) t_{major} = 29.44 min, t_{minor} = 18.00 min.

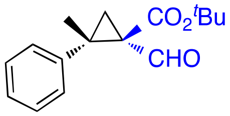

***tert*-butyl 1-formyl-2-methyl-2-phenylcyclopropanecarboxylate (1k):** $[\alpha]^{20}_{D}$ = -113.47 (*c* = 0.49, $CHCl_3$). ^{1}H NMR (250 MHz, $CDCl_3$): δ 9.44 (s, 1H), 7.33-7.08 (m, 5H), 2.18 (d, *J* = 4.96 Hz, 1H), 1.79 (d, *J* = 4.96 Hz, 1H), 1.51 (s, 12H)**.** ^{13}C NMR (62.5 MHz, $CDCl_3$): δ 196.1, 167.9, 140.0, 128.8, 128.5, 127.3, 82.4, 48.0, 44.2, 28.3, 23.4, 23.3. IR (neat, cm^{-1}): 1702, 1369, 1275, 1154, 1127, 1052, 757, 700. HRMS (ESI) ($[M+H]^+$) Calcd. for $C_{16}H_{21}O_3$: 261.1485, Found: 261.1485. HPLC analysis: *ee* = 94%. Whelk (98% hexanes: 2% isopropanol, 0.8 ml/min) t_{major} = 11.58 min, t_{minor} = 18.90 min.

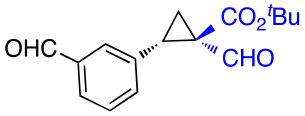

***tert*-butyl 1-formyl-2-(3-formylphenyl)cyclopropanecarboxylate (1l):** $[\alpha]^{20}_{D}$ = -68.55 (*c* = 1.00, $CHCl_3$). ^{1}H NMR (400 MHz, $CDCl_3$): δ 9.97 (s, 1H), 9.95 (s, 1H), 7.74 (t, *J* = 8.29 Hz, 2H), 7.50-7.41 (m, 2H), 3.40 (t, *J* = 8.87 Hz, 1H), 2.33 (dd, *J* = 8.65, 4.41 Hz, 1H), 2.02 (dd, *J* = 9.00, 4.54 Hz, 1H), 1.55 (s, 9H). ^{13}C NMR (100 MHz, $CDCl_3$): δ 195.3, 192.0, 169.1, 136.1, 135.5, 134.4, 130.6, 129.2, 128.7,

82.8, 41.7, 40.1, 28.1, 20.4. IR (neat, cm^{-1}): 1700, 1369, 1295, 1144, 751. HPLC analysis: *ee* = 95%. OJ-H (95% hexanes: 5% isopropanol, 1.0 ml/min) t_{major} = 11.54 min, t_{minor} = 10.17 min.

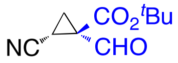

(1R,2R)-tert-butyl 2-cyano-1-formylcyclopropanecarboxylate (1m): *Major* isomer: ^1H NMR (500 MHz, $CDCl_3$): δ 10.25 (s, 1H), 2.37 (dd, *J* = 9.52, 7.54 Hz, 1H), 2.11-2.04 (m, 1H), 1.81 (dd, *J* = 9.55, 4.28 Hz, 1H), 1.58 (s, 9H). *Minor* isomer: ^1H NMR (500 MHz, $CDCl_3$): δ δ 10.26 (s, 1H), 2.49 (dd, *J* = 9.46, 7.65 Hz, 1H), 2.11-2.04 (m, 1H), 1.89 (dd, *J* = 9.48, 4.26 Hz, 1H), 1.54 (s, 9H). ^{13}C NMR (125 MHz, $CDCl_3$): δ 195.3, 193.2, 166.3, 165.3, 115.9, 115.2, 84.9, 84.6, 37.4, 37.3, 27.9, 22.9, 21.7, 16.9, 16.3. IR (neat, cm^{-1}): 2250, 1712, 1325, 1144, 840. HRMS (ESI) ($[M+H]^+$) Calcd. for $C_{10}H_{14}NO_3$: 178.0974, Found: 178.0974. HPLC analysis: *ee (major)* = 77 %. AD-H (99% hexanes: 1% isopropanol, 1.0 ml/min) t_{major} = 25.28 min, t_{minor} = 11.49 min.

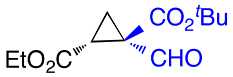

***tert*-butyl 2-ethyl 1-formylcyclopropane-1,2-dicarboxylate (1n):** $[\alpha]^{20}_D$ = -16.37 (*c* = 0.98, $CHCl_3$). *Major* isomer: ^1H NMR (250 MHz, $CDCl_3$): δ 9.93 (s, 1H), 4.08 (m, 2H), 2.63 (t, *J* = 8.22 Hz, 1H), 2.07-1.91 (m, 1H), 1.72-1.54 (m, 1H), 1.43 (s, 9H), 1.26-1.11 (m, 3H). *Minor* isomer: ^1H NMR (250 MHz, $CDCl_3$): δ 9.94 (s, 1H), 4.08 (m, 7H), 2.50 (t, *J* = 8.44 Hz, 1H), 2.07-1.91 (m, 1H), 1.72-1.54 (m, 1H), 1.43 (s, 9H), 1.26-1.11 (m, 3H). ^{13}C NMR (62.5 MHz, $CDCl_3$): δ 195.3, 193.3, 166.7, 166.6, 166.5, 165.3, 82.3, 82.0, 60.6, 60.5, 39.8, 38.1, 31.9, 31.5, 27.0, 26.9, 20.1, 19.2, 13.1, 13.0. IR (neat, cm^{-1}): 1724, 1370, 1290, 1143, 752. HRMS (ESI) ($[M+H]^+$) Calcd. for $C_{12}H_{19}O_5$: 243.1227, Found: 243.1229. GC analysis: *ee (major)* = 96 %. CP-Chirasil-Dex CB (Temp program: initial temp = 50° C, hold time = 5 min, Rate: 5.00° C/min, max temp =200° C, hold time= 5 min): t_{minor} = 25.80 min, t_{major} = 25.74 min.

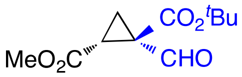

1-*tert*-butyl 2-methyl 1-formylcyclopropane-1,2-dicarboxylate(1o): $[\alpha]^{20}_D$ = -15.19 (*c* = 1.01, $CHCl_3$). *Major* isomer: ^1H NMR (250 MHz, $CDCl_3$): δ 9.94 (s, 1H), 3.64 (s, 3H), 2.64 (dd, *J* = 8.94, 7.82 Hz, 1H), 2.06-1.93 (m, 1H), 1.72-1.60 (m, 1H), 1.43 (s, 9H). *Minor* isomer: ^1H NMR (250 MHz, $CDCl_3$): δ1H NMR (250 MHz, Solvent) d ppm 9.96 (s, 1H), 3.65 (s, 3H), 2.51 (m, 1H), 2.06-1.93 (m, 1H), 1.72-1.60 (m, 1H), 1.44 (s, 9H), ^{13}C NMR (62.5 MHz, $CDCl_3$): δ 196.3, 194.4, 168.1, 168.0, 167.7, 166.4, 83.4, 83.1, 52.5, 52.4, 40.7, 39.1, 32.7, 32.3, 28.0, 27.9, 21.2, 20.4. IR (neat, cm^{-1}): 1712, 1370, 1290, 1249, 1142, 843, 753. HRMS (ESI) ($[M+H]^+$) Calcd. for $C_{11}H_{17}O_5$: 229.1071, Found: 229.1071. GC analysis: *ee*

(*major*) = 97 %. CP-Chirasil-Dex CB (Temp program: initial temp = 50° C, hold time = 5 min, Rate: 5.00° C/min, max temp =200° C, hold time= 5 min) t_{minor} = 24.68 min, t_{major} = 24.63 min.

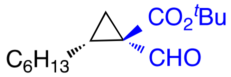

***tert*-butyl 1-formyl-2-hexylcyclopropanecarboxylate (1p):** $[\alpha]^{20}_{D}$ = -7.13 (*c* = 1.01, $CHCl_3$). ^1H NMR (400 MHz, $CDCl_3$): δ 10.28 (s, 1H), 2.07-1.96 (m, 1H), 1.63 (ddd, *J* = 18.88, 8.68, 3.59 Hz, 2H), 1.46 (m, 11H) , 1.28-1.19 (m, 8H),0.85 (t, *J* = 6.90 Hz, 3H). ^{13}C NMR (100 MHz, $CDCl_3$): δ 197.9, 170.4, 81.8, 39.3, 31.6, 29.6, 28.6, 28.1, 28.0, 25.8, 23.5, 22.5, 13.9. IR (neat, cm^{-1}): 1702, 1369, 1296, 1144, 751. HRMS (ESI) ($[M+Na]^+$) Calcd. for $C_{15}H_{27}O_3$: 277.1774, Found: 277.1781. GC analysis: *ee* =80 %. CP-Chirasil-Dex CB (Temp program: initial temp = 50° C, hold time = 5 min; Rate: 3.00° C/min, max temp =200° C, hold time= 5 min) t_{minor} = 37.68 min, t_{major} = 37.53 min.

V. Further Transformations of Chiral 1,1-Cyclopropaneformylesters

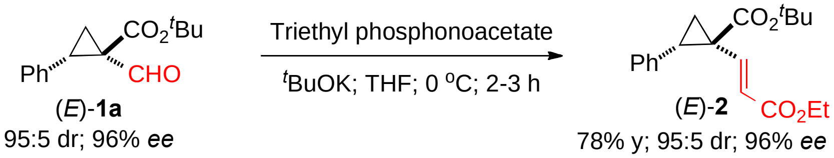

(1)

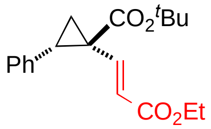

(E)-*tert*-butyl 1-(3-ethoxy-3-oxoprop-1-enyl)-2-phenyl cyclopropanecarboxylate (2): To an RBF containing potassium tert-butoxide (40.0 mg, 0.36 mmol) in THF (5 ml) at 0 °C, triethyl phosphonoacetate (81 mg, 0.36 mmol) in THF (2 ml) was added slowly via syringe. The mixture was allowed to stir for 30 min and then cyclopropane aldehyde **1a** (74 mg, 0.3 mmol) in THF (3 ml) was added dropwise via syringe. The reaction was allowed to stir at 0 °C for 2 h at which time TLC indicated all starting material was consumed. The reaction was then quenched by the addition of water. This mixture is extracted with ether (3 x 5 ml) and the combined organics are dried over Na_2SO_4. After concentration the crude mixture was purified by silica gel column chromatography to give pure product **2** in 78% yield. $[\alpha]^{20}_{D}$ = -36.11 (*c* = 0.97, $CHCl_3$). ^1H NMR (400 MHz, $CDCl_3$): δ 7.29-7.06 (m, 6H), 6.87 (d, *J* = 15.95 Hz, 1H), 5.60 (d, *J* = 15.95 Hz, 1H), 4.04 (q, *J* = 7.11 Hz, 2H), 3.12 (t, *J* = 8.42 Hz, 1H), 2.01 (dd, *J* = 9.15, 5.04 Hz, 1H), 1.68 (dd, *J* = 7.65, 5.04 Hz, 1H), 1.50 (s, 9H), 1.17 (t, *J* = 7.11 Hz, 3H), ^{13}C NMR (100 MHz, $CDCl_3$): δ 170.9, 166.1, 142.9, 134.6, 129.4, 128.2, 127.1, 121.3, 81.6, 60.0, 37.0, 33.1, 28.0, 19.8, 14.1. IR (neat, cm^{-1}): 1709,1369, 1270, 1185, 1145, 750. HRMS (ESI) ($[M+Na]^+$) Calcd. for $C_{19}H_{24}O_4Na$: 339.1567, Found:

339.1575. HPLC analysis: *ee* = 96%. Whelk (99% hexanes: 9% isopropanol, 1.0 ml/min) t_{major} = 16.79 min, t_{minor} = 22.21 min.

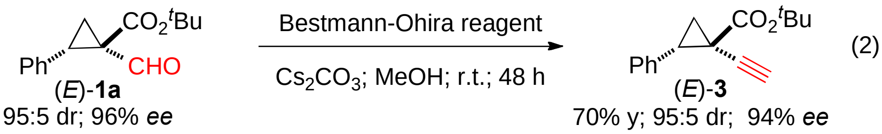

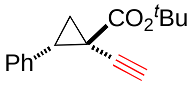

***tert*-butyl 1-ethynyl-2-phenylcyclopropanecarboxylate (3):** In a flame-dried flask under an atmosphere of nitrogen, the cyclopropane aldehyde **1a** (74 mg, 0.3 mmol) was dissolved in methanol (10 ml), and Cs_2CO_3 (815 mg, 2.5 mmol) was added. Then, the Bestmann-Ohira reagent[3] (192 mg, 1.0 mmol) was added, and the reaction mixture was stirred at room temperature for 48 h. After complete conversion, water was added, and the aqueous layer was extracted with ether (3 x 5 ml) and the combined organics are dried over Na_2SO_4, and filtered. The solvent was removed under reduced pressure, and the product was purified by flash column chromatography to give pure product **3** in 70% yield. $[\alpha]^{20}_{D}$ = -46.23 (c = 0.98, $CHCl_3$). ^1H NMR (400 MHz, $CDCl_3$): δ 7.30 (m, 5H), 2.93 (t, J = 8.66 Hz, 1H), 1.99 (s, 1H), 1.96 (dd, J = 9.20, 4.62 Hz, 1H), 1.69 (dd, J = 7.92, 4.62 Hz, 1H), 1.50 (s, 9H). ^{13}C NMR (100 MHz, $CDCl_3$): δ 169.9, 135.4, 128.7, 127.9, 127.1, 82.0, 80.1, 71.3, 34.7, 27.9, 25.5, 23.6. IR (neat, cm^{-1}): 1714, 1369, 1302, 1267, 1149, 755, 695. HRMS (ESI) ($[M+Na]^+$) Calcd. for $C_{16}H_{18}O_2Na$: 265.1199, Found: 265.1231. HPLC analysis: *ee* = 94%. OD-H (100% hexanes: 0% isopropanol, 1.0 ml/min) t_{major} = 19.61 min, t_{minor} = 18.22 min.

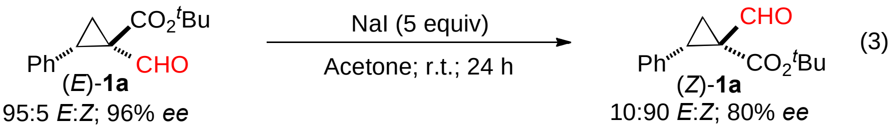

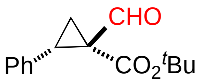

***tert*-butyl 1-formyl-2-phenylcyclopropanecarboxylate ((Z)-1a):** To a solution of *(E)*-cyclopropane **1a** (0.1 mmol) in acetone (1 ml) was added NaI (0.5 mmol). The mixture was stirred at room temperature for 24 h. The resulting mixture was concentrated and the residue was dissolved in ethyl acetate, and then washed with water. The organic layer was concentrated, according to crude NMR, *(Z)*-**1a** was formed in 90% yield. $[\alpha]^{20}_{D}$ = -131.42 (c = 0.63, $CHCl_3$). ^1H NMR (400 MHz, $CDCl_3$): δ 10.32 (s, 1H), 7.27 (m, 5H), 3.13 (t, J = 8.86 Hz, 1H), 2.29 (dd, J = 8.54, 4.27 Hz, 1H), 1.98 (dd, J = 9.18, 4.27 Hz, 1H), 1.10 (s, 9H). ^{13}C NMR (100 MHz, $CDCl_3$): δ 198.6, 167.1, 134.2, 129.5, 128.0, 127.6, 81.9, 43.0, 40.8, 27.5,

21.3. IR (neat, cm^{-1}): 1707, 1326, 1144, 696. HRMS (ESI) ($[M+Na]^+$) Calcd. for $C_{15}H_{18}O_3$: 269.1148, Found: 269.1150. GC analysis: *ee* =79 %. CP-Chirasil-Dex CB (Temp program: initial temp = 50° C, hold time = 5 min; Rate: 5.00° C/min, max temp =200° C, hold time= 5 min) t_{minor} = 28.30 min, t_{major} = 28.60 min.

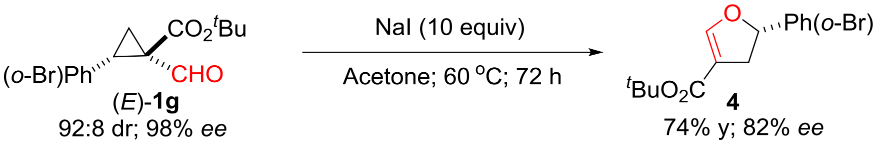

(4)

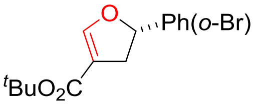

***tert*-butyl 5-(2-bromophenyl)-4,5-dihydrofuran-3-carboxylate (4):** To a solution of *(E)*-cyclopropane aldehyde **1g** (0.1 mmol) in acetone (1 ml) was added NaI (1.0 mmol). The mixture was stirred at 60 °C for 72 h. The resulting mixture was concentrated and the residue was dissolved in ethyl acetate, and then washed with water. The organic layer was concentrated, followed by flash silica gel chromatography to give diydrofuran **4** in 74% yield. $[\alpha]^{20}_D$ = -57.76 (c = 0.49, $CHCl_3$). ^{1}H NMR (400 MHz, $CDCl_3$): δ 7.19-7.14 (m, 1H), 7.56 (dd, J = 7.97, 1.13 Hz, 1H), 7.38 (dd, J = 7.80, 1.74 Hz, 1H), 7.33 (dd, J = 7.36, 0.97 Hz, 1H), 7.31 (t, J = 1.88 Hz, 1H), 5.97 (dd, J = 11.05, 8.04 Hz, 1H), 3.43 (ddd, J = 15.10, 11.06, 1.96 Hz, 1H), 2.61 (ddd, J = 15.10, 8.04, 1.84 Hz, 1H), 1.46 (s, 9H). ^{13}C NMR (100 MHz, $CDCl_3$): δ 164.3, 155.2, 140.9, 132.7, 129.2, 127.6, 126.2, 120.7, 110.2, 84.7, 80.0, 35.9, 28.2. IR (neat, cm^{-1}): 1698, 1630, 1123, 1099, 752. HRMS (ESI) ($[M+Na]^+$) Calcd. for $C_{15}H_{17}BrO_3Na$: 347.0253, Found: 347.0263. HPLC analysis: *ee* = 82%. Whelk (99% hexanes: 1% isopropanol, 0.8 ml/min) t_{major} = 11.82 min, t_{minor} = 13.94 min.

VI. X-ray Crystallography

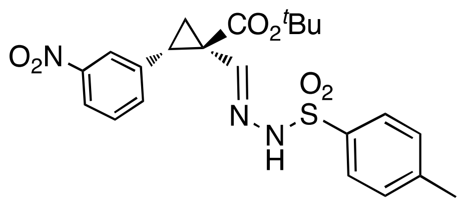

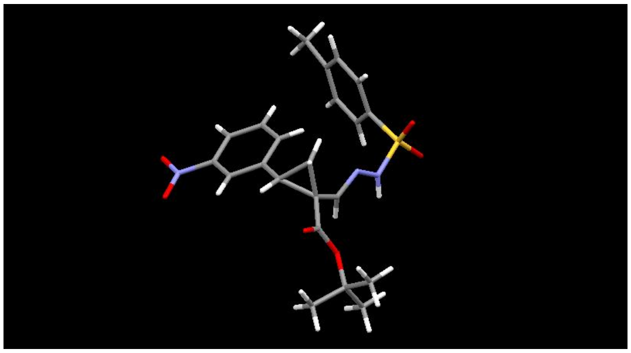

Table S1. Crystal data and structure refinement for 1ha	
Identification code	**1ha**
Empirical formula	$C_{22}H_{25}N_3O_6S$
Formula weight	459.51
Temperature/K	100.0
Crystal system	monoclinic
Space group	P21
a/Å	13.2053(5)
b/Å	5.3447(2)
c/Å	15.9599(7)
α/°	90
β/°	93.6740(10)
γ/°	90
Volume/Å^3	1124.11(8)
Z	2
ρ_{calc}mg/mm^3	1.358
m/mm^{-1}	1.656

F(000)	484.0
Crystal size/mm^3	0.16 × 0.14 × 0.11
Radiation	CuKα (λ = 1.54178)
2Θ range for data collection	8.43 to 132.63°
Index ranges	$-15 \le h \le 15$, $-6 \le k \le 6$, $-18 \le l \le 18$
Reflections collected	13901
Independent reflections	3462 [R_{int} = 0.0437, R_{sigma} = 0.0392]
Data/restraints/parameters	3462/1/301
Goodness-of-fit on F^2	1.070
Final R indexes [I>=2σ (I)]	R_1 = 0.0258, wR_2 = 0.0595
Final R indexes [all data]	R_1 = 0.0267, wR_2 = 0.0604
Largest diff. peak/hole / e Å^{-3}	0.26/-0.17
Flack parameter	0.092(8)

The X-ray diffraction data for compound **1ha** were measured on a Bruker D8 Venture PHOTON 100 CMOS system equipped with a Cu K_α INCOATEC Imus micro-focus source (λ = 1.54178 Å). Indexing was performed using *APEX2* [1] (Difference Vectors method). Data integration and reduction were performed using SaintPlus 6.01 [2]. Absorption correction was performed by multi-scan method implemented in SADABS [3]. Space groups were determined using XPREP implemented in APEX2 [1]. The structure was solved using SHELXS-97 (direct methods) and refined using SHELXL-97 (full-matrix least-squares on F^2) contained in APEX2 [1] and WinGX v1.70.01 [4,5,6,7] programs packages. All non-hydrogen atoms were refined anisotropically. H9 hydrogen atom of -NH group and H15 of –CH group have been found from difference Fourier

map and were freely refined. The remaining hydrogen atoms were placed in geometrically calculated positions and included in the refinement process using riding model with isotropic thermal parameters: Uiso(H) = 1.2Ueq(-CH,-CH_2), Uiso(H) = 1.5Ueq(-CH_3). Crystal data and refinement conditions are shown in Table S1.

[1] Bruker (2008). *APEX2* (Version 2008.1-0). Bruker AXS Inc., Madison, Wisconsin, USA.

[2] Bruker (2001b). SAINT-V6.28A. Data Reduction Software.

[3] Sheldrick, G. M. (1996). *SADABS. Program for Empirical Absorption Correction.* University of Gottingen, Germany.

[4] Farrugia L.J. Appl. Cryst. (1999). 32, 837±838

[5] Sheldrick, G.M. (1997) SHELXL-97. Program for the Refinement of Crystal

[6] Sheldrick, G.M. (1990) Acta Cryst. A46, 467-473

[7] Sheldrick, G. M. (2008). Acta Cryst. A64, 112-122.

VII. References

(1) Stojanov.Fm; Arnold, Z. *Collect. Czech. Chem. C.* **1967**, *32*, 2155.

(2) Gao, L. Z.; Hwang, G. S.; Ryu, D. H. *J. Am. Chem. Soc.* **2011**, *133*, 20708.

(3) Ohira, S. *Syn. Comm.* **1989**, *19*, 561.

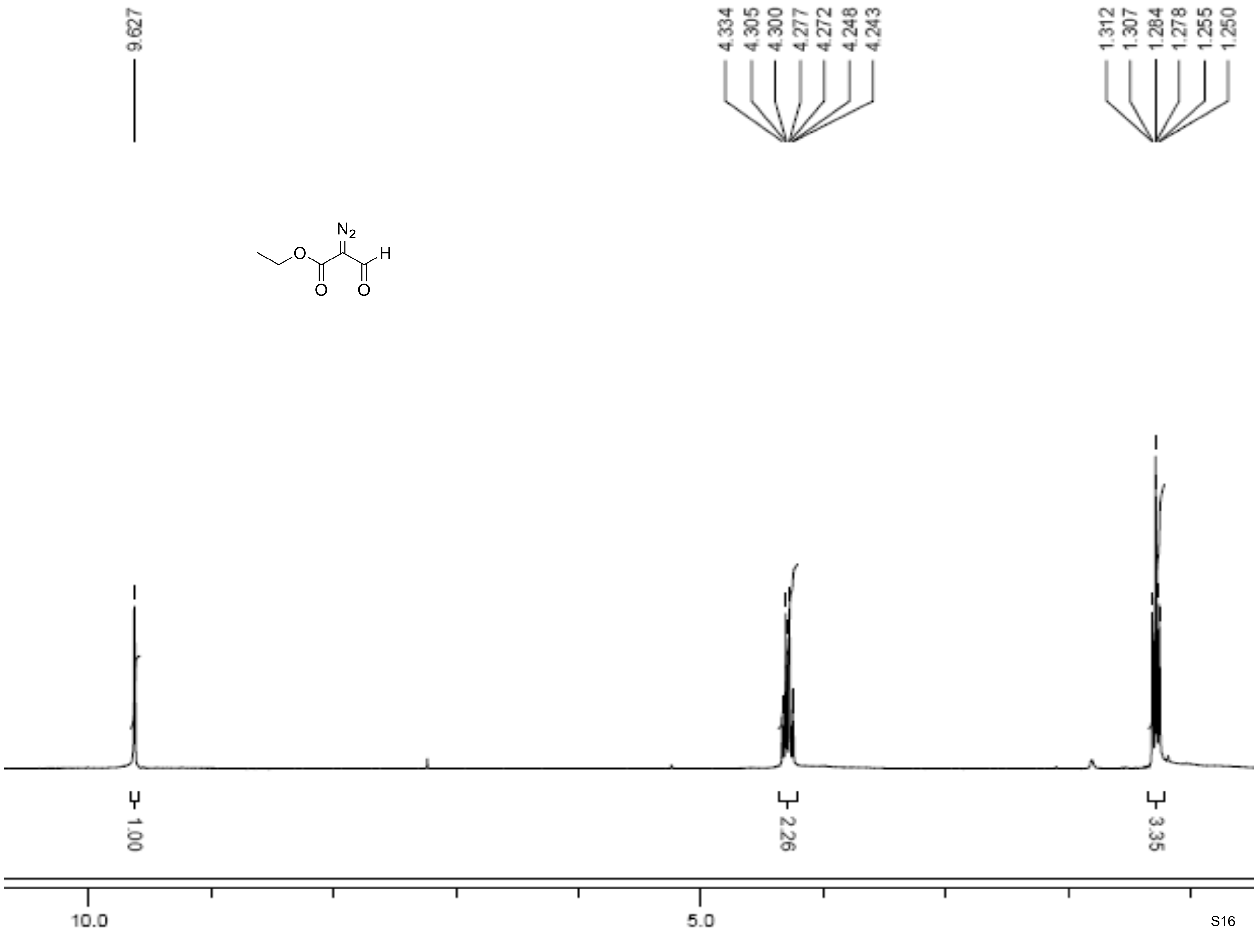
9.627
4.334
4.305
4.300
4.277
4.272
4.248
4.243
1.312
1.307
1.284
1.278
1.255
1.250
N2
O
H
O
O
1.00
2.26
3.35
10.0
5.0

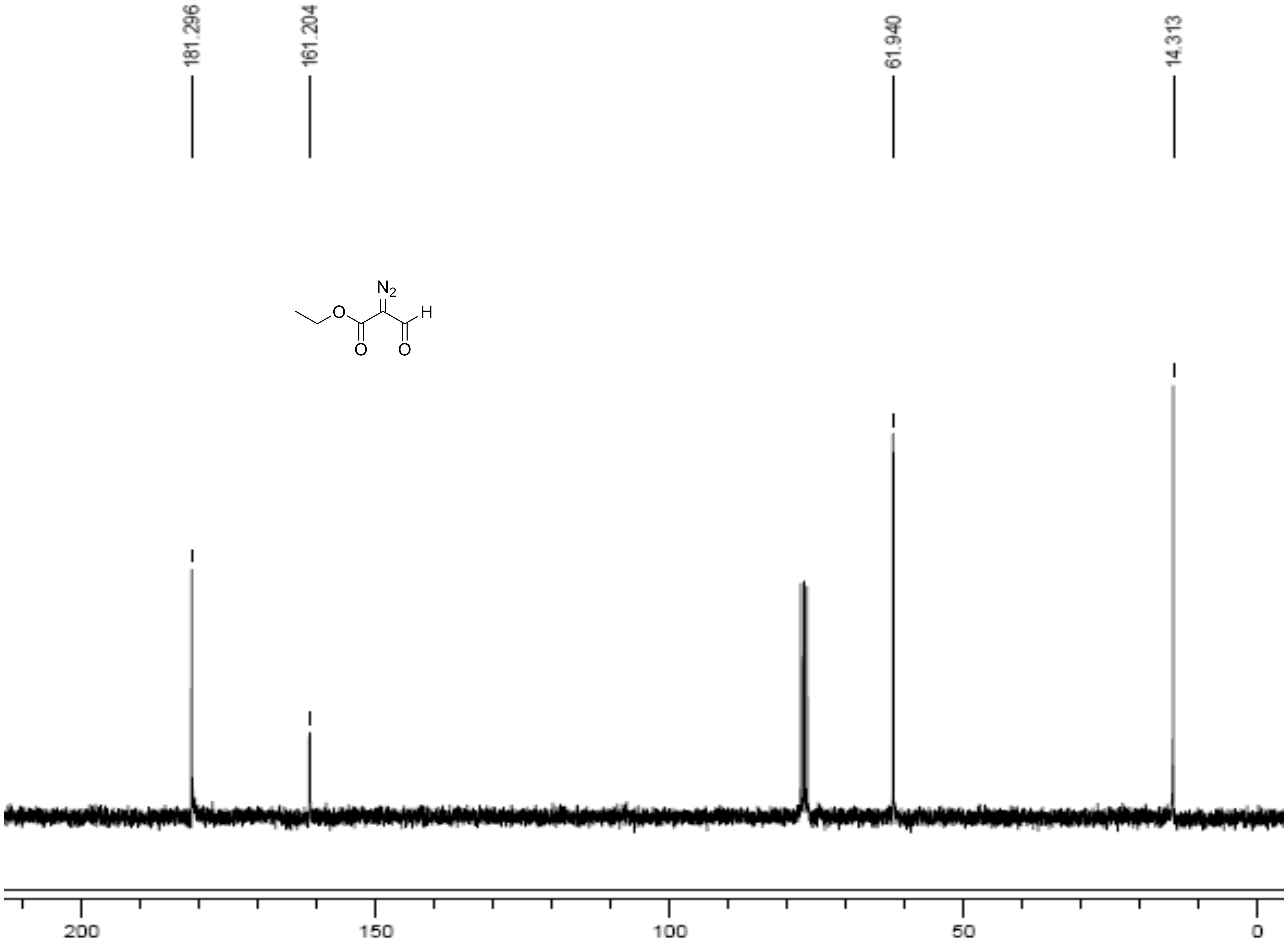
181.296
161.204
61.940
14.313
N2
O
H
O
O
200
150
100
50
0

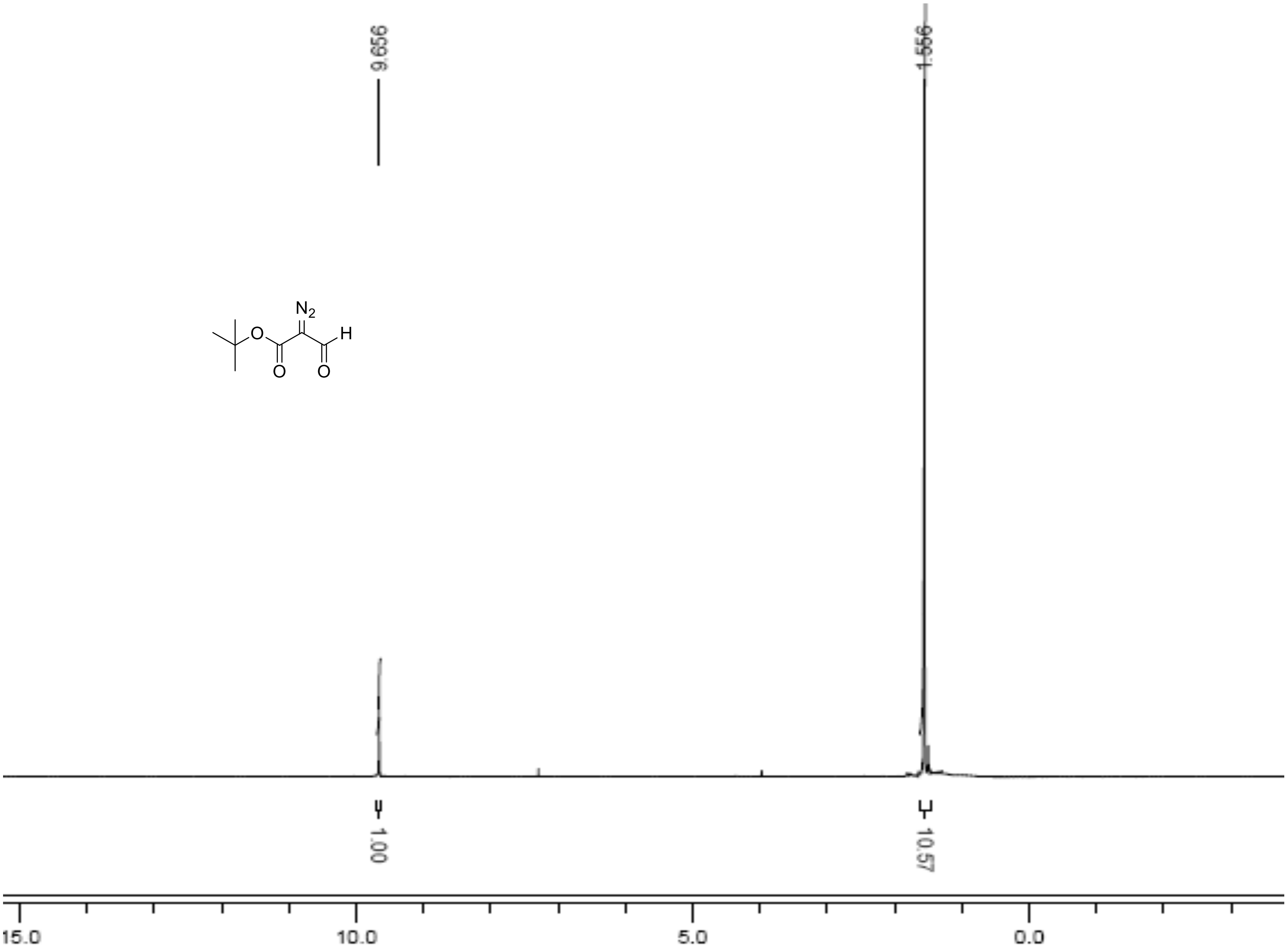
9.656
1.556
N_2
O
H
O
O
1.00
10.57
15.0
10.0
5.0
0.0

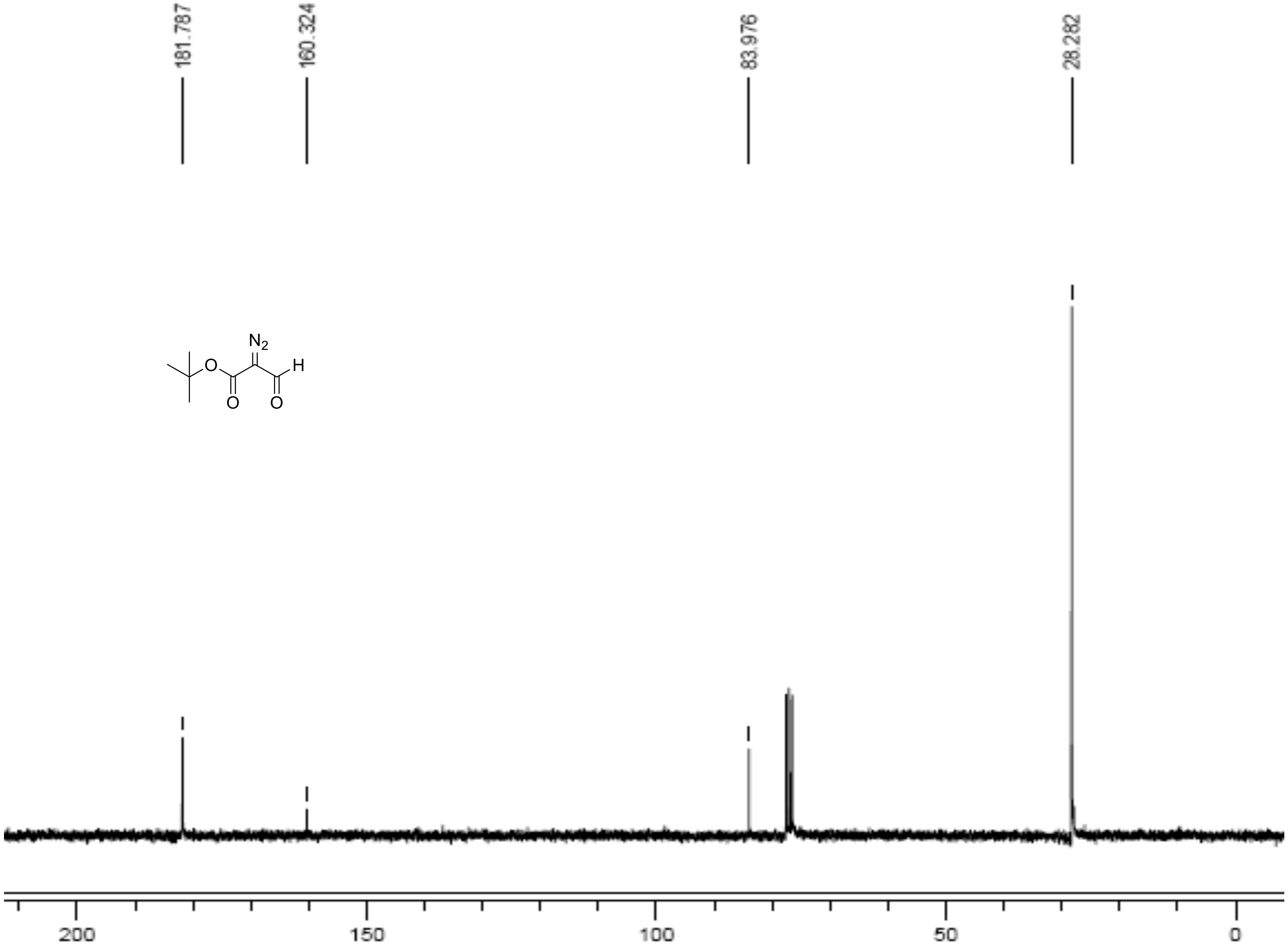
181.787
160.324
83.976
28.282
N2
O
H
O
O
200
150
100
50
0

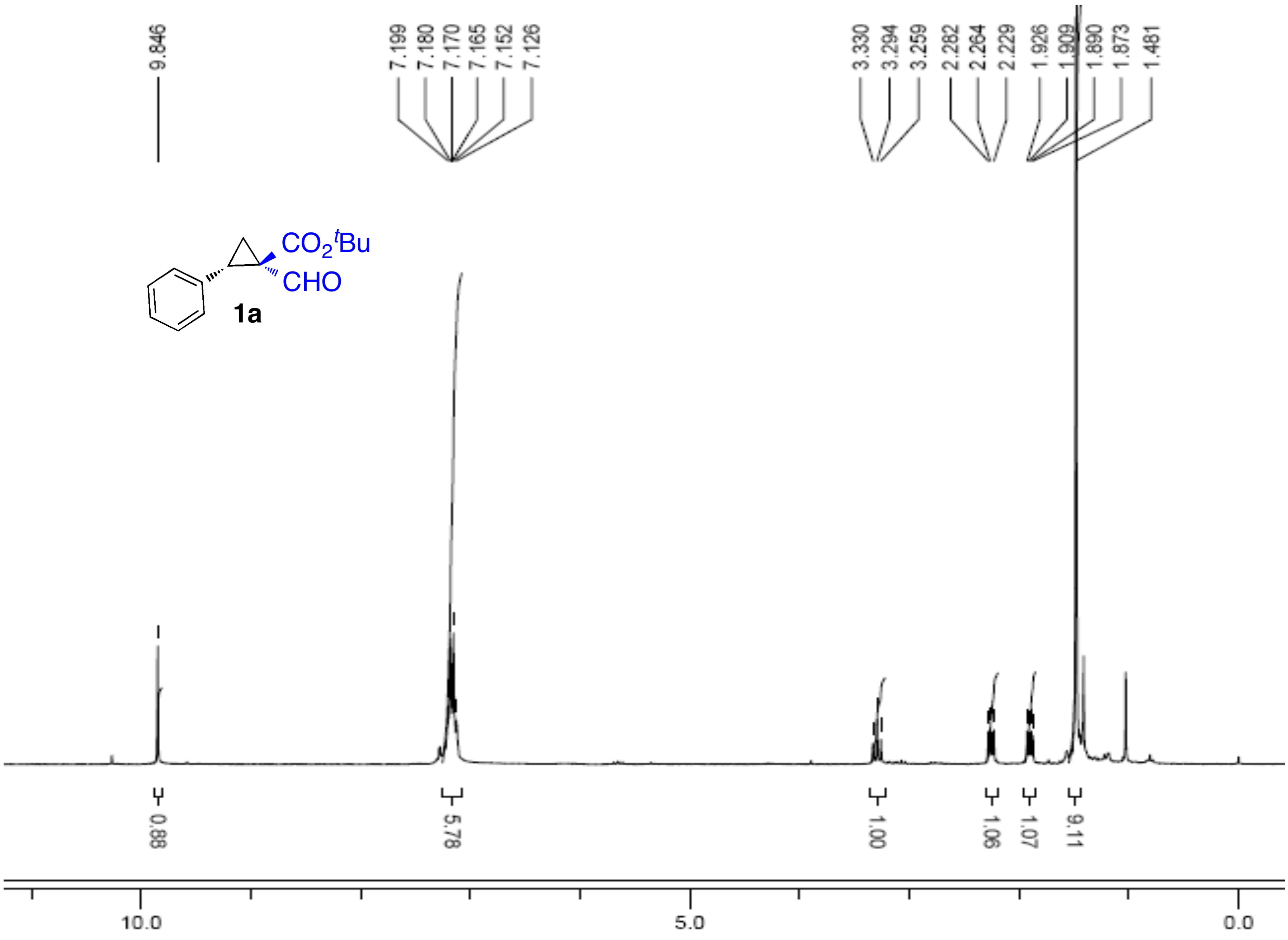
9.846
7.199
7.180
7.170
7.165
7.152
7.126
3.330
3.294
3.259
2.282
2.264
2.229
1.926
1.909
1.890
1.873
1.481
CO2tBu
CHO
1a
0.88
5.78
1.00
1.06
1.07
9.11
10.0
5.0
0.0

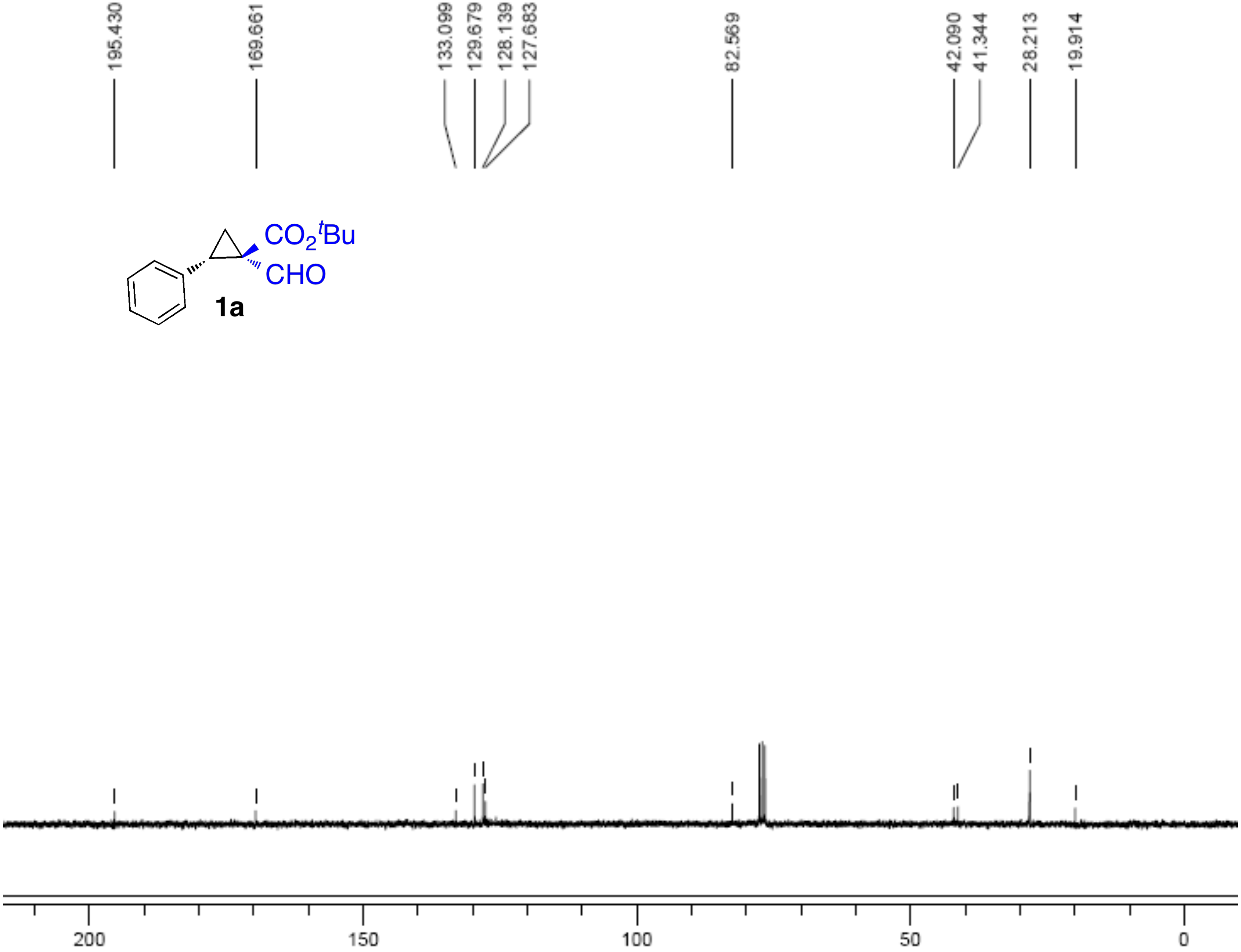
195.430
169.661
133.099
129.679
128.139
127.683
82.569
42.090
41.344
28.213
19.914
CO2tBu
CHO
1a
200
150
100
50
0

xx-vi-7 OJH 3%@0.8 ml
C:\EZStart\Projects\Default\Method\yang-ADH 5% 1.0ml.met
C:\EZStart\Projects\Default\Data\xx-vi-7 OJH 3%@0.8 ml

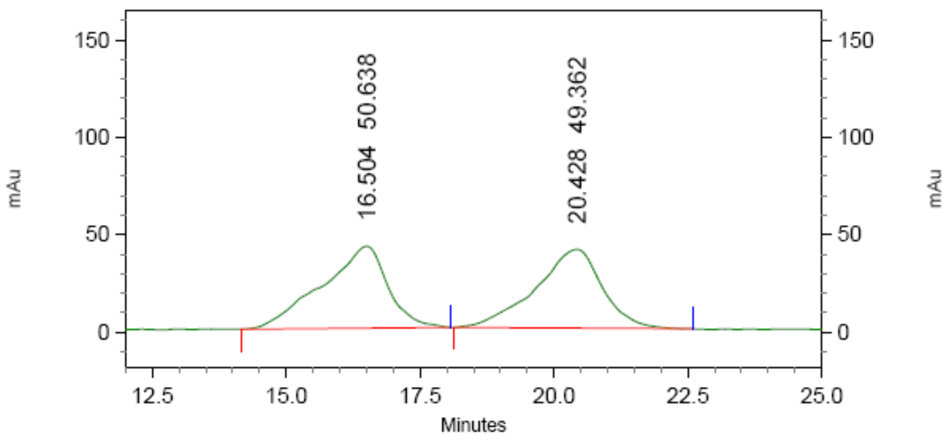

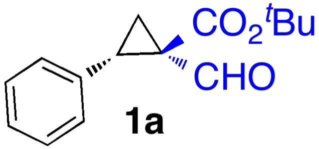

1a

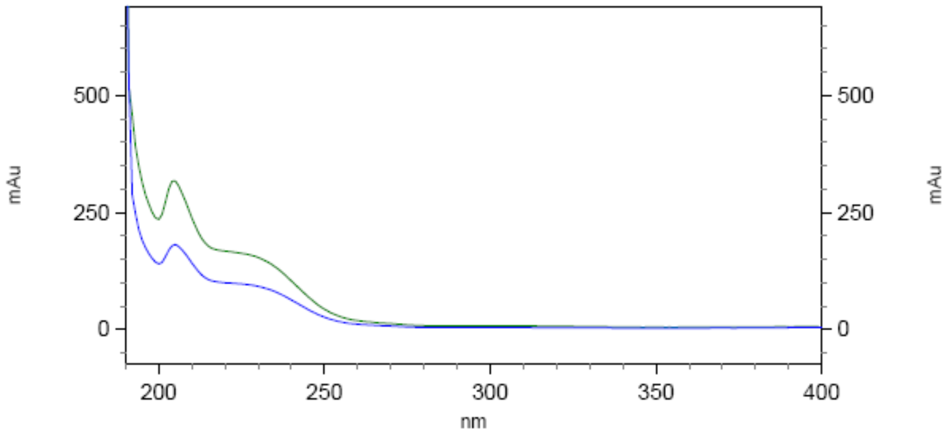

4: 231 nm, 4 nm
Results

Pk # Name	Retention Time	Area Percent
1	16.504	50.638
2	20.428	49.362
Totals		100.000

xx-vi-289 OJH 3%@0.8 ml again
C:\EZStart\Projects\Default\Method\Shifa 4-t-Butylbenzene (OD-H 10-90).met
C:\EZStart\Projects\Default\Data\xx-vi-289 OJH 3%@0.8 ml again

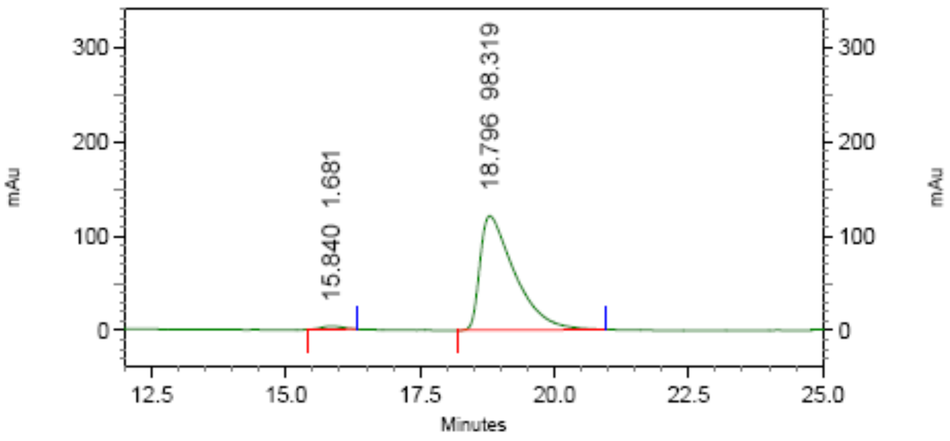

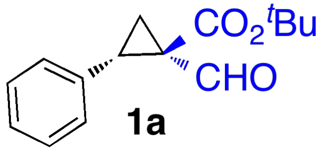

1a

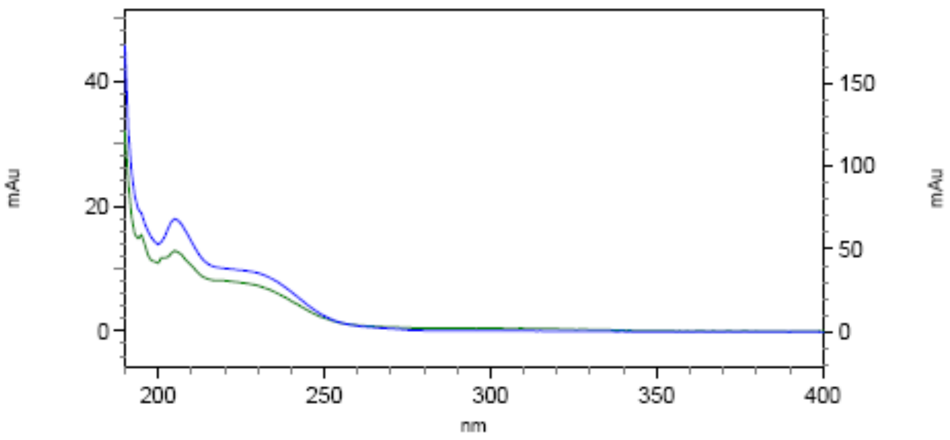

3: 226 nm, 4 nm
Results

Pk #	Name	Retention Time	Area Percent
1		15.840	1.681
2		18.796	98.319

Totals	
	100.000

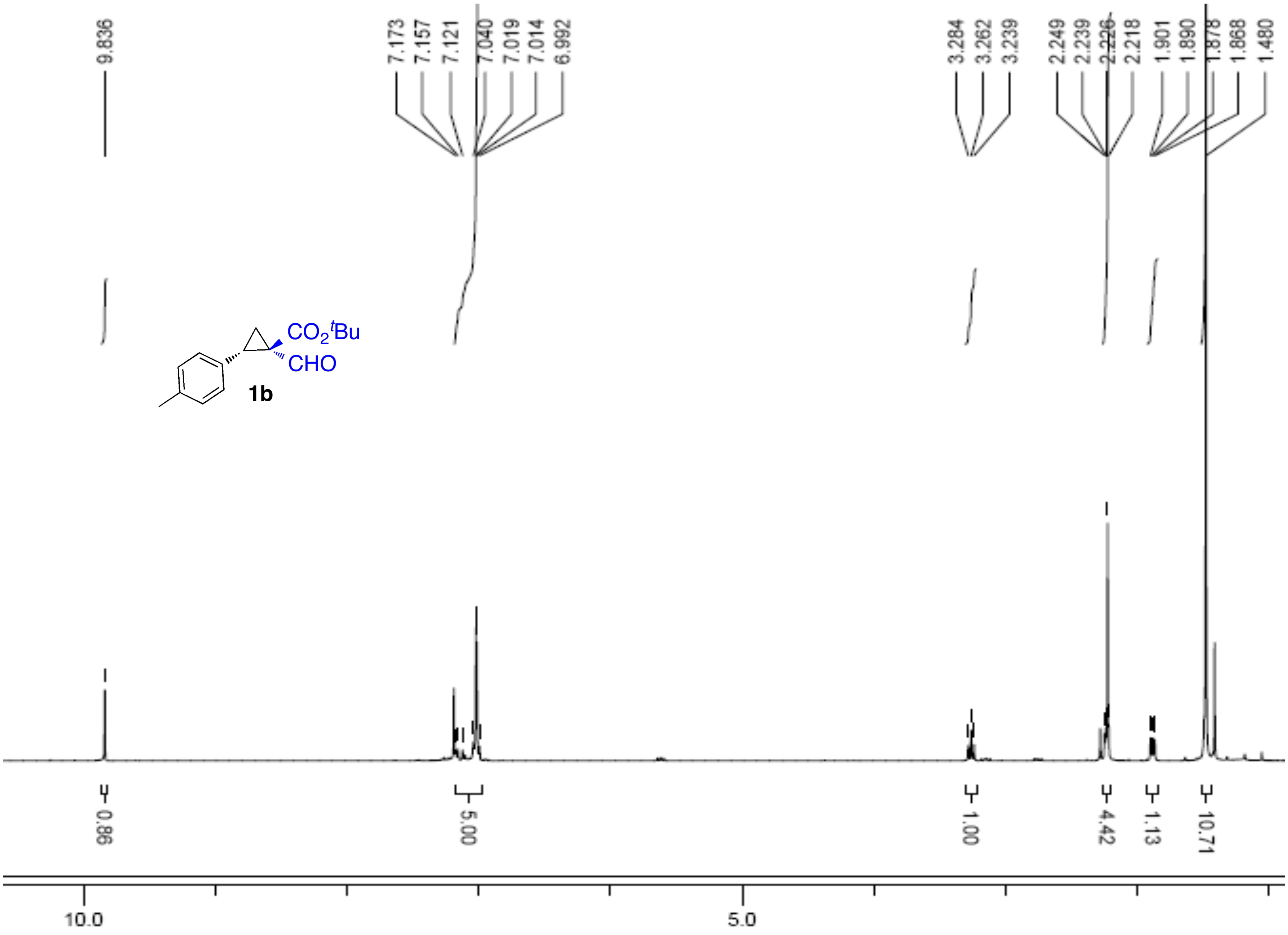
9.836
7.173
7.157
7.121
7.040
7.019
7.014
6.992
3.284
3.262
3.239
2.249
2.239
2.226
2.218
1.901
1.890
1.878
1.868
1.480
$CO_2{}^tBu$
CHO
1b
0.86
5.00
1.00
4.42
1.13
10.71
10.0
5.0

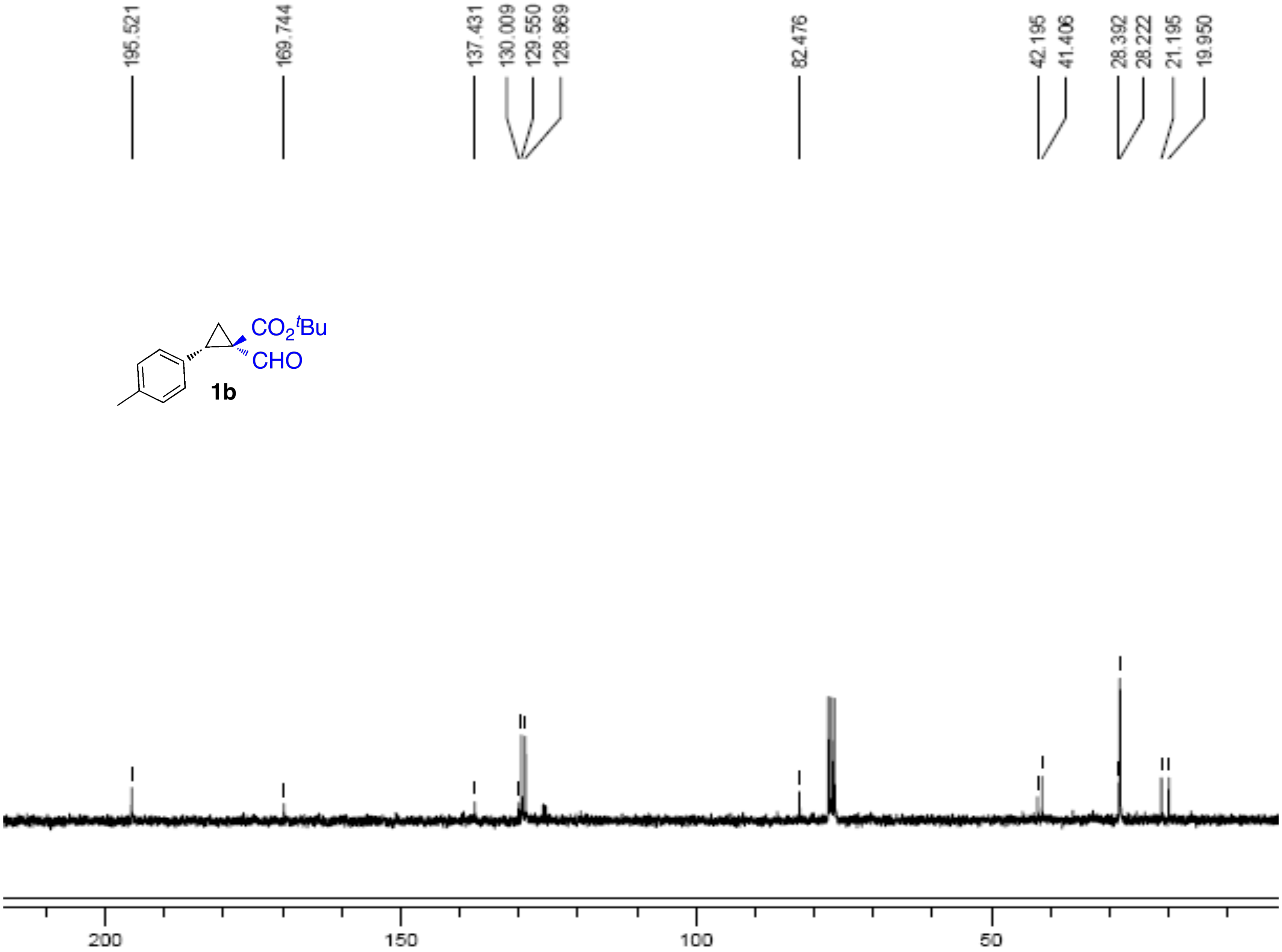
195.521
169.744
137.431
130.009
129.550
128.869
82.476
42.195
41.406
28.392
28.222
21.195
19.950
$CO_2{}^tBu$
CHO
1b
200
150
100
50

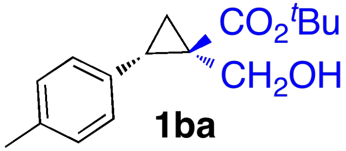
$CO_2{}^tBu$
CH_2OH

1ba

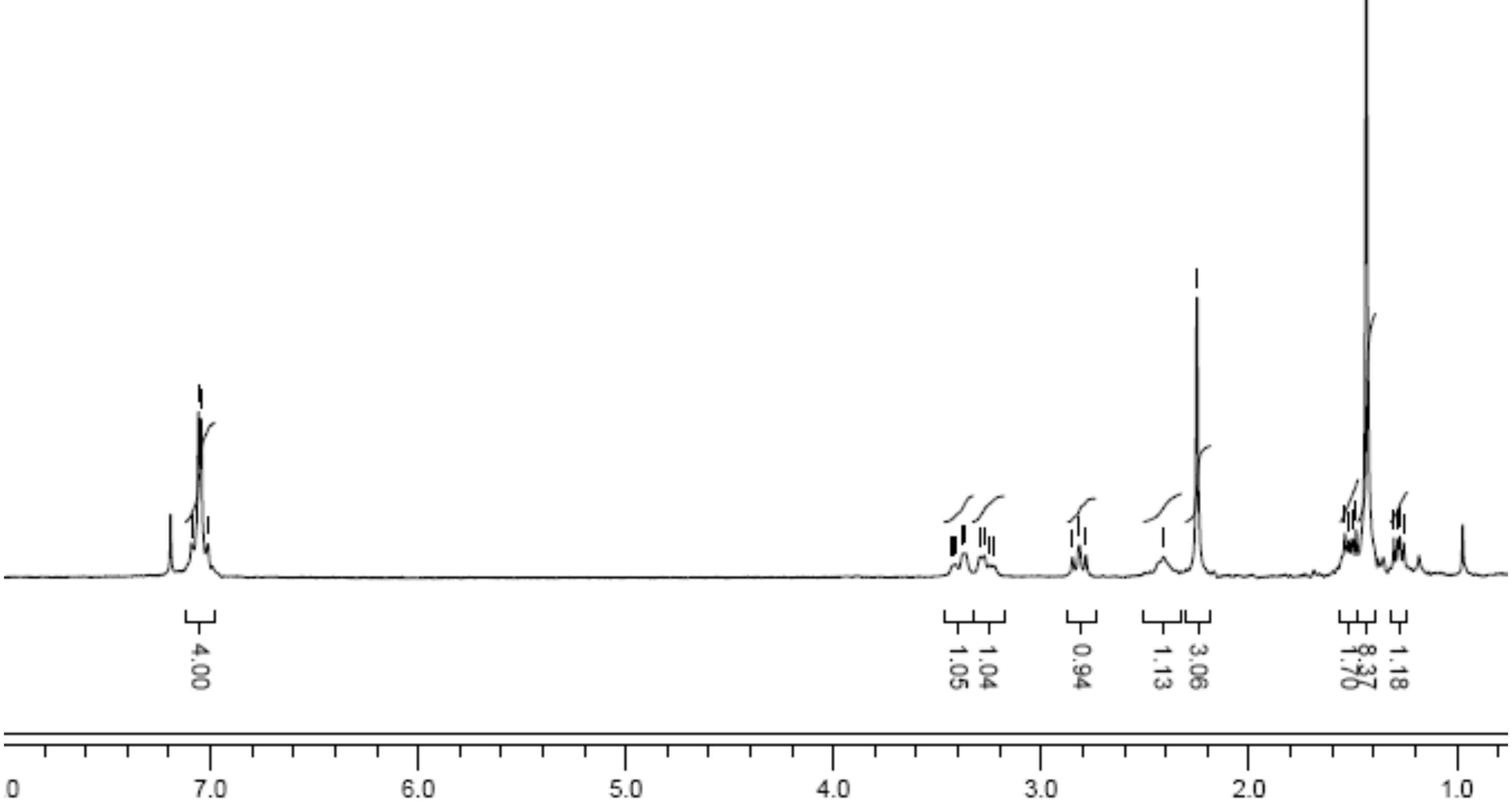
7.090
7.081
7.057
7.045
7.012
3.429
3.425
3.415
3.413
3.379
3.373
3.367
3.363
3.295
3.275
3.247
3.225
2.849
2.816
2.785
2.414
2.412
2.411
2.250
1.537
1.518
1.502
1.484
4.00
1.05
1.04
0.94
1.13
3.06
1.18
7.0
6.0
5.0
4.0
3.0
2.0
1.0

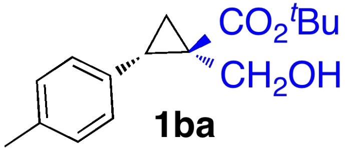

$CO_2{}^tBu$
CH_2OH

1ba

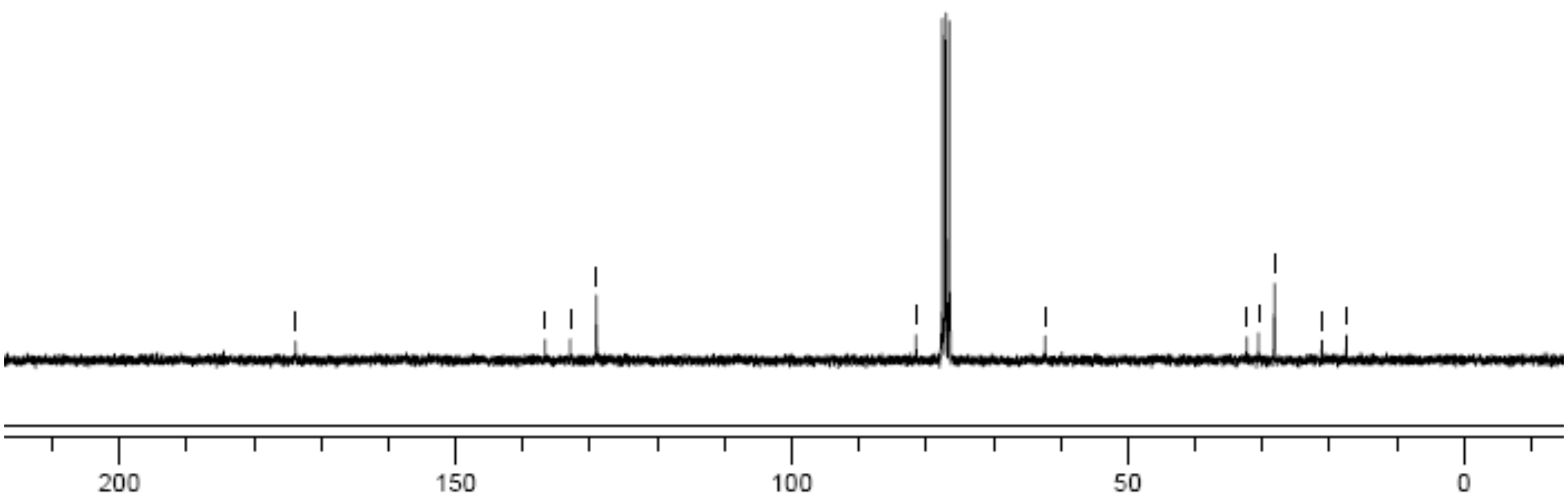

173.830
136.697
132.947
129.087
129.069
81.488
62.312
32.326
30.565
28.207
21.147
17.498
200
150
100
50
0

xx-vi-112 whelk 1%@0.8 ml
C:\EZStart\Projects\Default\Method\lmj-adh1&.met
C:\EZStart\Projects\Default\Data\xx-vi-112 whelk 1%@0.8 ml

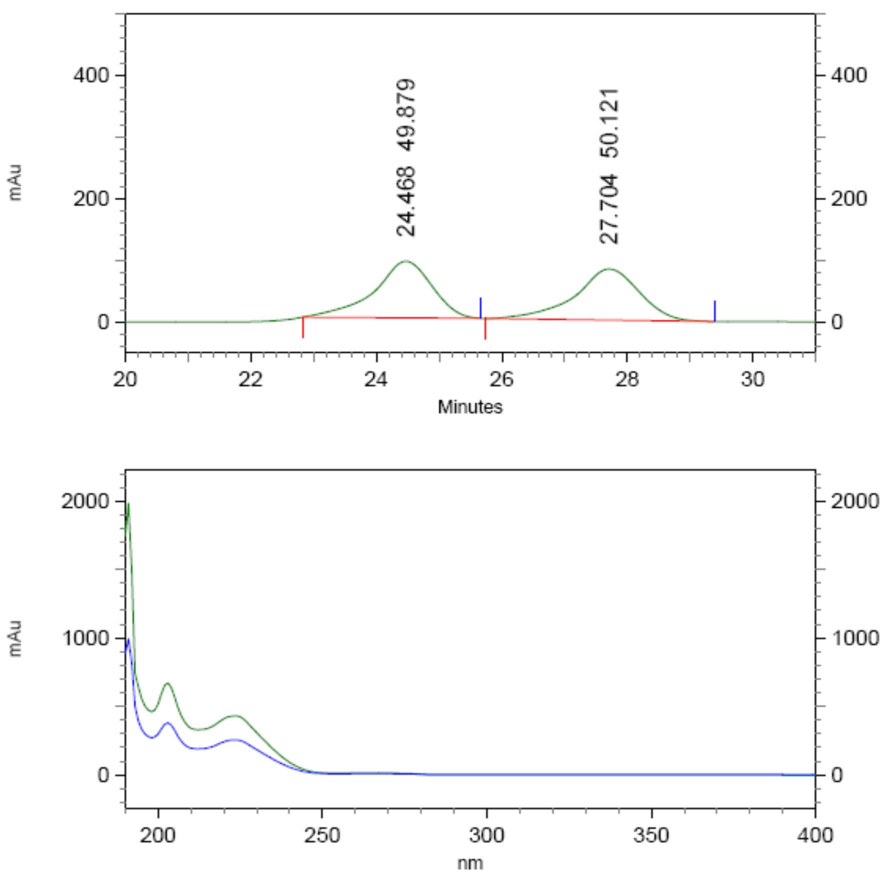

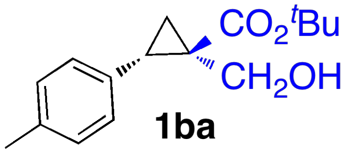

1ba

8: 242 nm, 4 nm
Results

Pk #	Name	Retention Time	Area Percent
1		24.468	49.879
2		27.704	50.121
Totals			100.000

xx-vi-113 whelk 1%@0.8 ml
C:\EZStart\Projects\Default\Method\lmj-adh1&.met
C:\EZStart\Projects\Default\Data\xx-vi-113 whelk 1%@0.8 ml

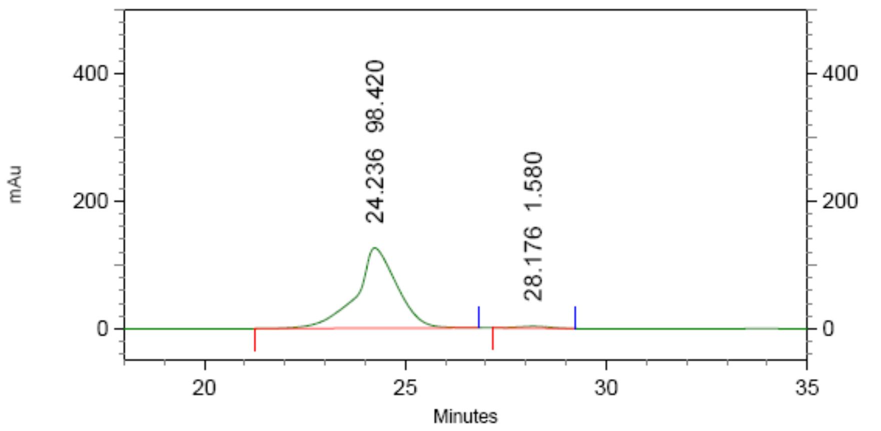

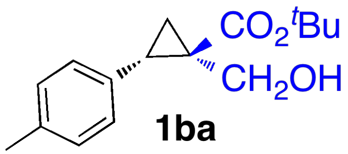
1ba

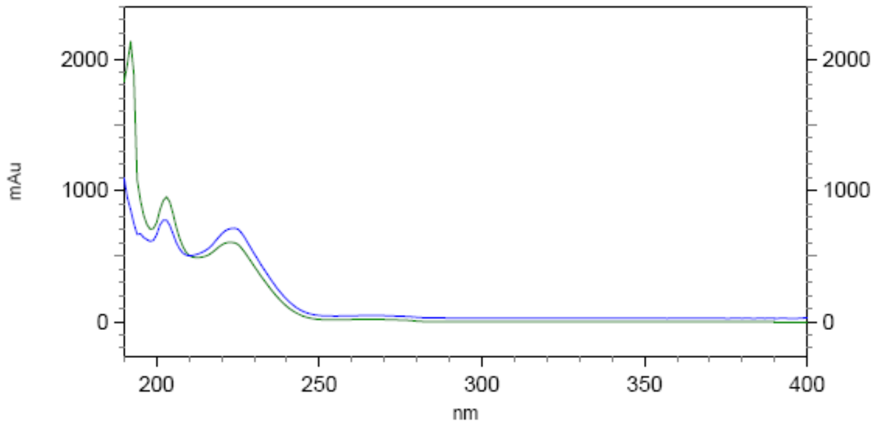

8: 232 nm, 4 nm
Results

Pk #	Name	Retention Time	Area Percent
1		24.236	98.420
2		28.176	1.580

Totals	
	100.000

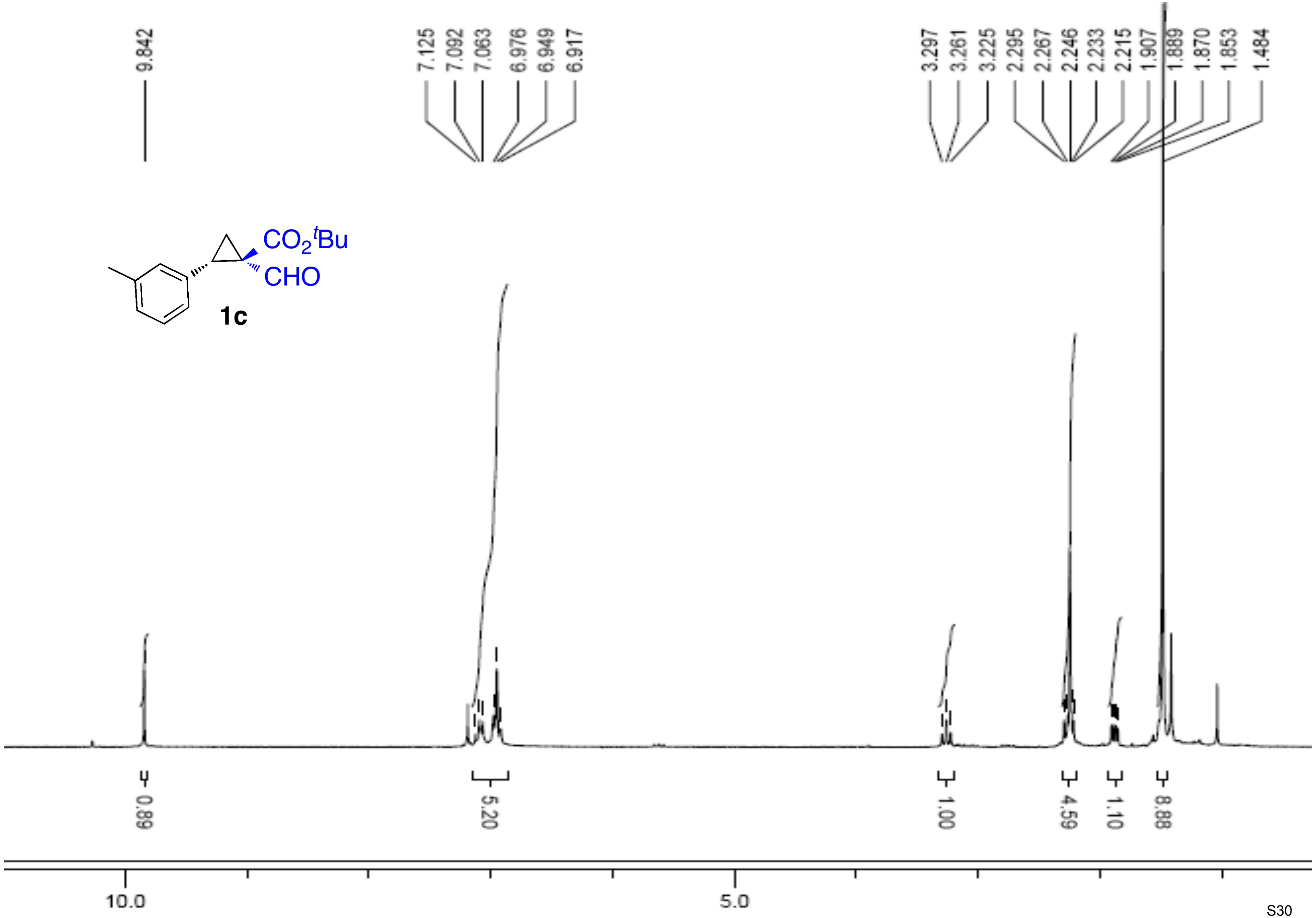
9.842
7.125
7.092
7.063
6.976
6.949
6.917
3.297
3.261
3.225
2.295
2.267
2.246
2.233
2.215
1.907
1.889
1.870
1.853
1.484
$CO_2{}^tBu$
CHO
1c
0.89
5.20
1.00
4.59
1.10
8.88
10.0
5.0

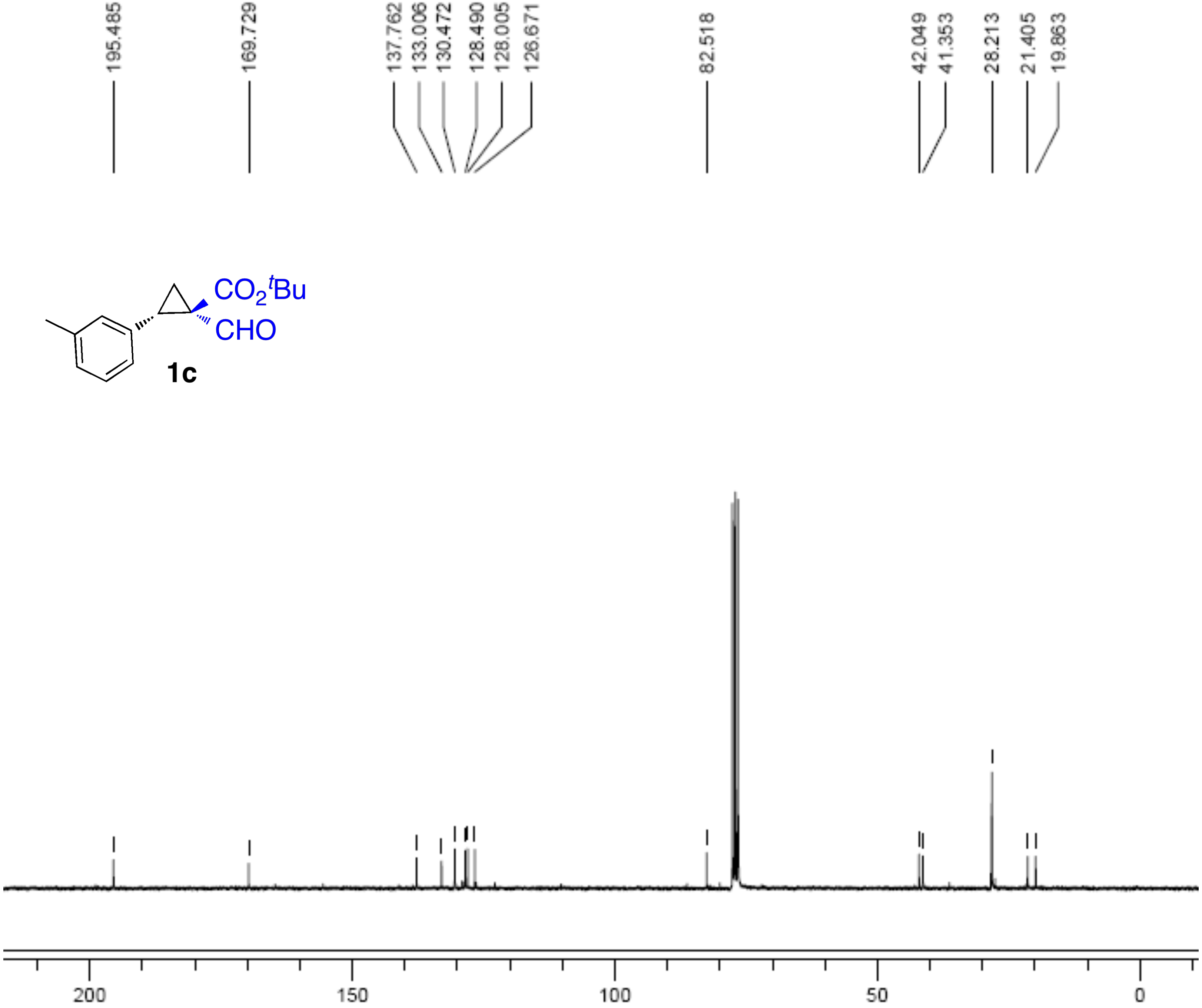

195.485
169.729
137.762
133.006
130.472
128.490
128.005
126.671
82.518
42.049
41.353
28.213
21.405
19.863
200
150
100
50
0
$CO_2{}^tBu$
CHO

1c

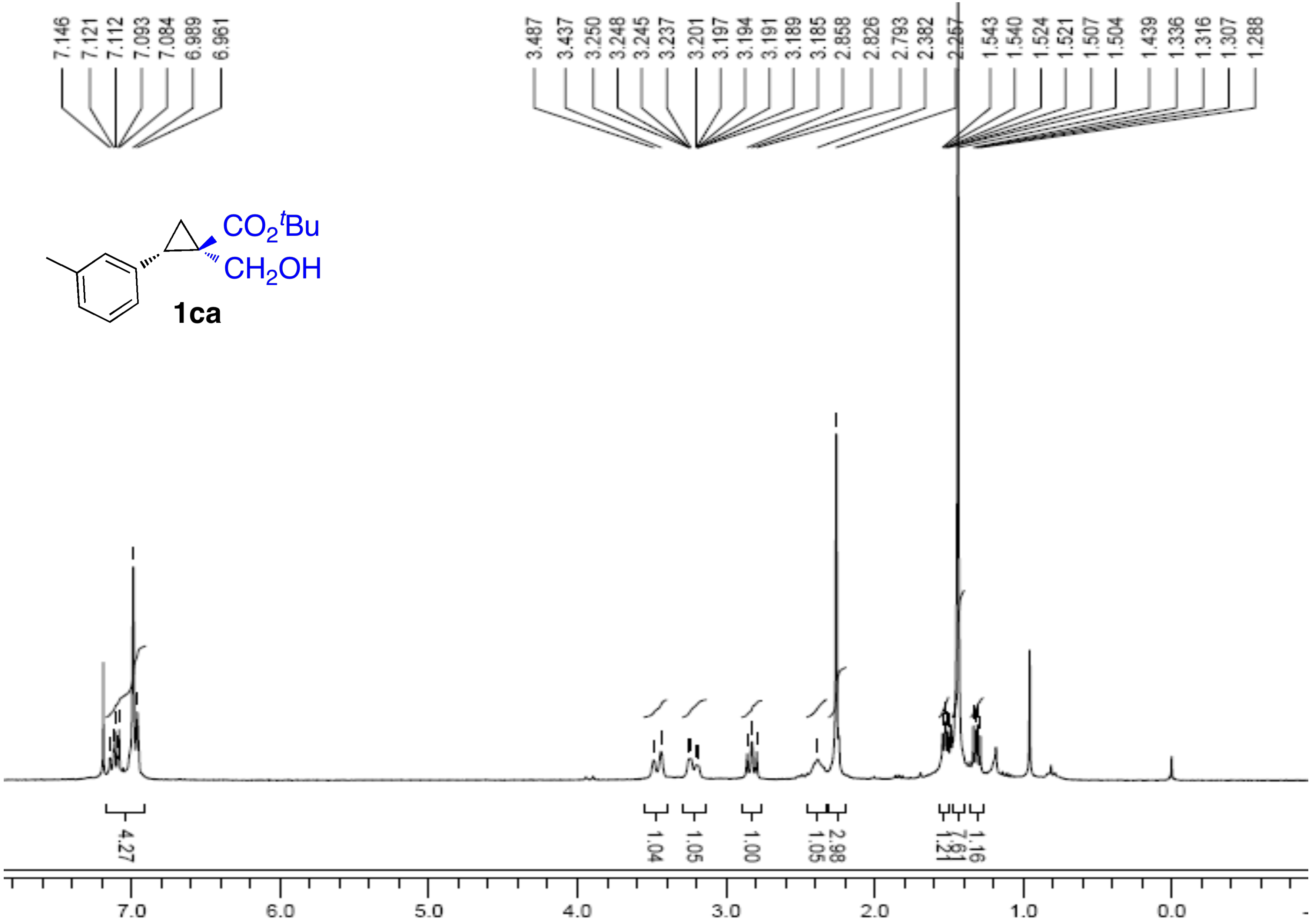
7.146
7.121
7.112
7.093
7.084
6.989
6.961
3.487
3.437
3.250
3.248
3.245
3.237
3.201
3.197
3.194
3.191
3.189
3.185
2.858
2.826
2.793
2.382
2.257
1.543
1.540
1.524
1.521
1.507
1.504
1.439
1.336
1.316
1.307
1.288
CO2tBu
CH2OH
1ca
4.27
1.04
1.05
1.00
1.05
2.98
1.21
7.61
1.16
7.0
6.0
5.0
4.0
3.0
2.0
1.0
0.0

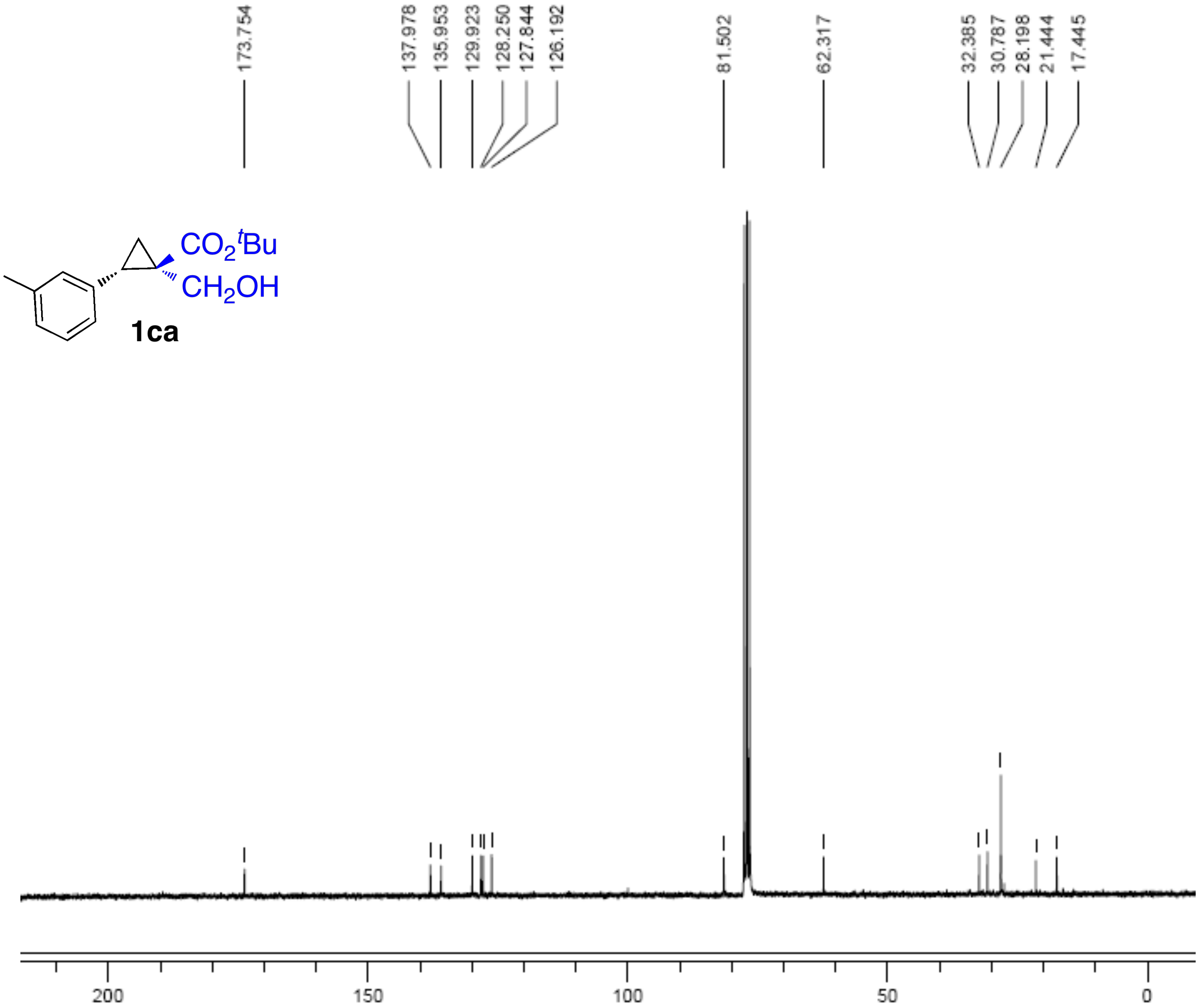
173.754
137.978
135.953
129.923
128.250
127.844
126.192
81.502
62.317
32.385
30.787
28.198
21.444
17.445
$CO_2{}^tBu$
CH_2OH
1ca
200
150
100
50
0

xx-vii-293 ADH 1%@0.8 ml
C:\EZStart\Projects\Default\Method\Shifa-P-Cyclopropane 1%Whelk.met
C:\EZStart\Projects\Default\Data\xx-vii-293 ADH 1%@0.8 ml

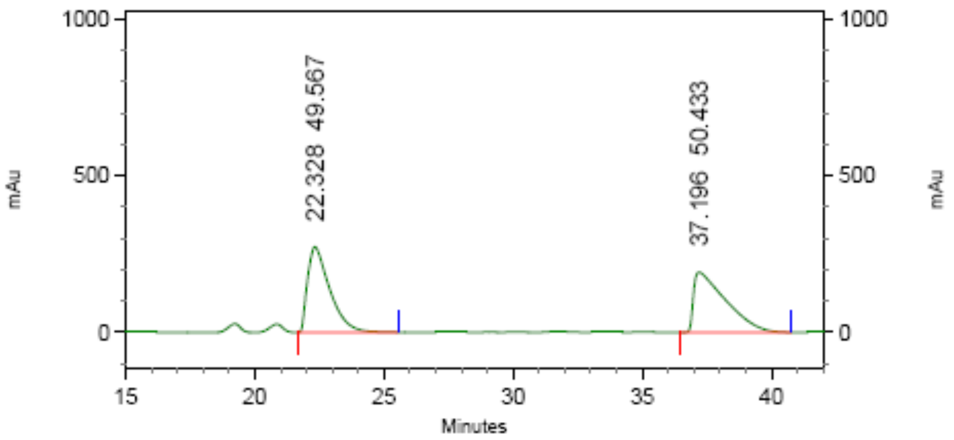

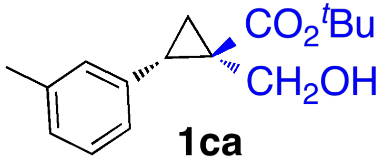

1ca

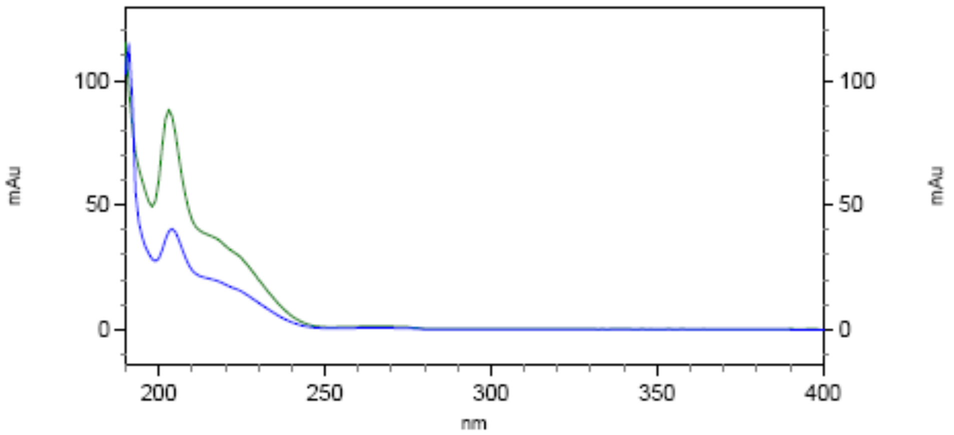

2: 225 nm, 4 nm
Results

Pk # Name	Retention Time	Area Percent
1	22.328	49.567
2	37.196	50.433
Totals		100.000

xx-vi-121 ADH 1%@0.8 ml
C:\EZStart\Projects\Default\Method\Shifa-P-Cyclopropane 1%Whelk.met
C:\EZStart\Projects\Default\Data\xx-vi-122 ADH 1%@0.8 ml

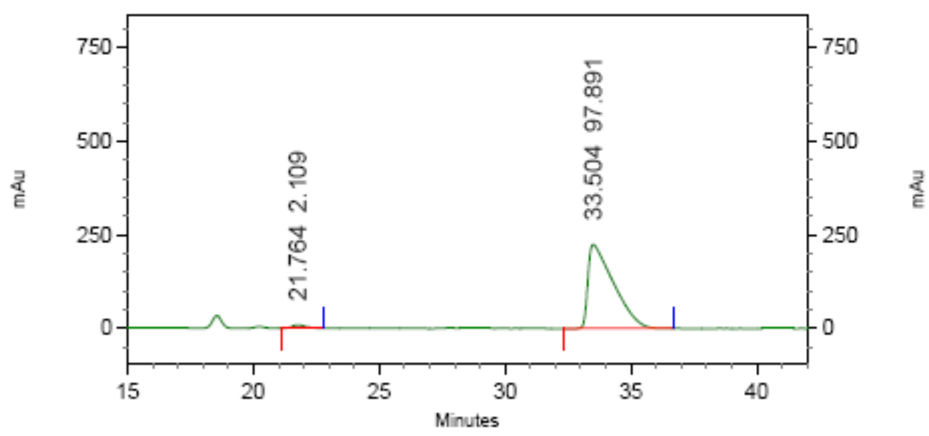

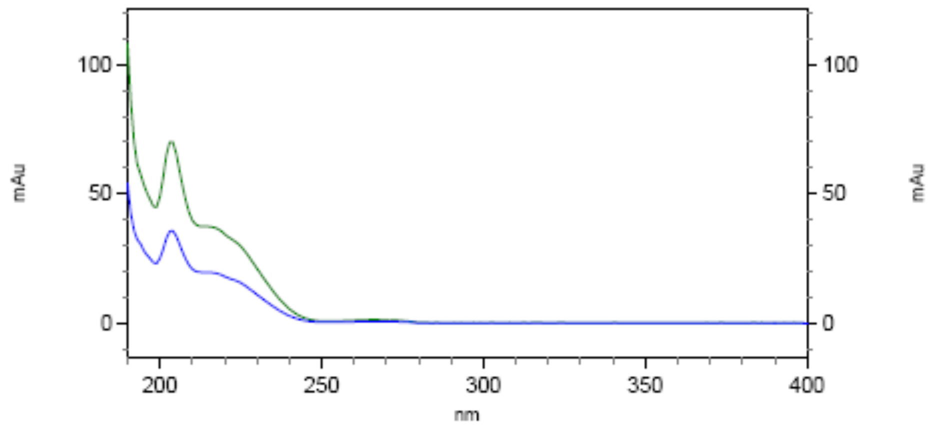

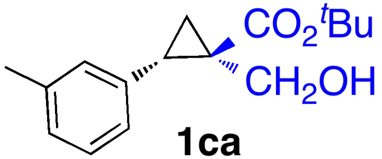

1ca

2: 225 nm, 4 nm
Results

Pk # Name	Retention Time	Area Percent
1	21.764	2.109
2	33.504	97.891
Totals		100.000

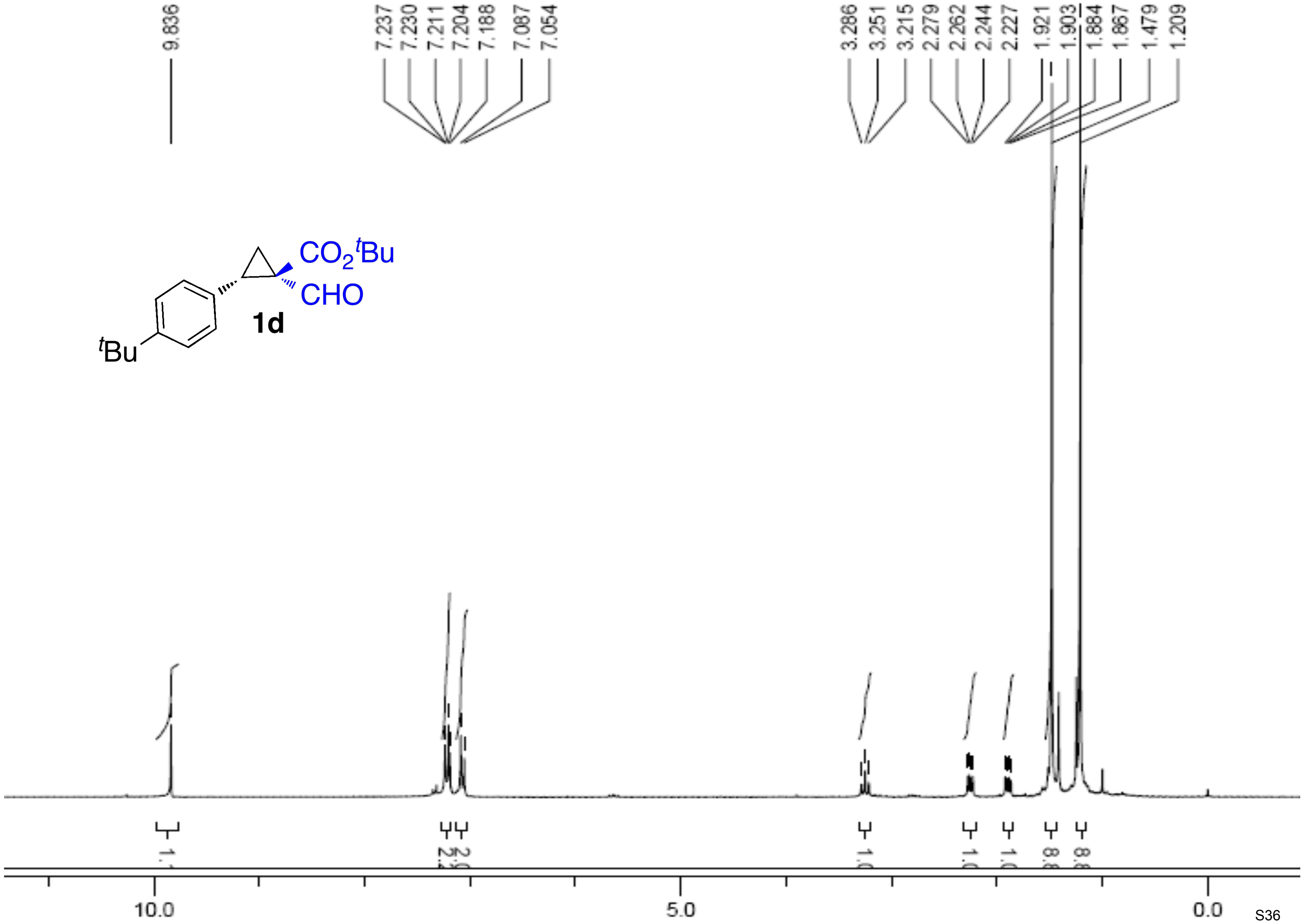
9.836
7.237 7.230 7.211 7.204 7.188 7.087 7.054
3.286 3.251 3.215 2.279 2.262 2.244 2.227 1.921 1.903 1.884 1.867 1.479 1.209
CO2tBu
CHO
tBu
1d
10.0
5.0
0.0

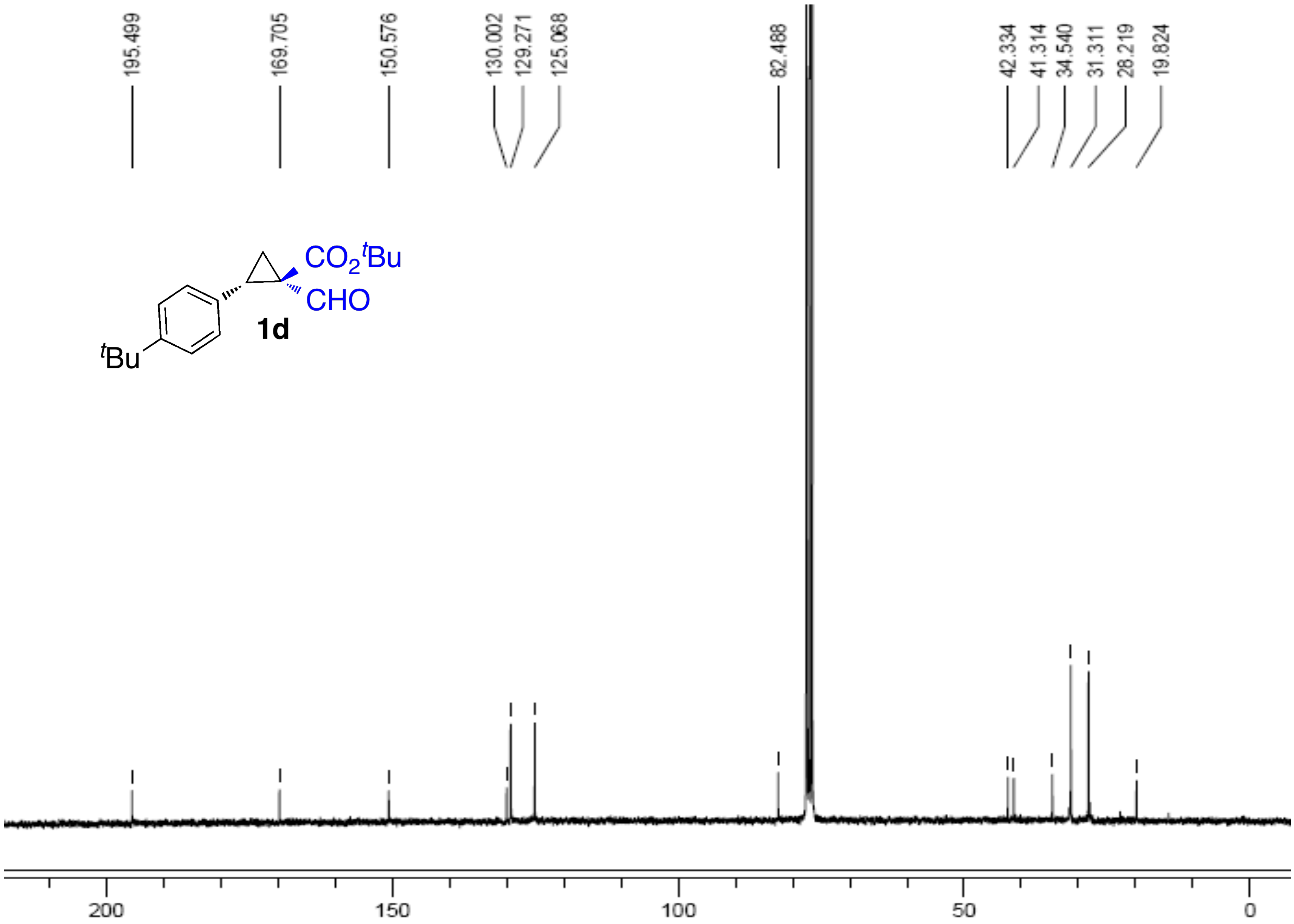
195.499
169.705
150.576
130.002
129.271
125.068
82.488
42.334
41.314
34.540
31.311
28.219
19.824
$CO_2{}^tBu$
CHO
^tBu
1d
200
150
100
50
0

xx-vi-121SD whelk 1%@0.8 ml
C:\EZStart\Projects\Default\Method\Shifa-EDA.met
E:\HPLC\xx-vi-121SD whelk 1%@0.8 ml

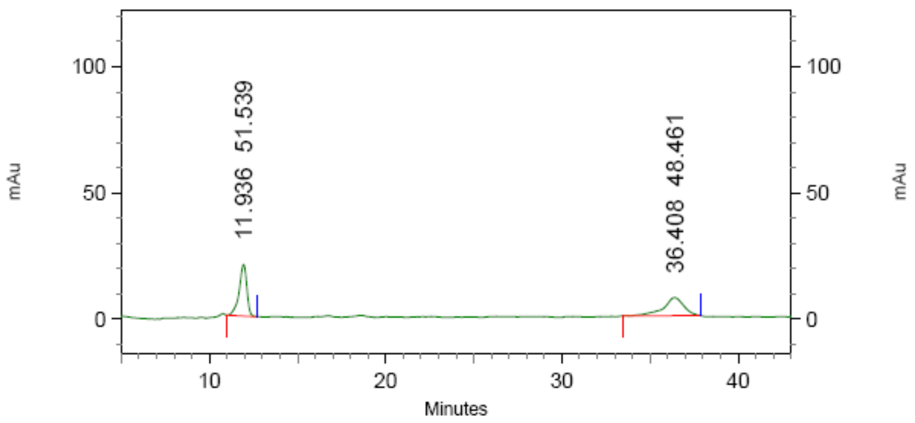

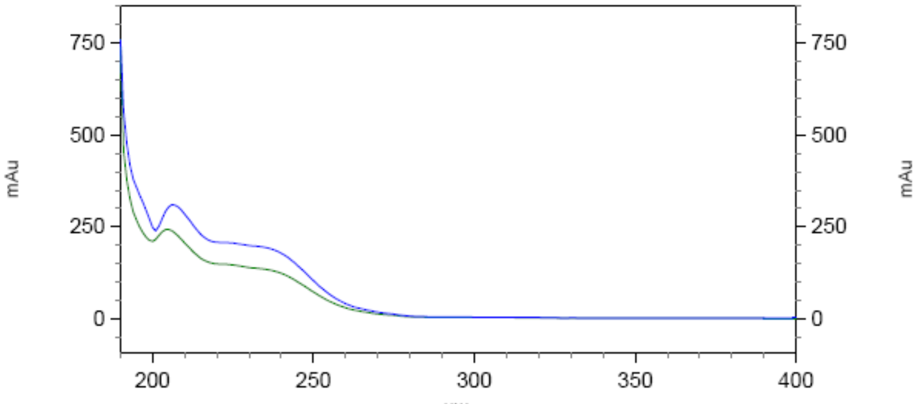

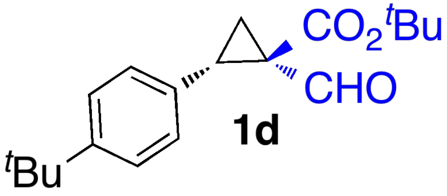

1d

1: 224 nm, 4 nm
Results

Pk #	Name	Retention Time	Area Percent
1		11.936	51.539
2		36.408	48.461
Totals			100.000

xx-vi-120SD whelk 1%@0.8 ml
C:\EZStart\Projects\Default\Method\Shifa-EDA.met
C:\EZStart\Projects\Default\Data\xx-vi-120SD whelk 1%@0.8 ml

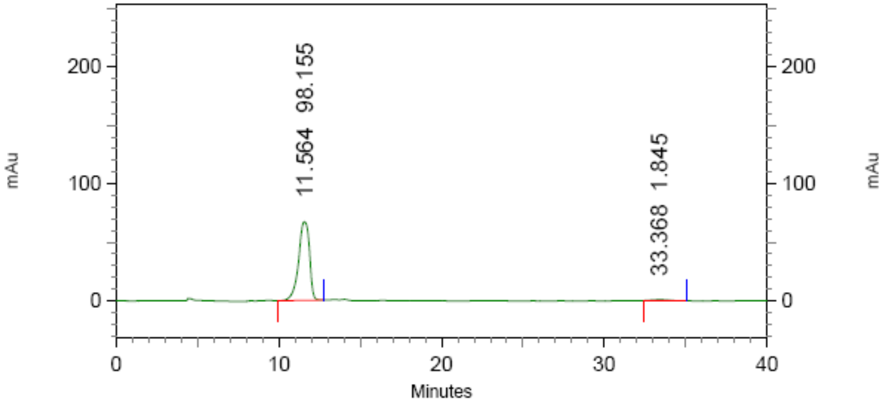

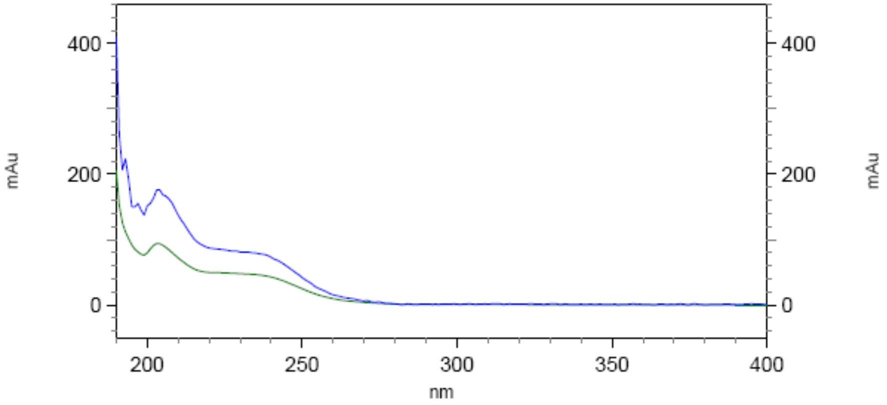

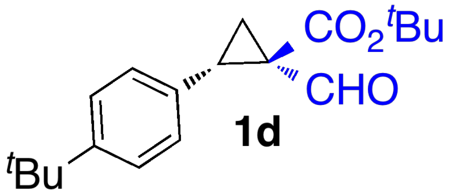

1d

2: 250 nm, 4 nm
Results

Pk #	Name	Retention Time	Area Percent
1		11.564	98.155
2		33.368	1.845
Totals			100.000

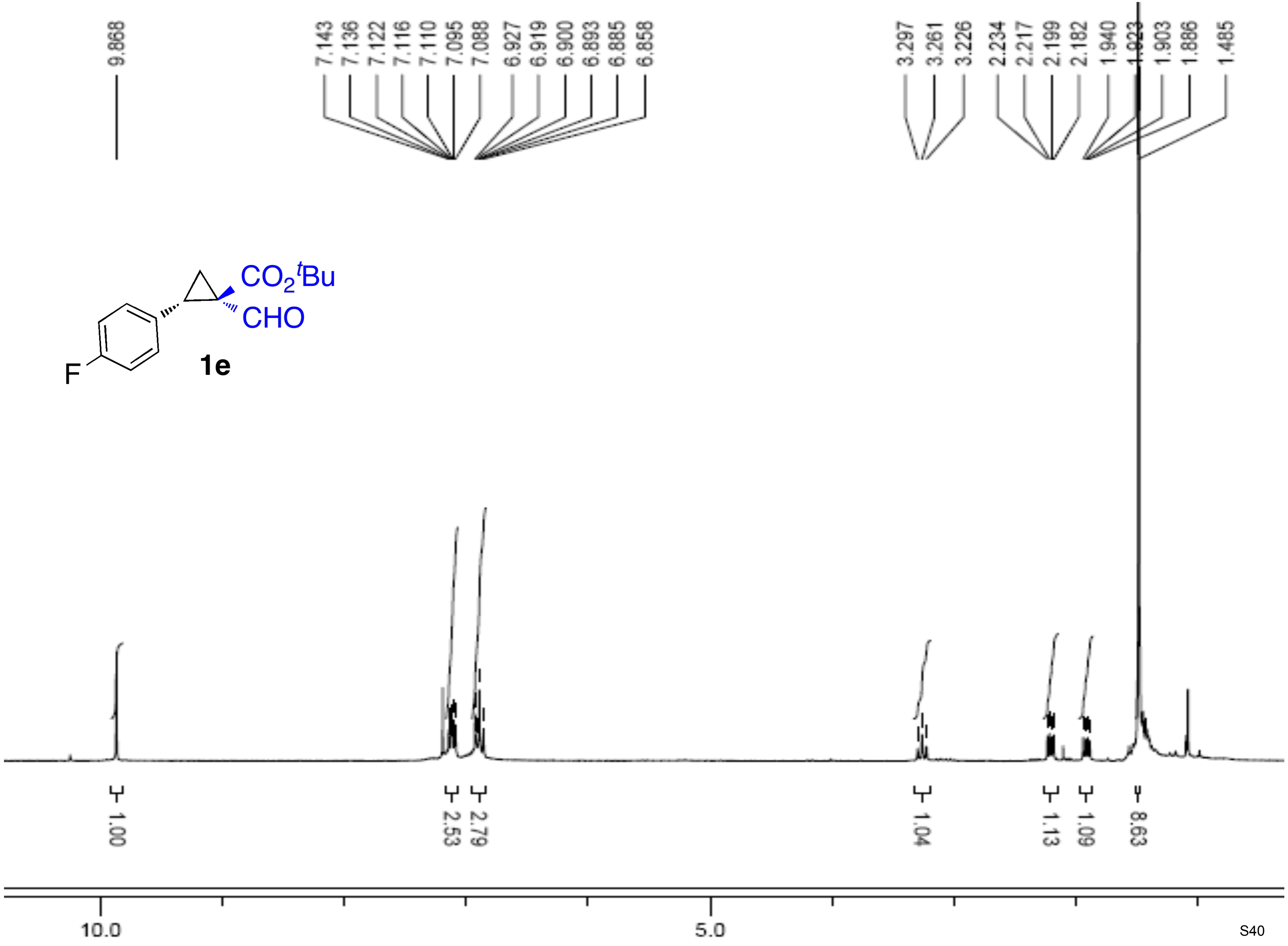
9.868
7.143
7.136
7.122
7.116
7.110
7.095
7.088
6.927
6.919
6.900
6.893
6.885
6.858
3.297
3.261
3.226
2.234
2.217
2.199
2.182
1.940
1.923
1.903
1.886
1.485
CO2tBu
CHO
F
1e
1.00
2.53
2.79
1.04
1.13
1.09
8.63
10.0
5.0

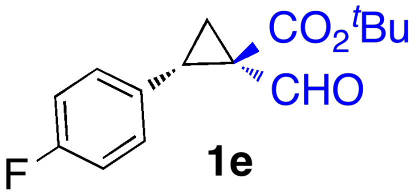
CO2tBu
CHO
F

1e

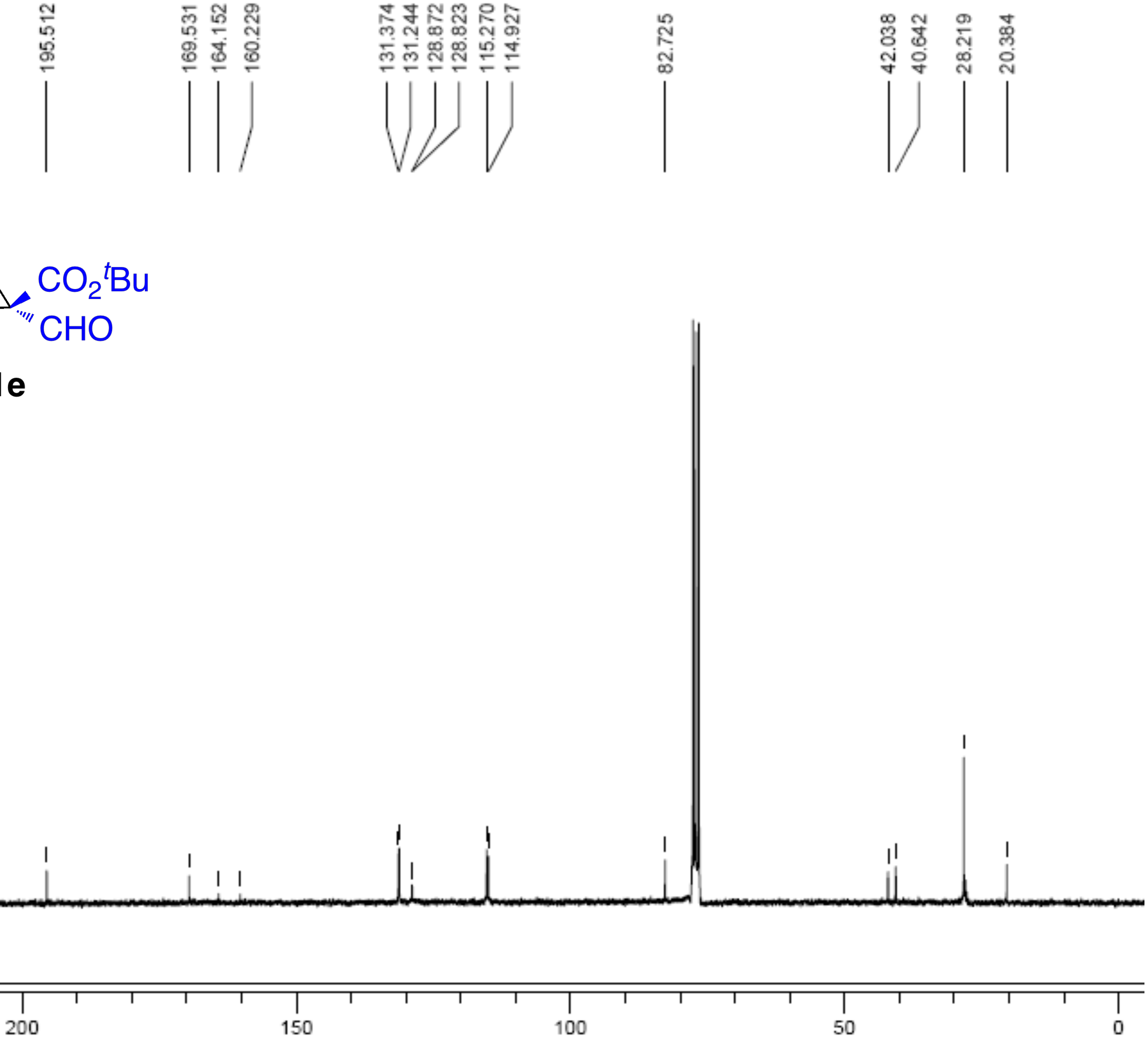
195.512
169.531
164.152
160.229
131.374
131.244
128.872
128.823
115.270
114.927
82.725
42.038
40.642
28.219
20.384
200
150
100
50
0

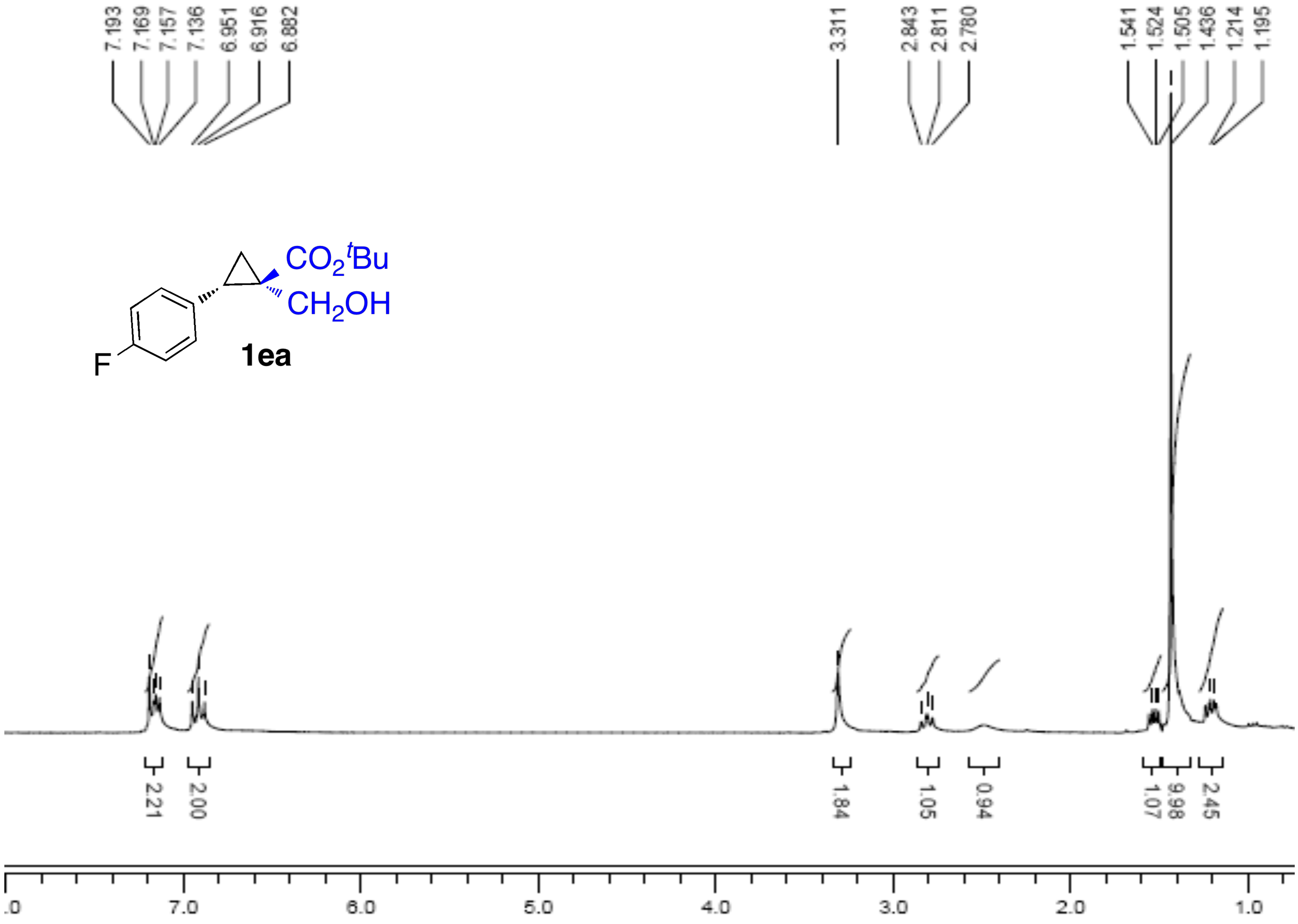
7.193
7.169
7.157
7.136
6.951
6.916
6.882
3.311
2.843
2.811
2.780
1.541
1.524
1.505
1.436
1.214
1.195
CO2tBu
CH2OH
F
1ea
2.21
2.00
1.84
1.05
0.94
1.07
9.98
2.45
.0
7.0
6.0
5.0
4.0
3.0
2.0
1.0

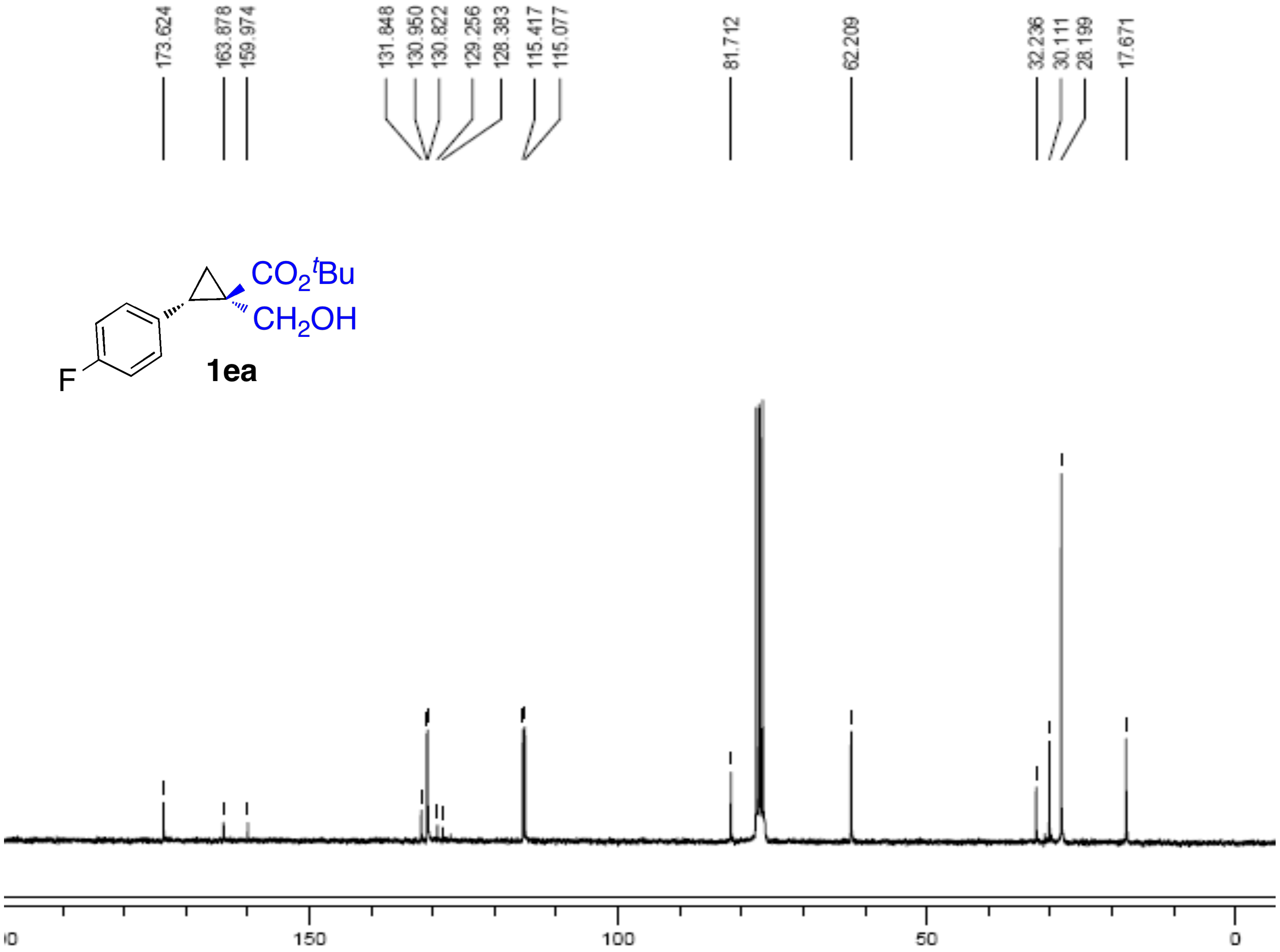
173.624
163.878
159.974
131.848
130.950
130.822
129.256
128.383
115.417
115.077
81.712
62.209
32.236
30.111
28.199
17.671
CO2tBu
CH2OH
F
1ea
150
100
50
0

xx-vii-295 ADH 1%@0.8 ml
C:\EZStart\Projects\Default\Method\Shifa 4-t-Butylbenzene (OD-H 10-90).met
C:\EZStart\Projects\Default\Data\xx-vii-295 ADH 1%@0.8 ml

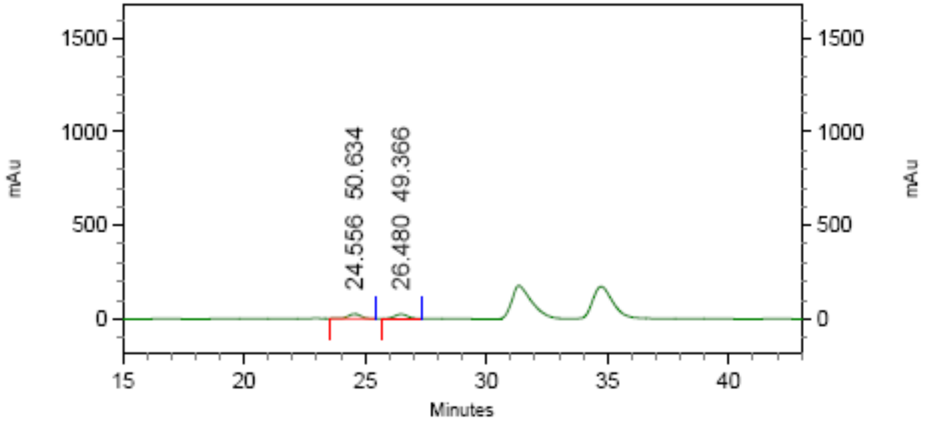

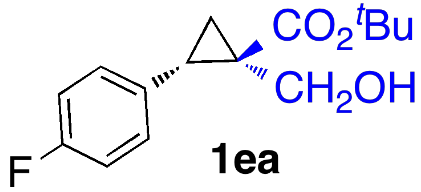

1ea

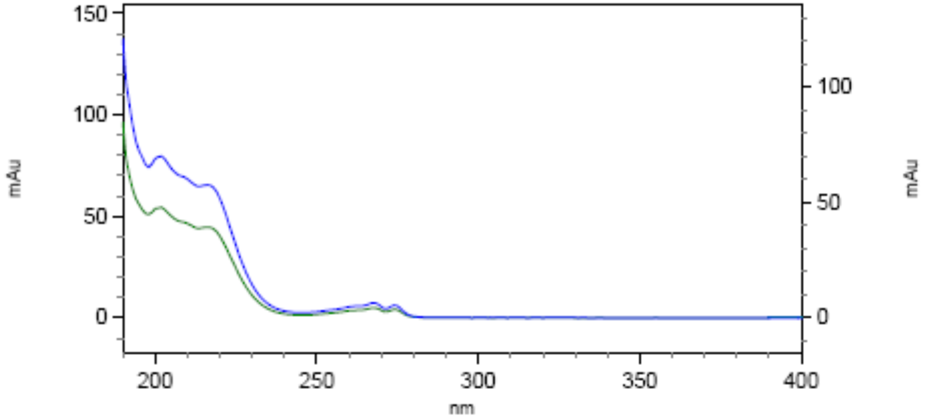

3: 216 nm, 4 nm

Results

Pk #	Name	Retention Time	Area Percent
1		24.556	50.634
2		26.480	49.366
Totals			100.000

xx-vii-299 ADH 1%@0.8 ml again
C:\EZStart\Projects\Default\Method\Shifa 4-t-Butylbenzene (OD-H 10-90).met
C:\EZStart\Projects\Default\Data\xx-vii-299 ADH 1%@0.8 ml again

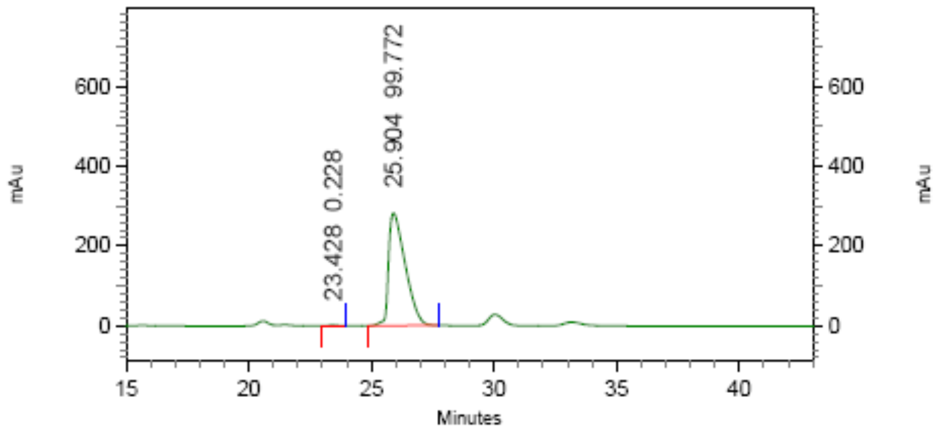

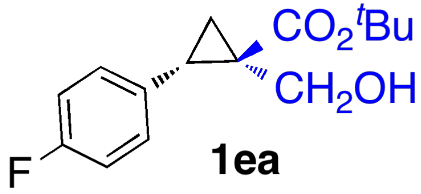

1ea

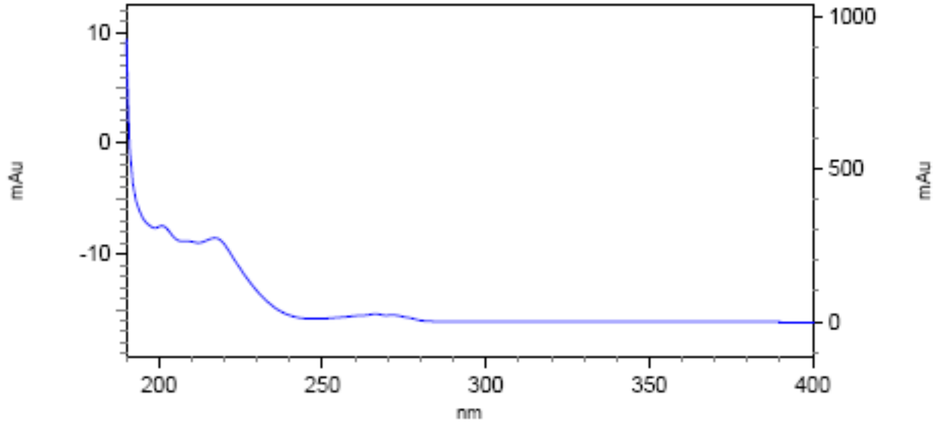

3: 229 nm, 4 nm
Results

Pk #	Name	Retention Time	Area Percent
1		23.428	0.228
2		25.904	99.772
Totals			100.000

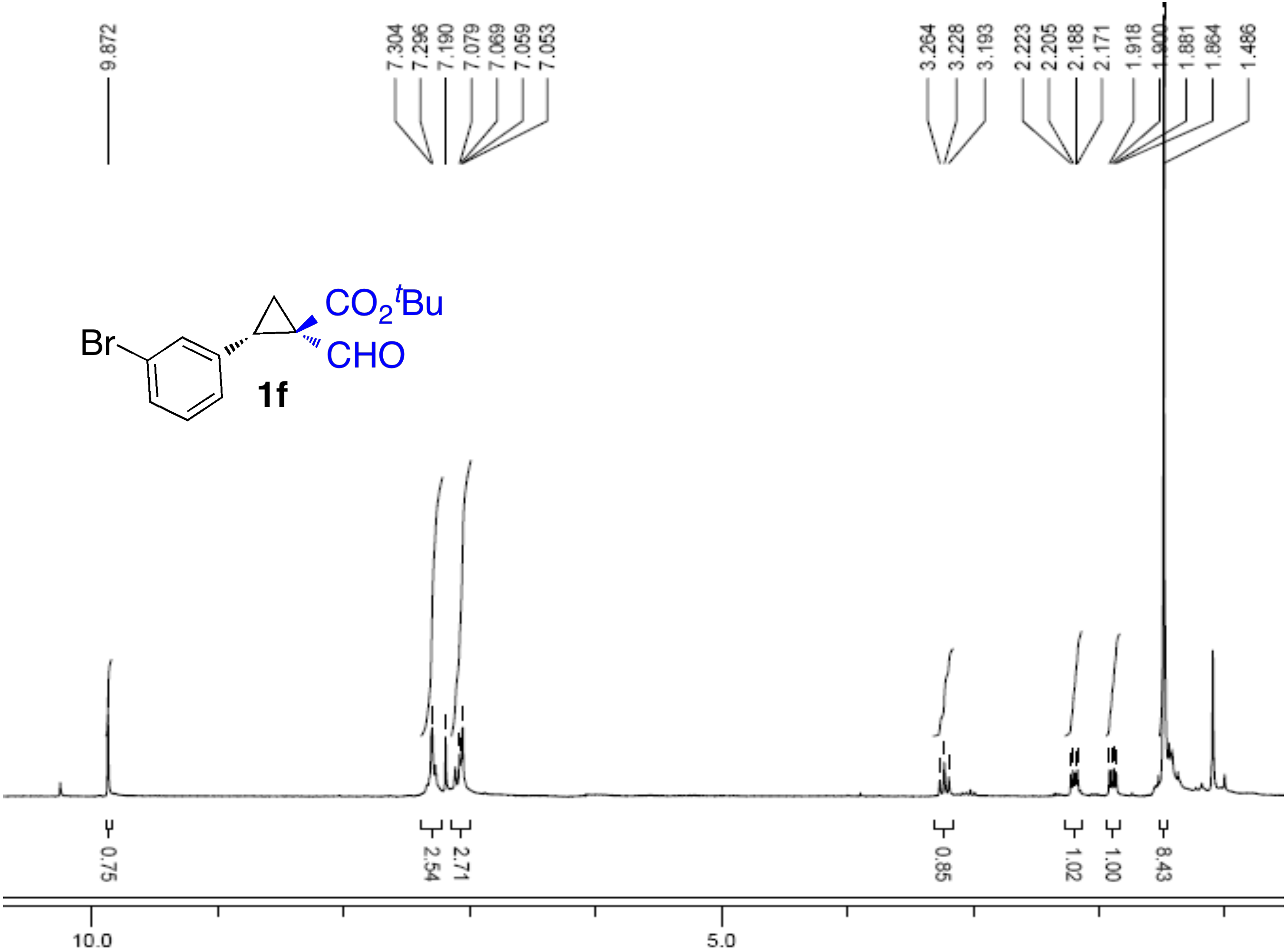
9.872
7.304
7.296
7.190
7.079
7.069
7.059
7.053
3.264
3.228
3.193
2.223
2.205
2.188
2.171
1.918
1.900
1.881
1.864
1.486
Br
CO2tBu
CHO
1f
0.75
2.54
2.71
0.85
1.02
1.00
8.43
10.0
5.0

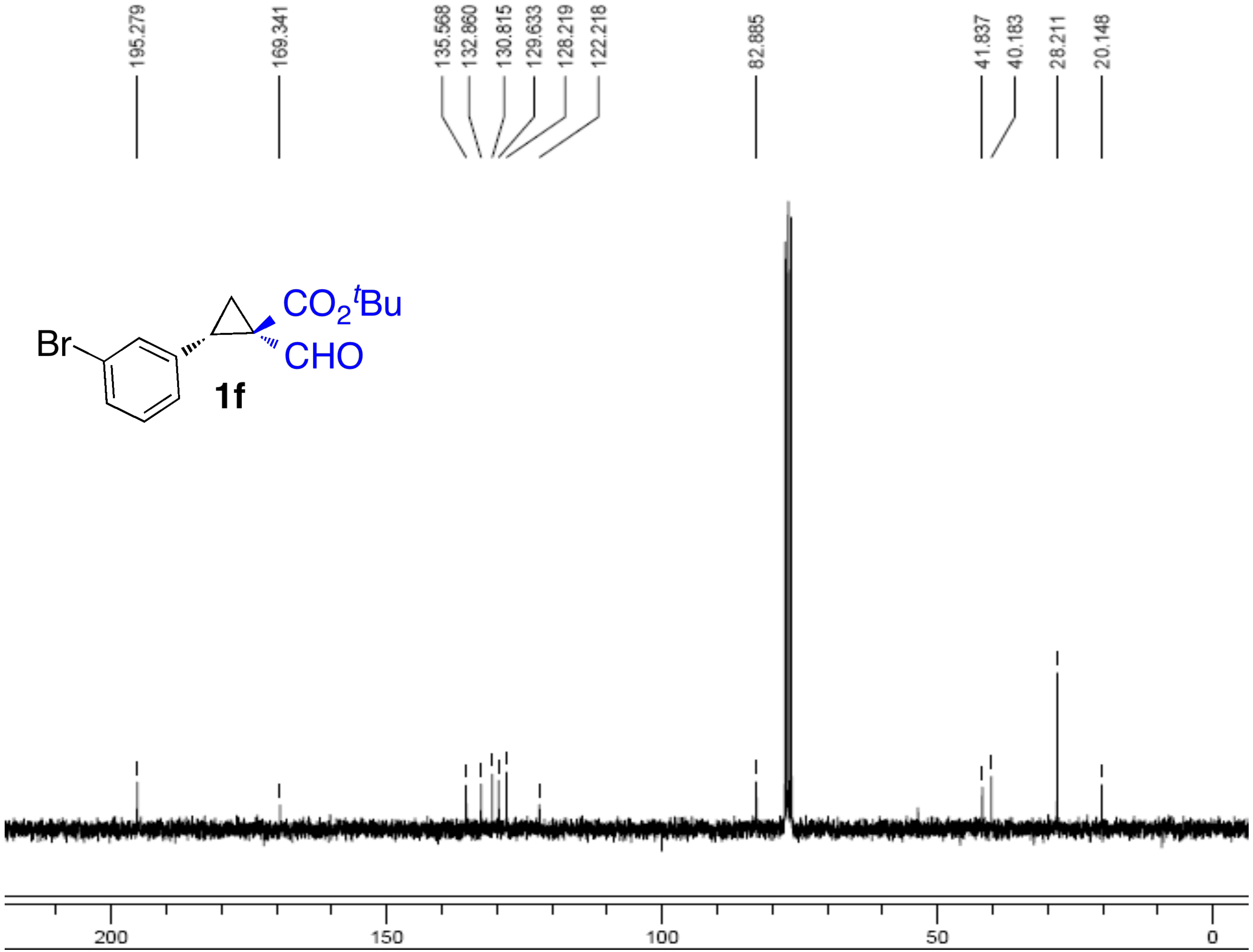

195.279
169.341
135.568
132.860
130.815
129.633
128.219
122.218
82.885
41.837
40.183
28.211
20.148
Br
$CO_2{}^tBu$
CHO
1f
200
150
100
50
0

xx-vi-67-2 SDwhelk 2%@0.8 ml
C:\EZStart\Projects\Default\Method\Shifa-Nitro-Cyclopropane-1%.met
C:\EZStart\Projects\Default\Data\xx-vi-67-2 SDwhelk 2%@0.8 ml

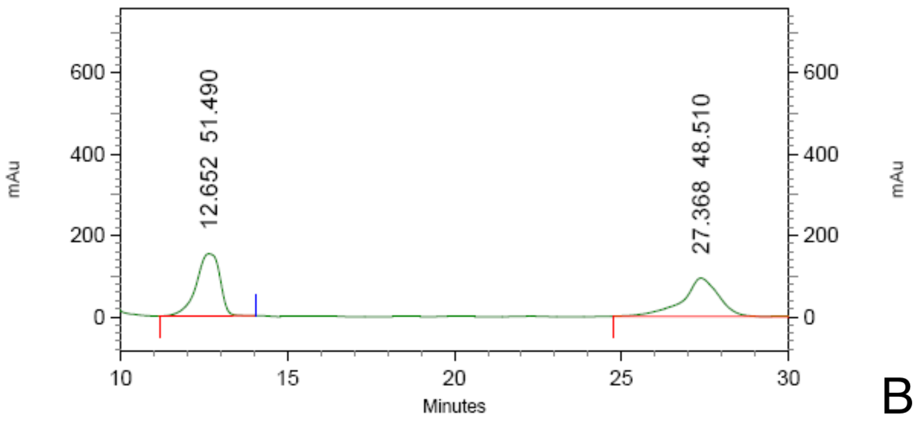

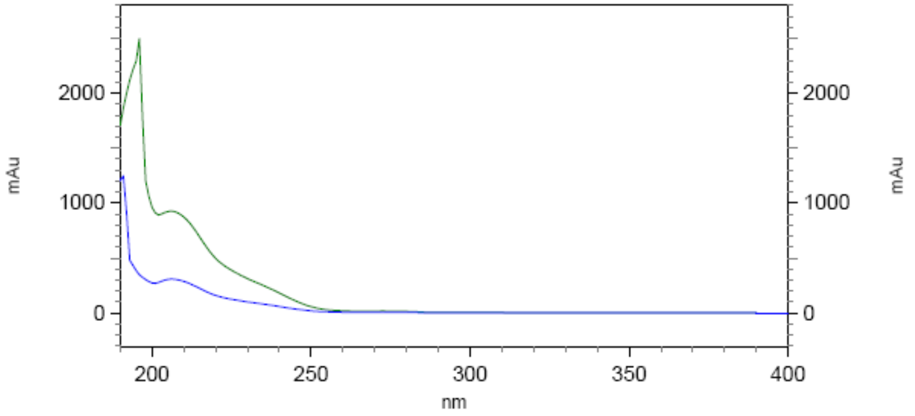

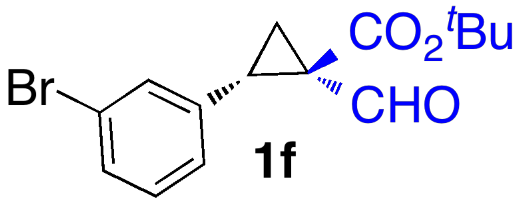

1f

4: 231 nm, 4 nm
Results

Pk #	Name	Retention Time	Area Percent
1		12.652	51.490
2		27.368	48.510
Totals			100.000

```
xx-vi-82 SDwhelk 2%@0.8 ml
C:\EZStart\Projects\Default\Method\Shifa-Nitro-Cyclopropane-1%.met
C:\EZStart\Projects\Default\Data\xx-vi-82 SDwhelk 2%@0.8 ml
```

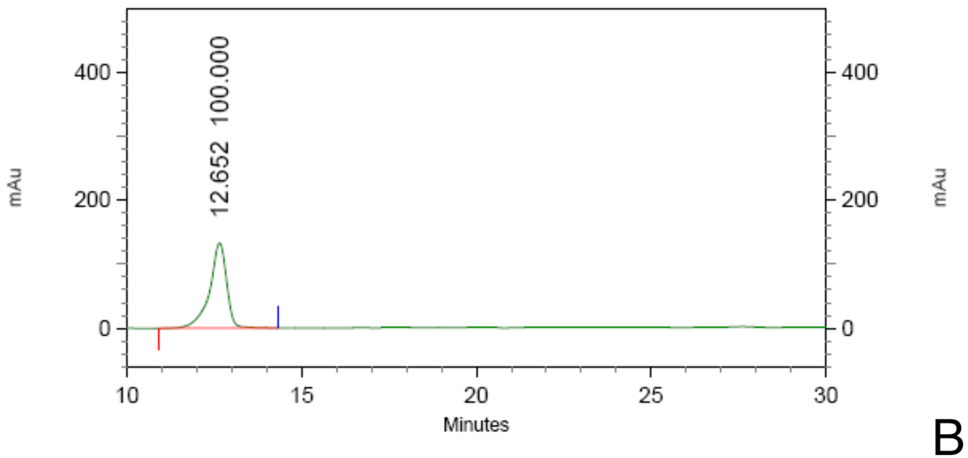

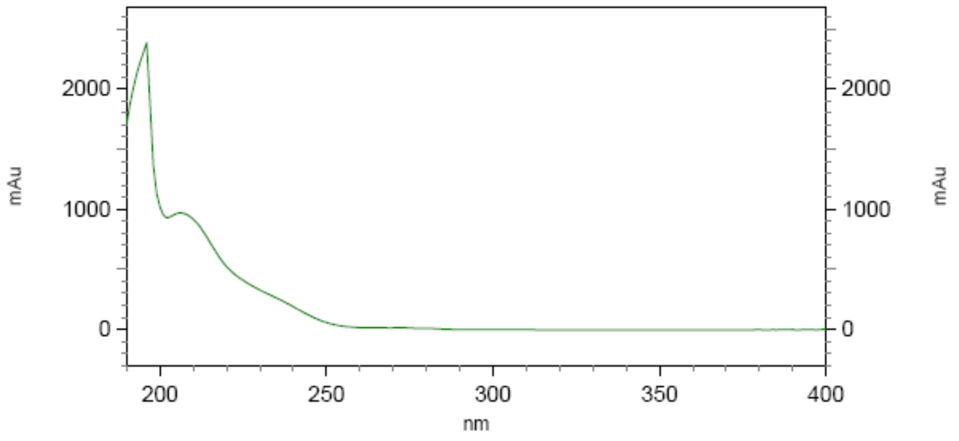

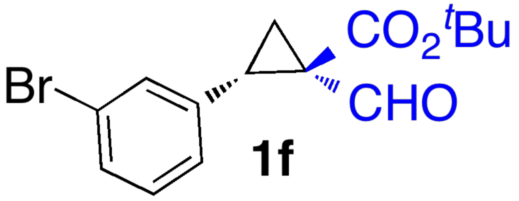

1f

4: 230 nm, 4 nm

Results

Pk #	Name	Retention Time	Area Percent
1		12.652	100.000
Totals			100.000

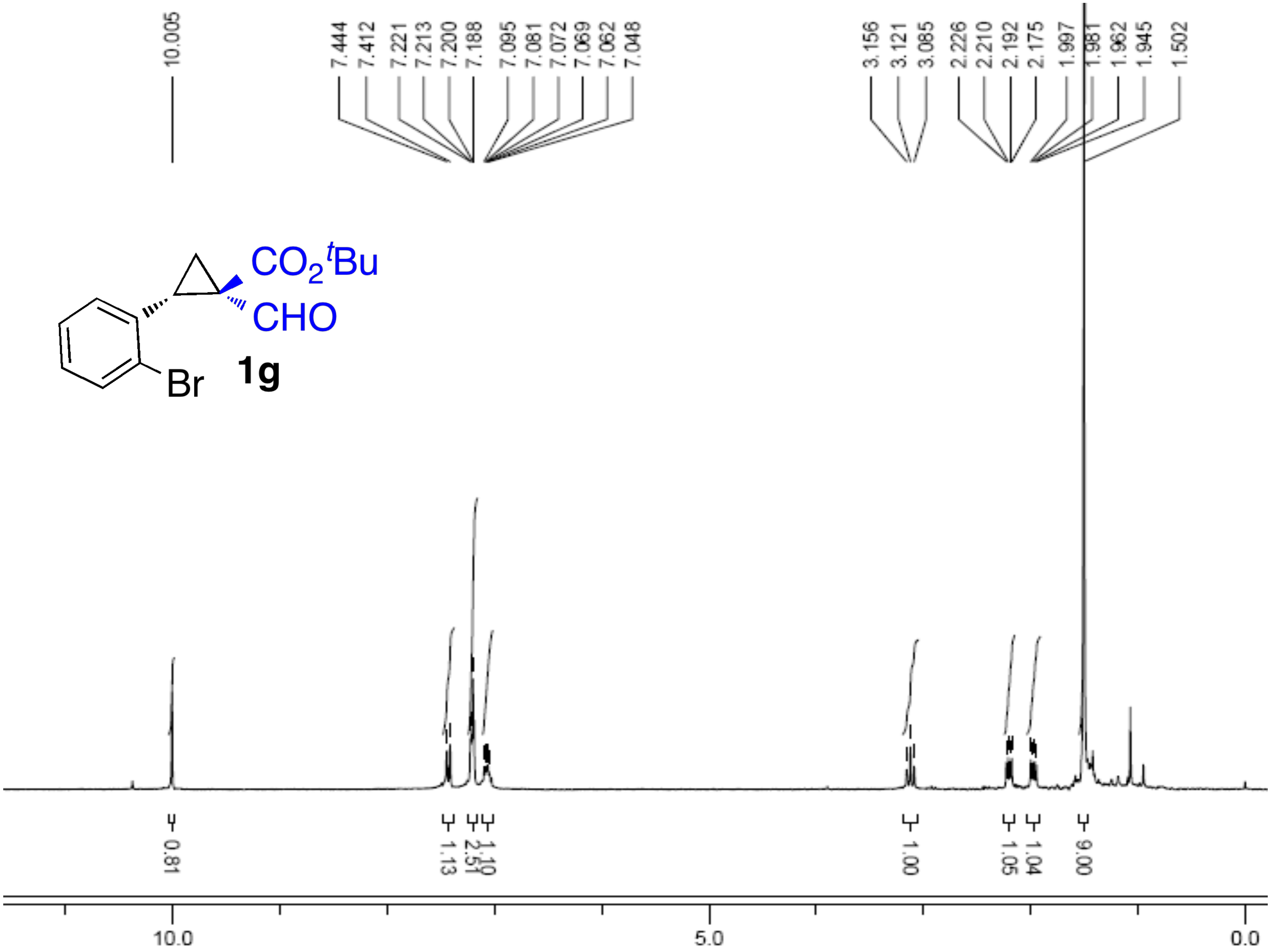
10.005
7.444
7.412
7.221
7.213
7.200
7.188
7.095
7.081
7.072
7.069
7.062
7.048
3.156
3.121
3.085
2.226
2.210
2.192
2.175
1.997
1.981
1.962
1.945
1.502
$CO_2{}^tBu$
CHO
Br
1g
0.81
1.13
2.51
1.10
1.00
1.05
1.04
9.00
10.0
5.0
0.0

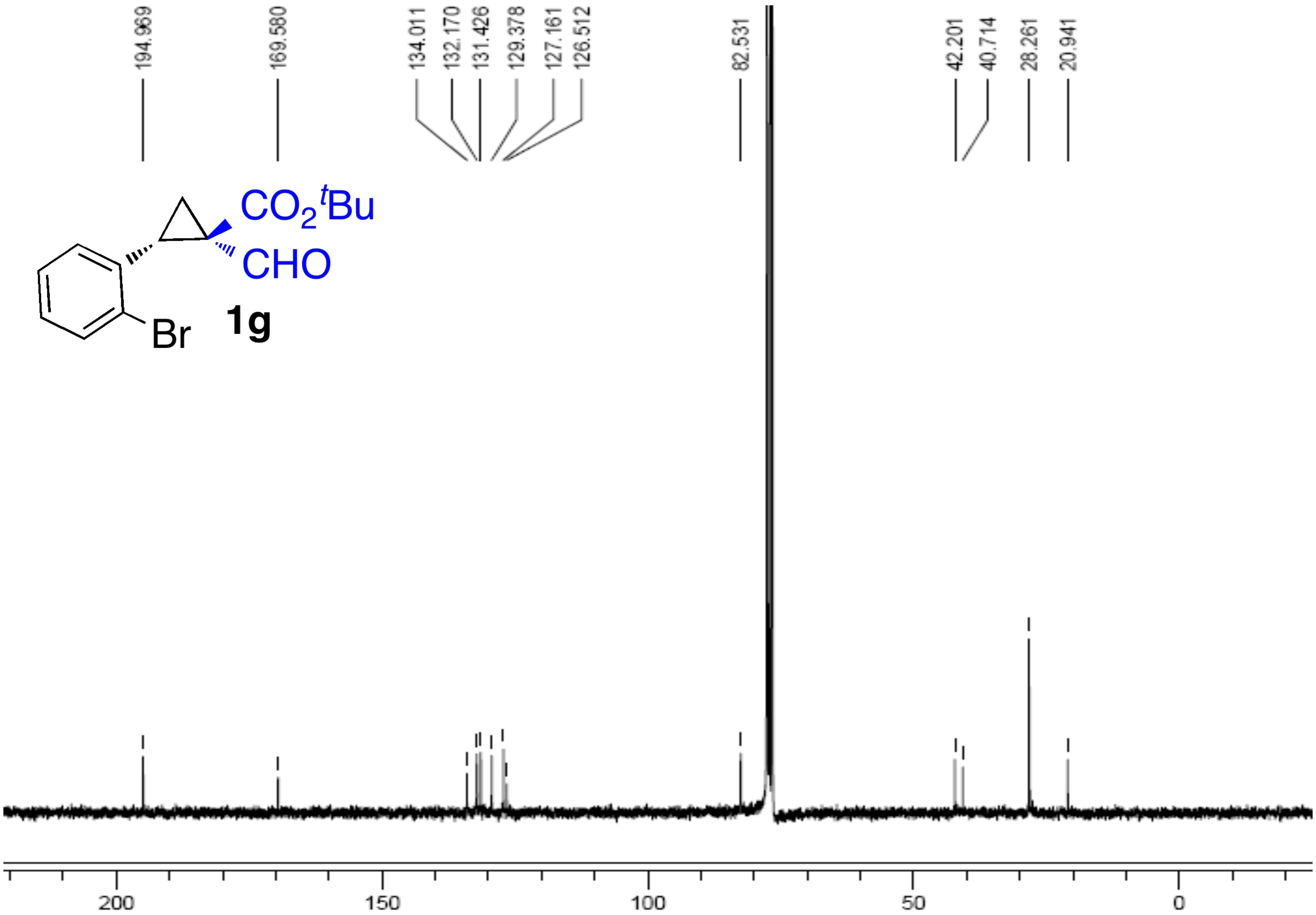

194.969
169.580
134.011
132.170
131.426
129.378
127.161
126.512
82.531
42.201
40.714
28.261
20.941
CO2tBu
CHO
Br
1g
200
150
100
50
0

xx-vi-67-3 whelk 1%@0.8 ml AGAIN
C:\EZStart\Projects\Default\Method\Shifa-EDA.met
E:\HPLC\xx-vi-67-3 whelk 1%@0.8 ml AGAIN

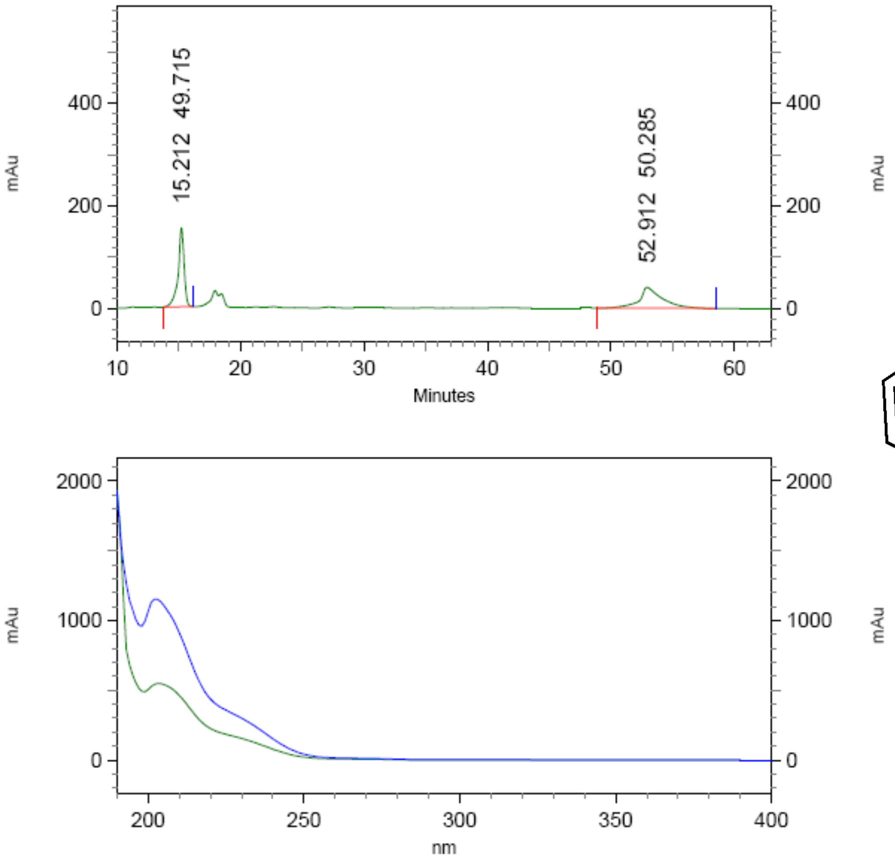

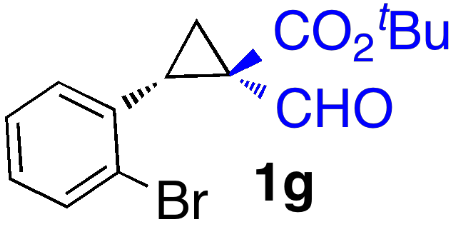

1g

2: 226 nm, 4 nm
Results

Pk #	Name	Retention Time	Area Percent
1		15.212	49.715
2		52.912	50.285
Totals			100.000

xx-vi-83 whelk 1%@0.8 ml
C:\EZStart\Projects\Default\Method\Shifa-EDA.met
E:\HPLC\xx-vi-83 whelk 1%@0.8 ml

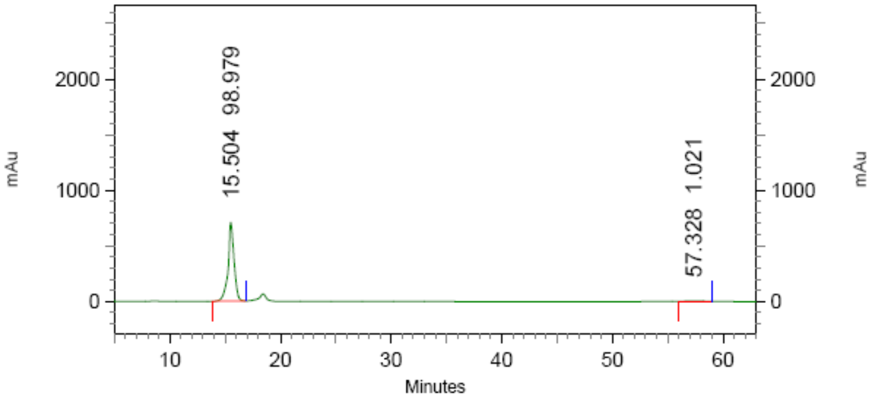

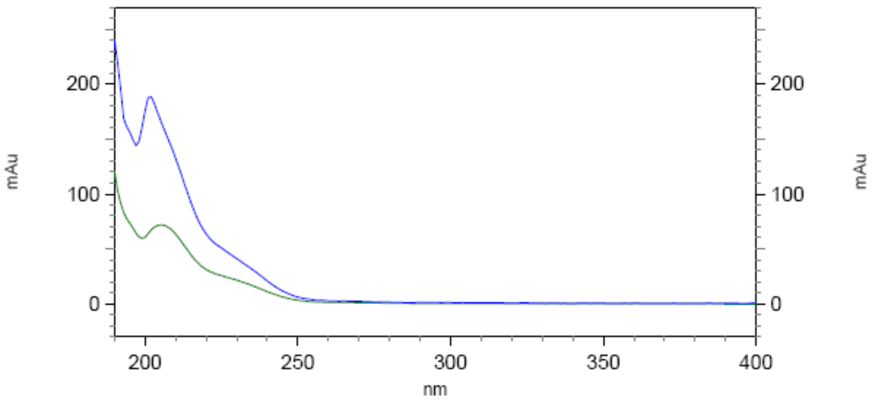

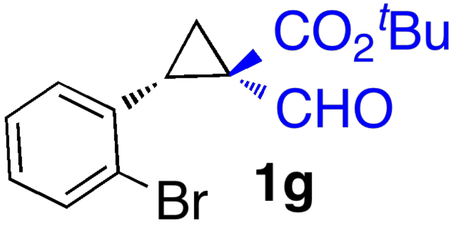

1g

2: 226 nm, 4 nm
Results

Pk #	Name	Retention Time	Area Percent
1		15.504	98.979
2		57.328	1.021
Totals			100.000

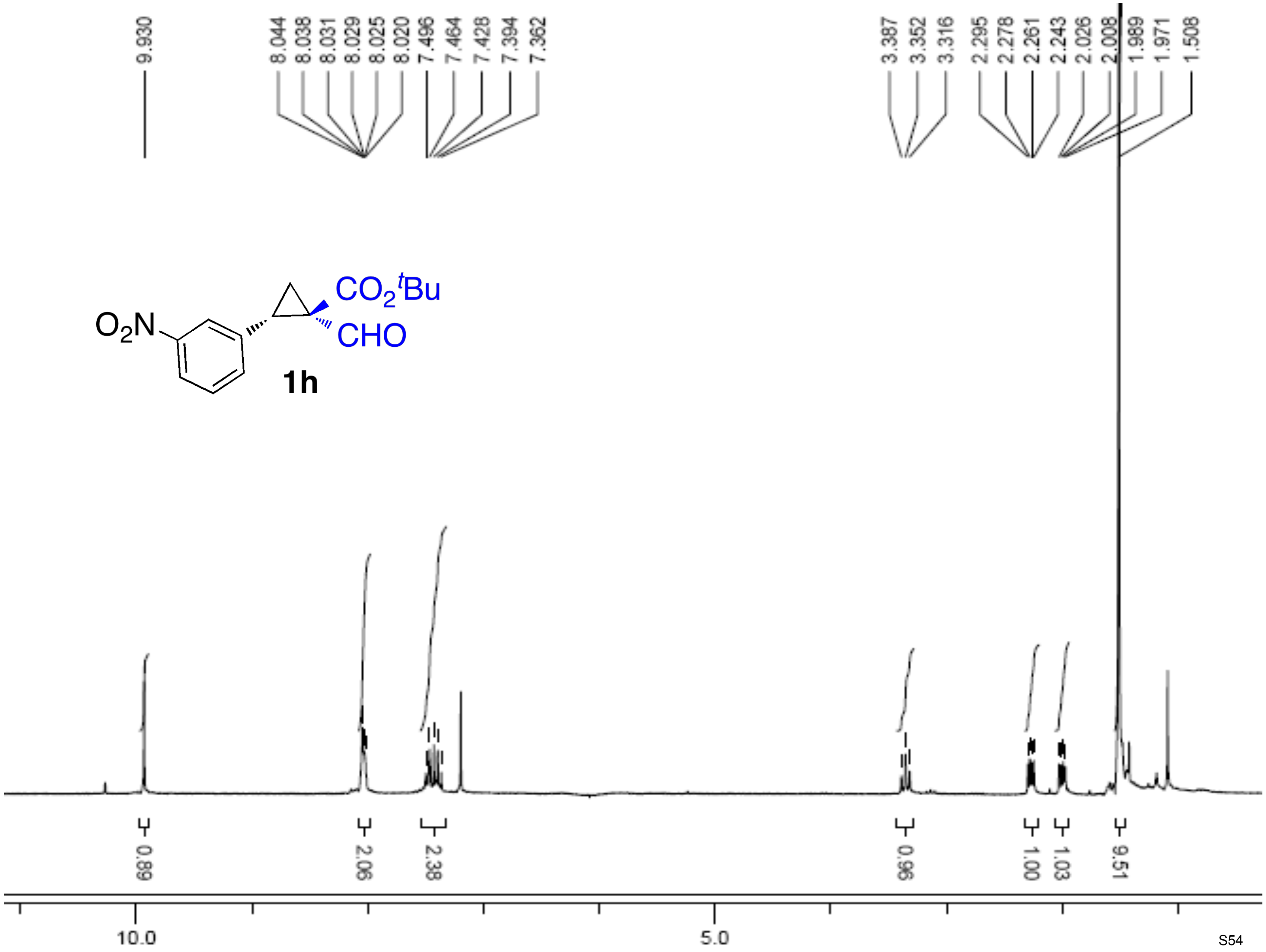
9.930
8.044 8.038 8.031 8.029 8.025 8.020 7.496 7.464 7.428 7.394 7.362
3.387 3.352 3.316 2.295 2.278 2.261 2.243 2.026 2.008 1.989 1.971 1.508
O_2N
$CO_2{}^tBu$
CHO
1h
0.89
2.06
2.38
0.96
1.00
1.03
9.51
10.0
5.0

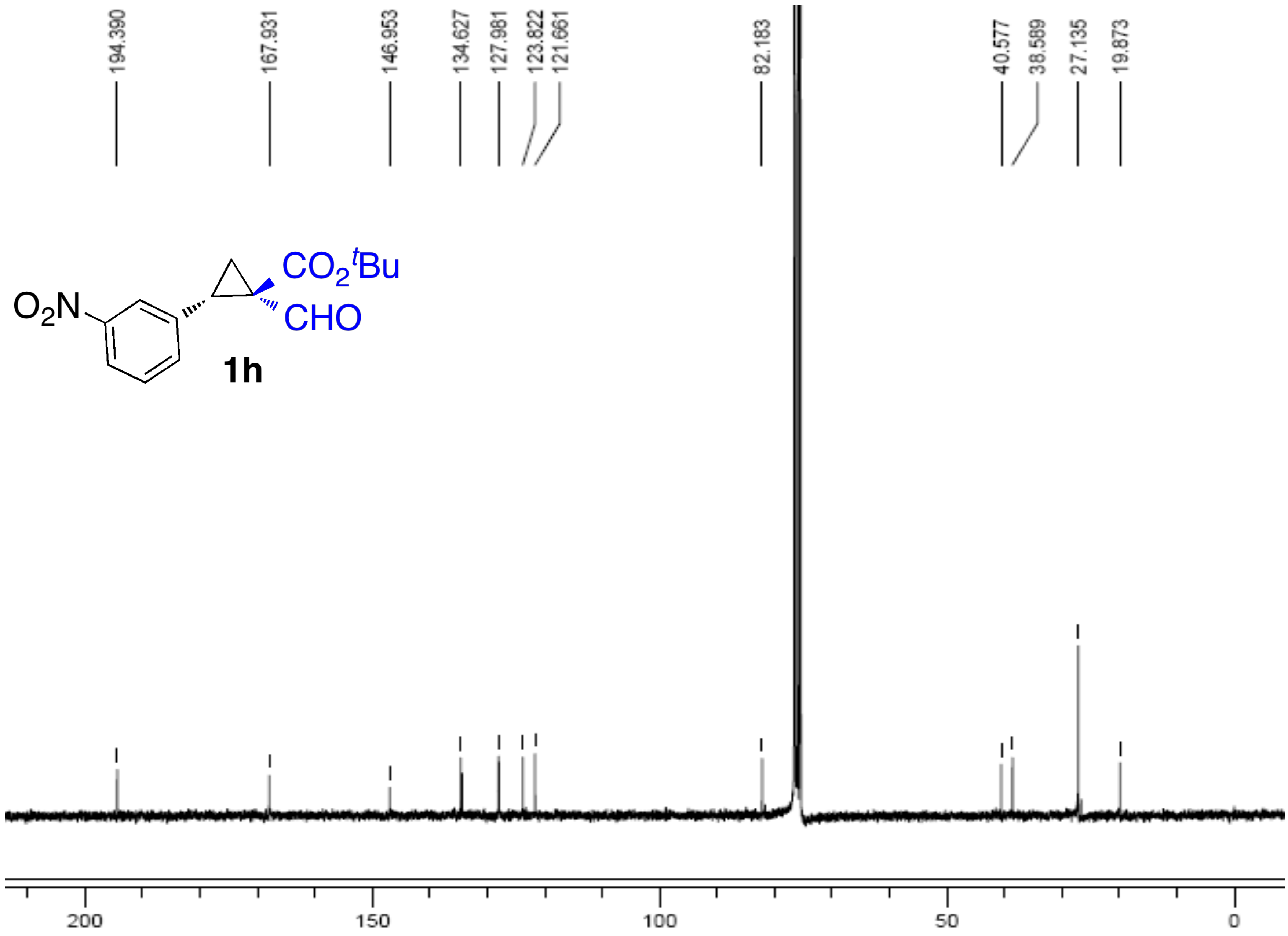
194.390
167.931
146.953
134.627
127.981
123.822
121.661
82.183
40.577
38.589
27.135
19.873
$CO_2{}^tBu$
CHO
O_2N
1h
200
150
100
50
0

xx-vi-67-1SDwhelk 2%@0.8 ml
C:\EZStart\Projects\Default\Method\Shifa-Nitro-Cyclopropane-1%.met
C:\EZStart\Projects\Default\Data\xx-vi-67-1SDwhelk 2%@0.8 ml

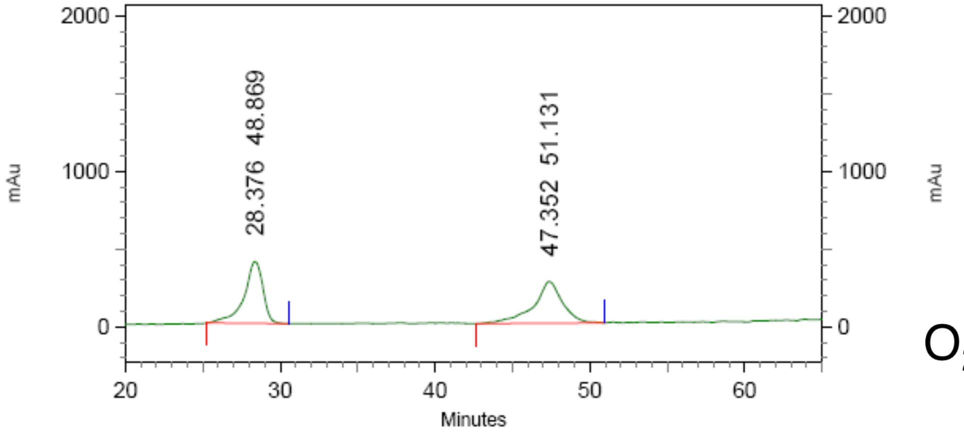

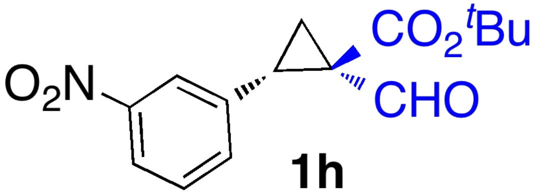

1h

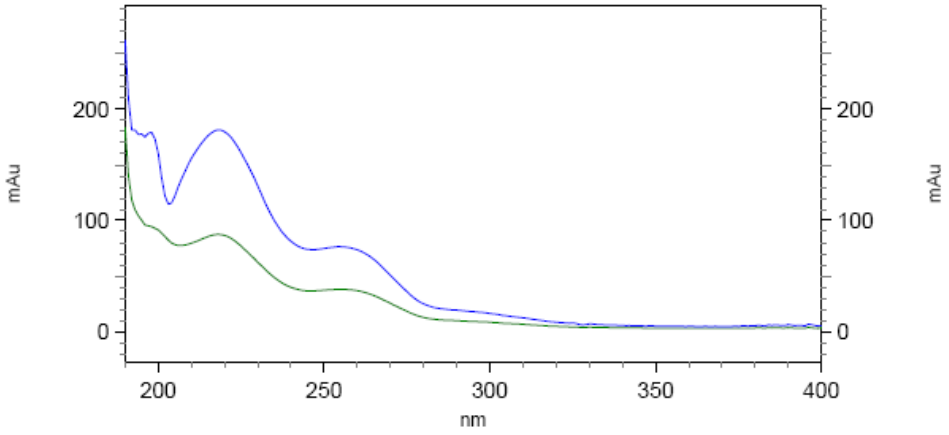

4: 221 nm, 4 nm
Results

Pk # Name	Retention Time	Area Percent
1	28.376	48.869
2	47.352	51.131
Totals		100.000

xx-vi-81 SDwhelk 2%@0.8 ml
C:\EZStart\Projects\Default\Method\Shifa-Nitro-Cyclopropane-1%.met
C:\EZStart\Projects\Default\Data\xx-vi-81 SDwhelk 2%@0.8 ml

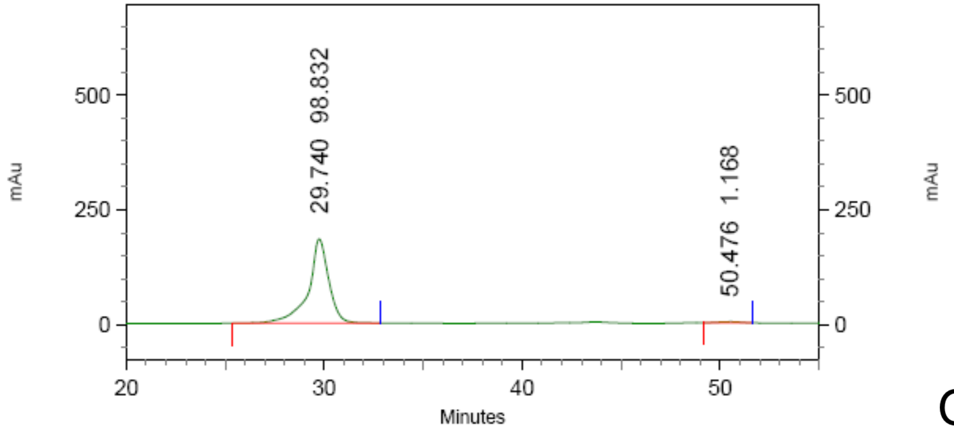

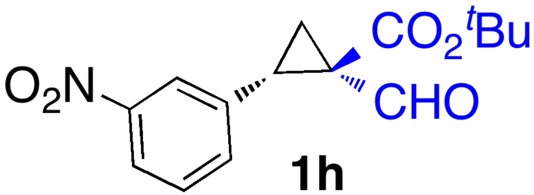

1h

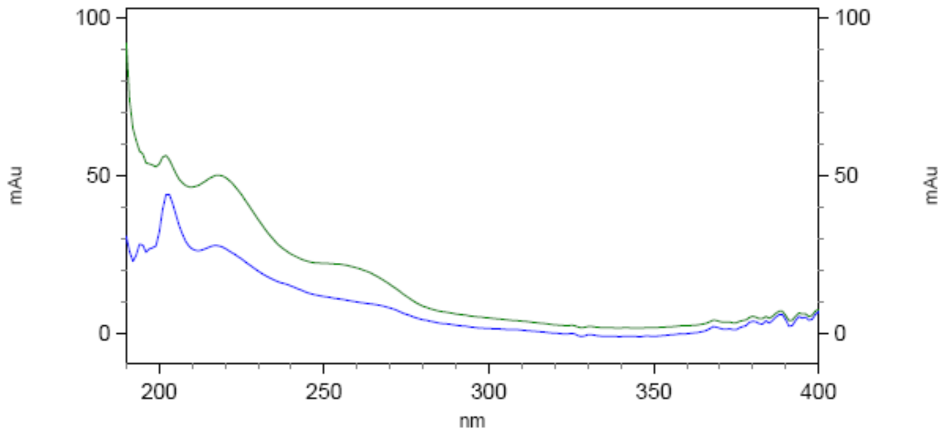

4: 231 nm, 4 nm
Results

Pk #	Name	Retention Time	Area Percent
1		29.740	98.832
2		50.476	1.168
Totals			100.000

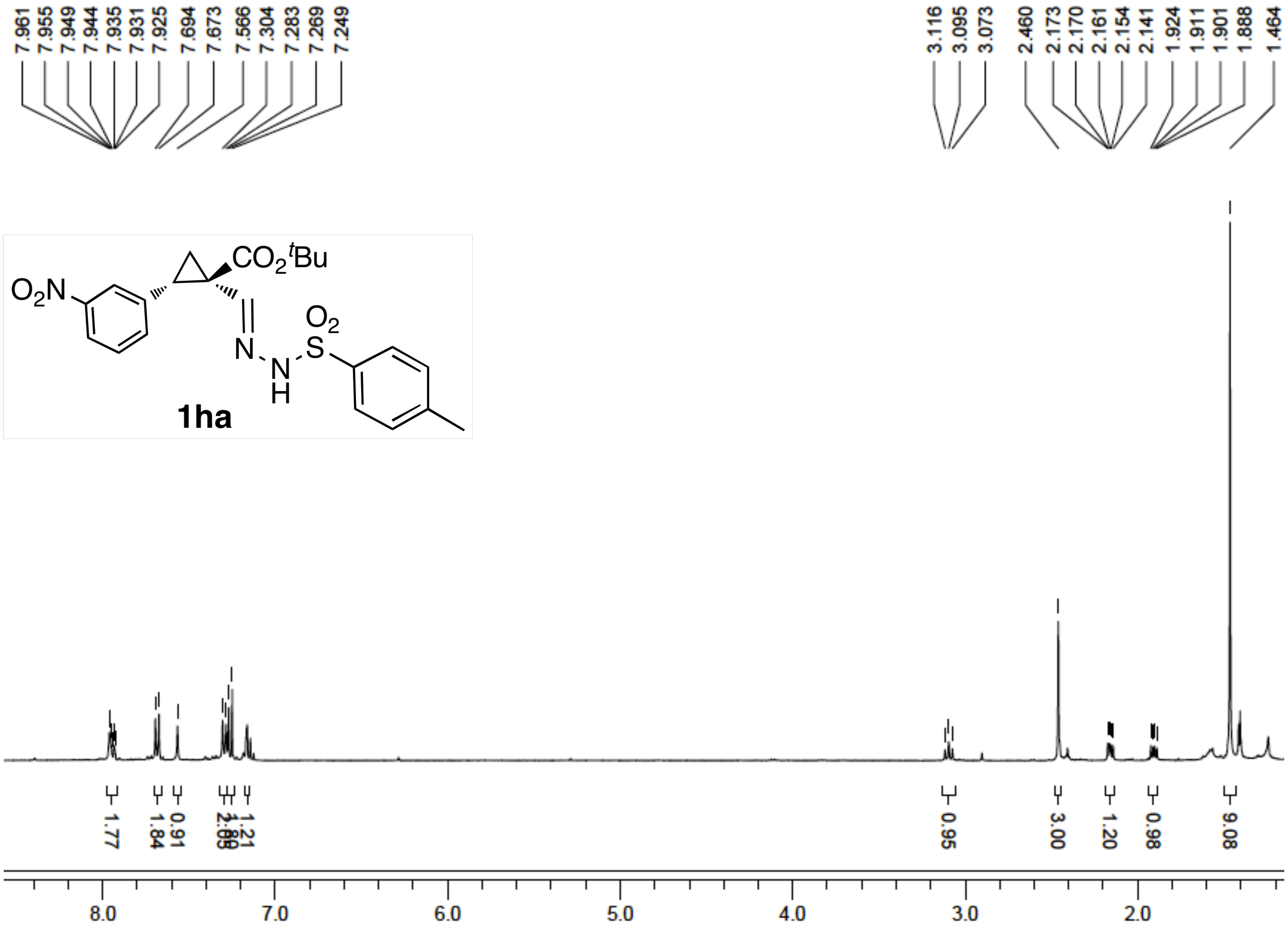
7.961
7.955
7.949
7.944
7.935
7.931
7.925
7.694
7.673
7.566
7.304
7.283
7.269
7.249
3.116
3.095
3.073
2.460
2.173
2.170
2.161
2.154
2.141
1.924
1.911
1.901
1.888
1.464
1.77
1.84
0.91
1.21
0.95
3.00
1.20
0.98
9.08
8.0
7.0
6.0
5.0
4.0
3.0
2.0

1ha

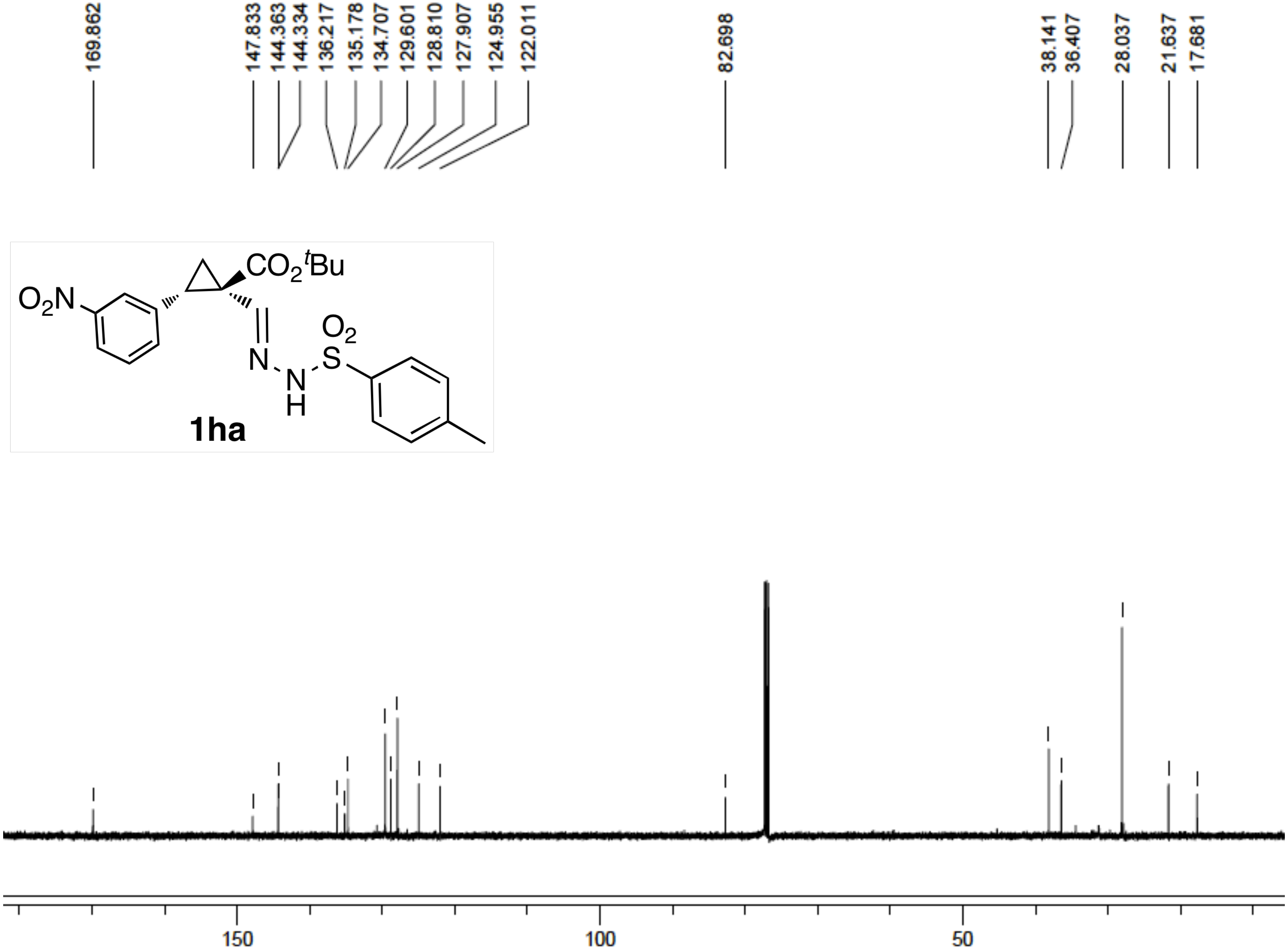
169.862
147.833
144.363
144.334
136.217
135.178
134.707
129.601
128.810
127.907
124.955
122.011
82.698
38.141
36.407
28.037
21.637
17.681
CO2tBu
O2N
O2
S
N
N
H
1ha
150
100
50

XC-IX-84-10%-WHELK1ml
C:\EZStart\Projects\Default\Data\XC-IX-84-10%-WHELK1ml
C:\EZStart\Projects\Default\Method\XC-Whelk10%1ml.met

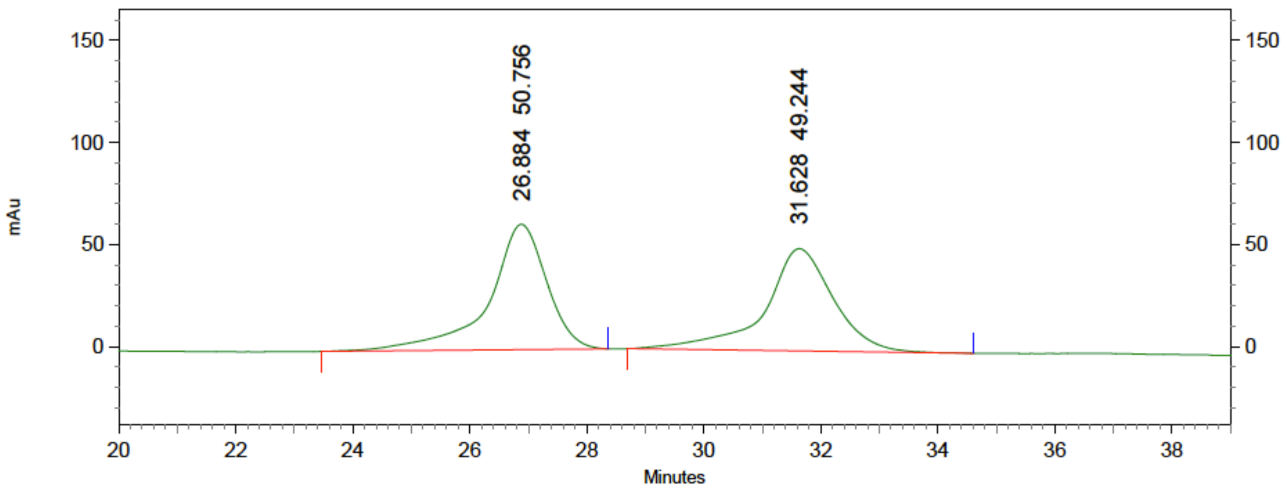

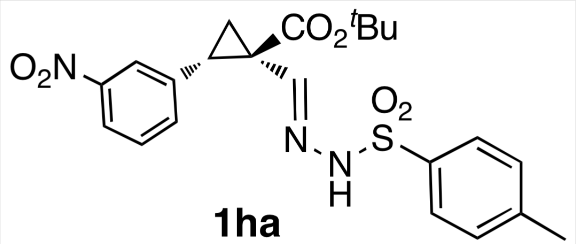

1ha

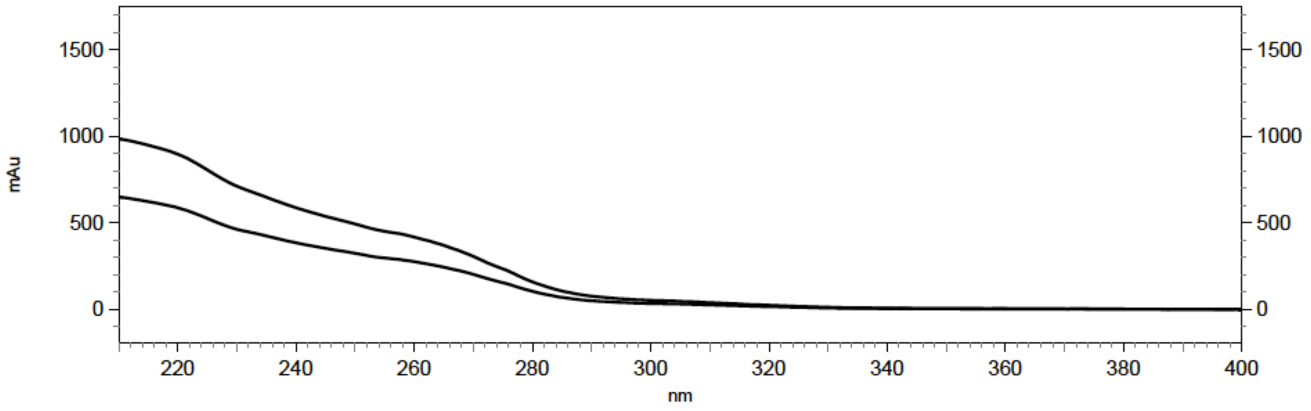

8: 230 nm, 4 nm Results

Pk #	Retention Time	Area Percent
1	26.884	50.756
2	31.628	49.244
Totals		100.000

XC-IX-69-10%-WHELK1ml
C:\EZStart\Projects\Default\Data\XC-IX-69-10%-WHELK1ml
C:\EZStart\Projects\Default\Method\XC-Whelk10%1ml.met

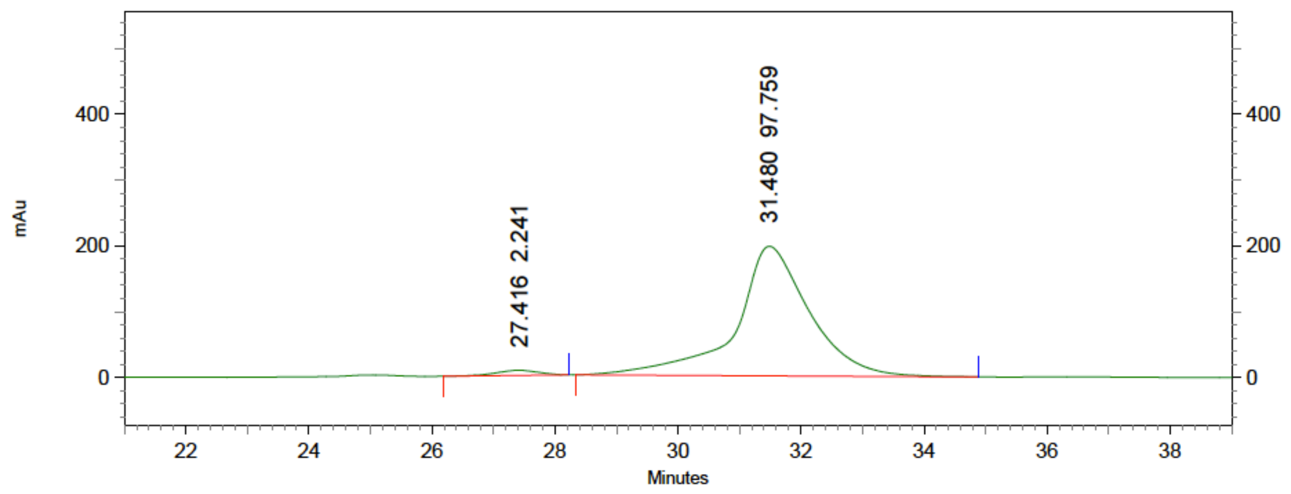

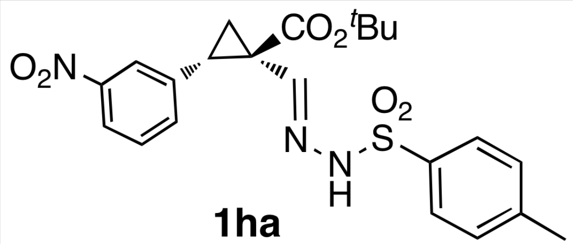

1ha

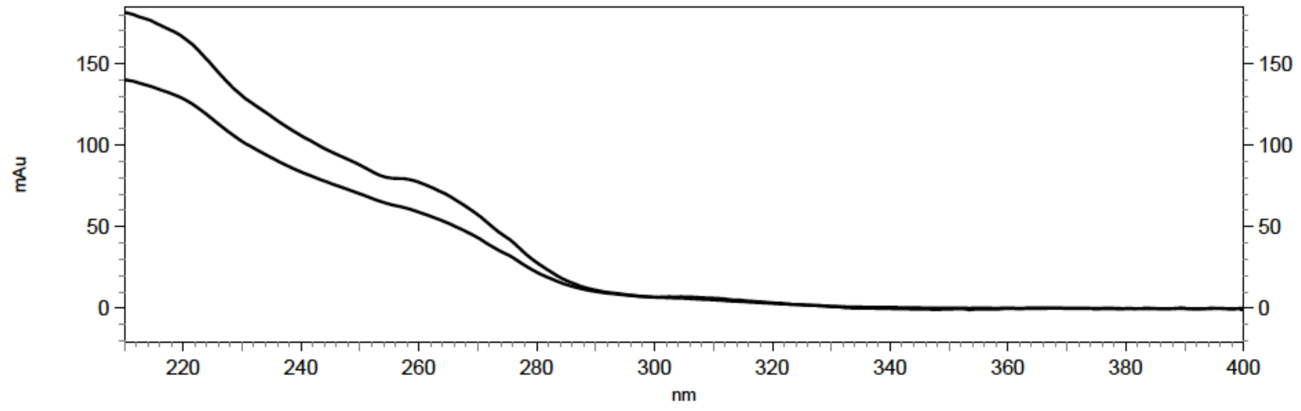

8: 239 nm, 4 nm Results

Pk #	Retention Time	Area Percent
1	27.416	2.241
2	31.480	97.759
Totals		100.000

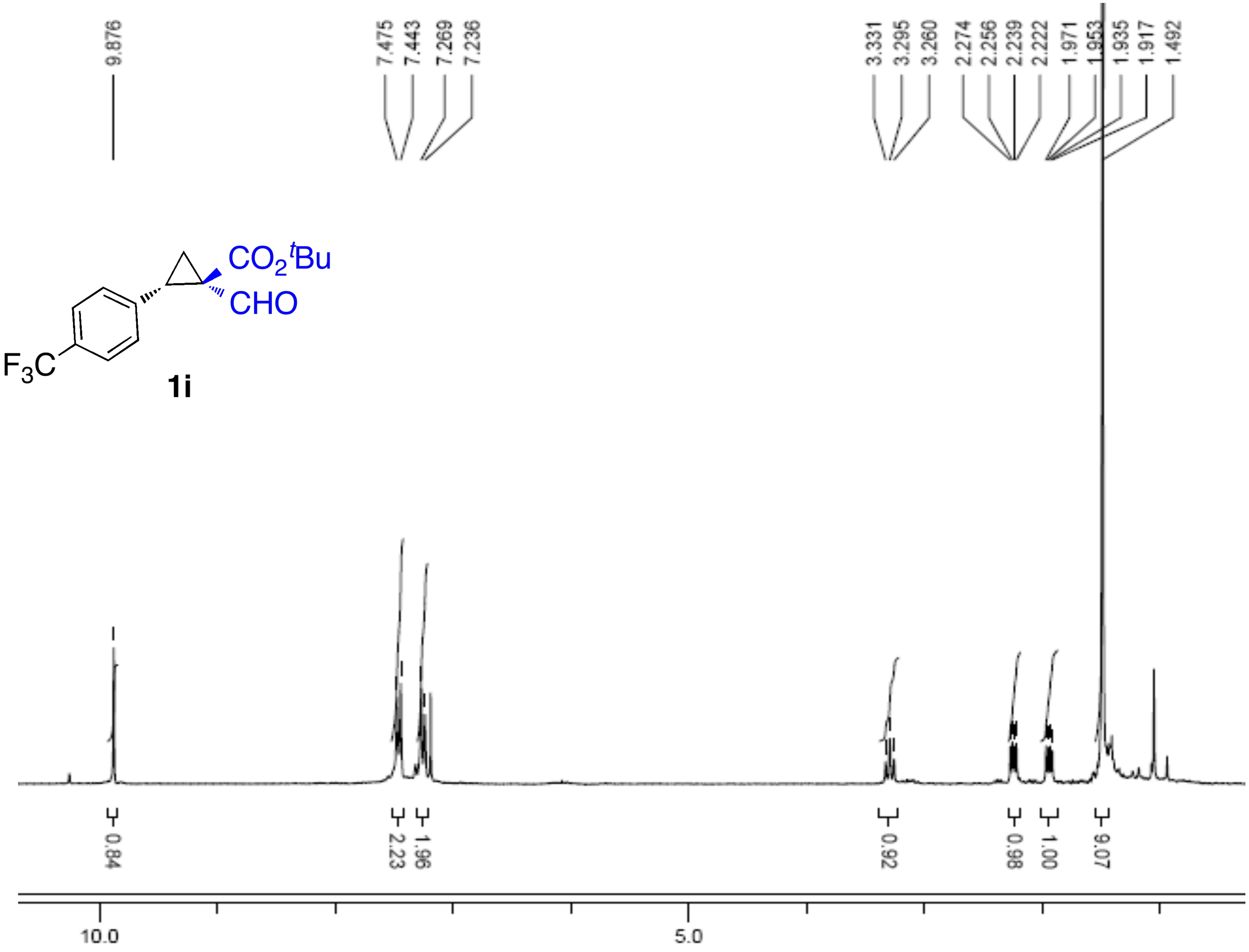
9.876
7.475
7.443
7.269
7.236
3.331
3.295
3.260
2.274
2.256
2.239
2.222
1.971
1.953
1.935
1.917
1.492
CO2tBu
CHO
F3C
0.84
2.23
1.96
0.92
0.98
1.00
9.07
10.0
5.0

1i

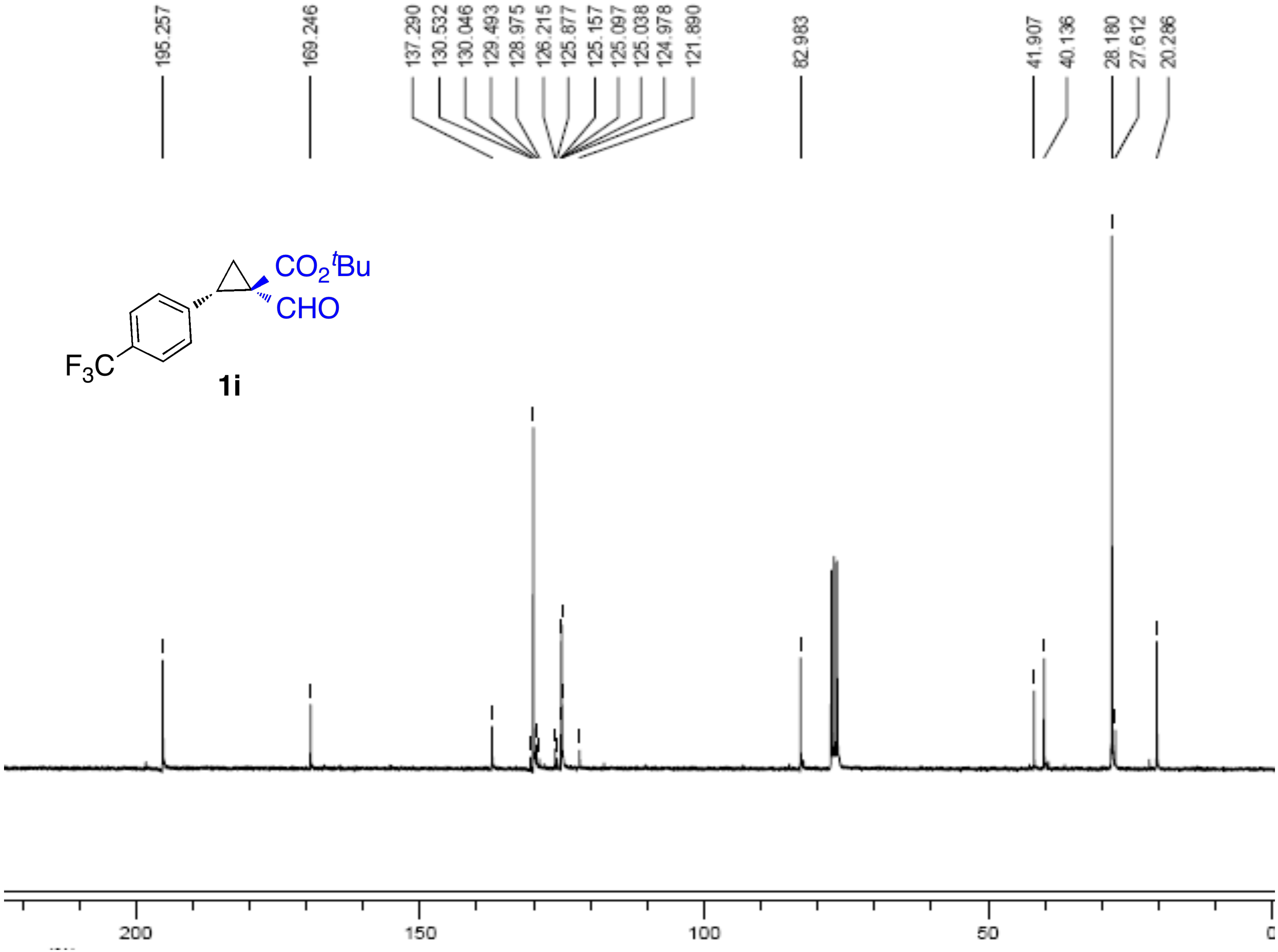
195.257
169.246
137.290
130.532
130.046
129.493
128.975
126.215
125.877
125.157
125.097
125.038
124.978
121.890
82.983
41.907
40.136
28.180
27.612
20.286
$CO_2{}^tBu$
CHO
F_3C
1i
200
150
100
50
0

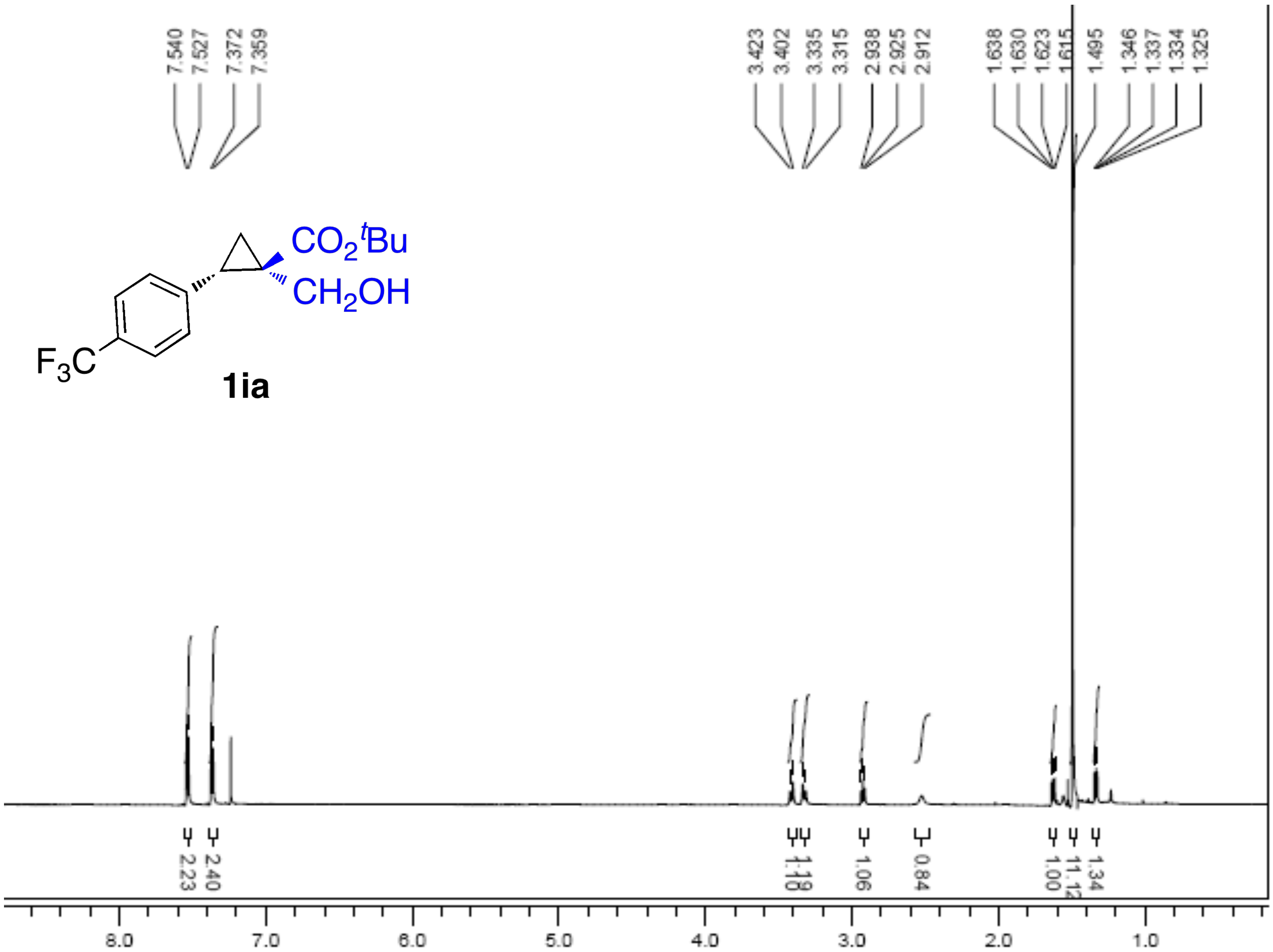
7.540
7.527
7.372
7.359
3.423
3.402
3.335
3.315
2.938
2.925
2.912
1.638
1.630
1.623
1.615
1.495
1.346
1.337
1.334
1.325
CO2tBu
CH2OH
F3C
1ia
2.23
2.40
1.10
1.19
1.06
0.84
1.00
11.12
1.34
8.0
7.0
6.0
5.0
4.0
3.0
2.0
1.0

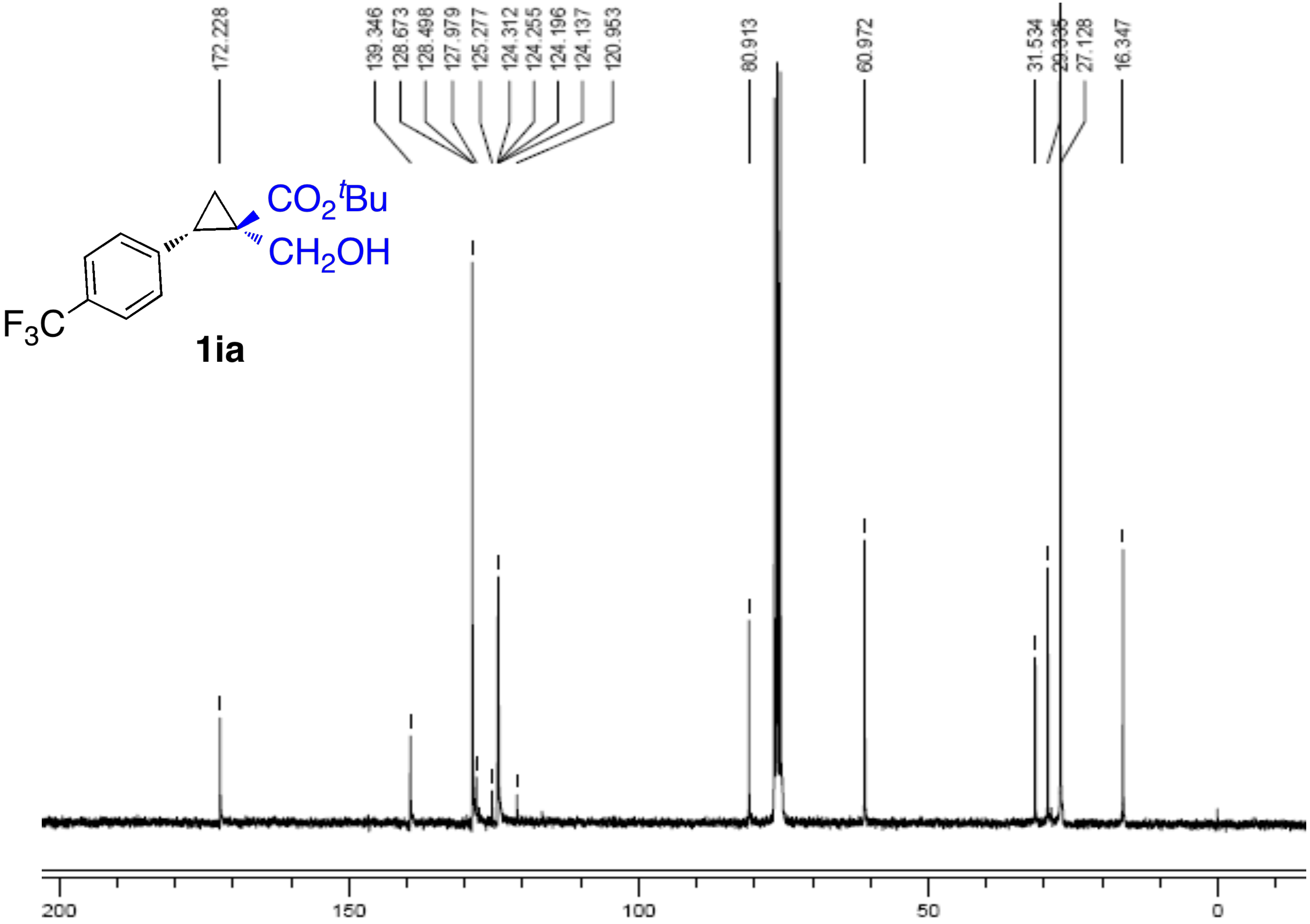

172.228
139.346
128.673
128.498
127.979
125.277
124.312
124.255
124.196
124.137
120.953
80.913
60.972
31.534
29.335
27.128
16.347
$CO_2{}^tBu$
CH_2OH
F_3C
1ia
200
150
100
50
0

xx-vii-294 ADH 1%@0.8 ml
C:\EZStart\Projects\Default\Method\JRT-WK-1%1ML90MIN.met
C:\EZStart\Projects\Default\Data\xx-vii-294 ADH 1%@0.8 ml

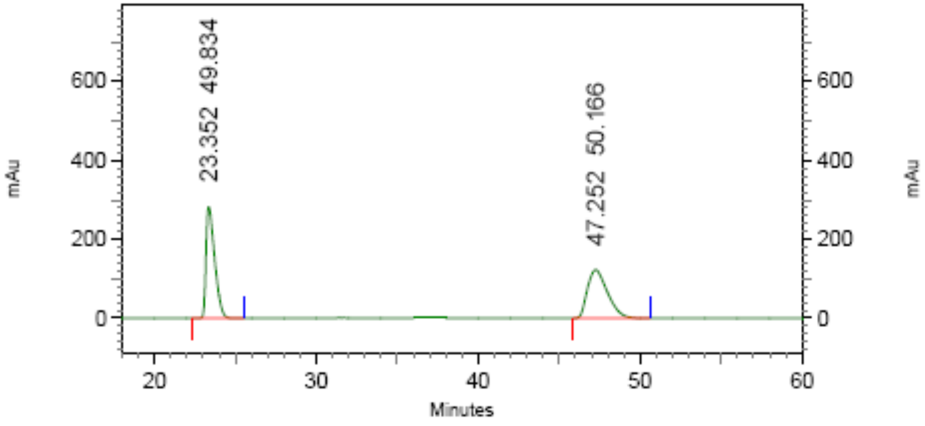

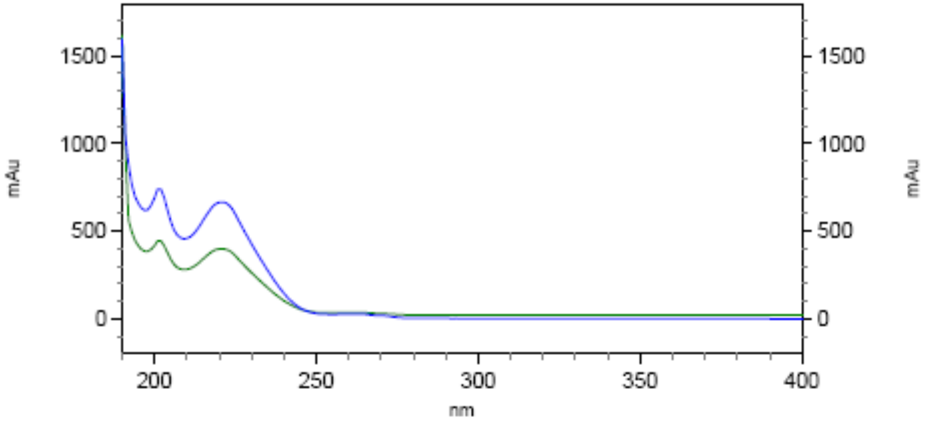

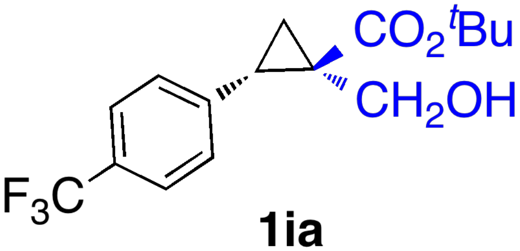

1ia

3: 227 nm, 4 nm
Results

Pk #	Name	Retention Time	Area Percent
1		23.352	49.834
2		47.252	50.166
Totals			100.000

xx-vii-300 ADH 1%@0.8 ml
C:\EZStart\Projects\Default\Method\Shifa-P-Cyclopropane 1%Whelk.met
C:\EZStart\Projects\Default\Data\xx-vii-300 ADH 1%@0.8 ml

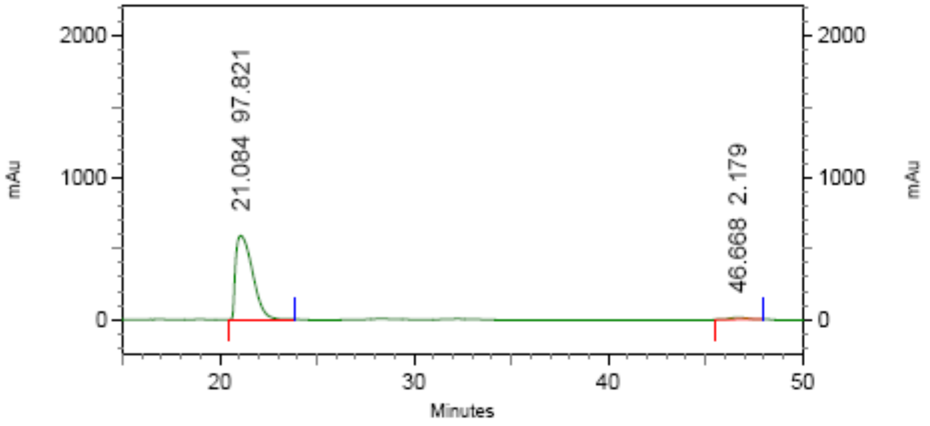

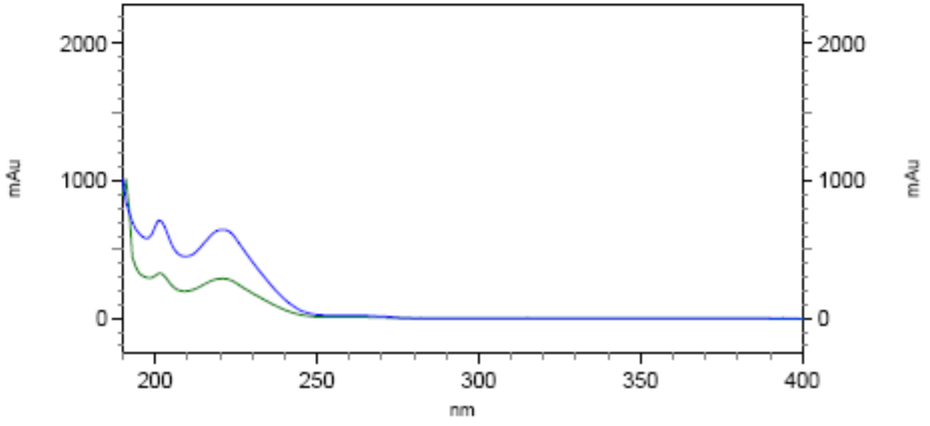

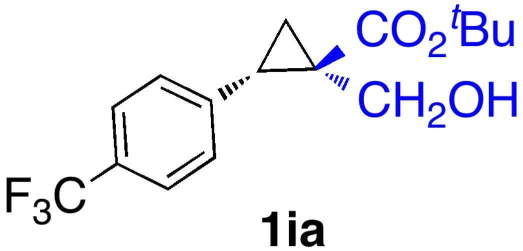

1ia

2: 225 nm, 4 nm

Results

Pk #	Name	Retention Time	Area Percent
1		21.084	97.821
2		46.668	2.179
Totals			100.000

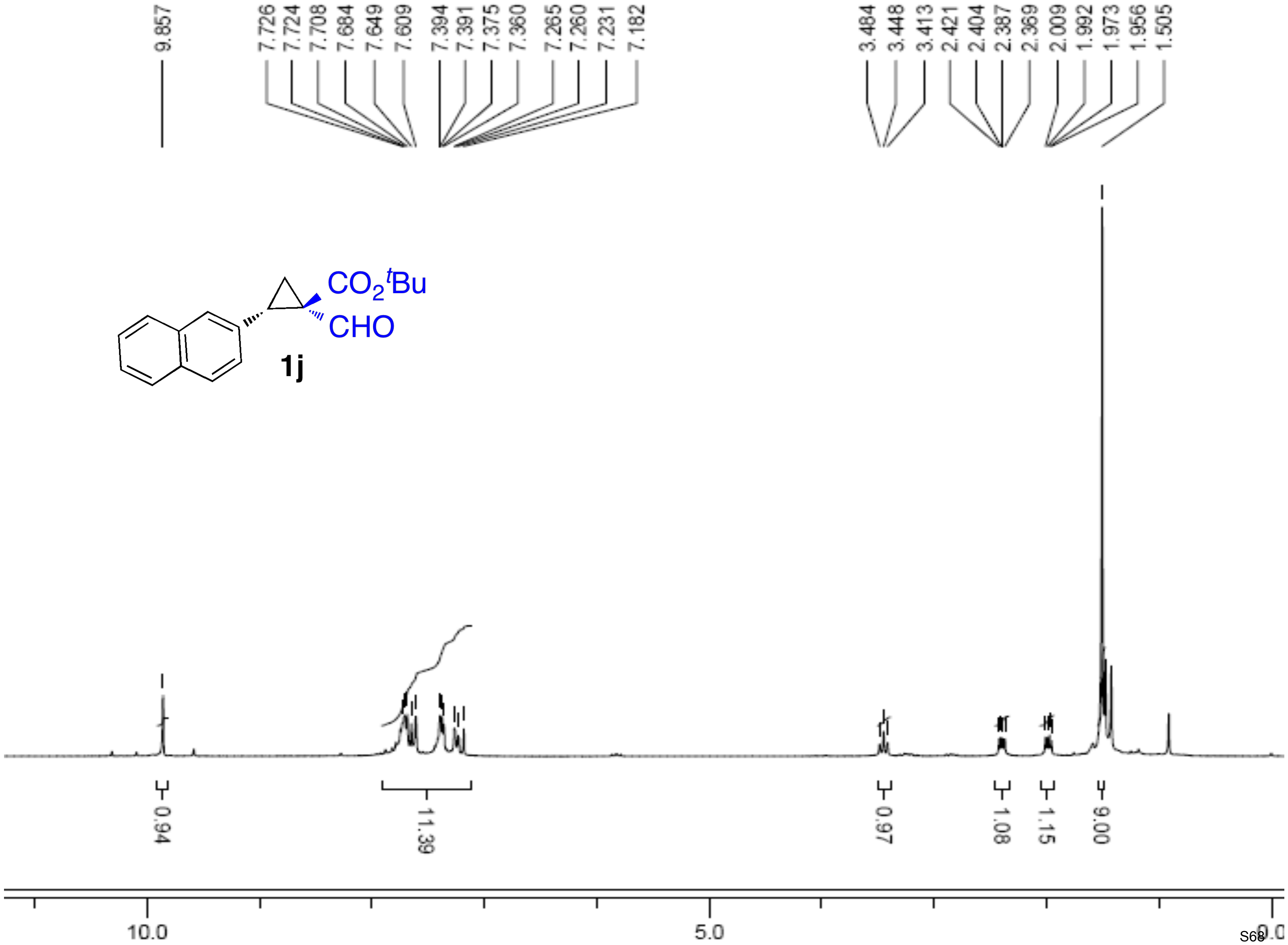

9.857
7.726
7.724
7.708
7.684
7.649
7.609
7.394
7.391
7.375
7.360
7.265
7.260
7.231
7.182
3.484
3.448
3.413
2.421
2.404
2.387
2.369
2.009
1.992
1.973
1.956
1.505
$CO_2{}^tBu$
CHO
1j
0.94
11.39
0.97
1.08
1.15
9.00
10.0
5.0
0.0

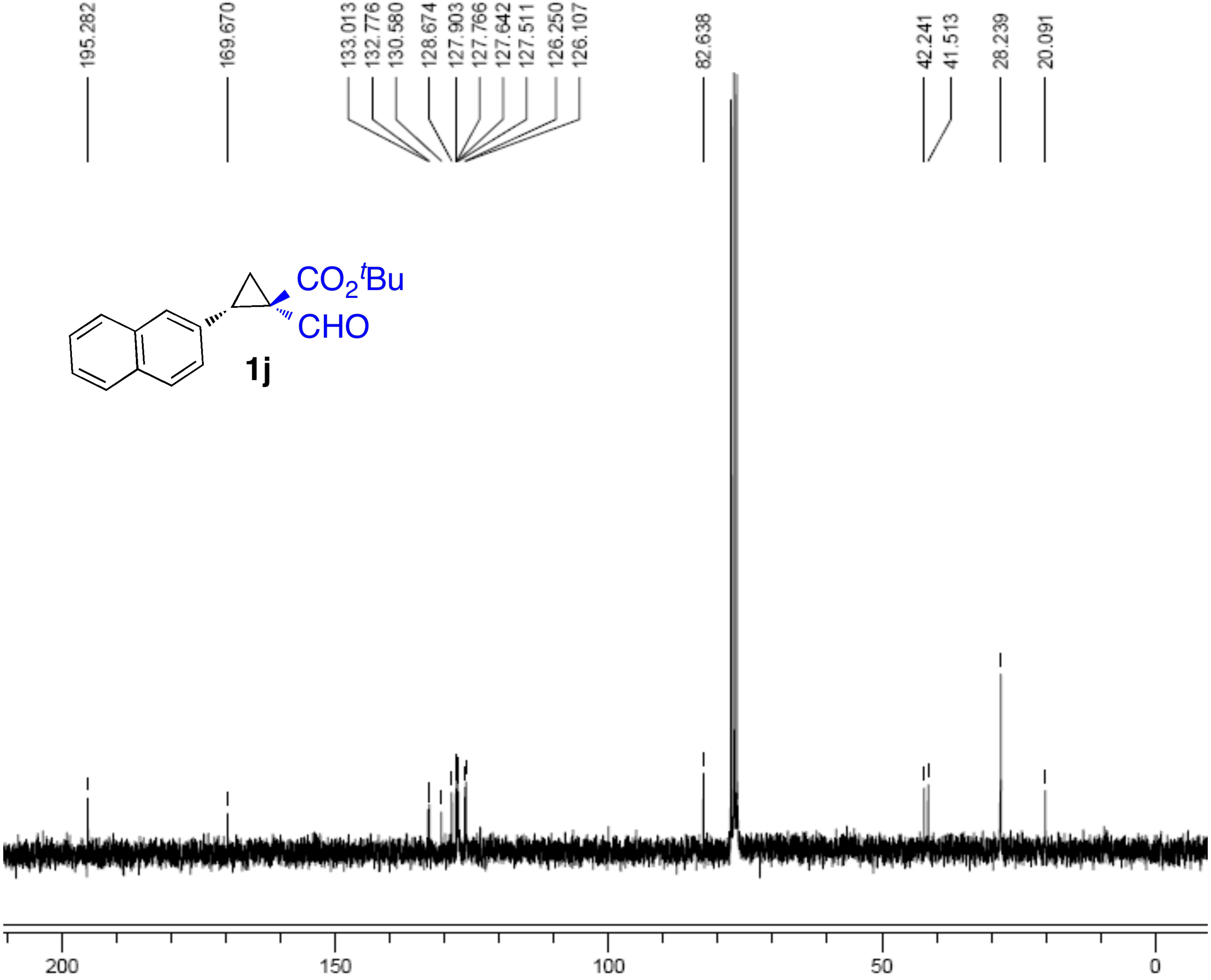
195.282
169.670
133.013
132.776
130.580
128.674
127.903
127.766
127.642
127.511
126.250
126.107
82.638
42.241
41.513
28.239
20.091
200
150
100
50
0
$CO_2{}^tBu$
CHO

1j

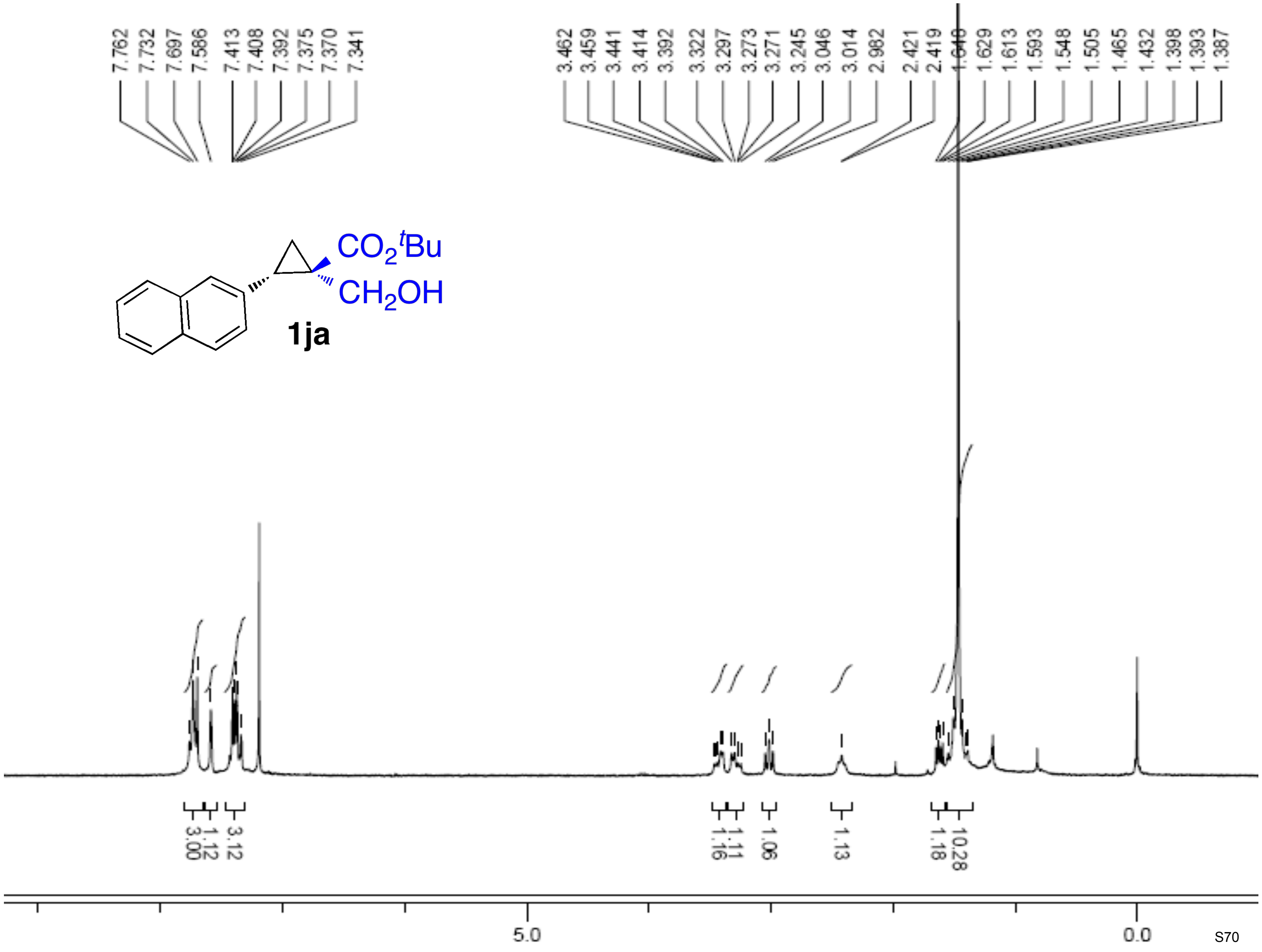
7.762
7.732
7.697
7.586
7.413
7.408
7.392
7.375
7.370
7.341
3.462
3.459
3.441
3.414
3.392
3.322
3.297
3.273
3.271
3.245
3.046
3.014
2.982
2.421
2.419
1.629
1.613
1.593
1.548
1.505
1.465
1.432
1.398
1.393
1.387
$CO_2{}^tBu$
CH_2OH
1ja
3.00
1.12
3.12
1.16
1.11
1.06
1.13
1.18
10.28
5.0
0.0

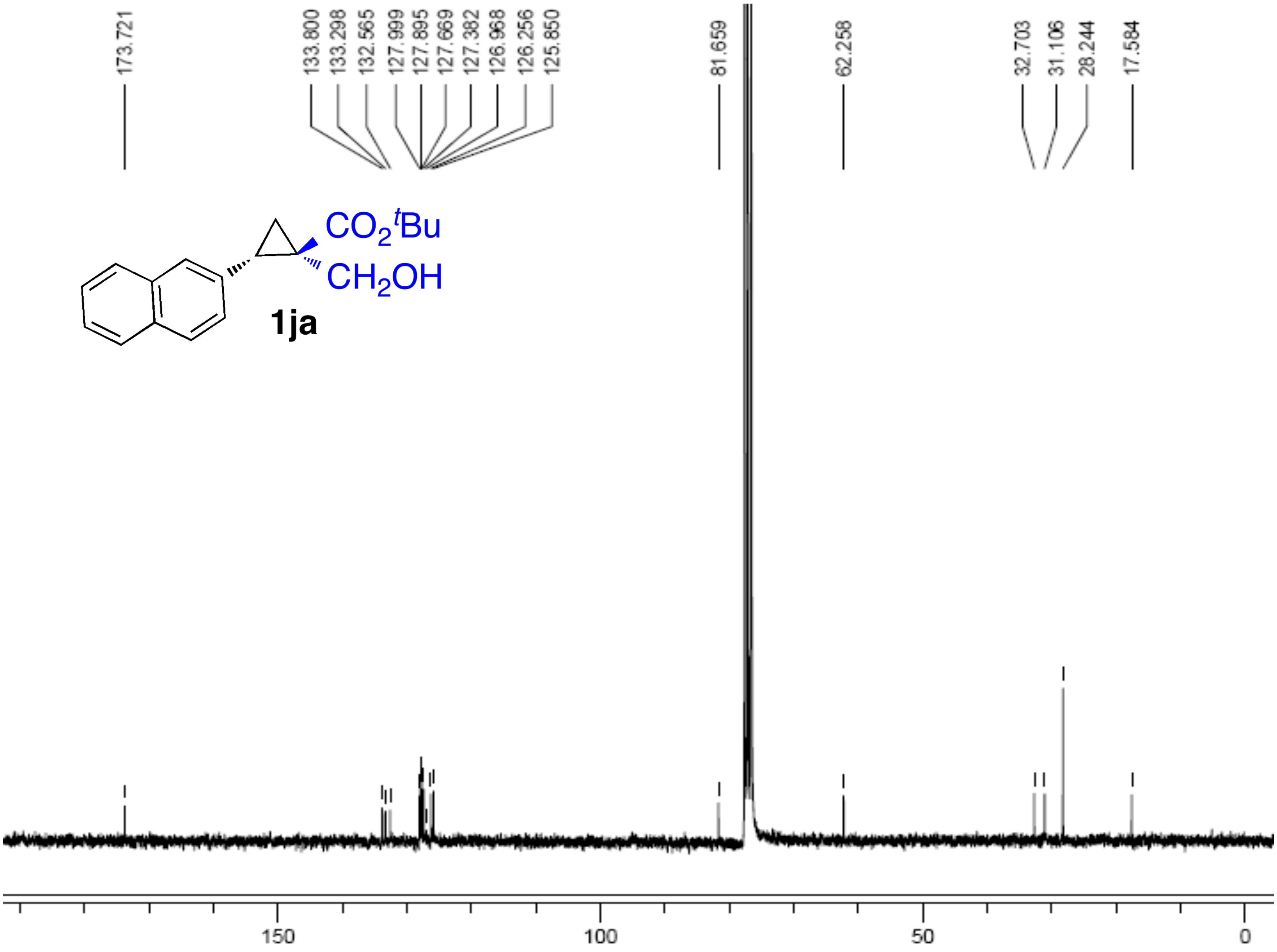

173.721
133.800
133.298
132.565
127.999
127.895
127.669
127.382
126.968
126.256
125.850
81.659
62.258
32.703
31.106
28.244
17.584
CO2tBu
CH2OH
1ja
150
100
50
0

xx-vi-137 ODH 3%@0.8 ml
C:\EZStart\Projects\Default\Method\Shifa-EDA.met
C:\EZStart\Projects\Default\Data\xx-vi-137 ODH 3%@0.8 ml

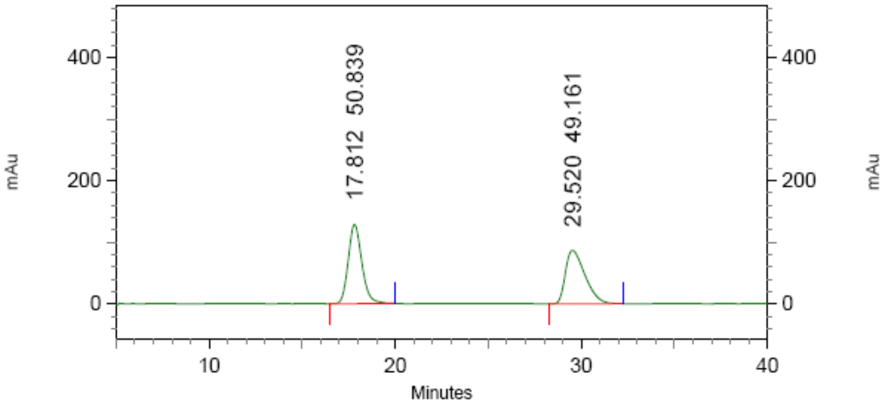

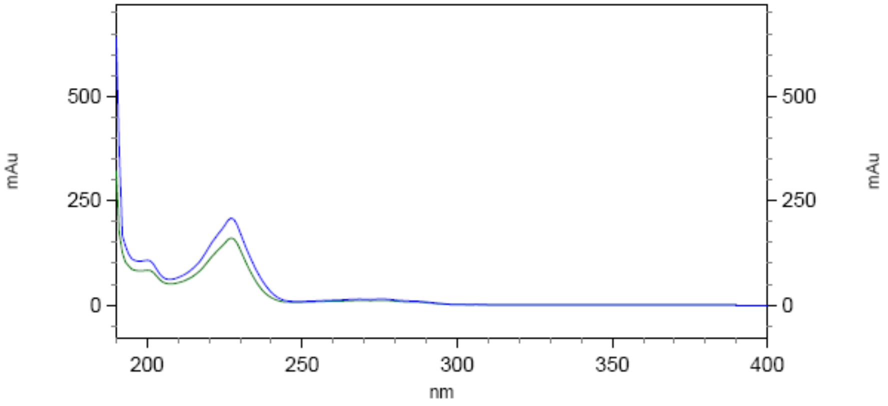

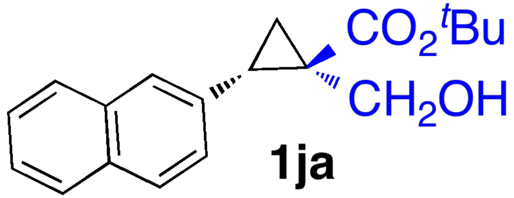

1ja

2: 254 nm, 4 nm
Results

Pk # Name	Retention Time	Area Percent
1	17.812	50.839
2	29.520	49.161
Totals		100.000

xx-vi-139 ODH 3%@0.8 ml
C:\EZStart\Projects\Default\Method\Shifa-EDA.met
C:\EZStart\Projects\Default\Data\xx-vi-139 ODH 3%@0.8 ml

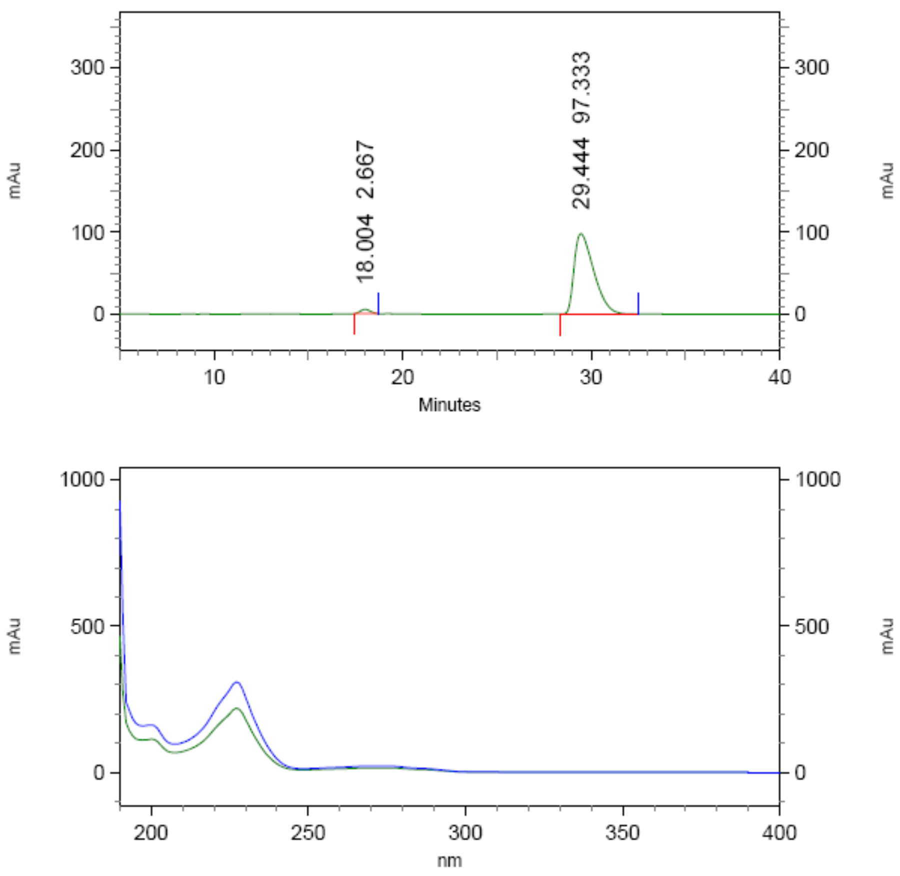

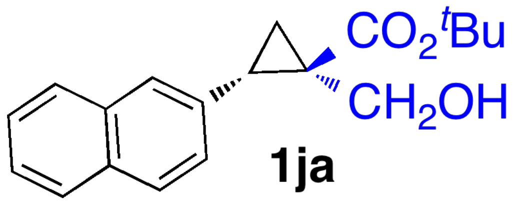

1ja

2: 254 nm, 4 nm
Results

Pk # Name	Retention Time	Area Percent
1	18.004	2.667
2	29.444	97.333
Totals		100.000

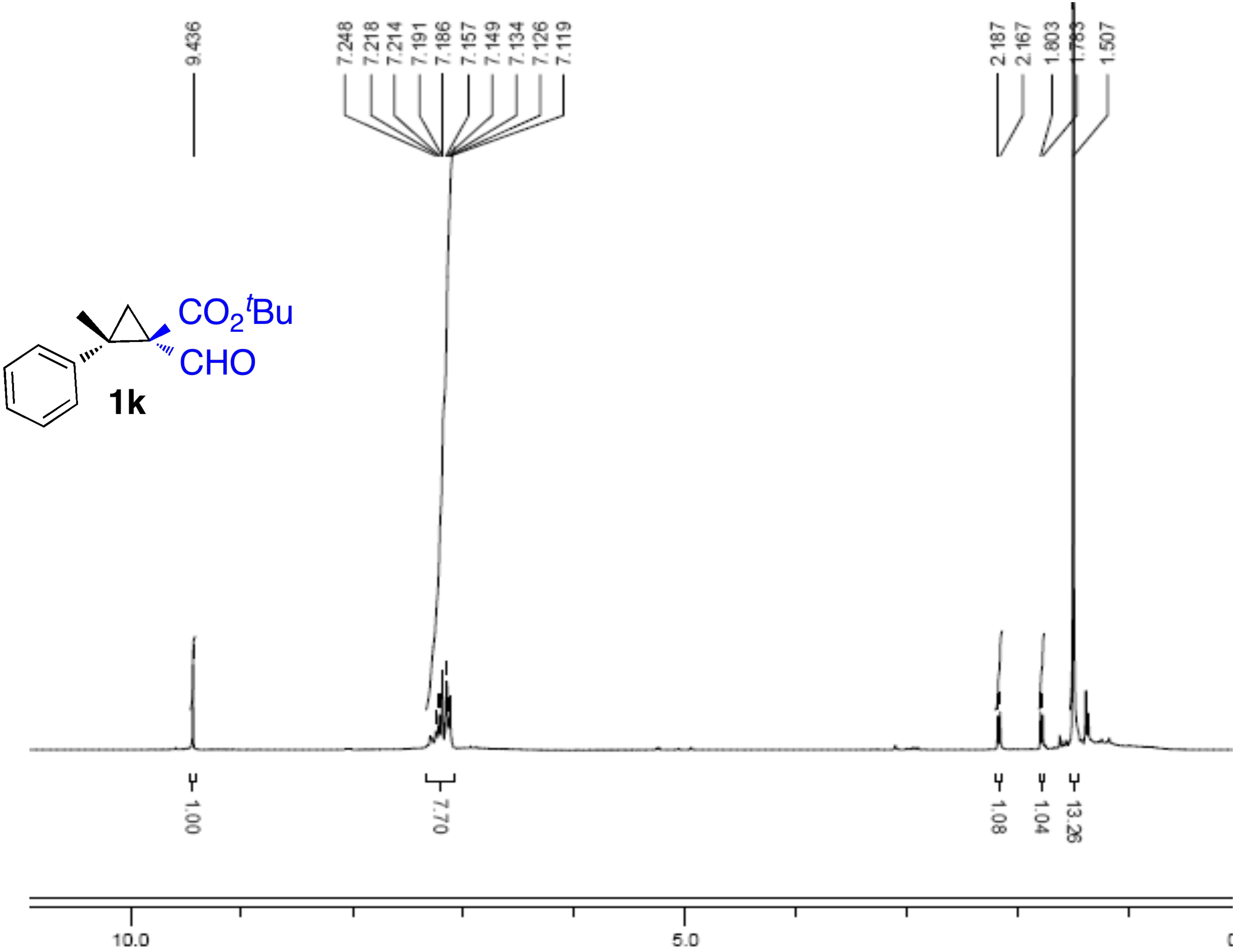
9.436
7.248
7.218
7.214
7.191
7.186
7.157
7.149
7.134
7.126
7.119
2.187
2.167
1.803
1.783
1.507
CO2tBu
CHO
1k
1.00
7.70
1.08
1.04
13.26
10.0
5.0

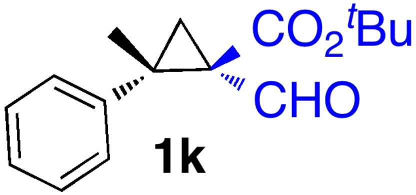
CO2tBu
CHO

1k

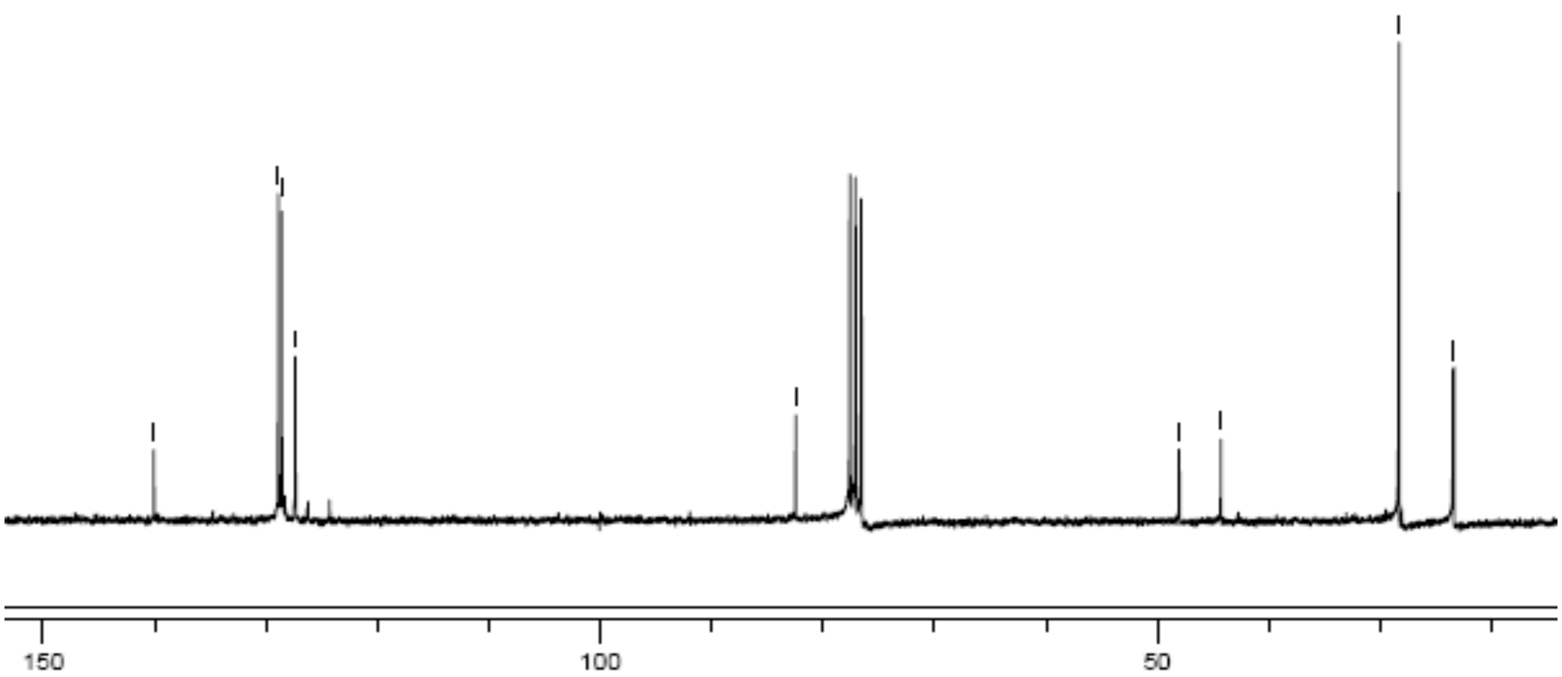
140.049
128.894
128.577
127.359
82.455
48.041
44.286
28.305
23.461
23.371
150
100
50

```
xx-vii-298 whelk 2%@0.8 ml
C:\EZStart\Projects\Default\Method\Shifa 4-t-Butylbenzene (OD-H 10-90).met
C:\EZStart\Projects\Default\Data\xx-vii-298 whelk 2%@0.8 ml
```

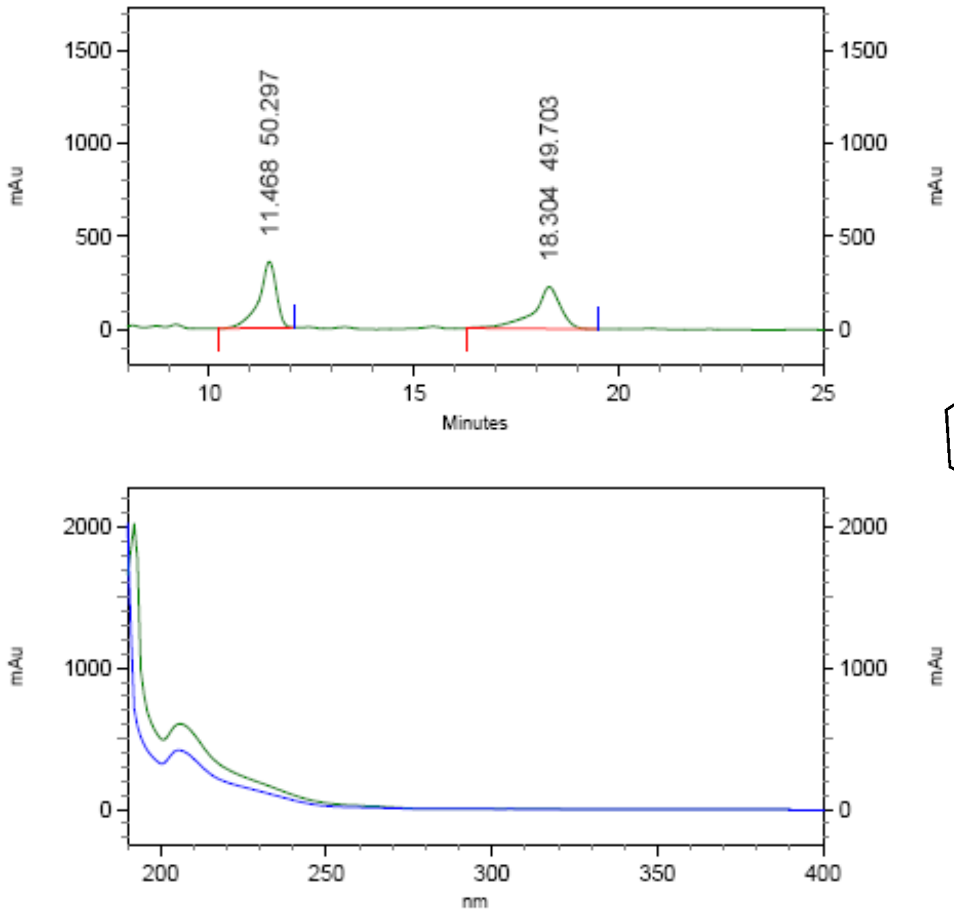

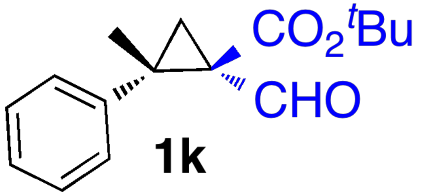

1k

4: 219 nm, 4 nm

Results

Pk #	Name	Retention Time	Area Percent
1		11.468	50.297
2		18.304	49.703

Totals	
	100.000

```
xx-vii-297 whelk 2%@0.8 ml
C:\EZStart\Projects\Default\Method\Shifa 4-t-Butylbenzene (OD-H 10-90).met
C:\EZStart\Projects\Default\Data\xx-vii-297 whelk 2%@0.8 ml
```

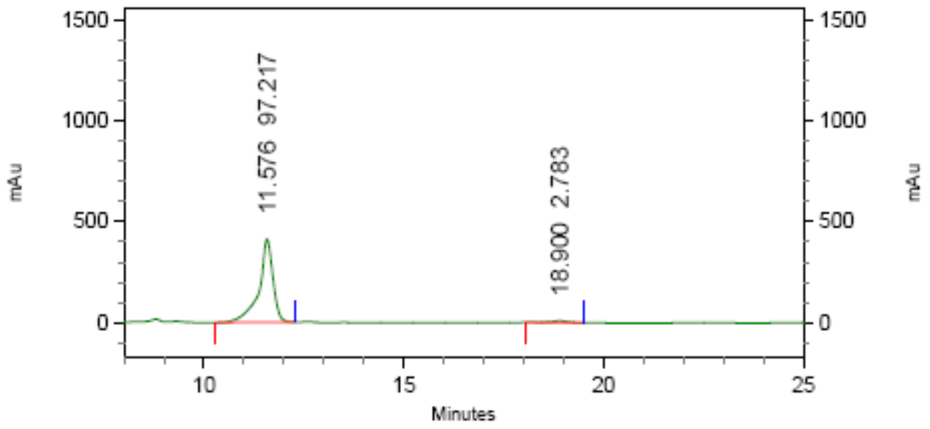

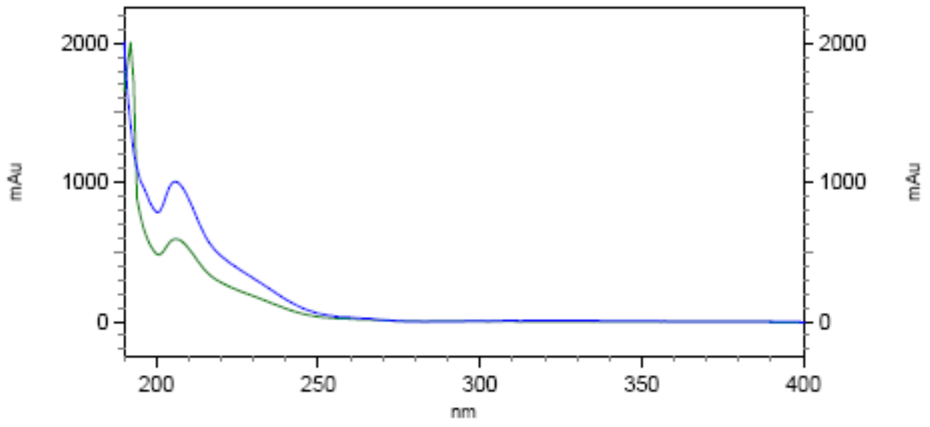

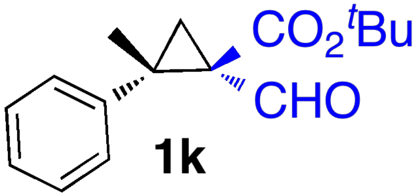

1k

4: 219 nm, 4 nm

Results

Pk #	Name	Retention Time	Area Percent
1		11.576	97.217
2		18.900	2.783
Totals			100.000

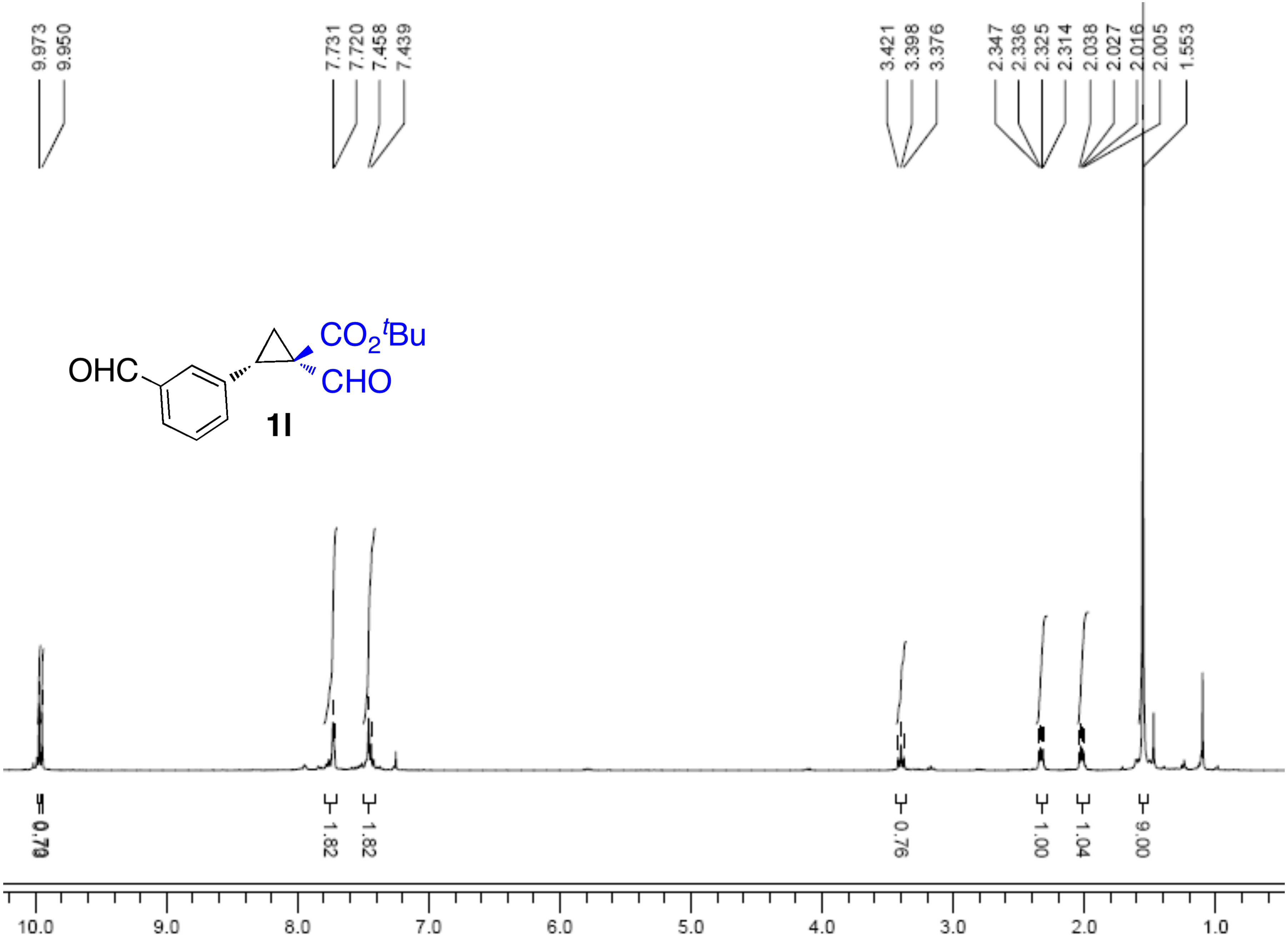
9.973
9.950
7.731
7.720
7.458
7.439
3.421
3.398
3.376
2.347
2.336
2.325
2.314
2.038
2.027
2.016
2.005
1.553
OHC
CO2tBu
CHO
1l
0.70
1.82
1.82
0.76
1.00
1.04
9.00
10.0
9.0
8.0
7.0
6.0
5.0
4.0
3.0
2.0
1.0

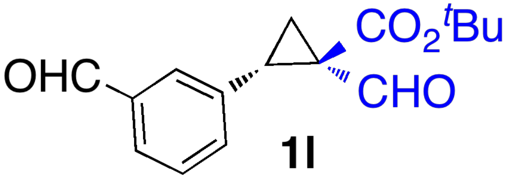
OHC
CO2tBu
CHO

1l

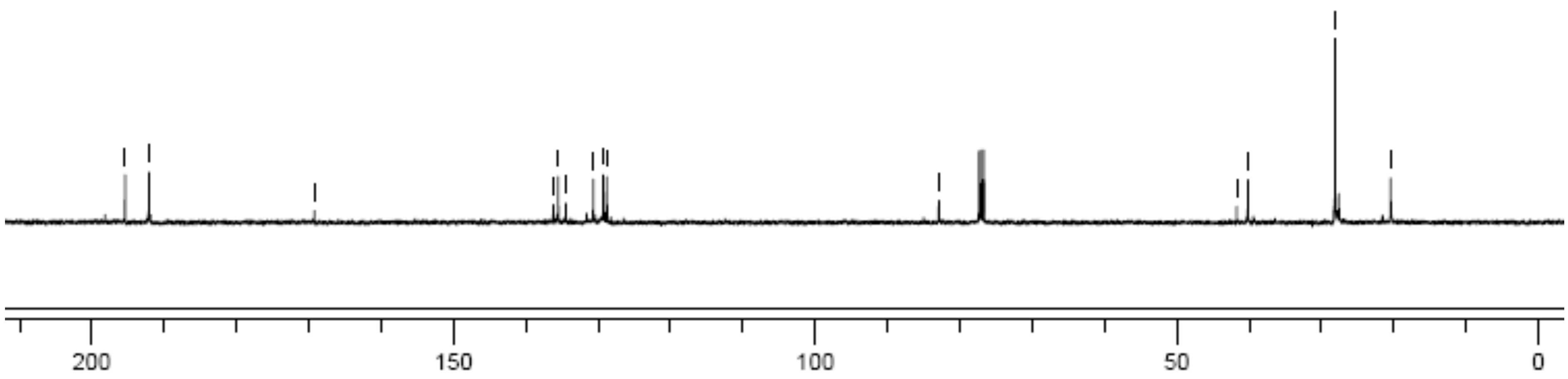
195.394
192.080
169.199
136.192
135.515
134.468
130.670
129.273
128.773
82.891
41.711
40.183
28.128
20.443
200
150
100
50
0

xx-viii-122-1 OJH 5%@1.0 ml
C:\EZStart\Projects\Default\Method\snowtemp short.met
C:\EZStart\Projects\Default\Data\xx-viii-122-1 OJH 5%@1.0 ml

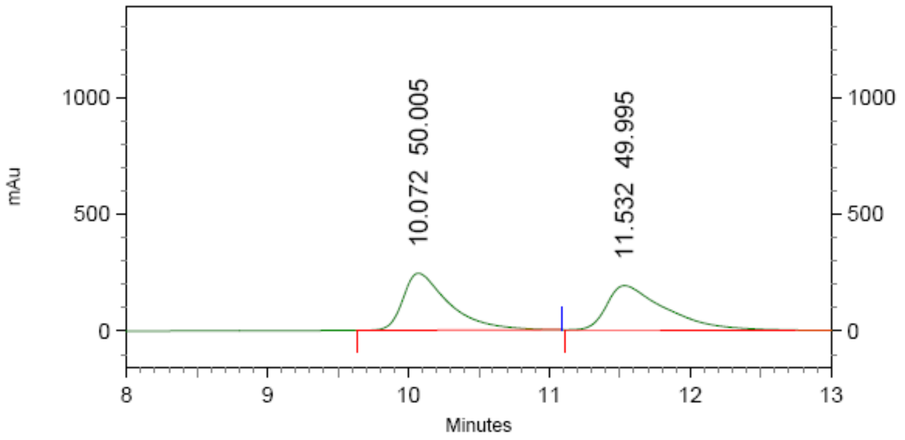

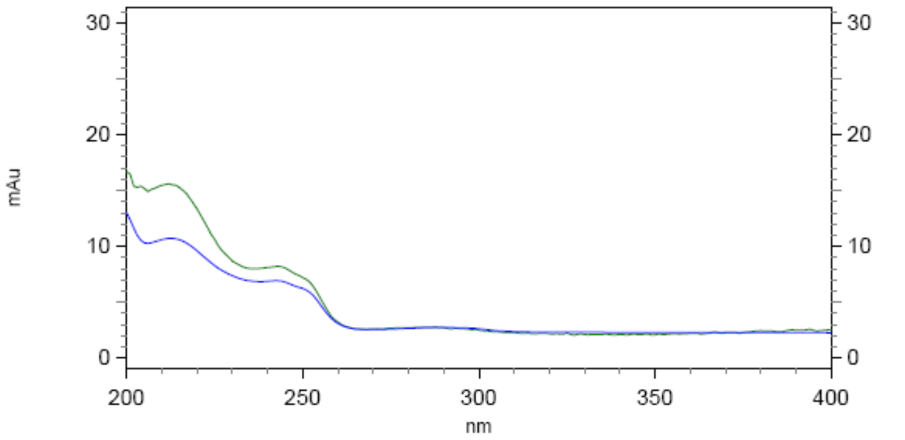

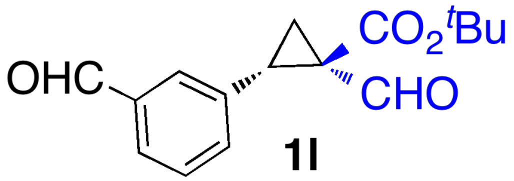

1l

8: 223 nm, 4 nm
Results

Pk #	Name	Retention Time	Area Percent
1		10.072	50.005
2		11.532	49.995
Totals			100.000

xx-viii-122-2 OJH 5%@1.0 ml
C:\EZStart\Projects\Default\Method\snowtemp short.met
C:\EZStart\Projects\Default\Data\xx-viii-122-2 OJH 5%@1.0 ml

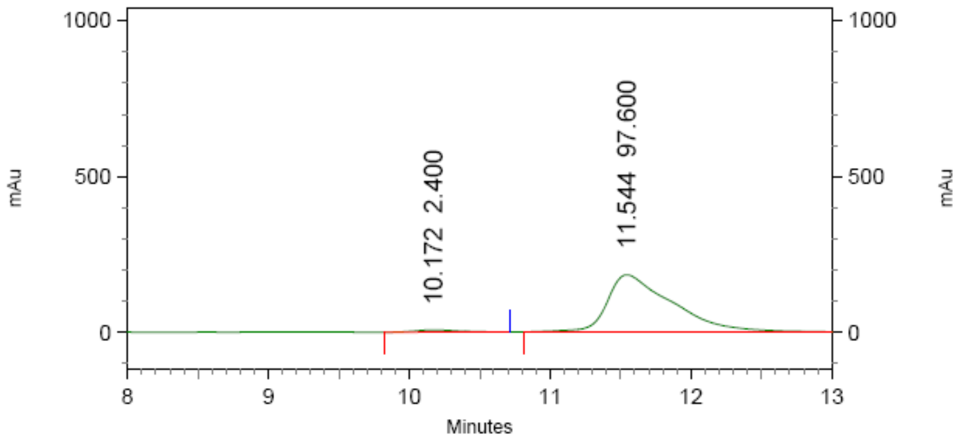

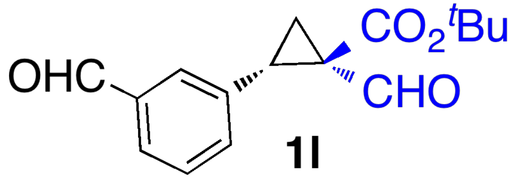

1l

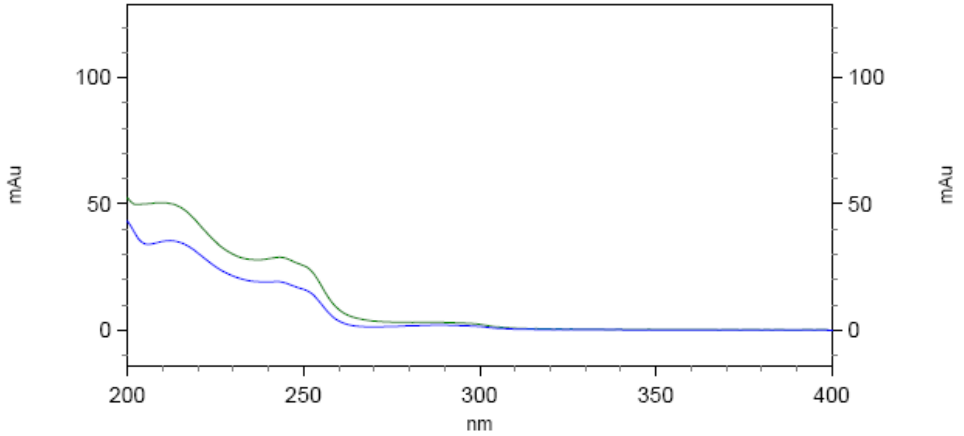

8: 223 nm, 4 nm

Results

Pk #	Name	Retention Time	Area Percent
1		10.172	2.400
2		11.544	97.600
Totals			100.000

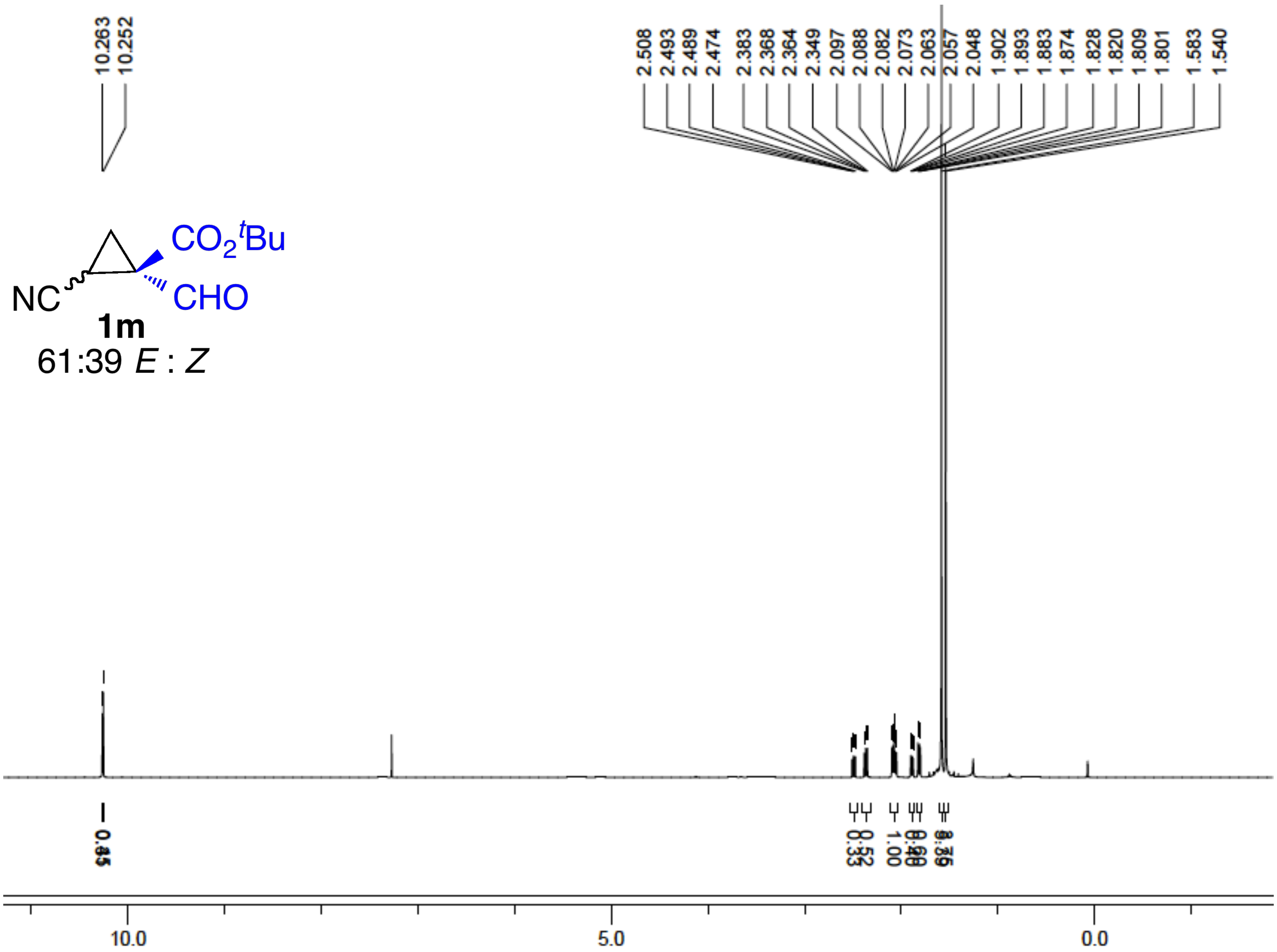

10.263
10.252
2.508
2.493
2.489
2.474
2.383
2.368
2.364
2.349
2.097
2.088
2.082
2.073
2.063
2.057
2.048
1.902
1.893
1.883
1.874
1.828
1.820
1.809
1.801
1.583
1.540
$CO_2{}^tBu$
CHO
NC
1.00
10.0
5.0
0.0

1m
61:39 *E* : *Z*

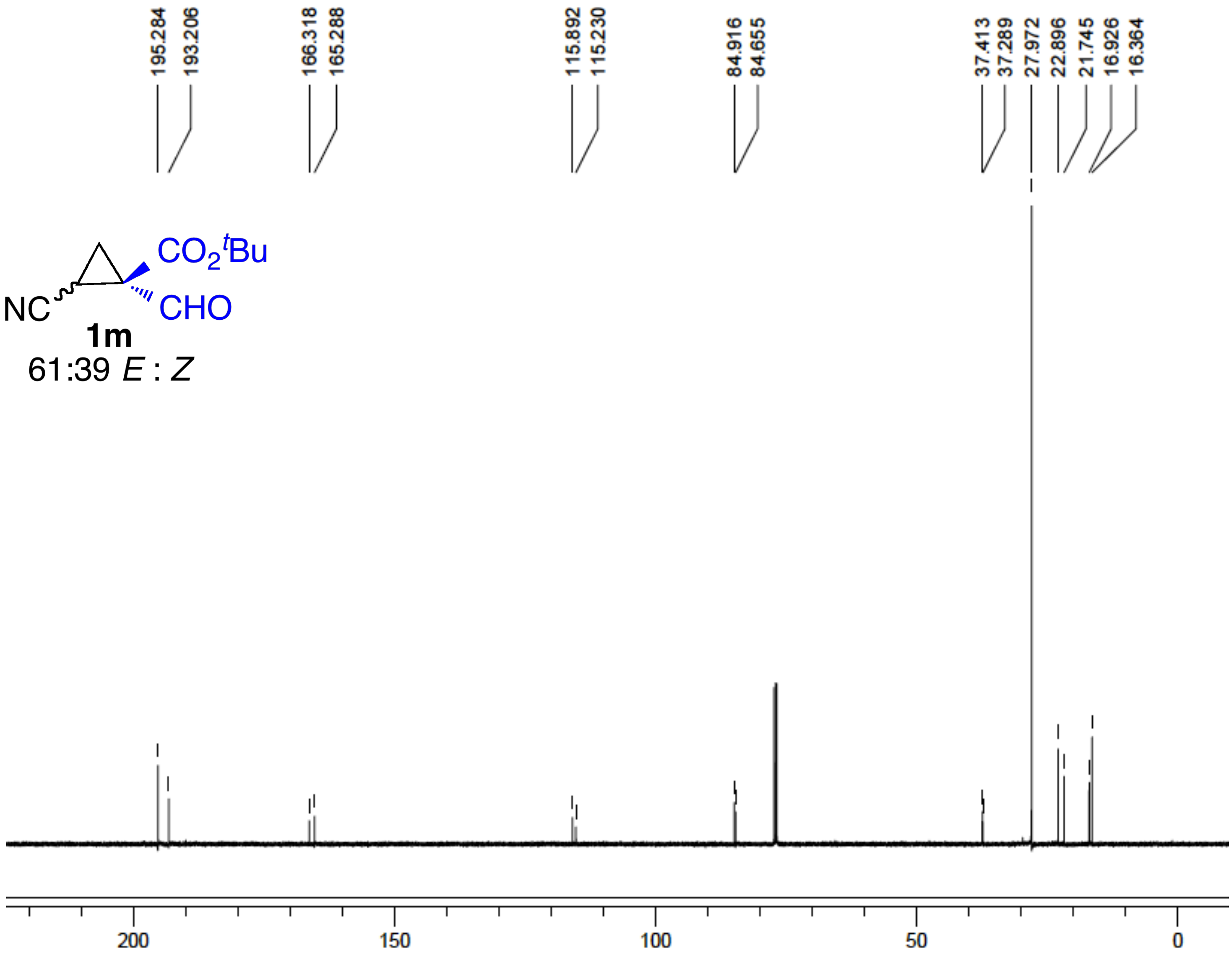
195.284
193.206
166.318
165.288
115.892
115.230
84.916
84.655
37.413
37.289
27.972
22.896
21.745
16.926
16.364
$CO_2{}^tBu$
CHO
NC
200
150
100
50
0

1m

61:39 *E* : *Z*

XC-IX-216RE-ADH1%1ml
C:\EZStart\Projects\Default\Method\XC-1%-ADH1ml.met
C:\EZStart\Projects\Default\Data\XC-IX-216RE-ADH1%1ml

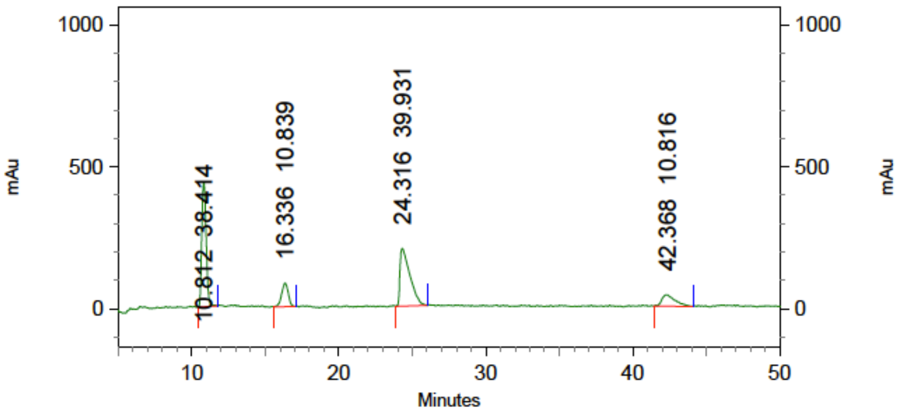

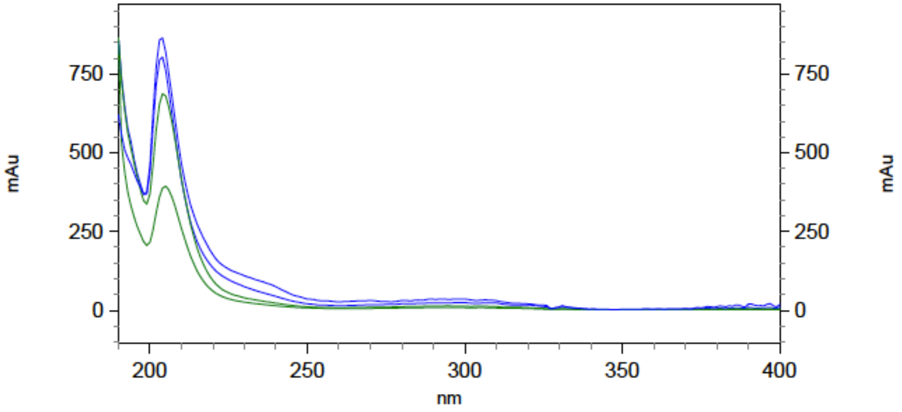

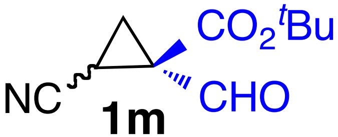

1m

3: 206 nm, 4 nm
Results

Name	Retention Time	Area Percent	Pk #
	10.812	38.414	1
	16.336	10.839	2
	24.316	39.931	3
	42.368	10.816	4
Totals		100.000	

XC-IX-220-ADH1%1ml
C:\EZStart\Projects\Default\Method\XC-1%-ADH1ml.met
C:\EZStart\Projects\Default\Data\XC-IX-220-ADH1%1ml

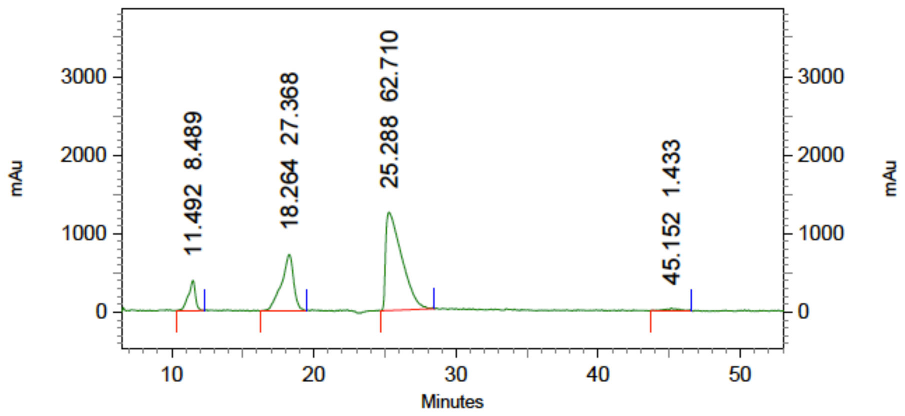

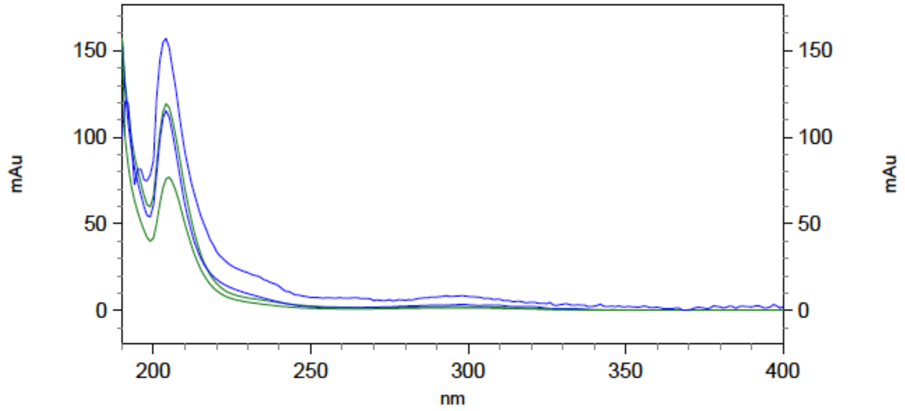

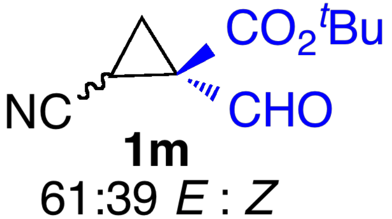

1m
61:39 *E* : *Z*

3: 207 nm, 4 nm
Results

Name	Retention Time	Area Percent	Pk #
	11.492	8.489	1
	18.264	27.368	2
	25.288	62.710	3
	45.152	1.433	4
Totals		100.000	

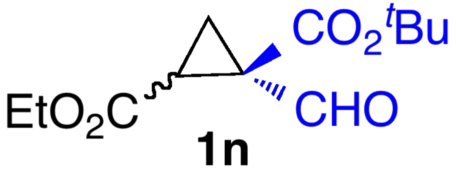
CO2tBu
CHO
EtO2C

1n
60:40 *E* : *Z*

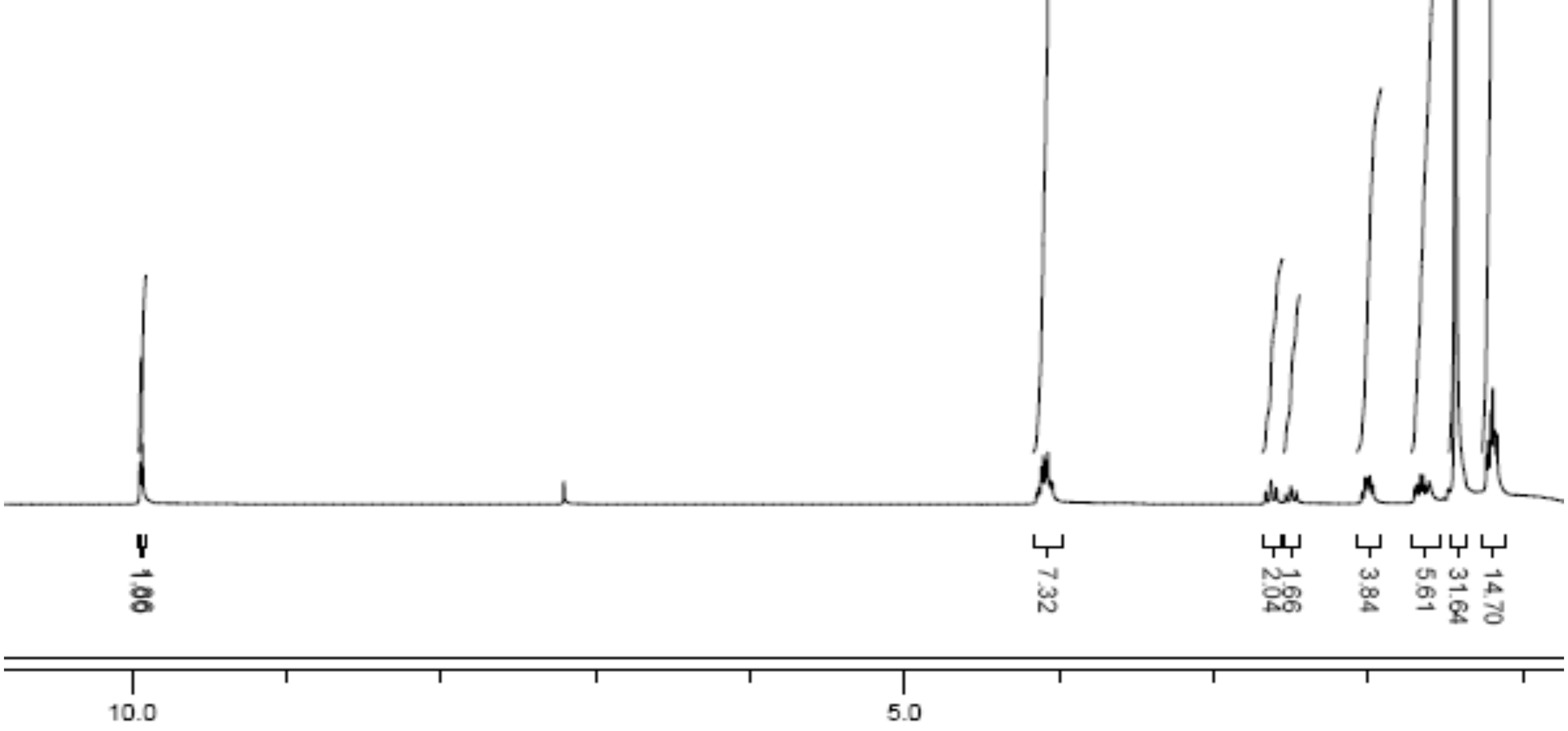
1.433
1.430
7.32
2.04
1.66
3.84
5.61
31.64
14.70
10.0
5.0

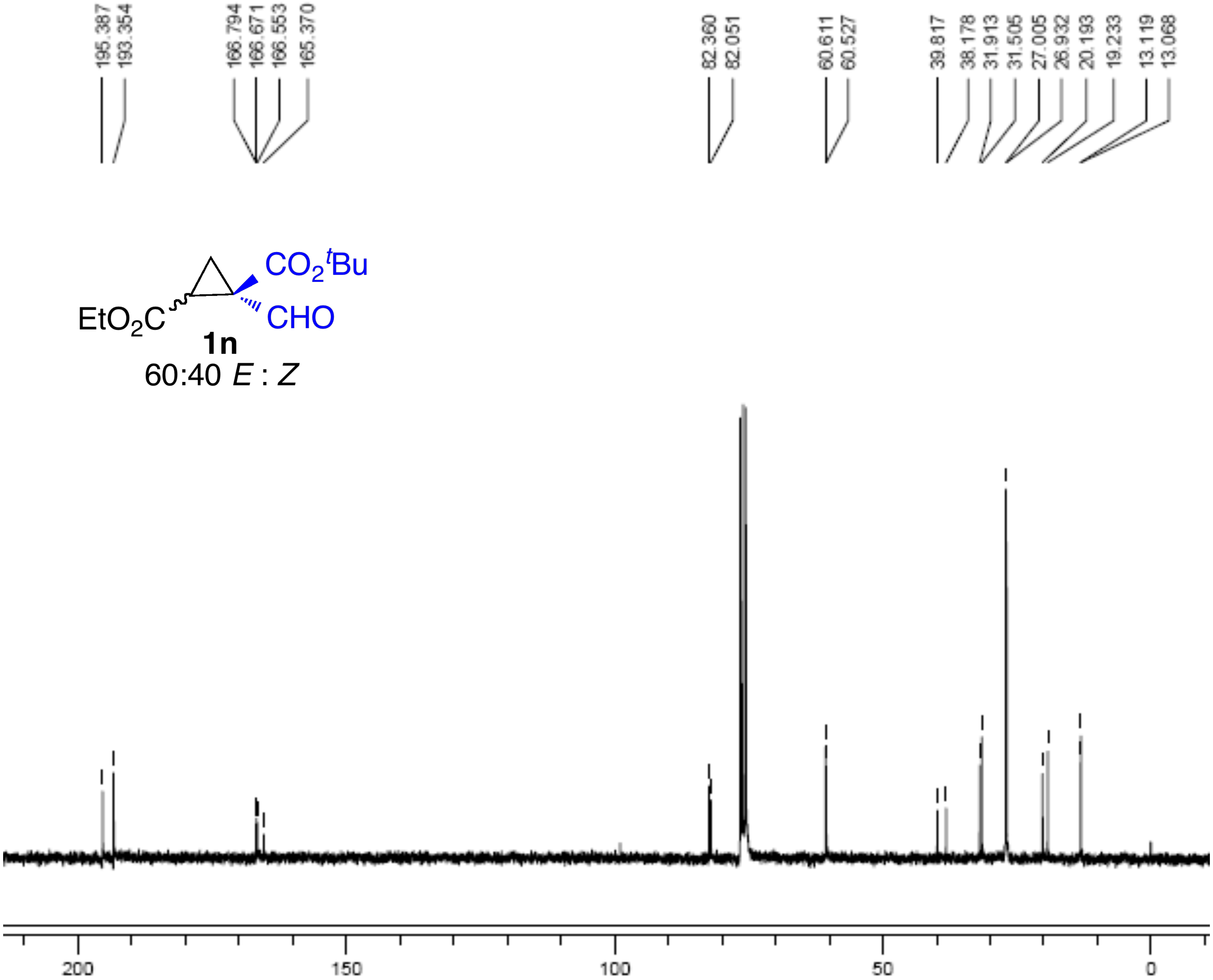
195.387
193.354
166.794
166.671
166.553
165.370
82.360
82.051
60.611
60.527
39.817
38.178
31.913
31.505
27.005
26.932
20.193
19.233
13.119
13.068
CO2tBu
EtO2C
CHO
200
150
100
50
0

1n

60:40 *E* : *Z*

Sample Name : **xx-vi-94 DCB 5 again**
Sample ID : **xx-vi-94 DCB 5 again**

Chromatogram

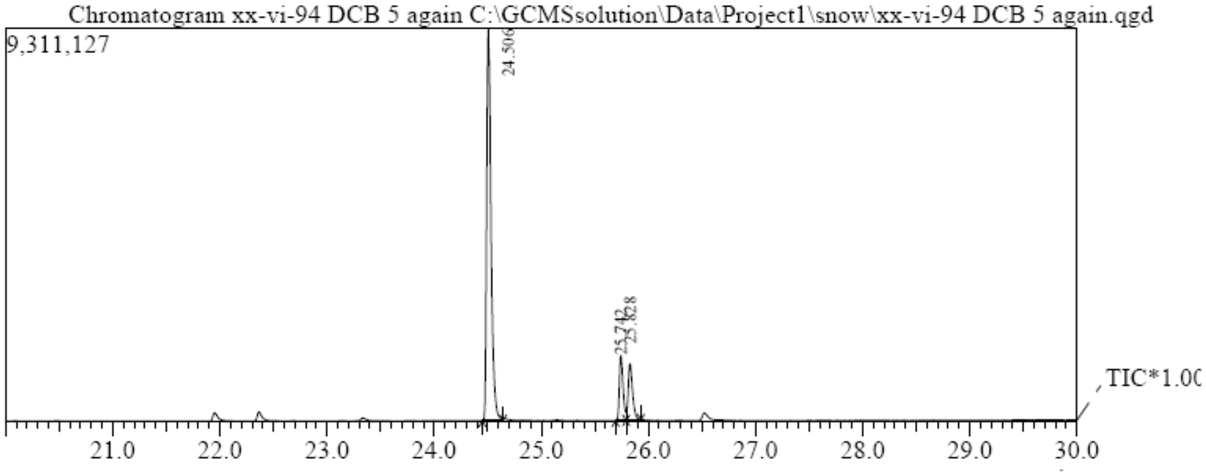

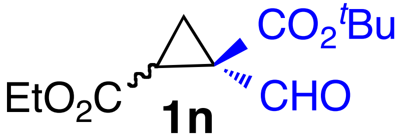

1n

Peak Report TIC

Peak#	R.Time	Area%	Name	Base m/z
1	24.506	78.02		57.10
2	25.742	10.98		57.10
3	25.828	11.00		57.10
		100.00		

Spectrum

Peak#:1 R.Time:24.5(Scan#:5853)
MassPeaks:157

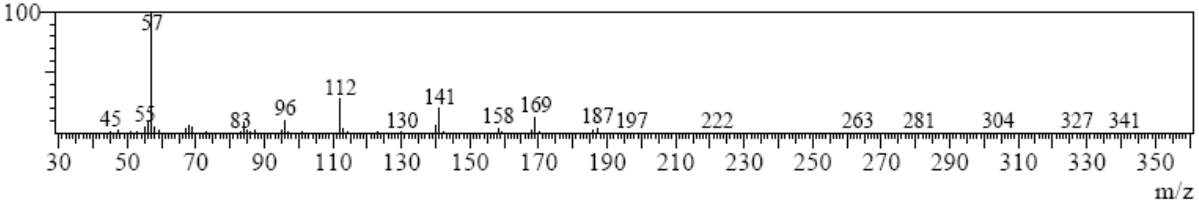

Peak#:2 R.Time:25.7(Scan#:6224)
MassPeaks:140

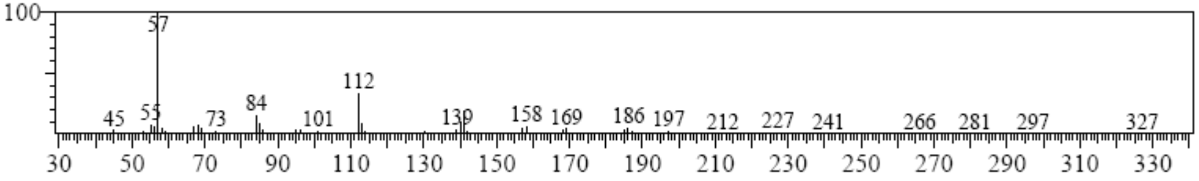

Sample Name : **xx-vi-102 DCB 5**
Sample ID : **xx-vi-102 DCB 5**

Chromatogram

Chromatogram xx-vi-102 DCB 5 C:\GCMSsolution\Data\Project1\snow\xx-vi-102 DCB 5.qgd

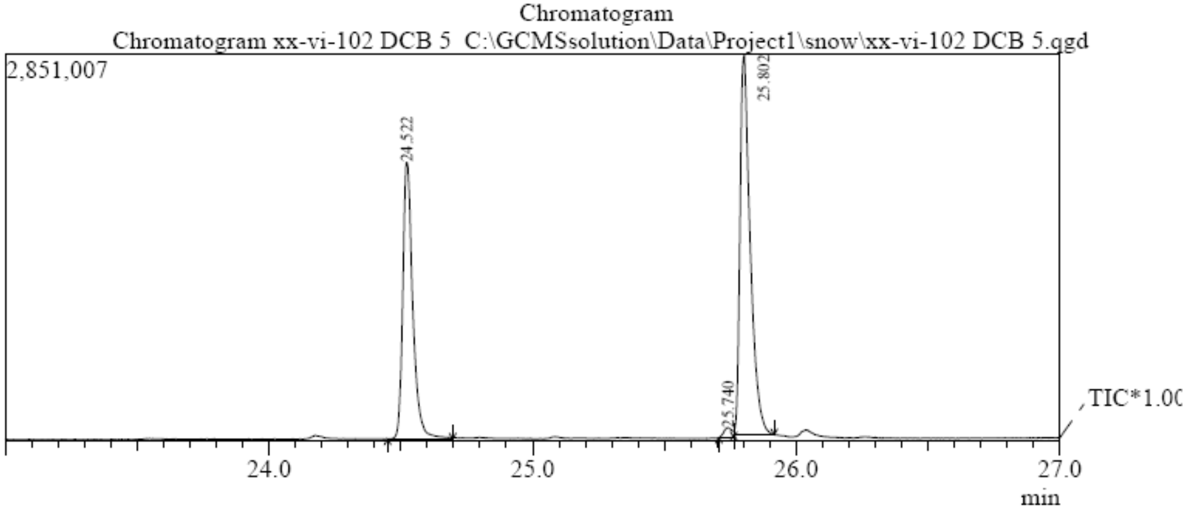

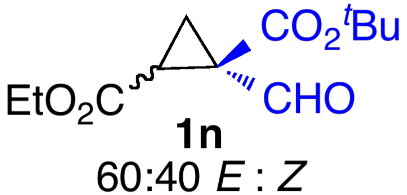

1n
60:40 *E* : *Z*

Peak Report TIC

Peak#	R.Time	Area%	Name	Base m/z
1	24.522	41.14		57.10
2	25.740	1.23		57.10
3	25.802	57.63		57.10
		100.00		

Spectrum

Peak#:1 R.Time:24.5(Scan#:5858)
MassPeaks:136

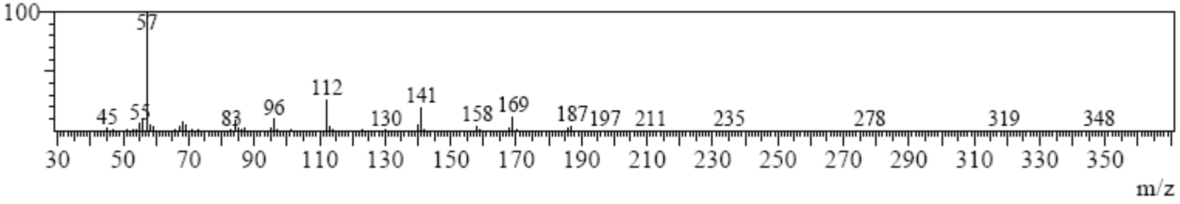

Peak#:2 R.Time:25.7(Scan#:6223)
MassPeaks:96

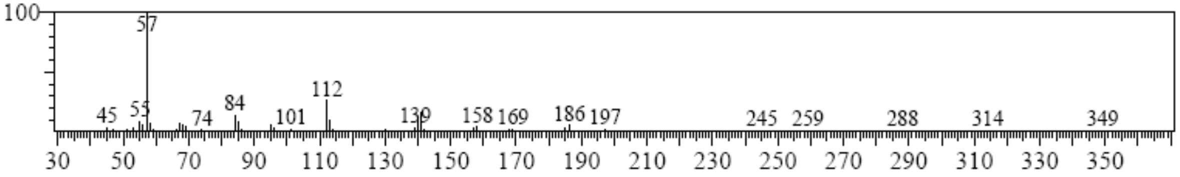

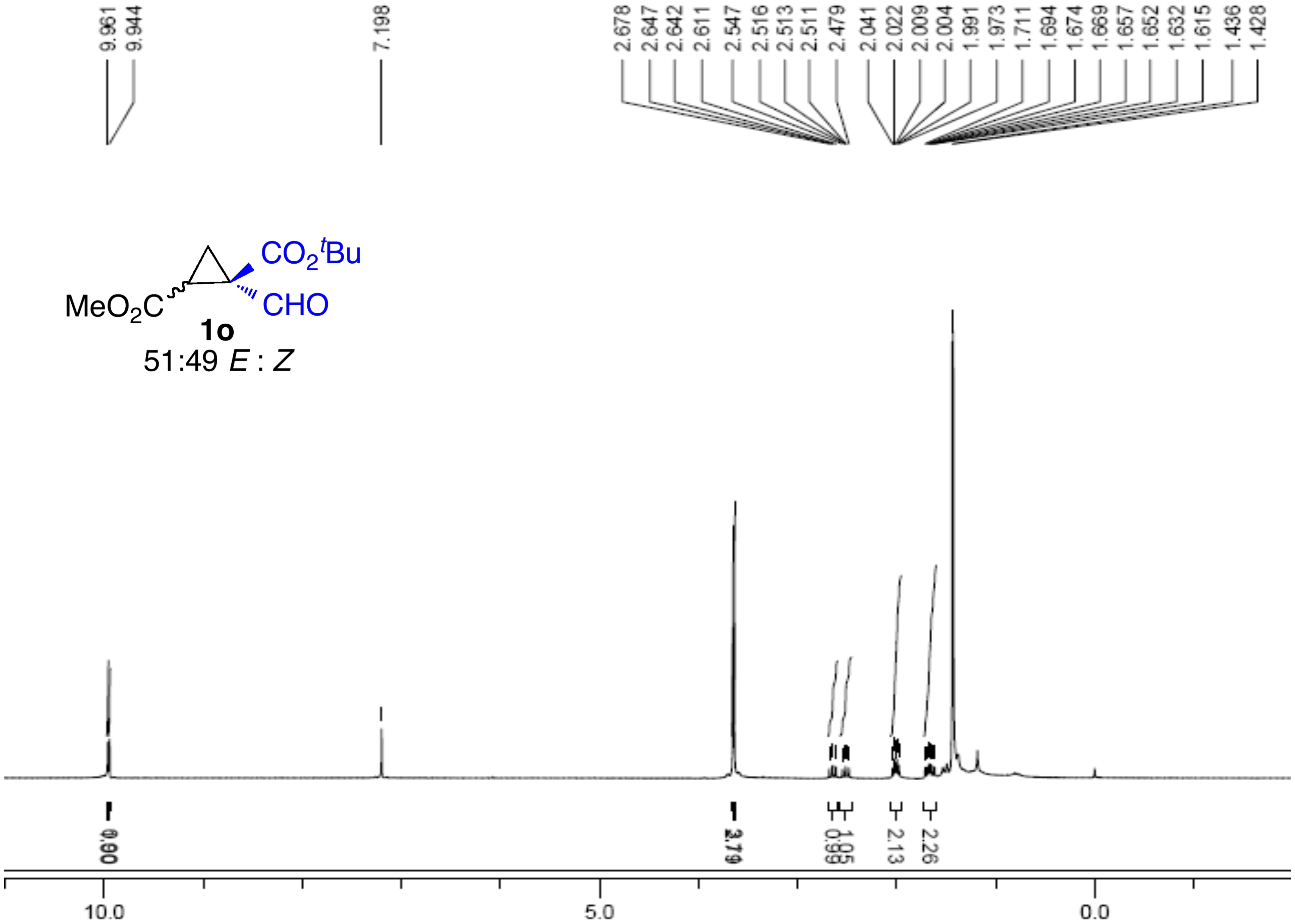
9.961
9.944
7.198
2.678
2.647
2.642
2.611
2.547
2.516
2.513
2.511
2.479
2.041
2.022
2.009
2.004
1.991
1.973
1.711
1.694
1.674
1.669
1.657
1.652
1.632
1.615
1.436
1.428
CO2tBu
CHO
MeO2C
1o
51:49 E : Z
0.90
3.79
0.99
1.05
2.13
2.26
10.0
5.0
0.0

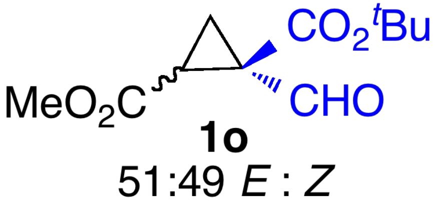
MeO2C
CO2tBu
CHO

1o

51:49 *E* : *Z*

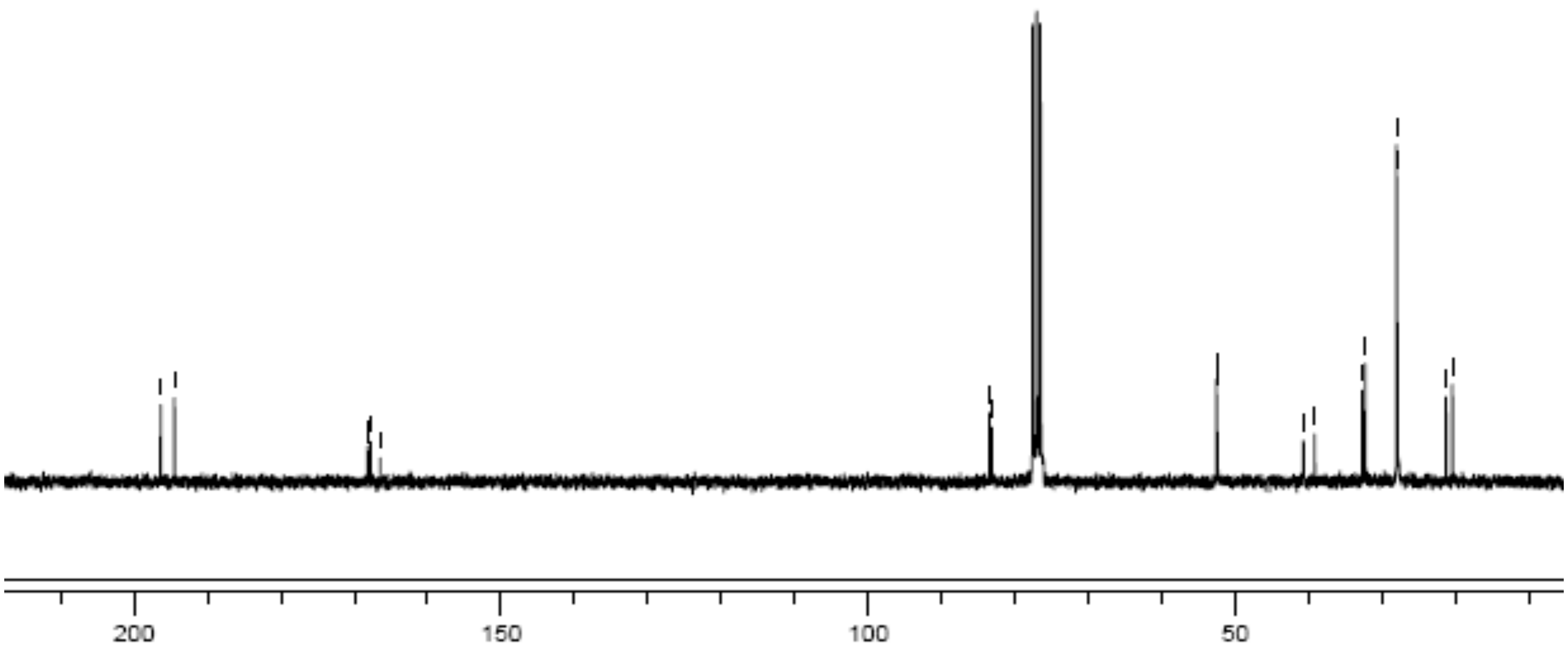
196.357
194.475
168.160
168.035
167.755
166.460
83.466
83.153
52.548
52.460
40.703
39.193
32.726
32.323
28.038
27.953
21.287
20.434
200
150
100
50

Sample Name : **xx-vi-93 DexCB 5**
Sample ID : **xx-vi-93 DexCB 5**

Chromatogram

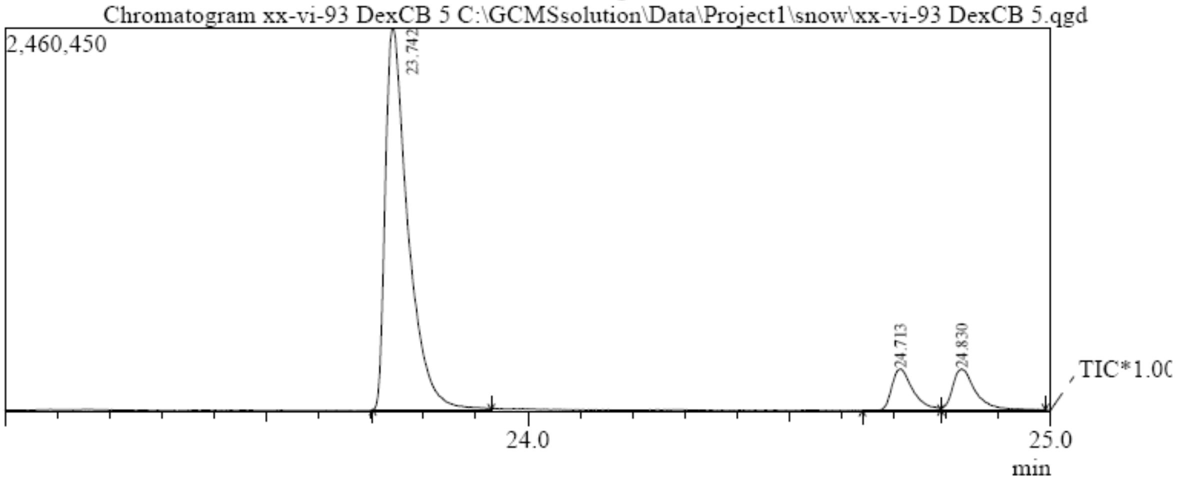

Peak Report TIC

Peak#	R.Time	Area%	Name	Base m/z
1	23.742	82.78		57.10
2	24.713	8.44		57.10
3	24.830	8.78		57.10
		100.00		

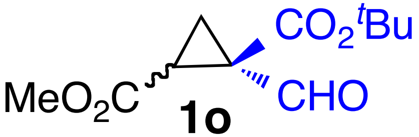

1o

Spectrum

Peak#:1 R.Time:23.7(Scan#:5624)
MassPeaks:116

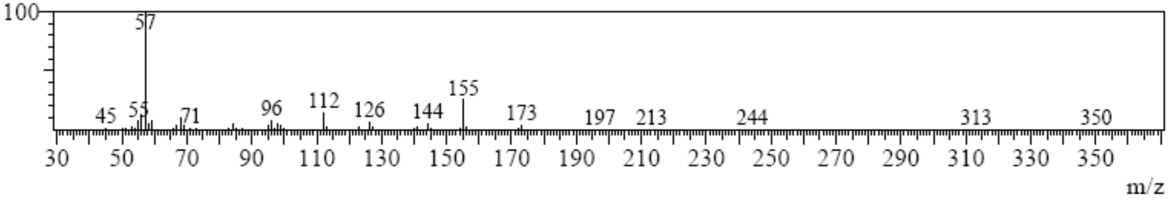

Peak#:2 R.Time:24.7(Scan#:5915)
MassPeaks:103

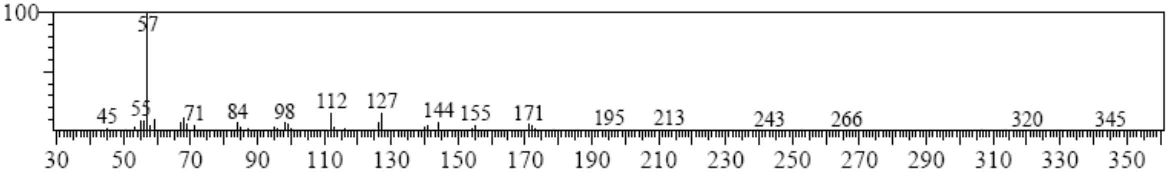

Sample Name : xx-vi-103 DexCB 5
Sample ID : xx-vi-103 DexCB 5

Chromatogram

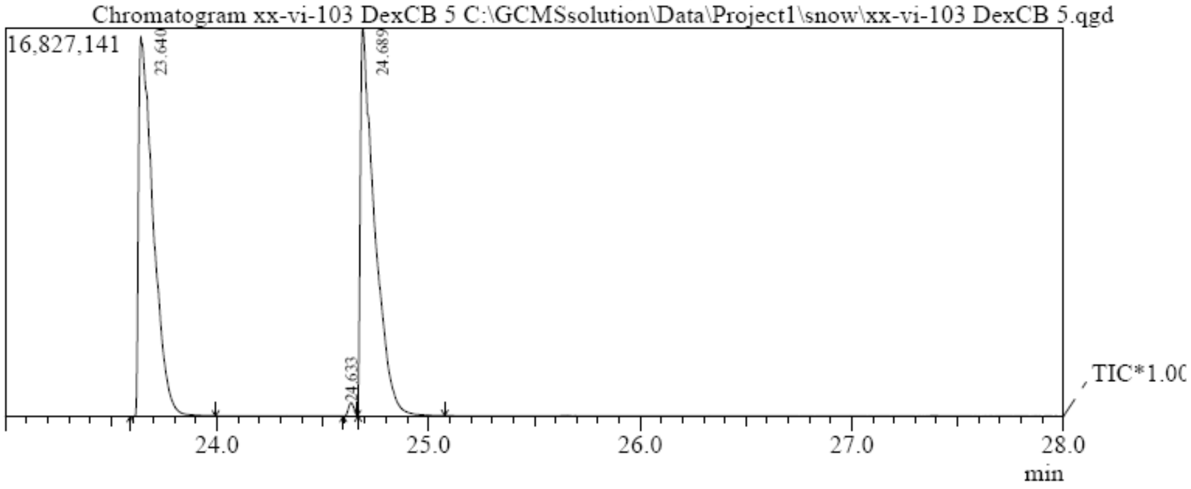

Peak Report TIC

Peak#	R.Time	Area%	Name	Base m/z
1	23.640	49.71		57.10
2	24.633	0.74		57.10
3	24.689	49.55		57.10
		100.00		

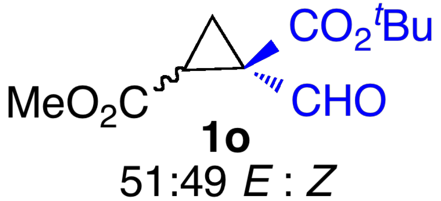

1o
51:49 *E* : *Z*

Spectrum

Peak#:1 R.Time:23.6(Scan#:5593)
MassPeaks:150

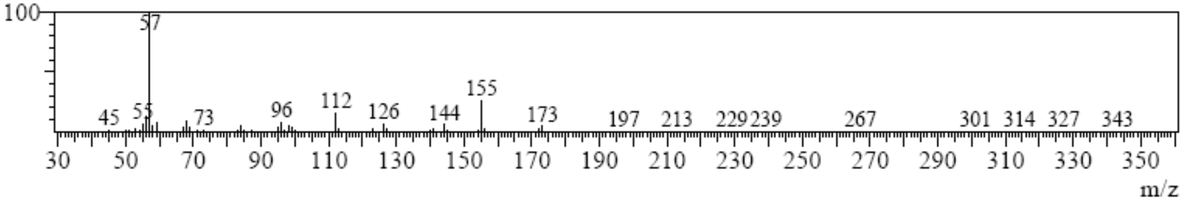

Peak#:2 R.Time:24.6(Scan#:5891)
MassPeaks:104

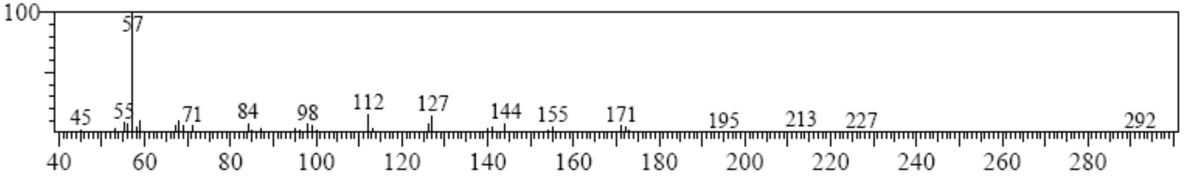

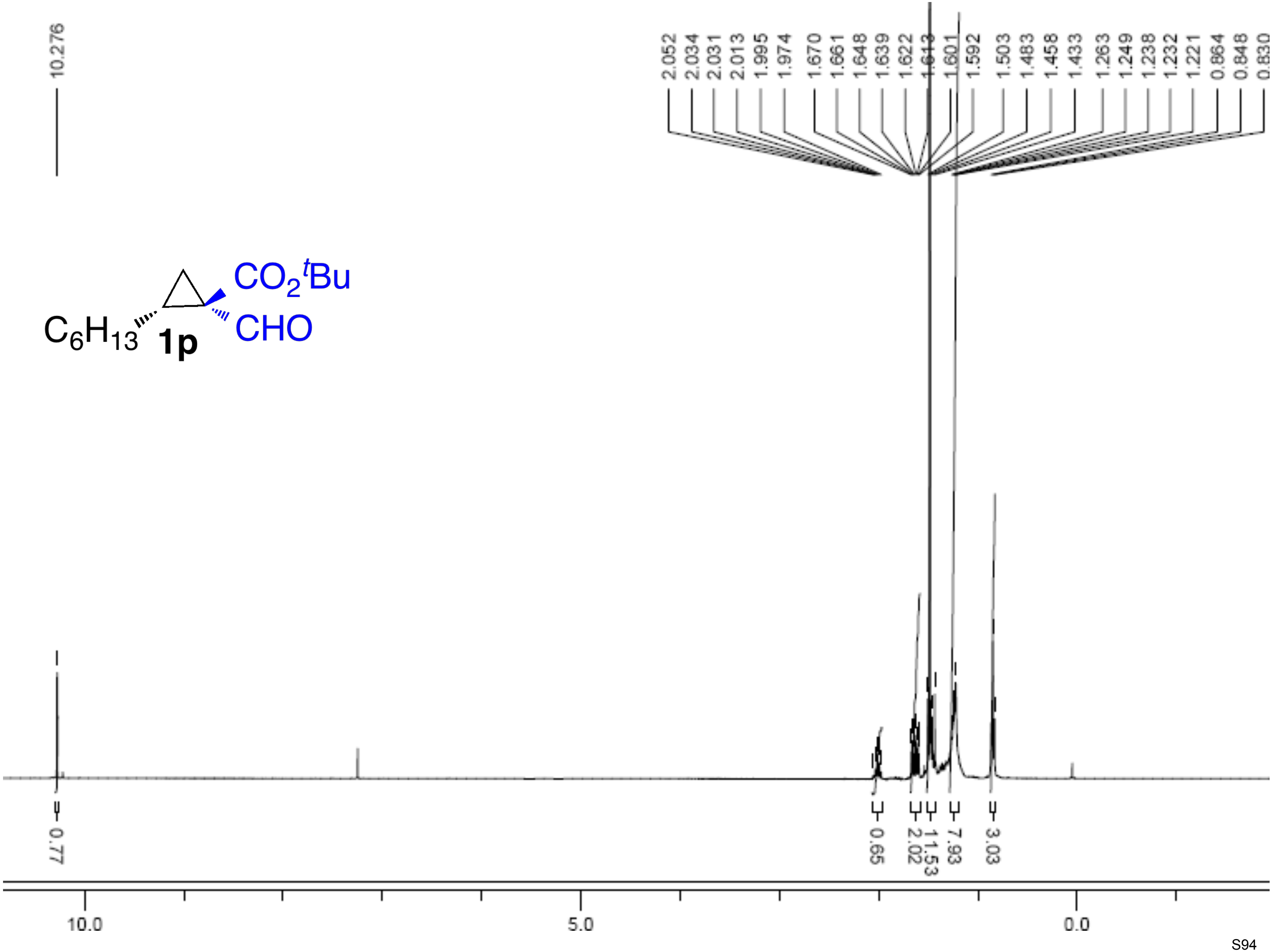
10.276
2.052
2.034
2.031
2.013
1.995
1.974
1.670
1.661
1.648
1.639
1.622
1.613
1.601
1.592
1.503
1.483
1.458
1.433
1.263
1.249
1.238
1.232
1.221
0.864
0.848
0.830
CO2tBu
CHO
C6H13
1p
0.77
0.65
2.02
11.53
7.93
3.03
10.0
5.0
0.0

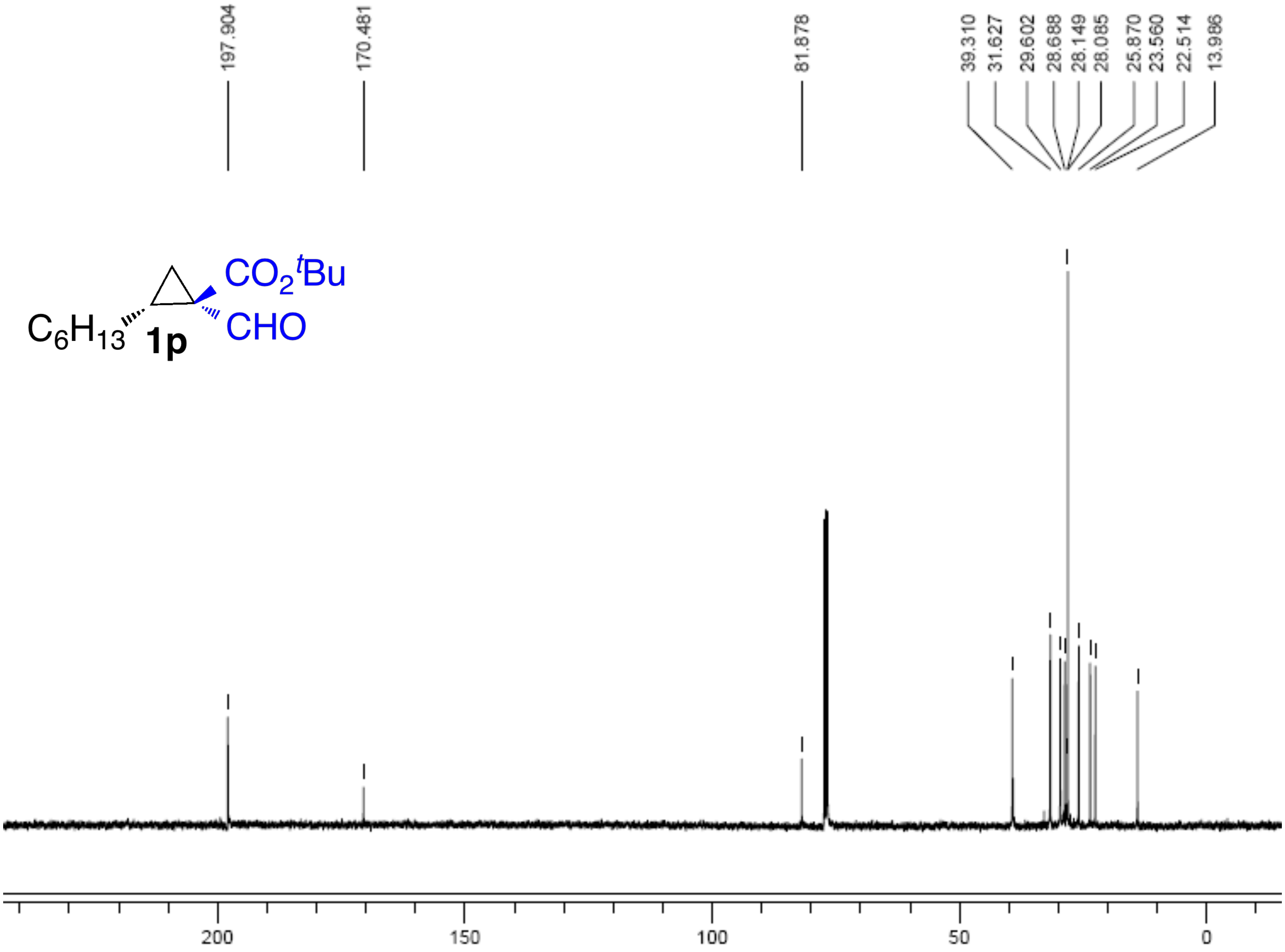

197.904
170.481
81.878
39.310
31.627
29.602
28.688
28.149
28.085
25.870
23.560
22.514
13.986
200
150
100
50
0

Sample Name : **xx-viii-113 DB 3**
Sample ID : **xx-viii-113 DB 3**

Chromatogram

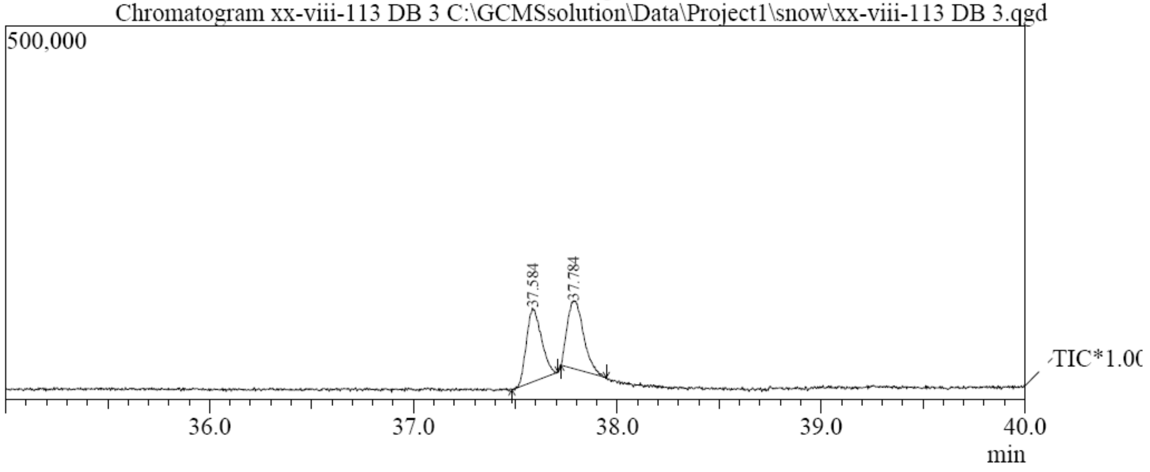

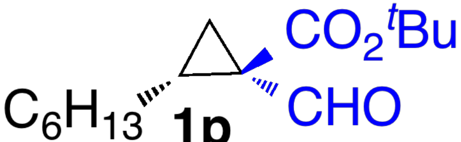

1p

Peak Report TIC

Peak#	R.Time	Area%	Name	Base m/z
1	37.584	49.91		57.10
2	37.784	50.09		57.05
		100.00		

Spectrum

Peak#:1 R.Time:37.6(Scan#:9776)
MassPeaks:136

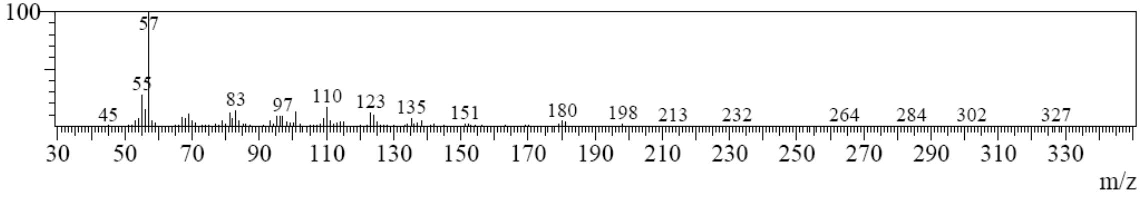

Peak#:2 R.Time:37.8(Scan#:9836)
MassPeaks:137

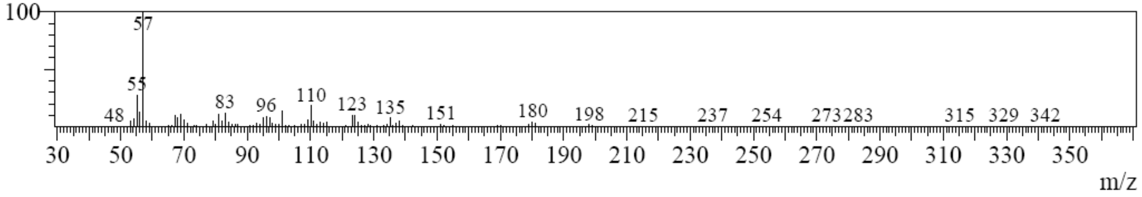

Sample Name : **xx-viii-117 DB 3**
Sample ID : **xx-viii-117 DB 3**

Chromatogram

Chromatogram xx-viii-117 DB 3 C:\GCMSsolution\Data\Project1\snow\xx-viii-117 DB 3.qgd

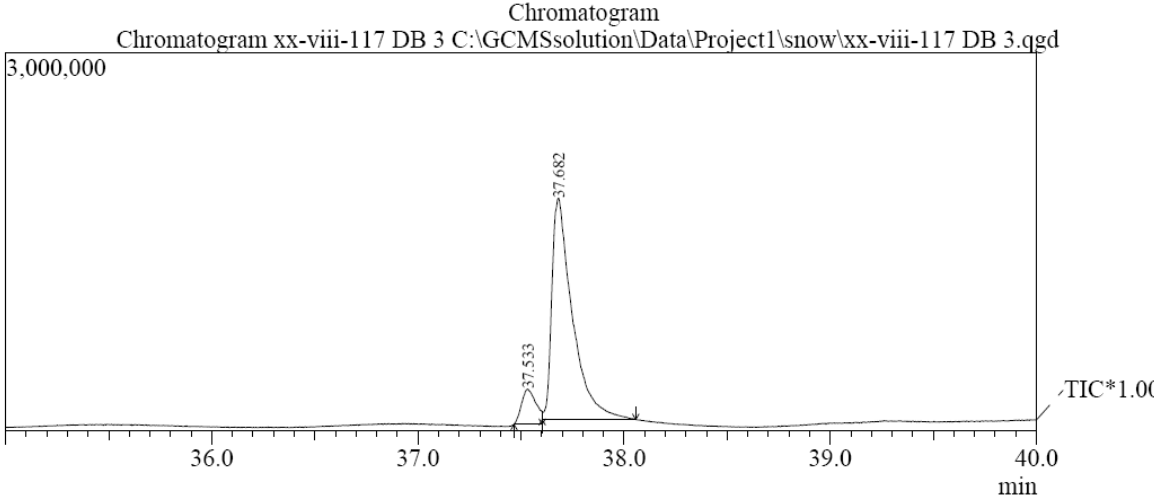

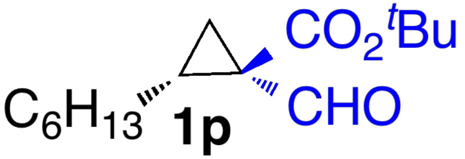

Peak Report TIC

Peak#	R.Time	Area%	Name	Base m/z
1	37.533	10.12		57.10
2	37.682	89.88		57.10
		100.00		

Spectrum

Peak#:1 R.Time:37.5(Scan#:9761)
MassPeaks:165

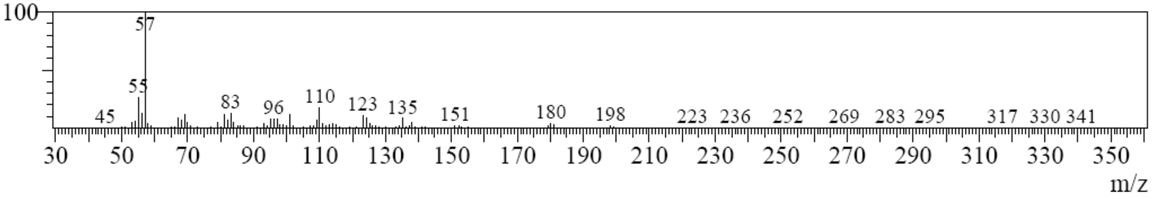

Peak#:2 R.Time:37.7(Scan#:9806)
MassPeaks:169

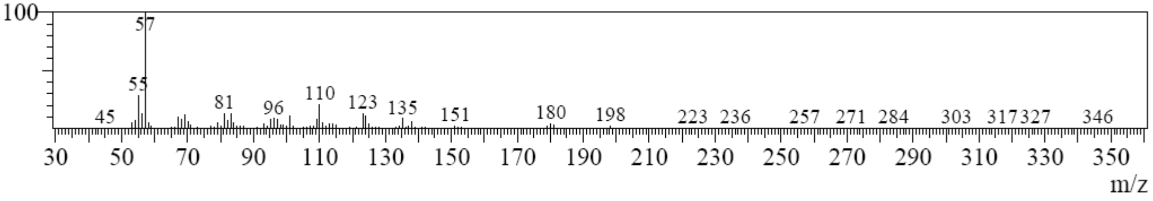

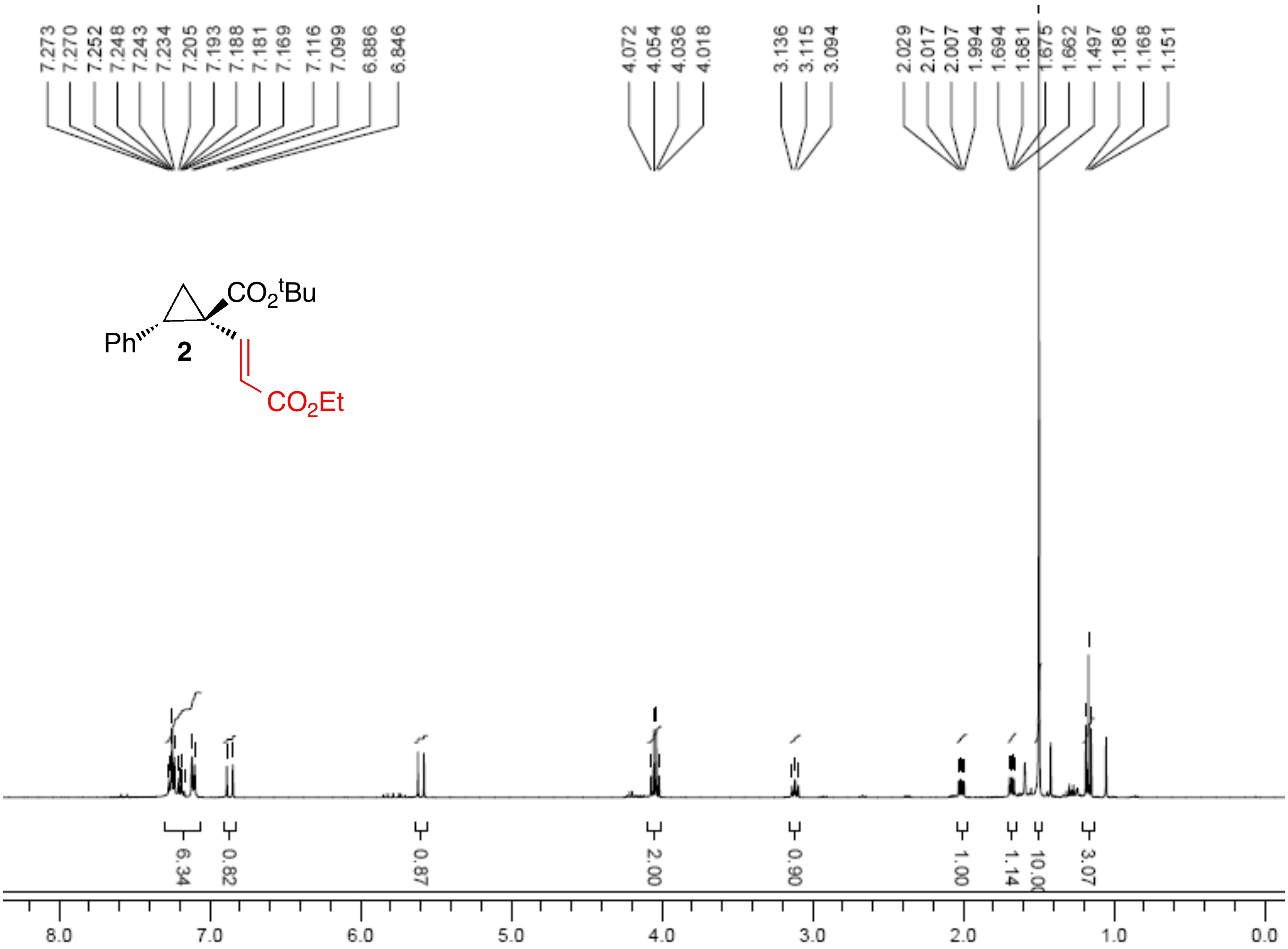
7.273 7.270 7.252 7.248 7.243 7.234 7.205 7.193 7.188 7.181 7.169 7.116 7.099 6.886 6.846
4.072 4.054 4.036 4.018
3.136 3.115 3.094
2.029 2.017 2.007 1.994 1.694 1.681 1.675 1.662 1.497 1.186 1.168 1.151
CO2tBu
Ph
2
CO2Et
6.34 0.82 0.87 2.00 0.90 1.00 1.14 10.00 3.07
8.0 7.0 6.0 5.0 4.0 3.0 2.0 1.0 0.0

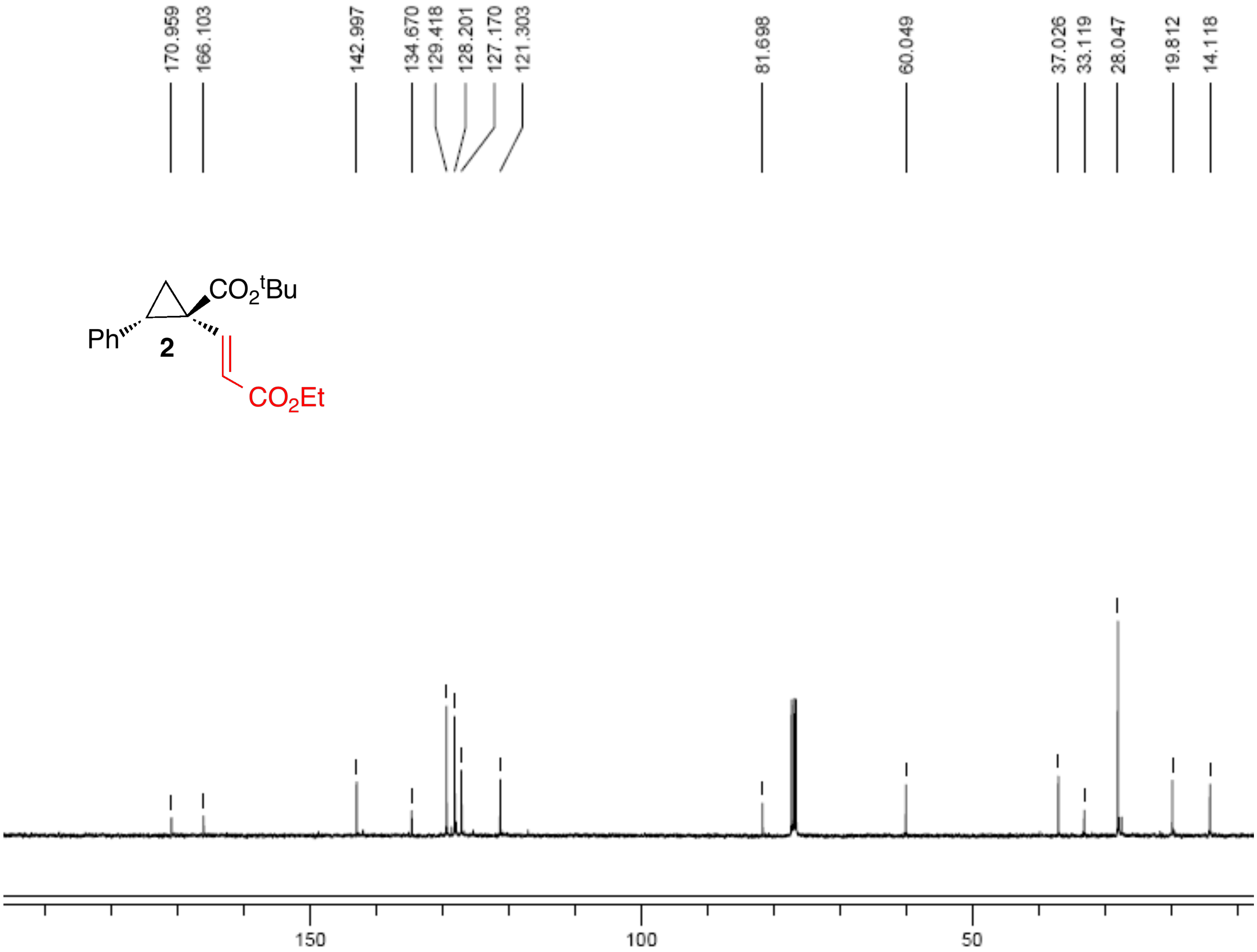
170.959
166.103
142.997
134.670
129.418
128.201
127.170
121.303
81.698
60.049
37.026
33.119
28.047
19.812
14.118
CO2tBu
Ph
2
CO2Et
150
100
50

```
xx-viii-110 whelk 1%@1.0 ml
C:\EZStart\Projects\Default\Method\Shifa-P-Cyclopropane 5%Whelk.met
C:\EZStart\Projects\Default\Data\xx-viii-110 whelk 1%@1.0 ml
```

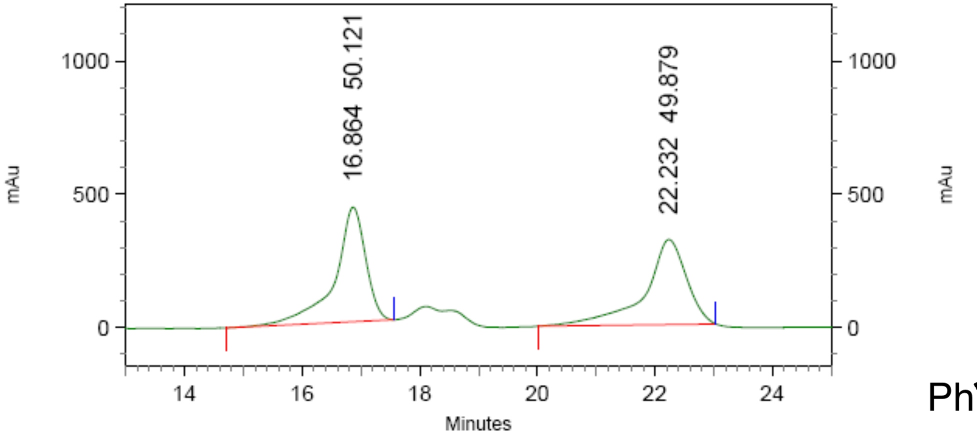

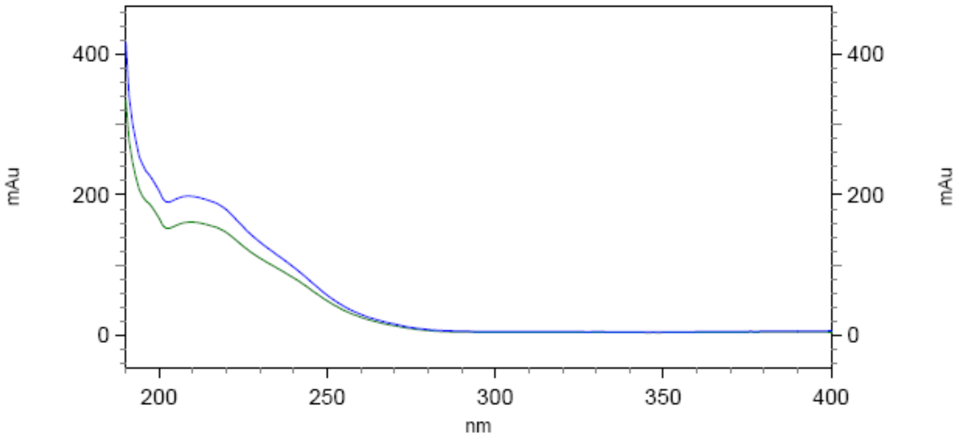

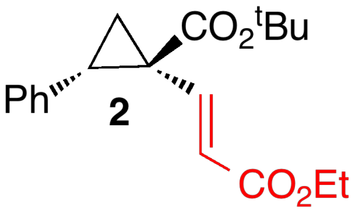

2: 209 nm, 4 nm

Results

Pk # Name	Retention Time	Area Percent
1	16.864	50.121
2	22.232	49.879
Totals		100.000

xx-viii-114 whelk 1%@1.0 ml again
C:\EZStart\Projects\Default\Method\Shifa-P-Cyclopropane 5%Whelk.met
C:\EZStart\Projects\Default\Data\xx-viii-114 whelk 1%@1.0 ml again

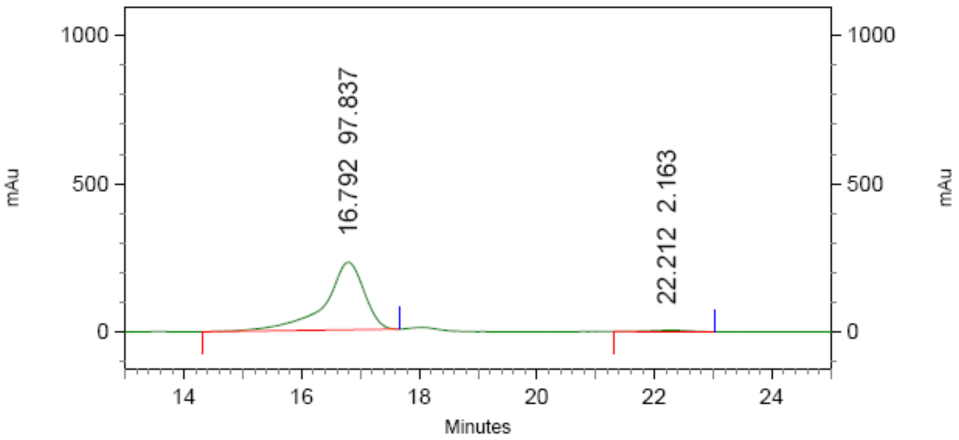

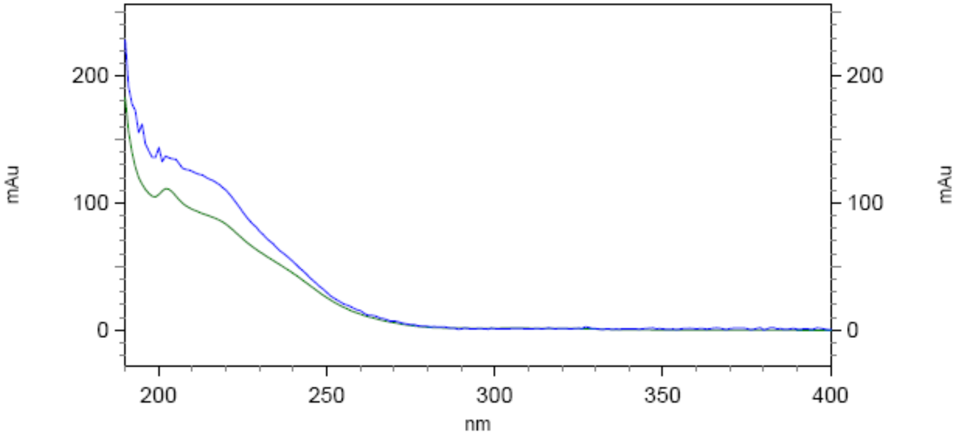

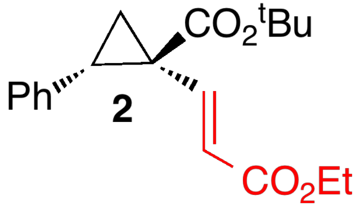

2

2: 209 nm, 4 nm
Results

Pk #	Name	Retention Time	Area Percent
1		16.792	97.837
2		22.212	2.163
Totals			100.000

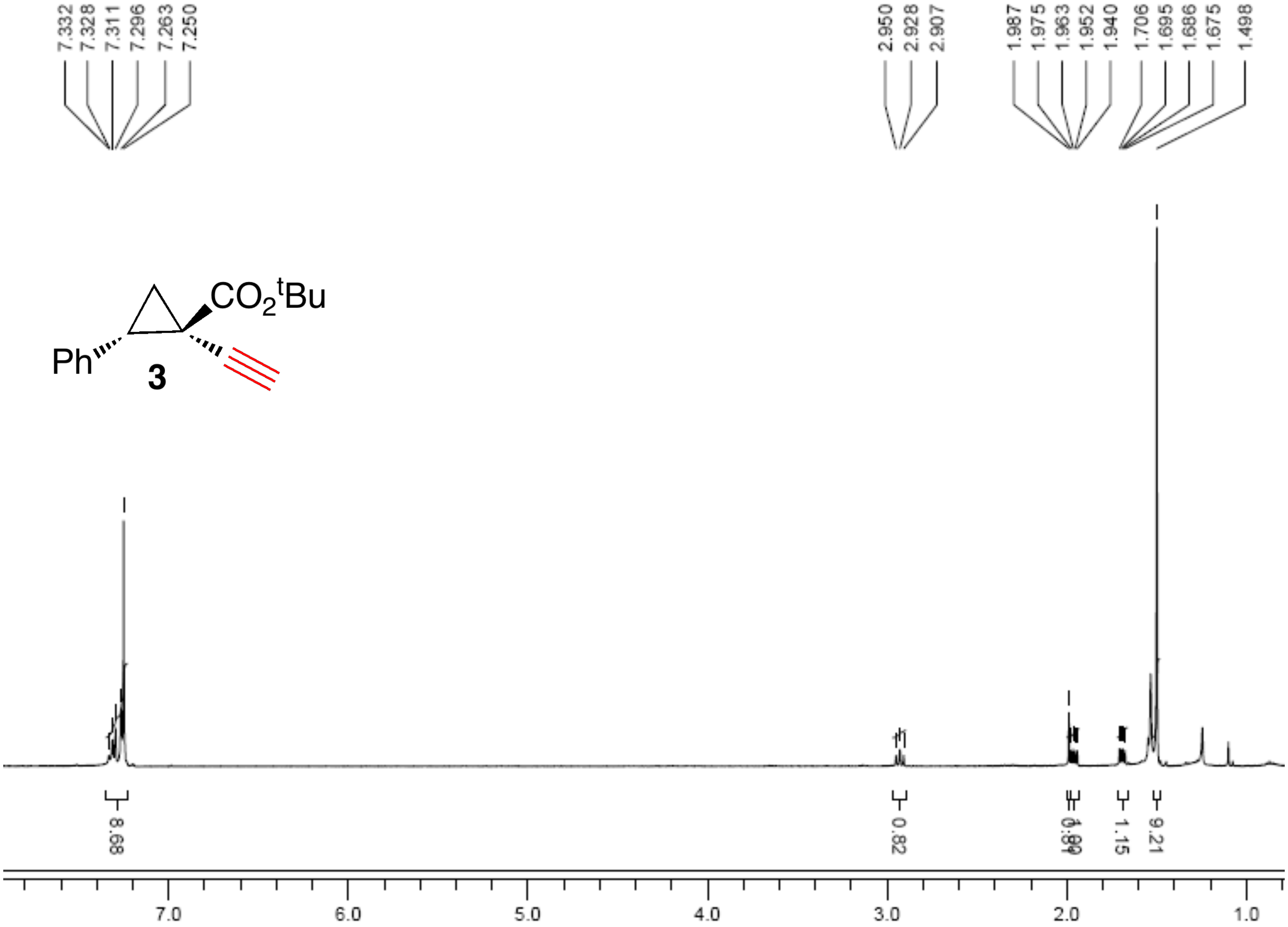
7.332
7.328
7.311
7.296
7.263
7.250
2.950
2.928
2.907
1.987
1.975
1.963
1.952
1.940
1.706
1.695
1.686
1.675
1.498
$CO_2{}^tBu$
Ph
3
8.68
0.82
1.15
9.21
7.0
6.0
5.0
4.0
3.0
2.0
1.0

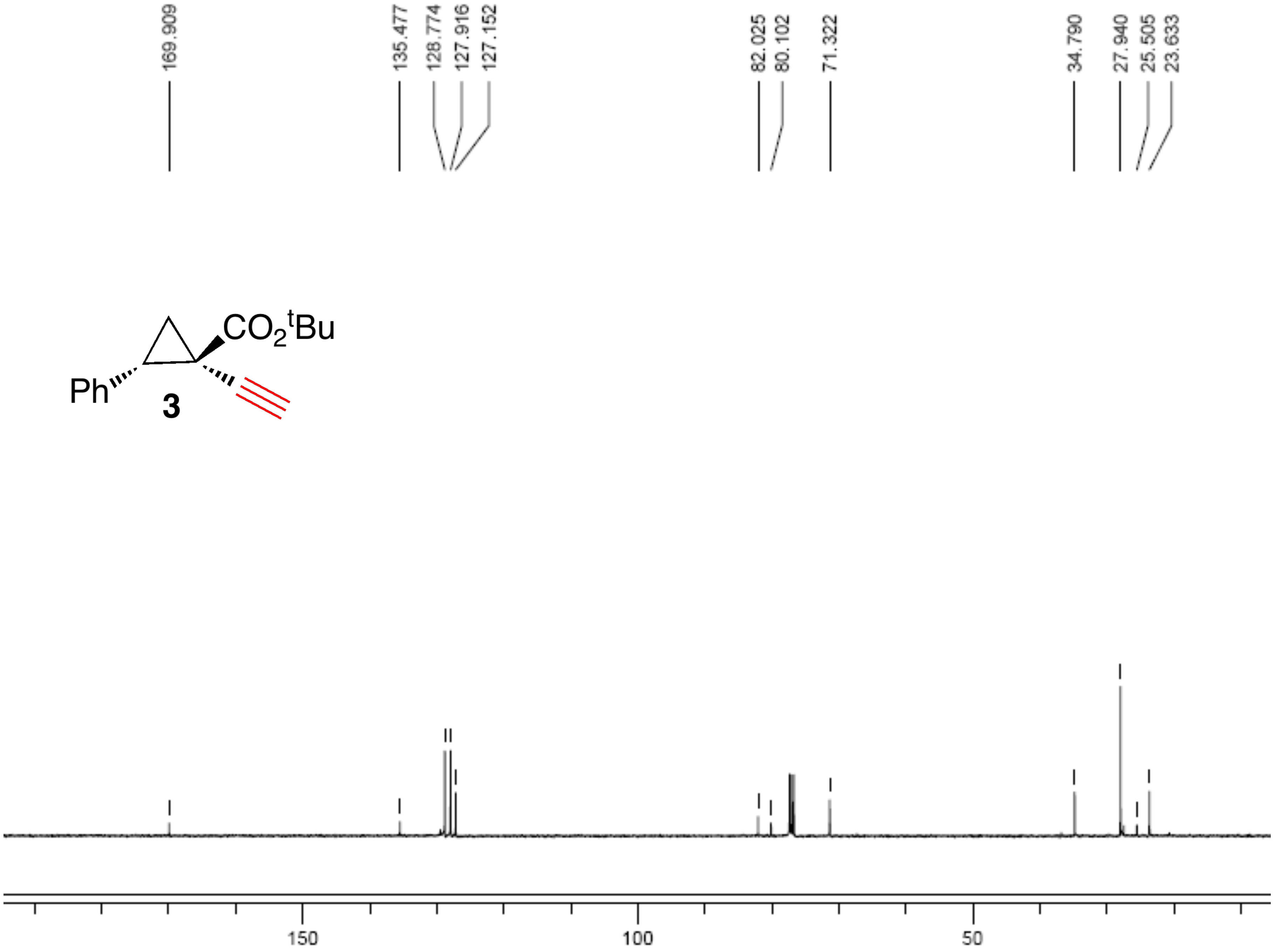
169.909
135.477
128.774
127.916
127.152
82.025
80.102
71.322
34.790
27.940
25.505
23.633
150
100
50
CO2tBu
Ph

3

xx-viii-112 ODH 0%@1.0 ml
C:\EZStart\Projects\Default\Method\Shifa-P-Cyclopropane 5%Whelk.met
C:\EZStart\Projects\Default\Data\xx-viii-112pure ODH 0%@1.0 ml again

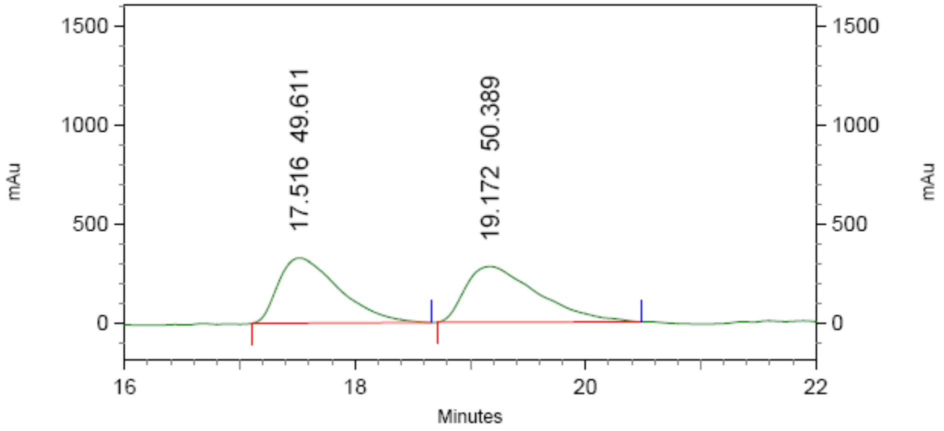

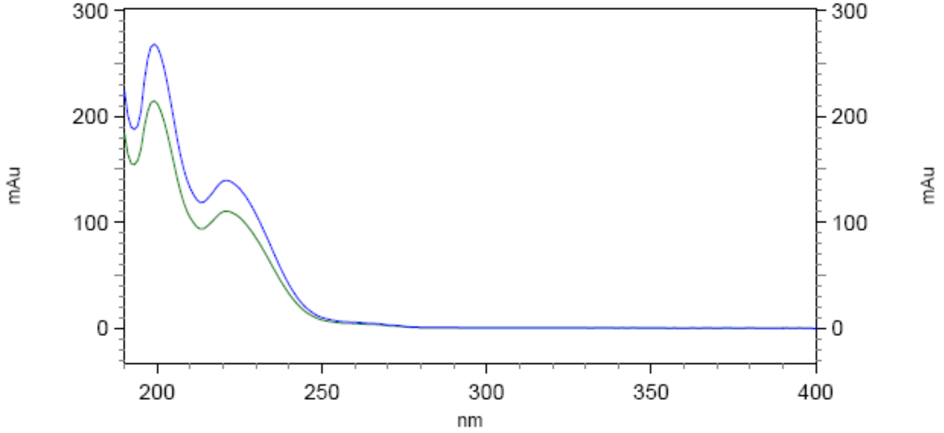

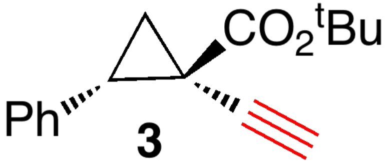

3

6: 207 nm, 4 nm
Results

Pk # Name	Retention Time	Area Percent
1	17.516	49.611
2	19.172	50.389
Totals		100.000

xx-viii-116pure ODH 0%@1.0 ml again
C:\EZStart\Projects\Default\Method\Shifa-P-Cyclopropane 5%Whelk.met
C:\EZStart\Projects\Default\Data\xx-viii-116pure ODH 0%@1.0 ml again

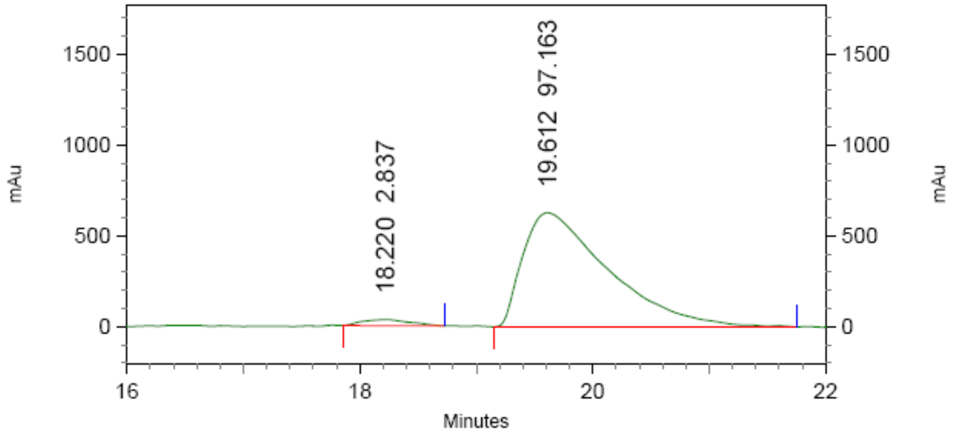

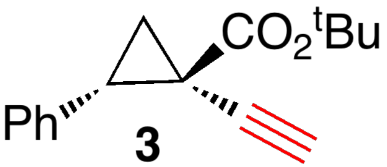

3

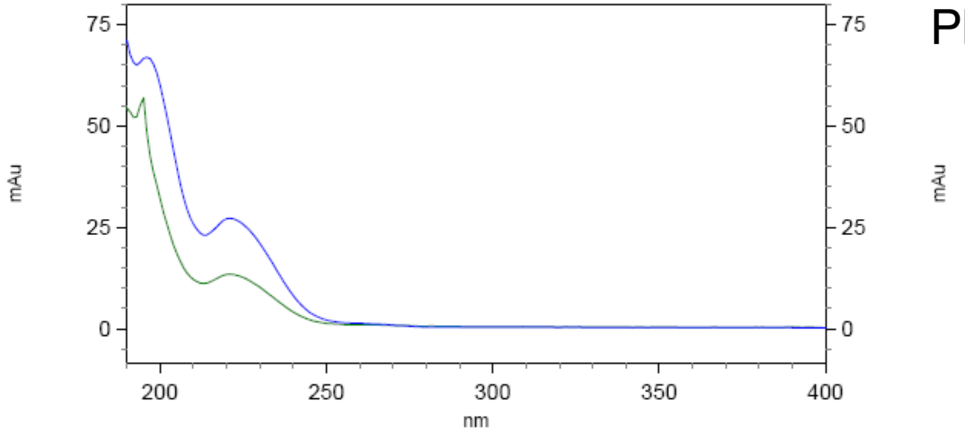

6: 207 nm, 4 nm
Results

Pk #	Name	Retention Time	Area Percent
1		18.220	2.837
2		19.612	97.163
Totals			100.000

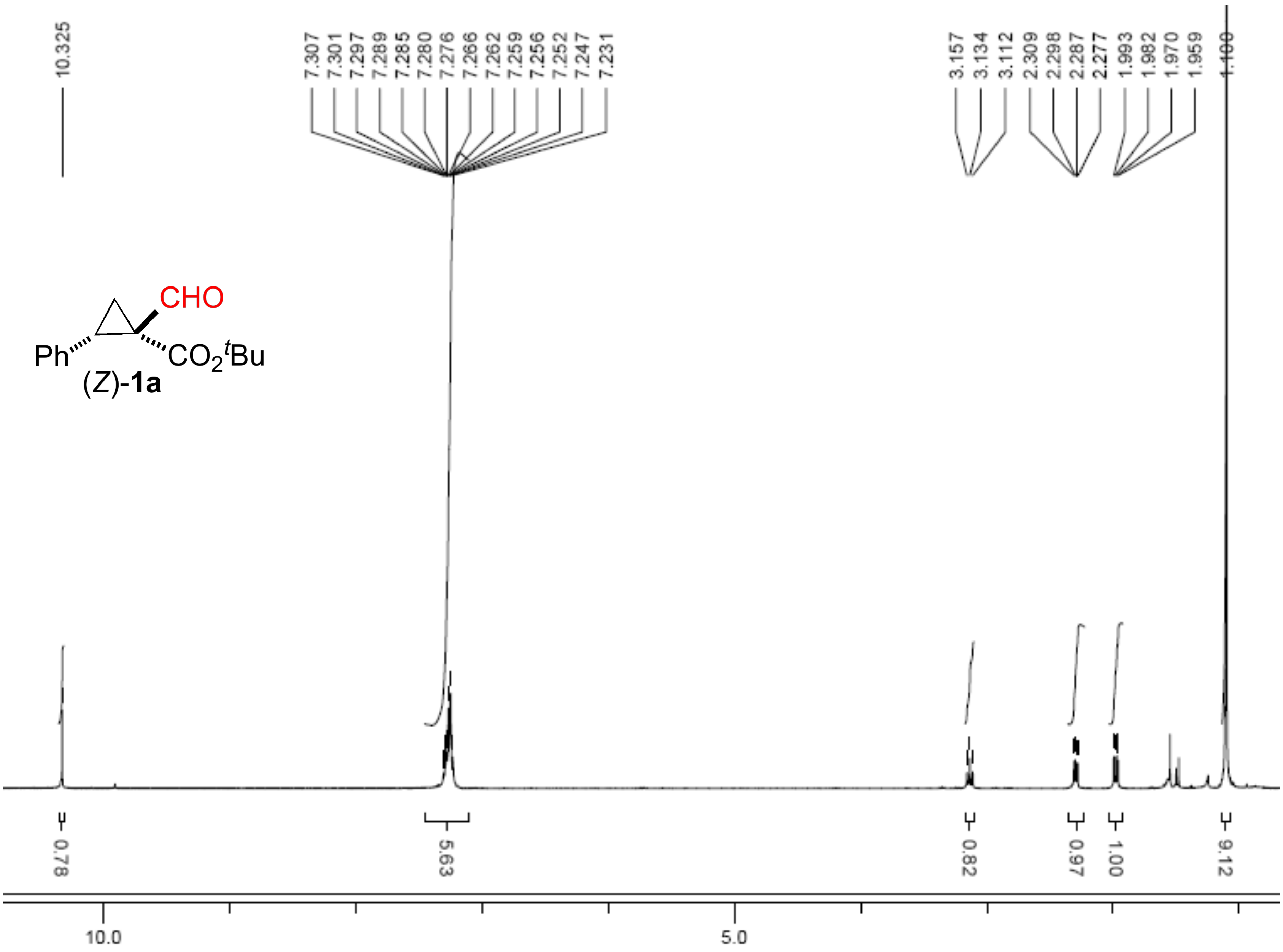
10.325
7.307
7.301
7.297
7.289
7.285
7.280
7.276
7.266
7.262
7.259
7.256
7.252
7.247
7.231
3.157
3.134
3.112
2.309
2.298
2.287
2.277
1.993
1.982
1.970
1.959
1.100
CHO
Ph
CO2tBu
(Z)-1a
0.78
5.63
0.82
0.97
1.00
9.12
10.0
5.0

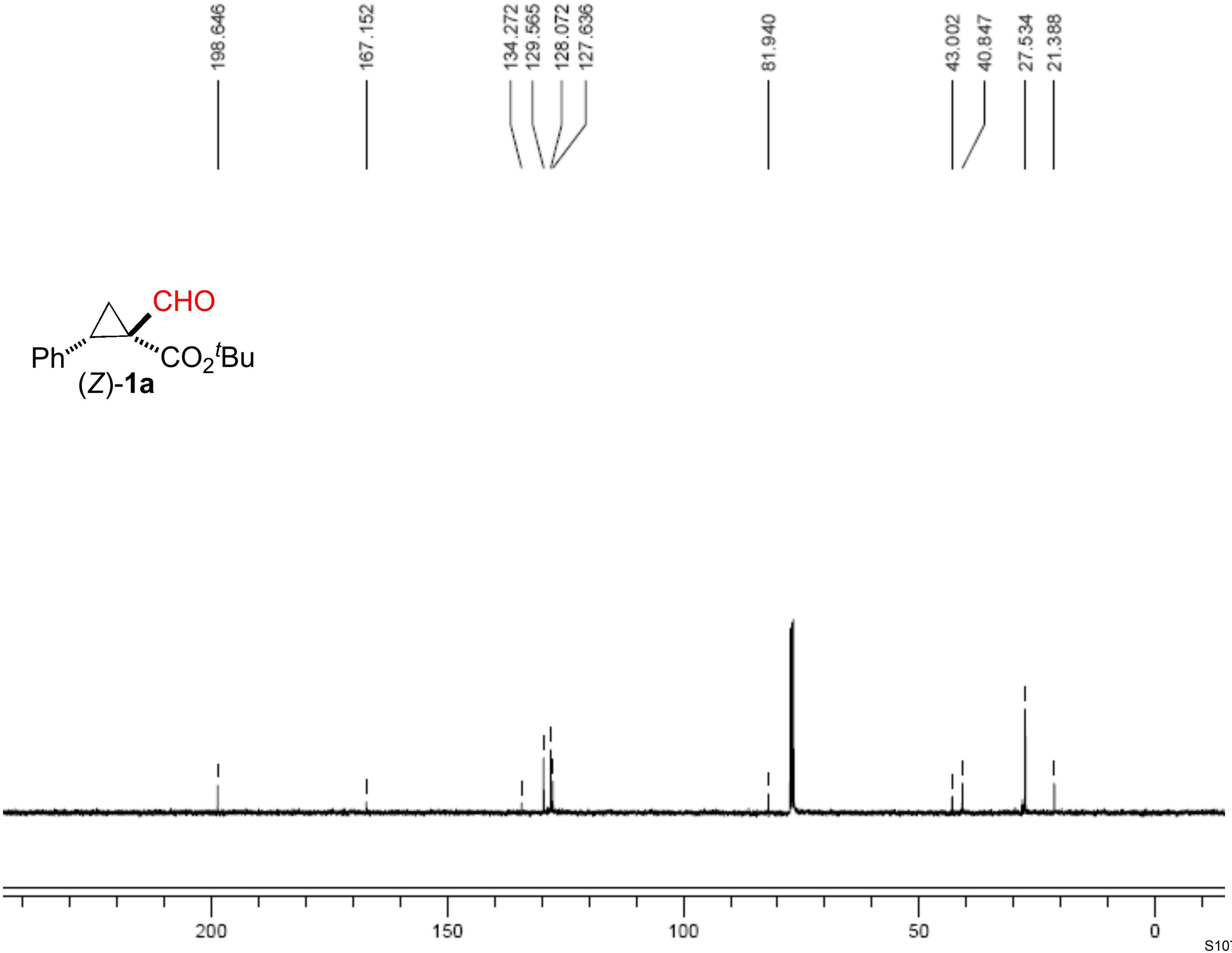
198.646
167.152
134.272
129.565
128.072
127.636
81.940
43.002
40.847
27.534
21.388
CHO
Ph
$CO_2{}^tBu$
(*Z*)-**1a**
200
150
100
50
0

Sample Name : **xx-viii-111 36 h DB 5**
Sample ID : **xx-viii-111 36 h DB 5**

Chromatogram

Chromatogram xx-viii-111 36 h DB 5 C:\GCMSsolution\Data\Project1\snow\xx-viii-111 36 h DB 5.qgd

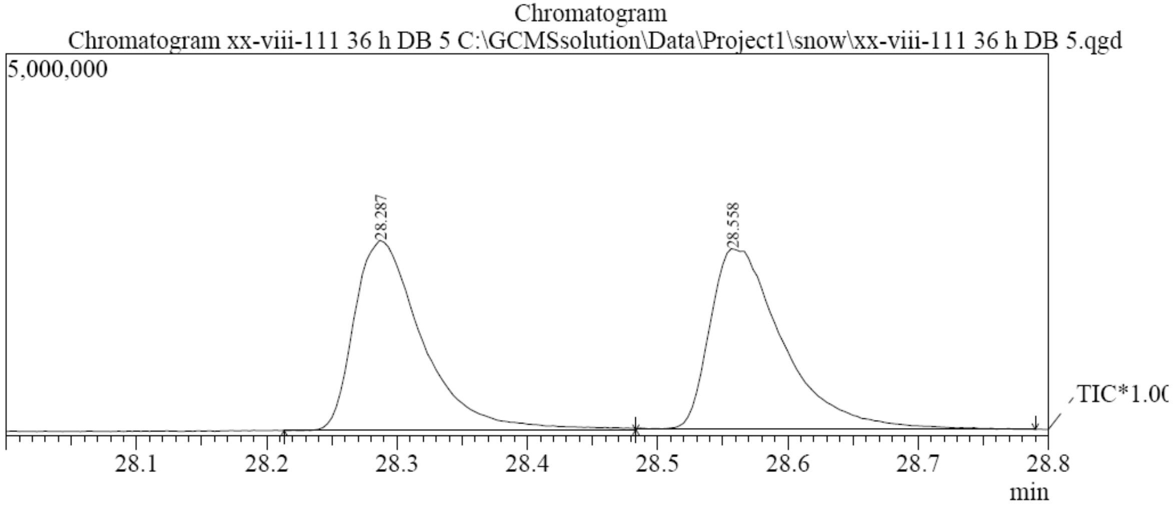

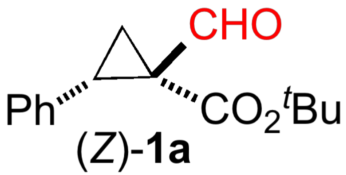

(*Z*)-**1a**

Peak Report TIC

Peak#	R.Time	Area%	Name	Base m/z
1	28.287	49.60		57.10
2	28.558	50.40		57.10
		100.00		

Spectrum

Peak#:1 R.Time:28.3(Scan#:6987)
MassPeaks:199

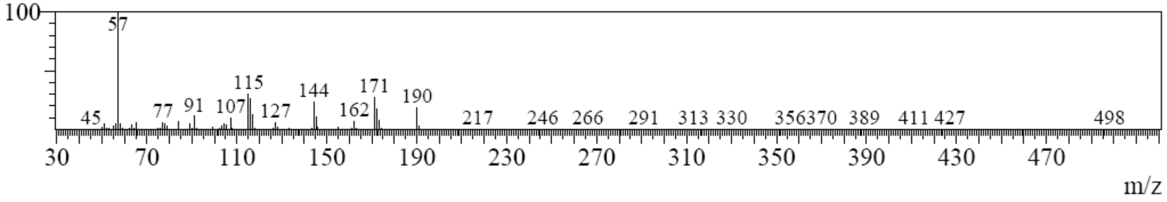

Peak#:2 R.Time:28.6(Scan#:7068)
MassPeaks:218

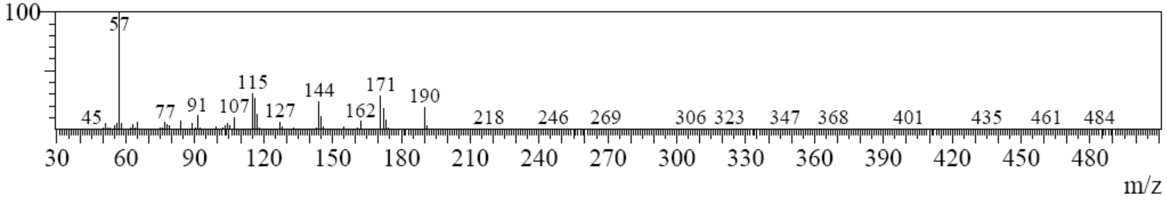

Sample Informatior

Sample Name : **xx-viii-115 DB 5**
Sample ID : **xx-viii-115 DB 5**

Chromatogram

Chromatogram xx-viii-115 DB 5 C:\GCMSsolution\Data\Project1\snow\xx-viii-115 DB 5.qgd

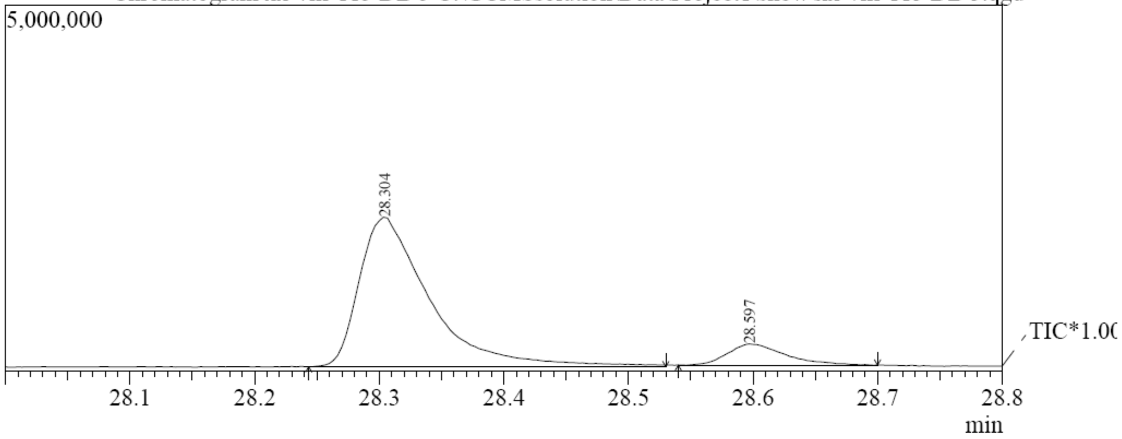

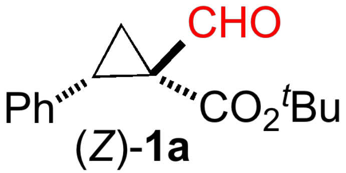

(*Z*)-**1a**

Peak Report TIC

Peak#	R.Time	Area%	Name	Base m/z
1	28.304	89.61		57.10
2	28.597	10.39		57.10
		100.00		

Spectrum

Peak#:1 R.Time:28.3(Scan#:6992)
MassPeaks:206

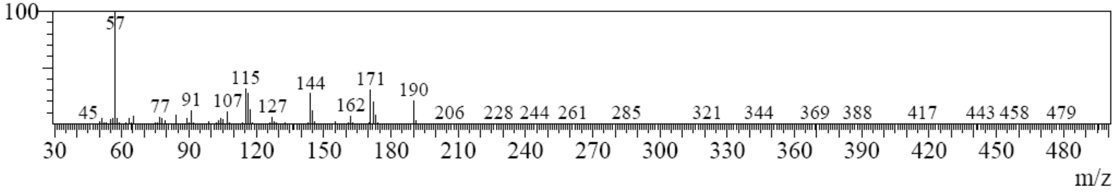

Peak#:2 R.Time:28.6(Scan#:7080)
MassPeaks:195

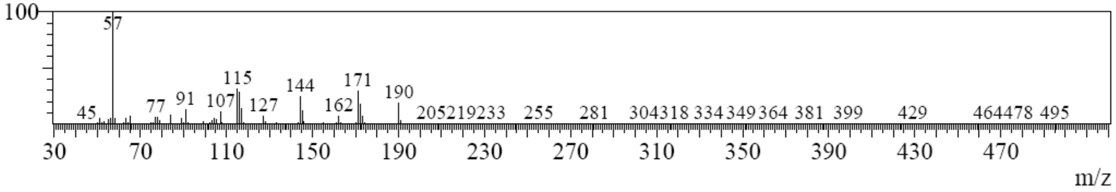

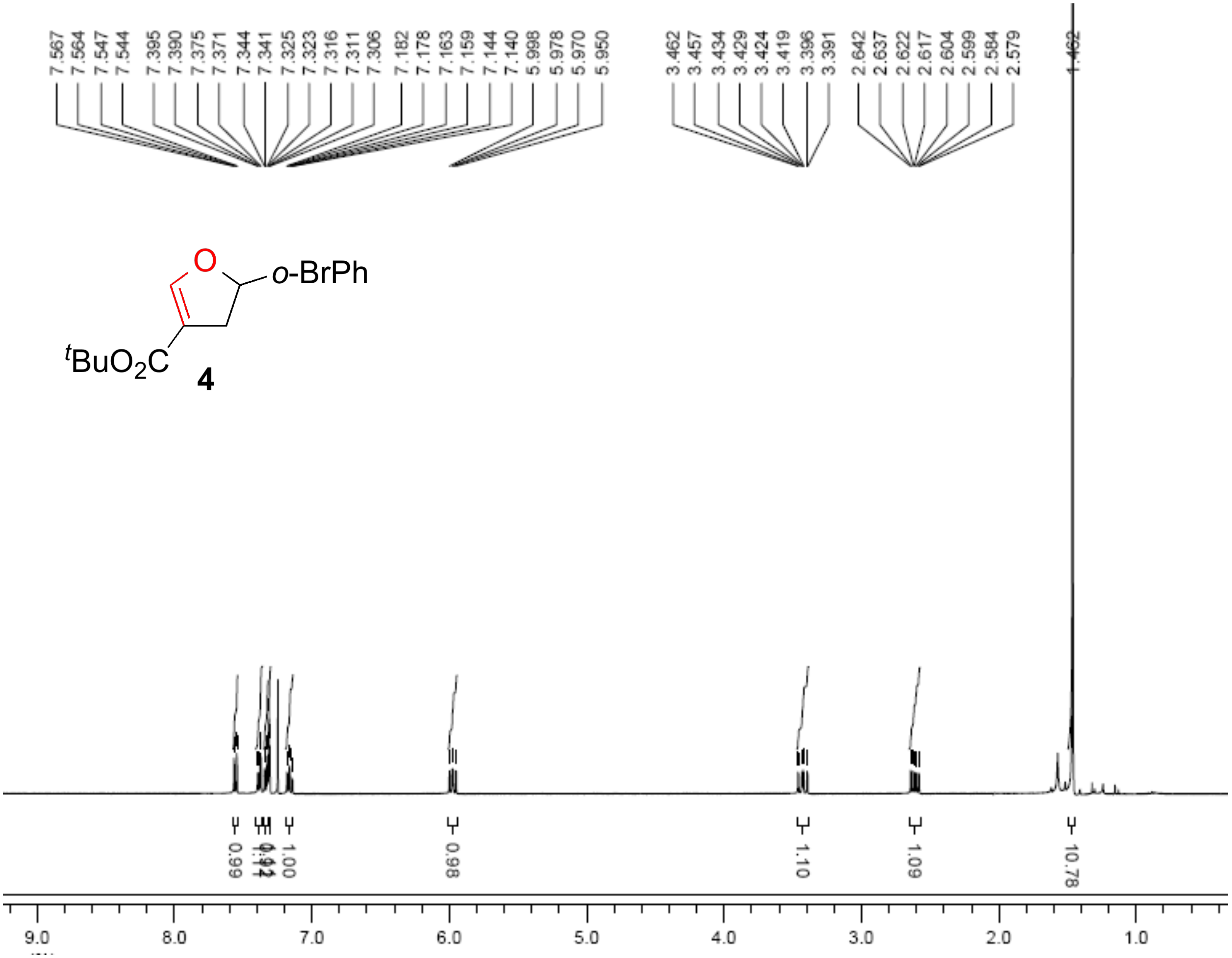
7.567
7.564
7.547
7.544
7.395
7.390
7.375
7.371
7.344
7.341
7.325
7.323
7.316
7.311
7.306
7.182
7.178
7.163
7.159
7.144
7.140
5.998
5.978
5.970
5.950
3.462
3.457
3.434
3.429
3.424
3.419
3.396
3.391
2.642
2.637
2.622
2.617
2.604
2.599
2.584
2.579
1.462
O
o-BrPh
$^tBuO_2C$
4
0.99
1.00
0.98
1.10
1.09
10.78
9.0
8.0
7.0
6.0
5.0
4.0
3.0
2.0
1.0

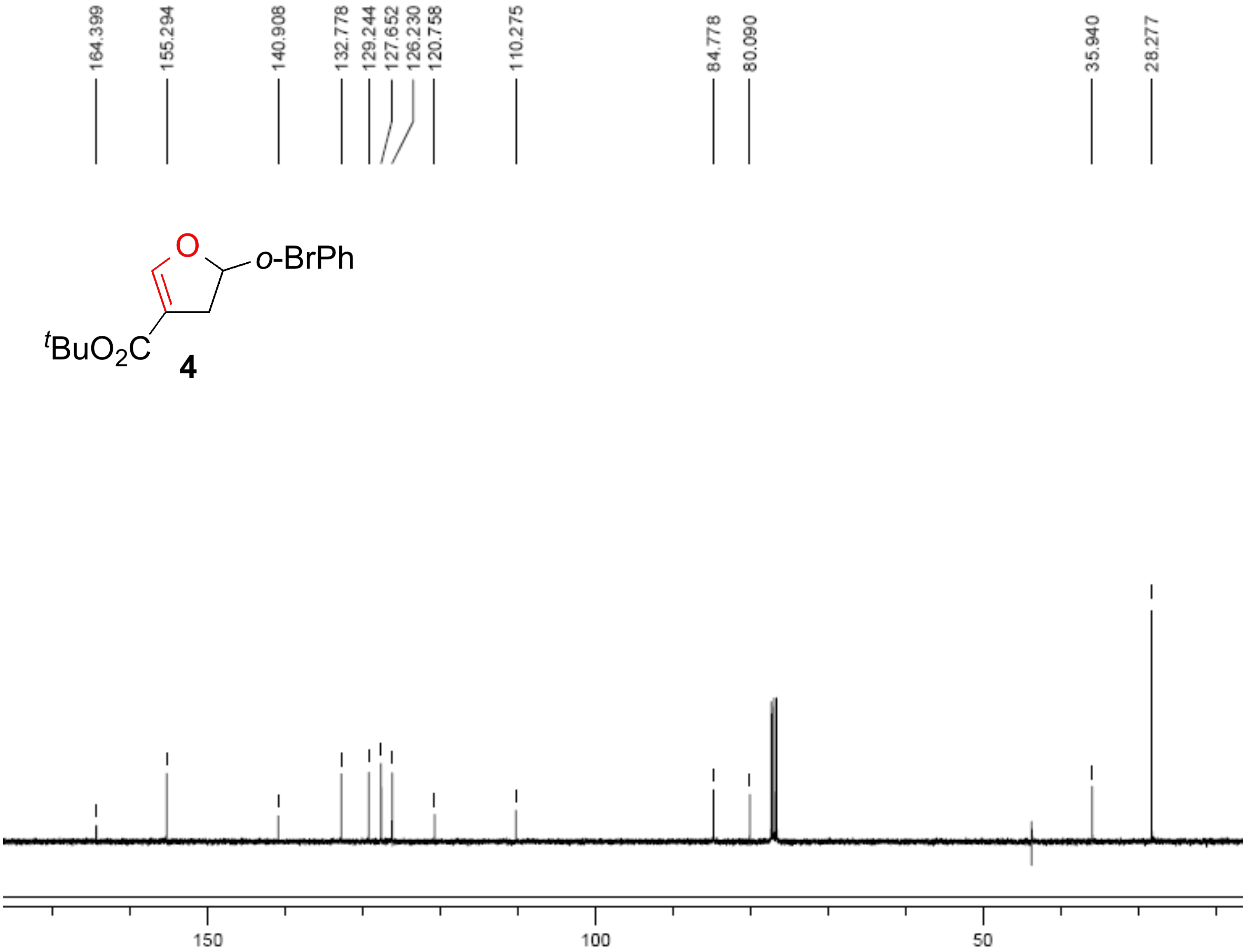

164.399
155.294
140.908
132.778
129.244
127.652
126.230
120.758
110.275
84.778
80.090
35.940
28.277
150
100
50
O
o-BrPh
tBuO2C

4

xx-viii-31-1 Whelk 1%@0.8 ml
C:\EZStart\Projects\Default\Method\snowtemp 2 .met
C:\EZStart\Projects\Default\Data\xx-viii-31-1 Whelk 1%@0.8 ml

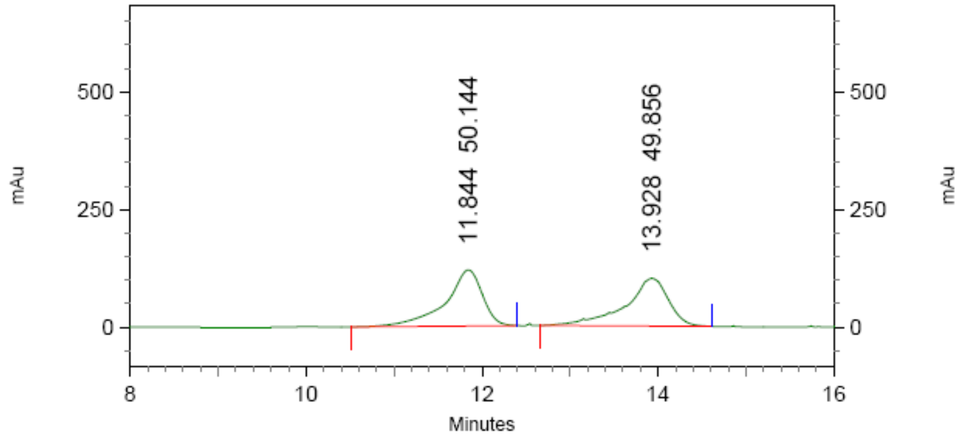

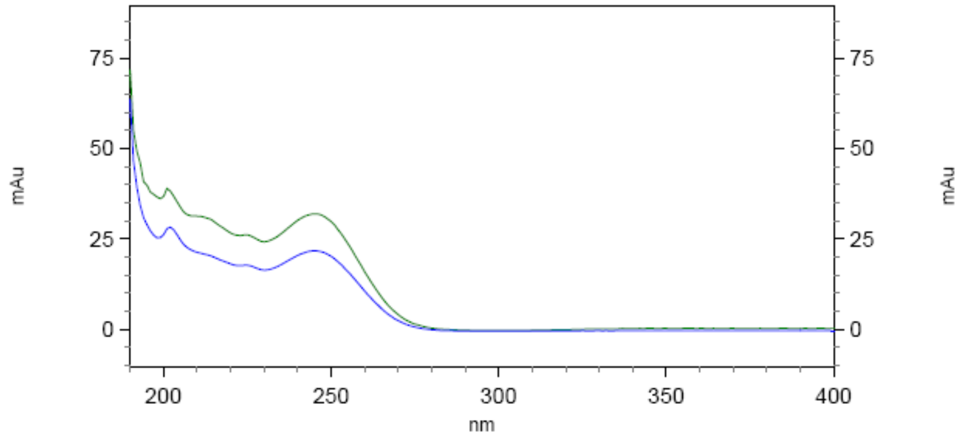

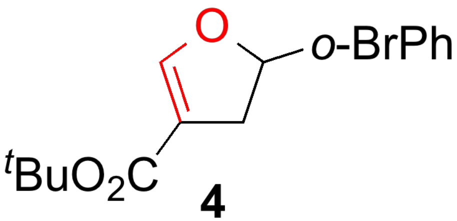

4

1: 247 nm, 4 nm
Results

Pk # Name	Retention Time	Area Percent
1	11.844	50.144
2	13.928	49.856
Totals		100.000

xx-viii-51-3 whelk 1%@0.8 ml
C:\EZStart\Projects\Default\Method\JTL-3%-ODH1ml.met
E:\xx-viii-51-3 whelk 1%@0.8 ml

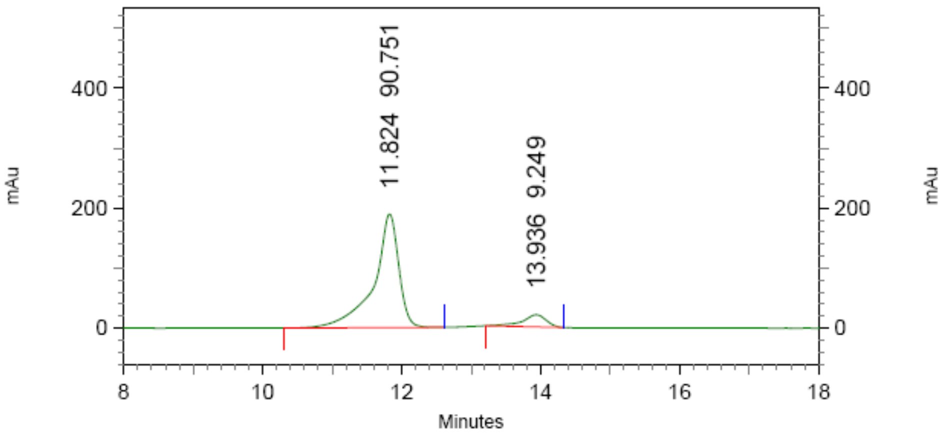

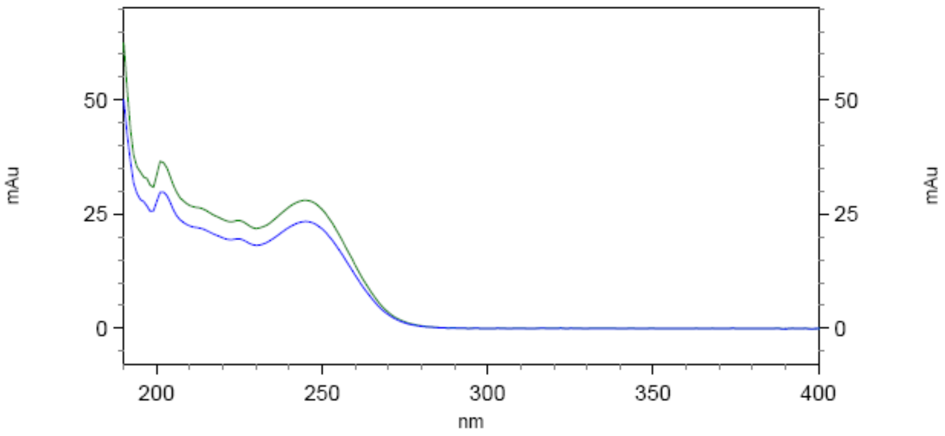

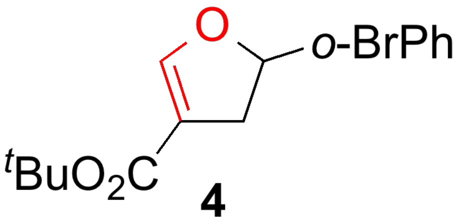

4

5: 228 nm, 4 nm

Results

Name	Retention Time	Area Percent	Pk #
	11.824	90.751	1
	13.936	9.249	2
Totals		100.000	
